# Sooty Shearwater Migration Optimizer: A Novel Metaheuristic Algorithm for Engineering Optimization and Photovoltaic Parameter Identification

**DOI:** 10.3390/biomimetics11100751

**Published:** 2026-10-18

**Authors:** Xiaohan Lin, Xiangkun Song, Jian Zhao

**Affiliations:** School of Science, University of Science and Technology Liaoning, Anshan 114051, China; linxiaohan@stu.ustl.edu.cn (X.L.); song@stu.ustl.edu.cn (X.S.)

**Keywords:** bio-inspired computation, optimization algorithm, swarm intelligence, engineering problems, photovoltaic parameter identification, numerical optimization

## Abstract

This paper develops a new metaheuristic optimizer, named the Sooty Shearwater Migration Optimizer (SSMO), by drawing inspiration from the long-distance migration patterns of sooty shearwaters. The proposed SSMO integrates elite guidance, wind-driven migration, molting memory guidance, and adaptive foraging to coordinate global exploration with local exploitation. Its optimization performance is assessed in the CEC2017 benchmark suite under 30D, 50D, and 100D settings and in the CEC2022 benchmark suite under 10D and 20D settings. Compared with 11 representative optimization algorithms, SSMO achieves the lowest average Friedman ranks in all five-dimensional settings. The Wilcoxon rank-sum results also generally favor SSMO. Additional comparisons with four differential evolution variants further demonstrate competitive performance under the tested configurations. Its practical applicability is further assessed using five engineering design problems, on which it obtains the lowest mean fitness values under the adopted evaluation settings. In addition, four photovoltaic parameter identification cases are used to evaluate its effectiveness in practical parameter estimation. Compared with the selected general-purpose algorithms, SSMO obtains the lowest error values for the considered evaluation metrics. Further comparison with four specialized photovoltaic parameter identification algorithms shows that SSMO remains competitive in most test cases. These findings suggest that SSMO is a promising optimizer for numerical benchmarks and practical engineering applications.

## 1. Introduction

Driven by carbon neutrality goals and the transition toward renewable energy, photovoltaic (PV) generation has become an important component of modern power systems. Accurate parameter identification is essential for PV modeling, performance evaluation, maximum power point tracking, and system optimization. However, key parameters, including photocurrent, saturation current, the diode ideality factor, series resistance, and shunt resistance, are difficult to measure directly. In practice, these parameters are usually estimated from measured current-voltage (I–V) data through inverse optimization [1]. Accurate and robust PV parameter identification therefore remains an important research topic [2].

PV parameter identification is commonly formulated as a constrained nonlinear optimization problem [3]. Its objective function is often nonconvex and multimodal. Measurement noise further increases uncertainty in the estimated parameters. Conventional gradient-based methods generally require derivative information and suitable initial values [4]. Their performance may deteriorate in the presence of local optima, flat regions, or strong coupling among parameters. These difficulties can reduce estimation accuracy and robustness under practical operating conditions.

Metaheuristic algorithms (MAs) provide a derivative-free approach to PV parameter identification because they can search nonlinear and multimodal solution spaces without requiring gradient information [5]. The search mechanisms employed by MAs vary considerably. Differential evolution (DE) is one of the most established population-based optimization methods and generates trial solutions using scaled difference vectors among population members [6]. Its representative variants include the self-adaptive differential evolution algorithm (SaDE), which adapts trial-vector generation strategies and control parameters according to previous search experience [7]; L-SHADE with ensemble sinusoidal parameter adaptation (L-SHADE-EpSin), which introduces an ensemble sinusoidal scheme for parameter control [8]; L-SHADE with semi-parameter adaptation hybridized with the covariance matrix adaptation evolution strategy (L-SHADE-SPACMA), which combines semi-parameter adaptation with covariance-based search [9]; and ring sub-population architecture-based differential evolution (RSDE), which organizes the population into interacting ring-connected subpopulations [10].

Other widely used metaheuristics generate search directions through different forms of population interaction. Particle swarm optimization (PSO) updates the velocity and position of each particle using its individual best experience and the best solution found by the swarm [11]. The grey wolf optimizer (GWO) guides candidate solutions using the three leading individuals in the population [12], whereas the whale optimization algorithm (WOA) combines encircling, spiral movement, and random exploration strategies [13]. More recently, the dung beetle optimizer (DBO) introduces role-specific rolling, breeding, foraging, and stealing behaviors to diversify the population search process [14]. These algorithms illustrate the diversity of existing population-based search mechanisms, including differential information, individual memory, multi-leader guidance, trajectory-based movement, and behavioral role division.

Metaheuristic methods have also been widely applied to PV parameter identification. PSO and its variants have been used to estimate unknown PV model parameters from measured I–V data [15,16]. More recently, the snow ablation optimizer (SAO) has been applied to improve parameter-identification accuracy [17]. Rank-improved teaching–learning-based optimization (RITLBO) introduces ranking and learning strategies to improve solution quality and robustness [18]. The multi-strategy synergistic learning snake optimizer (MSSO) combines several search strategies to preserve population diversity and strengthen local refinement [19], while the beaver behavior optimizer (BBO) employs behavior-inspired search operators to obtain competitive parameter estimates [20]. Table 1 summarizes representative algorithms that employ search mechanisms closely related to the present study or have been specifically applied to PV parameter identification.

Despite the reported progress, several issues remain. First, insufficient exploration may reduce population diversity and lead to premature convergence [21]. Second, some methods combine multiple search operators, control parameters, and switching rules, which can increase implementation and parameter-tuning complexity without necessarily ensuring consistent performance improvements [22]. Third, conclusions regarding algorithm performance can depend on the selected benchmark functions, competing methods, and experimental protocols [23]. Therefore, strong performance on a single benchmark suite or PV model does not by itself establish general effectiveness across problems with different dimensions and levels of complexity. These issues are particularly important for PV parameter identification because its objective landscape is nonlinear, multimodal, and strongly coupled [24,25]. Consequently, there remains a need for an optimizer that preserves useful population diversity while providing accurate local refinement and stable convergence across different PV models.

Motivated by these limitations, this study proposes the Sooty Shearwater Migration Optimizer (SSMO). The algorithm integrates four complementary search mechanisms. Elite guidance combines information from multiple high-quality individuals to reduce excessive dependence on a single leader. Wind-driven migration introduces differential information between population members to expand the search range. Molting memory guidance uses the best previously visited position to support local refinement, while adaptive foraging introduces fitness-dependent perturbations to maintain search flexibility. Their respective contributions are examined through an ablation study. The overall performance of SSMO is further evaluated using the CEC2017 and CEC2022 benchmark suites, representative adaptive DE variants, constrained engineering design problems, and four PV parameter-identification models.

The main contributions of this work are summarized as follows:

(1) A new bio-inspired metaheuristic, termed the Sooty Shearwater Migration Optimizer (SSMO), is developed. It integrates elite guidance, wind-driven migration, molting memory guidance, and adaptive foraging to improve population diversity, search accuracy, and convergence stability.
(2) The numerical performance of SSMO is systematically evaluated in the CEC2017 and CEC2022 benchmark suites in different dimensions. Ablation experiments examine the contribution of each search mechanism, and comparisons with DE and its adaptive variants are conducted.
(3) Five representative engineering optimization problems, including four constrained design problems and one unconstrained discrete problem, are used to further assess the practical effectiveness of SSMO.
(4) SSMO is applied to parameter identification for the SDM, DDM, TDM, and PVM. Its performance is compared with both general-purpose optimizers and recently reported PV parameter-identification methods under consistent experimental conditions, and the differences are further examined using statistical significance tests.

The remainder of this paper is organized as follows: Section 2 presents the biological inspiration and mathematical formulation of SSMO. Section 3 evaluates its numerical optimization performance in the CEC2017 and CEC2022 benchmark suites, including ablation analysis and comparisons with representative DE variants. Section 4 examines its performance on constrained engineering design problems. Section 5 applies SSMO to parameter identification for SDM, DDM, TDM, and PVM. Section 6 concludes the paper and outlines possible directions for future research.

## 2. Sooty Shearwater Migration Optimizer

### 2.1. Inspiration

**Figure 1:**
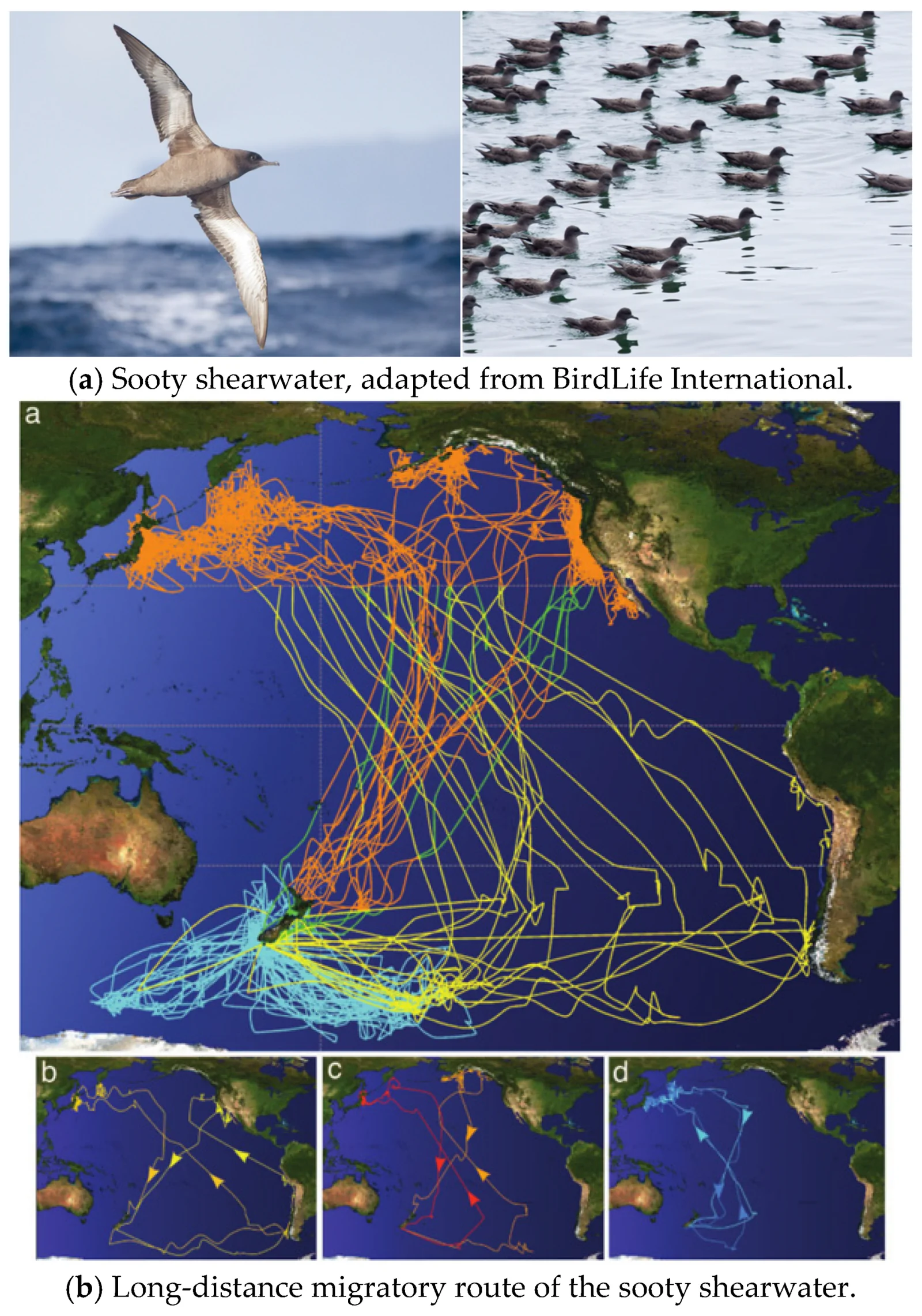
Sooty shearwater and its long-distance migratory route. Light blue indicates breeding-season movements, yellow northward migration, and orange North Pacific wintering areas and southward migration.

The sooty shearwater (*Ardenna grisea*) is a pelagic seabird known for its exceptionally long migrations and flexible foraging behavior. A schematic illustration of the species is shown in Figure 1a [26,27]. Its trans-equatorial migration allows it to exploit seasonally productive marine regions in both hemispheres. During the austral summer, the productive waters of the Southern Ocean and subantarctic regions provide important foraging habitats. During the boreal summer, high-latitude waters of the North Pacific serve as major non-breeding and feeding grounds [28,29]. The annual migration follows a characteristic figure-eight route across the Pacific Ocean and covers approximately 64,000 km, as shown in Figure 1b [28].

During migration, sooty shearwaters appear to use large-scale atmospheric circulation systems, particularly prevailing westerlies and easterly trade winds. These winds may facilitate rapid movement between the Northern and Southern Hemispheres [28]. After reaching the North Pacific, tracked individuals remain within one of three distinct non-breeding regions throughout the season [29]. Similar trans-equatorial migrations and the use of a probable northern molting area have also been reported for South Atlantic populations [30]. During breeding, Pacific populations alternate between short trips to nearby shelf waters and longer trips to distant oceanic foraging grounds [31], while Southwest Atlantic populations exhibit habitat-dependent foraging and diving patterns [32]. These observations indicate that their movement is influenced by atmospheric conditions, spatially distributed resources, and changes in foraging scale.

These biological observations provide the general inspiration for SSMO. Seasonal movement toward productive regions motivates the use of promising solutions as search references, while the influence of prevailing winds motivates a migration direction constructed from differences between population members. The global best-so-far position used in the molting memory mechanism is introduced as a computational memory and does not represent a biologically documented memory of an optimal molting location. Similarly, the fitness-dependent perturbation used in adaptive foraging is an optimization abstraction of the transition between broad and localized search. The following subsections distinguish the biological observation underlying each mechanism from the computational operators used to implement it.

### 2.2. Mathematical Model

In SSMO, the general biological observations described above are translated into four computational search mechanisms. Elite guidance uses several high-quality individuals as reference solutions to direct the population toward promising regions. Wind-driven migration introduces differences between population members to construct a stochastic migration direction. Molting memory guidance uses the global best-so-far position as additional search information, while adaptive foraging applies fitness-dependent perturbations to adjust the search scale. Their mathematical definitions are presented in the following subsections.

#### 2.2.1. Initial Population

As with most population-based MAs, SSMO begins by randomly generating a population of search agents within the feasible search space, where each agent corresponds to a sooty shearwater individual. The population is defined as:

(1) $$S=\left[\begin{array}{c} S_{1}\\ \begin{array}{c} S_{2}\\ \begin{array}{c} \vdots \\ \begin{array}{c} S_{i}\\ \begin{array}{c} \vdots \\ S_{N} \end{array} \end{array} \end{array} \end{array} \end{array}\right]=\left[\begin{array}{cccccc} s_{1,1} & s_{1,2} & \ldots & s_{1,j} & \ldots & s_{1,D}\\ s_{2,1} & s_{2,2} & \ldots & s_{2,j} & \ldots & s_{2,D}\\ \vdots & \vdots & \ddots & \vdots & \ddots & \vdots \\ s_{i,1} & s_{i,2} & \ldots & s_{i,j} & \ldots & s_{i,D}\\ \vdots & \vdots & \ddots & \vdots & \ddots & \vdots \\ s_{N,1} & s_{N,2} & \ldots & s_{N,j} & \ldots & s_{N,D} \end{array}\right],\left\{\begin{array}{c} i=1,2,...,N\\ j=1,2,...,D \end{array}\right.$$

where *S* ∈ *R^N^*^×*D*^ denotes the population matrix, *N* is the population size, and *D* is the problem dimension. Each row vector *S_i_* represents the position of the *i*-th search agent.

The initial population is generated as:

*S_i_* = *lb* + *rand*(1, *D*) ⊙ (*ub* − *lb*), *i* = 1, 2, …, *N* (2)

where *lb* and *ub* denote the lower and upper bounds of the search space, respectively. rand(1, *D*) is a *D*-dimensional random vector whose components are uniformly distributed in 0, 1, and ⊙ denotes the Hadamard product.

#### 2.2.2. Fitness Ranking and Migration Capability Division

At the beginning of each iteration, all individuals are sorted in ascending order of fitness:

*f*(*S*_[1]_) ≤ *f*(*S*_[2]_) ≤ ⋯ ≤ *f*(*S*_[*N*]_) (3)

where the square-bracket subscript [·] denotes the fitness rank. Therefore, *S*_[1]_ is the best individual in the current population, whereas *S*_[*N*]_ is the worst individual.

The numbers of elite individuals and promising reference individuals are defined as:

*L* = *max*(2, *round*(0.15*N*)),     *M* = *max*(5, *round*(0.6*N*)) (4)

According to the fitness ranking:

- Individuals *S*_[1]_, …, *S*_[*L*]_ form the elite group;
- Individuals *S*_[*L*+1]_, …, *S*_[*M*]_ form the main group;
- Individuals *S*_[*M*+1]_, …, *S*_[*N*]_ form the weak group.

The first *M* ranked individuals, including both the elite group and the main group, constitute the promising reference pool used by the wind-driven migration mechanism.

This division is dynamically updated rather than permanently assigned. Because the population is ranked again at every iteration, an individual can enter or leave each group according to the fitness of its current position. In SSMO, “migration capability” is an optimization term defined by the current fitness ranking and does not represent a documented biological classification or a permanent identity of an individual bird.

#### 2.2.3. Elite Guidance Mechanism

During long-distance migration, sooty shearwaters move toward seasonally productive regions. This observation provides the general inspiration for using promising positions as search references. In SSMO, favorable migration routes are computationally represented by high-ranking solutions, and directional information from multiple elite individuals is aggregated to reduce excessive dependence on the current best solution.

For the *i*-th individual, the elite guidance vector, rank-based elite weight, and stochastic activity factor are defined as:

(5) $$E_{i}(t)=\gamma_{i}\times \sum_{k=1}^{L}\omega_{k}\times \frac{S_{\left[k\right]}(t)-S_{i}(t)}{{\left|\left|S_{\left[k\right]}(t)-S_{i}(t)\right|\right|}_{2}+\varepsilon }$$

(6) $$\omega_{k}=e^{-\frac{k}{L}},\;\;\;\;\;\;k=1,2,...,L$$

(7) $$\gamma_{i}=\frac{3}{5}+\frac{4}{5}\cdot r_{i},\;\;\;\;\;\;r_{i}∼U(0,1)$$

where *E_i_*(*t*) denotes the aggregated elite guidance vector of the *i*-th individual, and *S*_[*k*]_(*t*) represents the individual ranked *k*-th in the current population. The difference *S*_[*k*]_(*t*) − *S_i_*(*t*) determines the direction from the current individual toward the *k*-th elite individual. The Euclidean norm in the denominator normalizes this direction, while *ε* = 10^−15^ prevents division by zero.

The coefficient *ω_k_* represents the influence weight of the *k*-th elite individual. Its value decreases exponentially with the fitness rank. Consequently, higher-ranked individuals exert stronger guidance, whereas lower-ranked elite individuals still provide supplementary migration directions. This weighting scheme enables SSMO to utilize several promising routes without assigning equal confidence to all elite individuals.

The stochastic activity factor *γ_i_* ∈ [0.6, 1.4] is a scalar coefficient used to adjust the magnitude of the aggregated elite guidance vector. A smaller value produces a shorter displacement, whereas a larger value produces a stronger displacement. The random variable *r_i_* is independently sampled from *U*(0, 1) for each individual at every iteration.

The elite guidance mechanism combines several normalized directions instead of relying only on the current best solution. Higher-ranked individuals provide stronger guidance through the rank-based weights, while the other elite individuals retain additional search directions.

#### 2.2.4. Wind-Driven Migration Mechanism

Sooty shearwaters use large-scale atmospheric circulation systems, particularly the westerlies and trade winds, during long-distance migration [29]. Wind conditions vary across regions, and their effects on flight direction and distance are not constant. Rather than directly modeling the physical properties of wind, SSMO translates this observation into a stochastic displacement constructed from the difference between a promising individual and a randomly selected individual.

The wind-driven vector, wind intensity coefficient, and random reference indices are defined as:

(8) $$W_{i}\left(t\right)=\eta_{i}\left[S_{\left[r_{2}\right]}\left(t\right)-S_{\left[r_{3}\right]}\left(t\right)\right]$$

(9) $$\eta_{i}=\frac{1}{2}+\sigma_{\eta }z_{i},\;\;\;\;\;\;z_{i}∼N\left(0,1\right)$$

*r*_2_ ∼ *U*_int_{1,2, …, *M*},      *r*_3_ ∼ *U*_int_{1,2, …, *N*} (10)

where *U_int_* denotes a discrete uniform distribution over an integer set. The individual $S_{r_{2}}$ is randomly selected from the first *M* ranked individuals, while $S_{r_{3}}$ is selected from the complete population. Their difference defines a displacement from a population-wide position toward a comparatively promising position.

The coefficient *η_i_* controls the magnitude of the wind-driven displacement. It follows a normal distribution with a mean of 0.5 and a standard deviation of *σ_η_*. In this study, *σ_η_* is set to 0.10. Most values of *η_i_* therefore remain close to 0.5. Smaller values weaken the displacement, whereas larger values increase the migration distance. This distribution introduces controlled variation while avoiding excessively large wind-driven steps.

Equation (8) uses the general differential-vector principle of DE [6], but it does not constitute a conventional DE mutation operator. In DE, a scaled difference between two population members is added to a base vector, followed by crossover and selection. SSMO does not employ this complete mutation-crossover procedure. Instead, one endpoint of the differential vector is selected from the first *M* ranked individuals, whereas the other is selected from the entire population. The scaling coefficient *η_i_* follows $N\left(0.5,\sigma_{\eta }^{2}\right)$. The resulting vector is incorporated into Equation (13) as a wind-driven displacement together with elite guidance and molting memory guidance. Thus, the mathematical distinction lies in the rank-biased construction of the differential vector and its integration with the other guidance components of SSMO.

**Figure 2:**
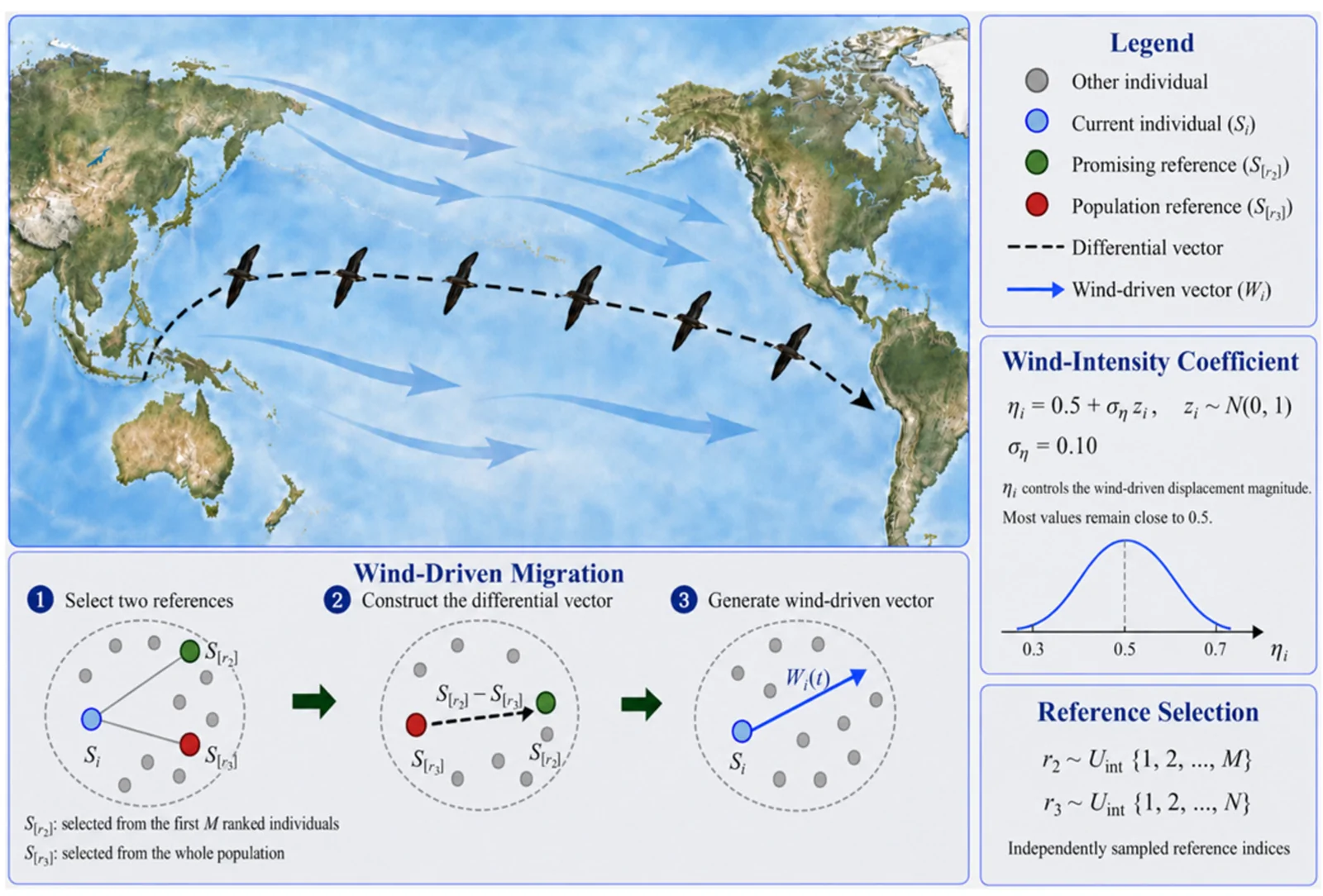
Schematic of the wind-driven migration mechanism.

This formulation retains the directional and distance information contained in population differences while biasing the movement toward promising regions. Selecting one endpoint from the entire population preserves information from individuals with different fitness levels. The stochastic coefficient provides controlled variation in migration distance. A schematic illustration of wind-driven migration, prepared with the assistance of AI tools, is shown in Figure 2.

#### 2.2.5. Molting Memory Guidance Mechanism

Tracking studies show that sooty shearwaters occupy distinct non-breeding regions in the North Pacific [29], and a probable northern molting area has also been reported for South Atlantic populations [30]. These observations provide the general inspiration for retaining information obtained from previously visited positions. In SSMO, this idea is implemented through a best-so-far archive. The archive is a computational memory and does not imply that sooty shearwaters identify and memorize an optimal molting location.

The molting memory vector and its attraction coefficient are defined as:

*G_i_* = *ψ_i_* ⋅ [*S_best_*(*t*) − *S_i_*(*t*)] (11)

(12) $$\psi_{i}=\frac{4}{5}+\frac{3}{5}\cdot r_{4},\;\;\;\;\;\;r_{4}∼U(0,1)$$

where S*_best_*(*t*) denotes the best solution found from initialization to iteration *t*, rather than only the best individual in the current population. The difference S*_best_*(*t*) − S*_i_*(*t*) determines the search direction from the current position toward the best-so-far solution. The random variable *r*_4_ is independently sampled for each individual at every iteration.

The coefficient *ψ_i_* ∈ [0.8, 1.4] controls the response to historical information. A value of *ψ_i_* < 1 produces weaker attraction toward the best-so-far position, whereas *ψ_i_* > 1 amplifies the memory displacement.

**Figure 3:**
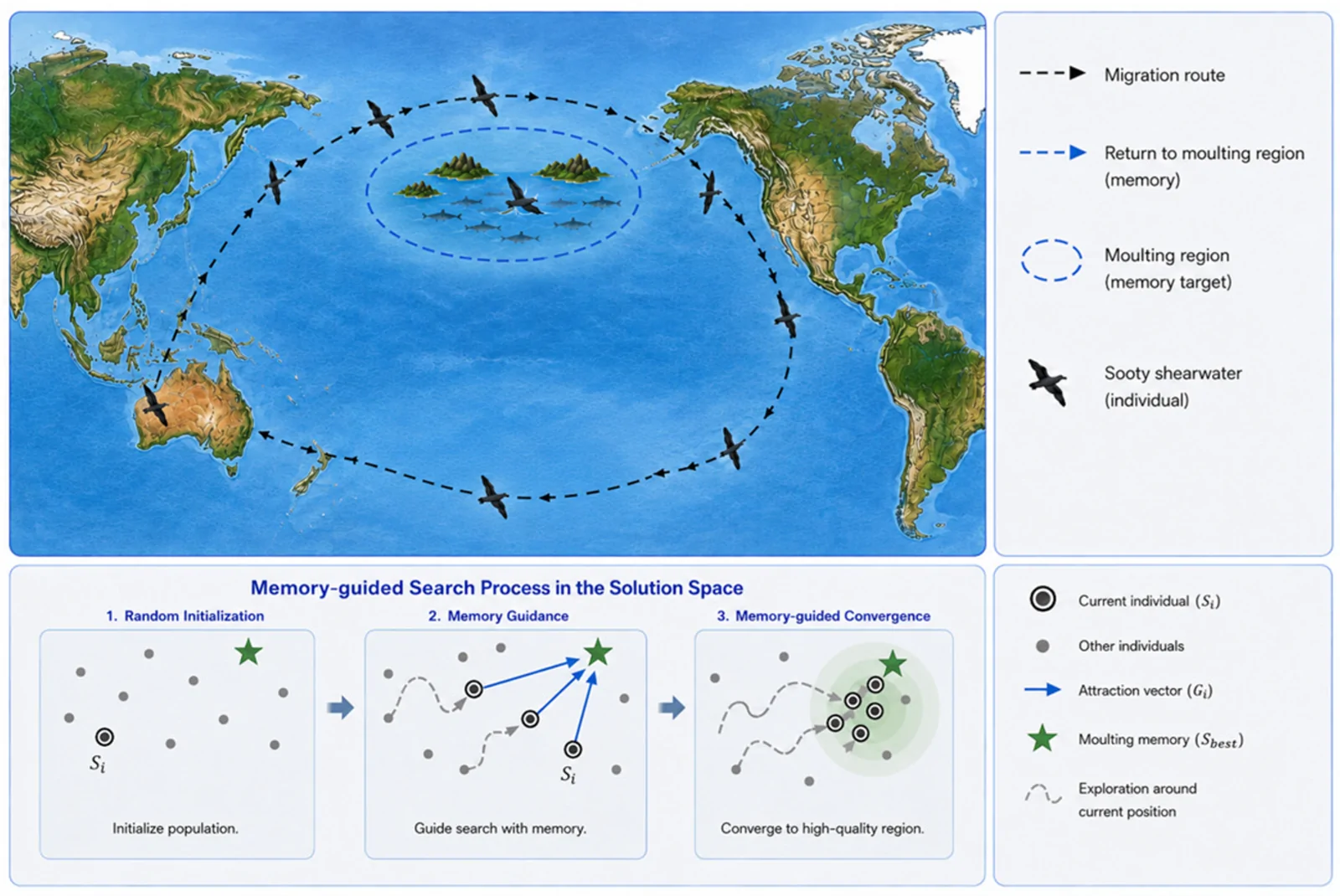
Schematic of the molting memory guidance mechanism.

The population-level molting memory guidance mechanism is illustrated in Figure 3.

#### 2.2.6. Migration Position Update

Following the definitions of the three search components, SSMO combines elite guidance, wind-driven migration, and molting memory guidance to update the migration position of each individual.

The migration position and the balance factor are defined as:

(13) $$\tilde{S_{i}}\left(t+1\right)=S_{i}\left(t\right)+\mu E_{i}+\left(1-\mu \right)G_{i}+W_{i}$$

(14) $$\mu =1-{\left(\frac{t}{T_{\max}}\right)}^{2}$$

where ${\tilde{S}}_{i}\left(t+1\right)$ denotes the temporary position obtained after the migration update and before the adaptive foraging operation. The vectors *E_i_*, *G_i_*, and *W_i_* represent elite guidance, molting memory guidance, and wind-driven movement, respectively. The variables *t* and *T_max_* denote the current iteration and the maximum number of iterations, respectively. The complementary weights *μ* and 1 − *μ* control the relative contributions of elite guidance and molting memory guidance. The wind-driven vector *W_i_* is not weighted by *μ* and therefore remains active throughout the search.

The balance factor *μ* follows a parameter-free quadratic schedule based on the normalized iteration progress *t*/*T_max_*. At the beginning of the search, *μ* is close to one, and a larger weight is therefore assigned to the multi-elite guidance term. Because the squared normalized iteration progress remains small during the early stage, *μ* decreases slowly, allowing the population to use information from multiple promising solutions for a longer period. As the search proceeds, the decrease in *μ* becomes faster, while the weight 1 − *μ* assigned to the molting memory guidance increases accordingly. This change gradually shifts the main guidance source from the current elite group to the best-so-far solution.

Compared with a linear decreasing schedule, the quadratic form maintains a larger value of *μ* during the early and middle stages. The two weights become equal when $t/T_{\max}=1/\sqrt{2}\approx 0.707$, rather than at the midpoint of the search. After this point, the contribution of the best-so-far direction increases rapidly, supporting stronger refinement during the later stage. The quadratic form is selected because it provides a smooth and delayed transition between the two guidance components without introducing an additional control parameter.

**Figure 4:**
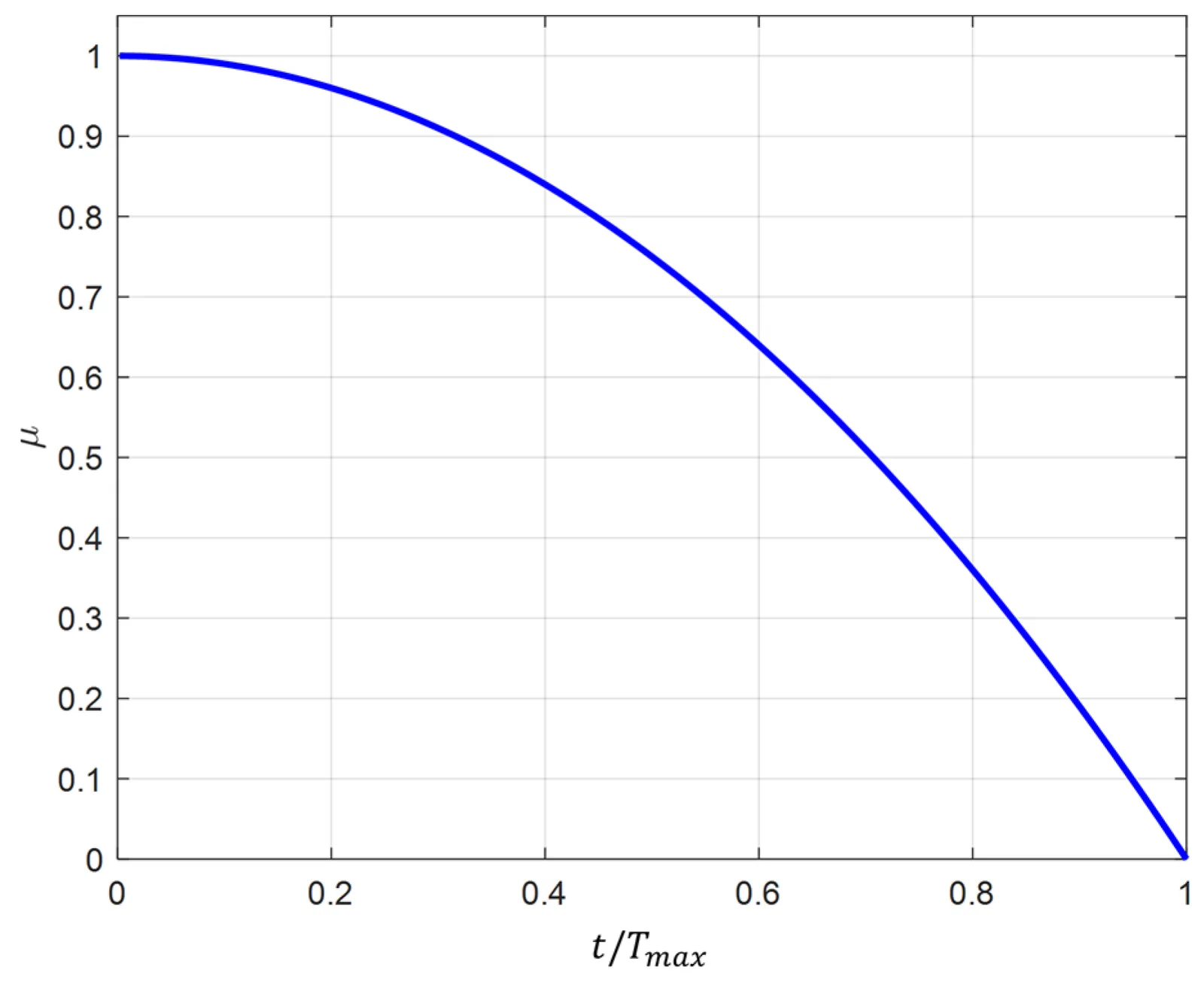
Evolution of the parameter *μ*.

The variations of *μ* and 1 − *μ* over the normalized iteration progress are illustrated in Figure 4. The wind-driven vector W*_i_* is not weighted by *μ* and therefore remains active throughout the search. The resulting position ${\tilde{S}}_{i}\left(t+1\right)$ is subsequently processed by the adaptive foraging strategy.

#### 2.2.7. Foraging Strategy

Sooty shearwaters exhibit different foraging and diving patterns across marine habitats [32]. This observation provides the general motivation for using different perturbation intensities during the search. In SSMO, the fitness value is used as a computational indicator of solution quality, and the population median is used to divide individuals into two foraging states.

The foraging parameters, dimensional perturbation mask, and candidate position are defined as:

(15) $$\left(p_{i},c_{i}\right)=\left\{\begin{array}{cc} \left(0.35q,0.03q\right), & f\left(S_{i}\left(t\right)\right)>f_{\mathrm{median}}\left(t\right)\\ \left(0.12q,0.005q\right), & f\left(S_{i}\left(t\right)\right)\leq f_{\mathrm{median}}\left(t\right) \end{array}\right.$$

(16) $$m_{i,j}=\left\{\begin{array}{cc} 1, & r_{5,i,j}<p_{i},\\ 0, & r_{5,i,j}\geq p_{i}, \end{array}\right.r_{5,i,j}∼U\left(0,1\right)$$

(17) $$\hat{S_{i}}\left(t+1\right)=\tilde{S_{i}}\left(t+1\right)+c_{i}\left(\mathrm{ub}-\mathrm{lb}\right)⊙m_{i}⊙\xi_{i},\;\;\;\;\;\;\xi_{i}∼N\left(0,I_{D}\right)$$

where *q* denotes the foraging-parameter scale and is fixed at 0.80. The parameter *p_i_* determines the probability of perturbing each dimension, whereas *c_i_* controls the perturbation magnitude relative to the corresponding variable range. The vector *m_i_* = [*m_i_*_,1_, *m_i_*_,2_, …, *m_i_*_,*D*_] is a *D*-dimensional binary mask. For each individual and dimension, *r*_5,*i*,*j*_ is independently sampled from *U*(0, 1) at every iteration. The vector *ξ_i_* is independently generated from a *D*-dimensional standard normal distribution, and ⊙ denotes the Hadamard product. Therefore, only the dimensions selected by *m_i_* receive stochastic perturbations.

For a minimization problem, an individual with fitness worse than *f*_median_(*t*) is considered to be in an unfavorable region. With the adopted value of *q*, each dimension is perturbed with a probability of 0.28, and the perturbation scale is 2.4% of the corresponding variable range. The expected number of perturbed dimensions is therefore 0.28*D*. This setting encourages broader exploration in regions with relatively poor fitness.

An individual whose fitness is no worse than the median is considered to be in a promising region. Its perturbation probability is reduced to 0.096, and its perturbation scale is 0.4% of the variable range. It therefore perturbs approximately 0.096*D* dimensions. This smaller perturbation preserves most of the information in the current position while supporting local refinement.

Before boundary correction, the ratio between the expected coordinate-wise perturbation variances of the two foraging states is:

(18) $$\frac{(0.35q)\times {\left(0.03q\right)}^{2}}{(0.12q)\times {\left(0.005q\right)}^{2}}=105$$

The common scaling factor q is cancelled from this ratio. Therefore, it adjusts the absolute perturbation intensity without changing the relative difference between the two foraging states. Individuals in unfavorable regions receive substantially stronger perturbations, whereas promising individuals perform smaller local adjustments.

**Figure 5:**
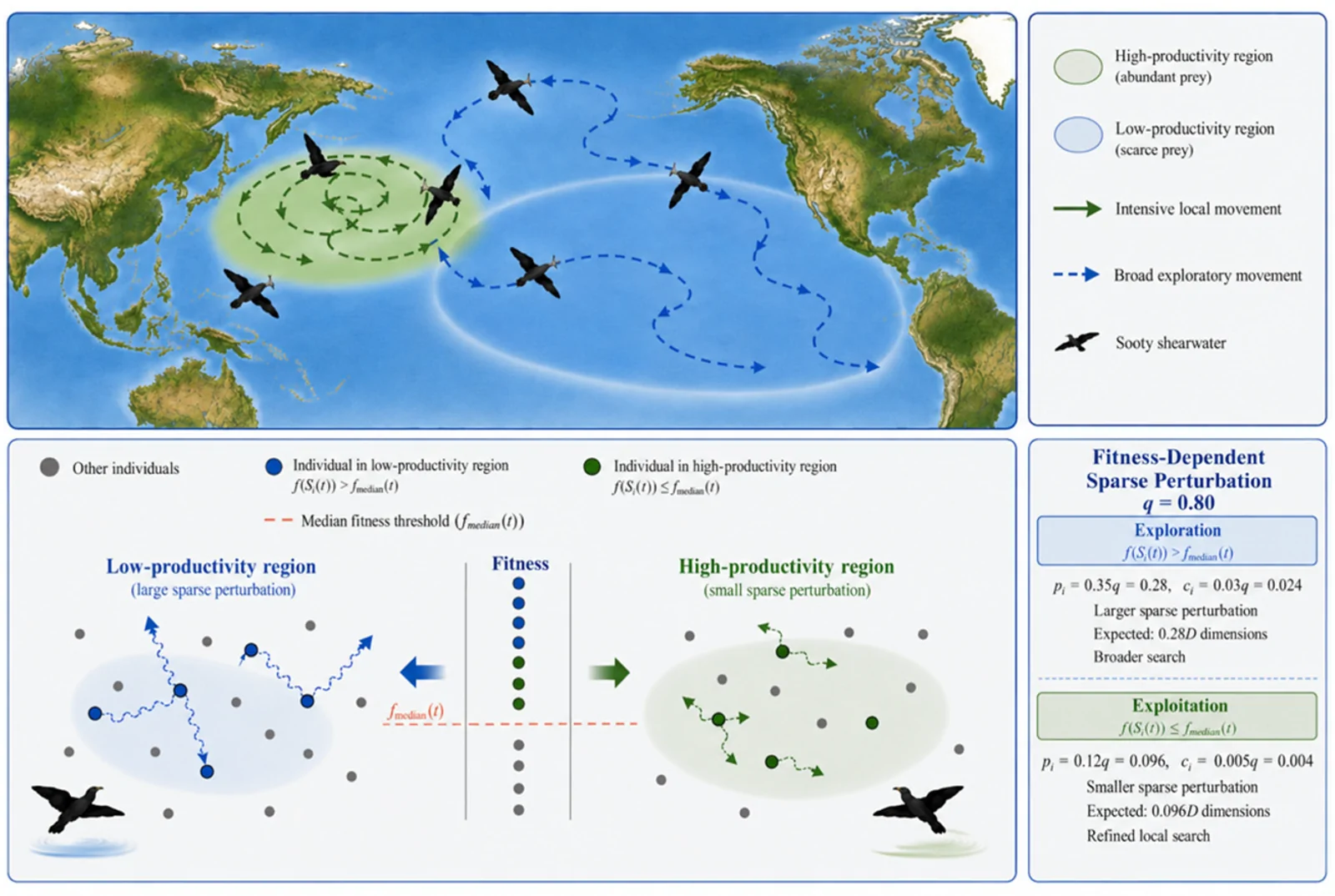
Schematic of the foraging strategy.

The dimensional mask reduces the likelihood of changing all variables simultaneously. This property helps preserve useful components of the migration position while allowing selected dimensions to explore new values. The two fitness-dependent foraging states and their different perturbation intensities are illustrated in Figure 5.

#### 2.2.8. Boundary Handling and Greedy Selection

The candidate position generated by the migration and foraging operations may contain components outside the feasible search space. Therefore, each decision variable is corrected independently using the following clipping rule:

(19) $$S_{i,j}\left(t+1\right)=\min\left[\max\left(\hat{S_{i,j}}\left(t+1\right),lb_{j}\right),ub_{j}\right],\;\;\;\;\;\;j=1,2,\ldots ,D$$

After boundary correction, the candidate position is evaluated once and compared with the current accepted position. For a minimization problem, the greedy selection rule is defined as

(20) $$S_{i}\left(t+1\right)=\left\{\begin{array}{cc} S_{i}\left(t+1\right), & \mathrm{if}f\left(S_{i}\left(t\right)\right)>f\left(S_{i}\left(t+1\right)\right)\\ S_{i}\left(t\right), & \mathrm{else} \end{array}\right.$$

The candidate position is retained if it has a better fitness value; otherwise, the current position is preserved.

**Figure 6:**
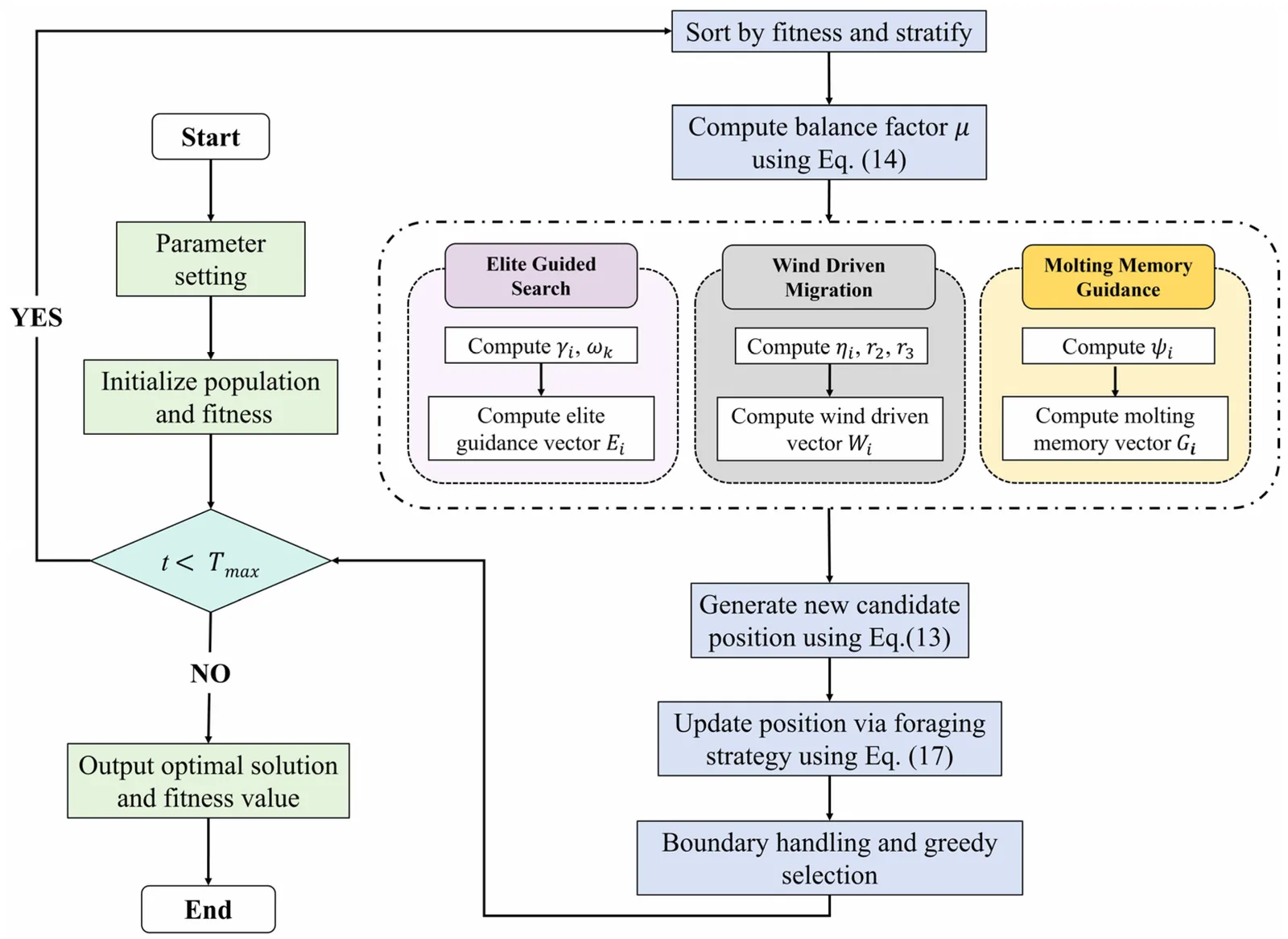
Flow chart of SSMO.

The complete procedure of SSMO, including population ranking, elite guidance, wind-driven migration, molting memory guidance, adaptive foraging, boundary handling, and greedy selection, is summarized in Algorithm 1 and Figure 6. The definitions and settings of the parameters and stochastic variables used in these operations are provided in Table 2. “Fixed” indicates that a quantity remains unchanged during an independent run, whereas “dynamic” indicates that it is updated deterministically or resampled during the iterative search.
**Algorithm 1** Pseudocode of the proposed SSMO**Input:** Search space bounds *lb* and *ub*, maximum number of iterations *T*_max_, problem dimension *D*, population size *N*, and objective function *f*. **Output:** The best candidate solution *S_best_* and its corresponding fitness value *f*(*S_best_*).1Initialize the population *S* uniformly within *lb*, *ub* using Equation (2).2Evaluate the fitness value of each individual.3Determine the initial best solution S*_best_* and its fitness value *f*(S*_best_*).4Set *t* = 15**While** *t* ≤ *T_max_* **do**6
Sort all individuals in ascending order of fitness.7
Divide the sorted population into elite group, main group, and weak group according to fitness ranking.8
**For** *i* = 1 to *N*
**do**9

Compute the stochastic activity factor *γ_i_* and elite weights *ω_k_*.10

Calculate the elite guidance vector *E_i_* using Equation (5).11

Update the wind intensity coefficient *η_i_* and randomly select the reference indices *r*_2_ and *r*_3_.12

Calculate the wind-driven vector *W_i_* using Equation (8).13

Compute the molting intensity factor *ψ_i_*.14

Calculate the molting memory vector *G_i_* using Equation (11).15

Compute the balance factor *μ* using Equation (14).16

Generate a candidate position according to Equation (13):17

$\tilde{S_{i}}\left(t+1\right)=S_{i}\left(t\right)+\mu E_{i}+\left(1-\mu \right)G_{i}+W_{i}$18

Carry out foraging strategy and update the position according to Equation (17)19

Apply boundary control to ensure that the candidate position remains within *lb*, *ub*.20

Evaluate the fitness value *f*(*S_i_*(*t* + 1)).21

**if** *f*(*S_i_*(*t* + 1)) < *f*(*S_i_*(*t*)) **then**22


Accept the candidate position: *S_i_*(*t* + 1).23

**else**24


Retain the current position: *S_i_*(*t* + 1) ← *S_i_*(*t*).25

**end if**26
**End for**27
Update the global best solution *S_best_* and its fitness value *f*(S*_best_*).28
 Set *t* = *t* + 1.29 
**End While**
30Return S*_best_* and *f*(*S_best_*).

#### 2.2.9. Relationship to Established Search Strategies

Several components of SSMO are related to established population-based optimization principles. Differential information, elite guidance, best-so-far information, and stochastic perturbation have been used in different forms by existing algorithms. The methodological contribution of SSMO lies in adapting established optimization principles to formulate its four search mechanisms and coordinating them within a unified position-update procedure. This design aims to balance exploration and exploitation and improve search efficiency. Table 3 summarizes the main relationships and differences between SSMO and the corresponding established strategies.

The overall update in Equation (13) distinguishes SSMO from applying these strategies independently. Balance factor *μ* gradually shifts the main guidance source from the rank-weighted multi-elite vector to the global best-so-far direction, while the rank-biased wind-driven vector remains active throughout the search. The resulting migration position is subsequently processed by the median-based sparse perturbation in Equation (17) and greedy selection in Equation (20). Thus, the distinctive mathematical structure of SSMO is the coordinated use of multi-elite directional aggregation, rank-biased differential displacement, nonlinear complementary guidance, and fitness-dependent dimensional perturbation.

### 2.3. Complexity Analysis

The time complexity of SSMO is derived by decomposing the algorithmic procedure into initialization, population ranking, position updating, and fitness evaluation. Let *N*, *D*, *T_max_*, *L*, and *F* represent the population size, search dimension, maximum iteration number, number of elite individuals, and computational cost required for a single objective-function evaluation, respectively.

The initialization procedure generates *N* candidate solutions in a *D*-dimensional search space, resulting in a computational complexity of *O*(*ND*). Evaluating the initial population requires *O*(*NF*), while selecting the initial global best solution requires *O*(*N*). Therefore, the total complexity of the initialization stage is *O*(*ND* + *NF*).

At the beginning of each iteration, the population is ranked according to fitness, requiring *O*(*N*log*N*). The elite and main groups are then formed from the ranked population. This assignment requires *O*(*N*) and is dominated by the sorting operation.

The iterative search consists of elite-guided movement, wind-driven migration, molting memory guidance, position updating, adaptive foraging, boundary handling, fitness evaluation, and greedy selection. Among these operations, elite guidance introduces the main additional population-level interaction. For each individual, the search direction is calculated using *L* elite individuals over *D* dimensions. Its computational complexity for the whole population is therefore *O*(*NLD*). The remaining vector-based operations are applied to all individuals over all dimensions and require *O*(*ND*). Greedy selection and global-best updating require *O*(*N*), while evaluating the (N) candidate solutions requires *O*(*NF*). Accordingly, the computational complexity of one iteration is *O*(*N*log*N* + *NLD* + *ND* + *NF*).

Combining the initialization and iterative stages, the total time complexity of SSMO is *O*(*ND* + *NF* + *T*_max_(*N*log*N* + *NLD* + *ND* + *NF*)).

Since the initialization cost occurs only once, the asymptotic time complexity is expressed as *O*(*T*_max_(*N*log*N* + *NLD* + *ND* + *NF*))

In SSMO, the number of elite individuals is determined by *L* = max(2, round(*ρN*)), where the elite proportion is fixed at *ρ* = 0.15. Consequently, the elite-guidance term *O*(*NLD*) corresponds asymptotically to *O*(*N*^2^*D*). SSMO therefore contains an additional population-level interaction term compared with standard DE, PSO, and GWO, whose basic position-update operations are generally *O*(*ND*) per iteration, excluding objective-function evaluations. In the present experiments, *N* = 30 and *L* = 5, which limits the size of the elite group. Nevertheless, population ranking and elite-guidance calculations introduce additional computational overhead. The memory complexity of SSMO is *O*(*ND*), mainly owing to the storage of the current population, candidate solutions, and their fitness values.

To quantify this overhead, the runtime of SSMO is compared with those of differential evolution (DE), particle swarm optimization (PSO), and the grey wolf optimizer (GWO). The comparison is conducted in F1, F10, F20, and F30 of the CEC2017 benchmark suite at *D* = 30. For all algorithms, the population size is fixed at 30, and the maximum number of objective-function evaluations is set to 300,000 for each independent run. Initialization evaluations are included in this budget, and each algorithm is independently executed 30 times. All algorithms are executed under the same software and hardware conditions. The recorded runtime includes the optimization process and objective-function evaluations but excludes result storage. Table 4 reports the mean and standard deviation of the runtime obtained from 30 independent runs.

As shown in Table 4, PSO has the lowest average runtime, whereas DE requires the longest runtime. SSMO records an average runtime of 1.7130 s, which is 23.2% lower than that of DE and 0.6% lower than that of GWO, but 50.2% higher than that of PSO. SSMO is slightly faster than GWO in F1, F10, and F20, while GWO is marginally faster in F30. The higher runtime of SSMO relative to PSO is consistent with the additional population ranking and elite-guidance operations identified in the complexity analysis. However, under the adopted population size and function-evaluation budget, the runtime of SSMO remains close to that of GWO and lower than that of DE. The runtime comparison complements the theoretical analysis by showing the computational overhead of SSMO under the adopted experimental settings.

## 3. Numerical Results and Analysis of Benchmark Test Suites

This section evaluates the optimization capability of SSMO in two widely used benchmark suites, namely CEC2017 under 30D, 50D, and 100D settings and CEC2022 under 10D and 20D settings [33,34]. A group of representative and competitive optimizers is selected for comparison, and the performance of SSMO is assessed using both visual convergence behavior and quantitative statistical indicators.

### 3.1. Experimental Setup

The comparison algorithms are selected to cover established reference methods and different search mechanisms relevant to SSMO. The main comparison includes 11 algorithms: PSO, GWO, WOA, DBO, the Great Wall Construction Algorithm (GWCA) [35], the Snow Geese Algorithm (SGA) [36], the Ivy Algorithm (IVY) [37], the Fata Morgana Algorithm (FATA) [38], the Polar Lights Optimizer (PLO) [39], the Superb Fairy-wren Optimization Algorithm (SFOA) [40], and the Electromagnetic Wave Propagation Algorithm (EMWPA) [41]. PSO provides a reference for individual-best and global-best guidance, GWO for guidance by multiple leading solutions, and WOA for exploration–exploitation switching. These methods allow the population-guidance mechanisms of SSMO to be assessed against established approaches. The remaining algorithms extend the comparison to recent methods with different population-update strategies. Among them, DBO provides a reference for role-specific search behaviors, while SGA is relevant to the migration-inspired framework of SSMO. To further assess SSMO against adaptive differential search, four DE variants-SaDE, L-SHADE-EpSin, L-SHADE-SPACMA, and RSDE-are examined separately in Section 3.5.

All algorithms are evaluated under identical conditions, with a population size of 30. The evaluation budgets follow the official settings of each benchmark suite. For CEC2017, the maximum number of function evaluations is set to 10,000 × *D*. For CEC2022, the maximum number is set to 200,000 for 10D and 1,000,000 for 20D. Each algorithm is independently executed 30 times. The dimensional settings are selected to assess performance as the search dimension increases and to provide complementary evaluations using two benchmark suites. For CEC2017, 30D serves as the baseline setting, while 50D and 100D extend the evaluation to progressively larger search spaces. This allows changes in solution quality, run-to-run variability, and relative algorithm performance to be examined with increasing dimensionality. For CEC2022, the 10D and 20D competition settings are adopted to assess performance in a second suite with a different collection of benchmark functions. Together, these experiments assess the dimensional robustness of SSMO up to 100D in CEC2017 and its performance across the two tested benchmark suites. The parameters of the compared algorithms follow their original studies, as listed in Table 5.

### 3.2. Convergence Performance Analysis

**Figure 7:**
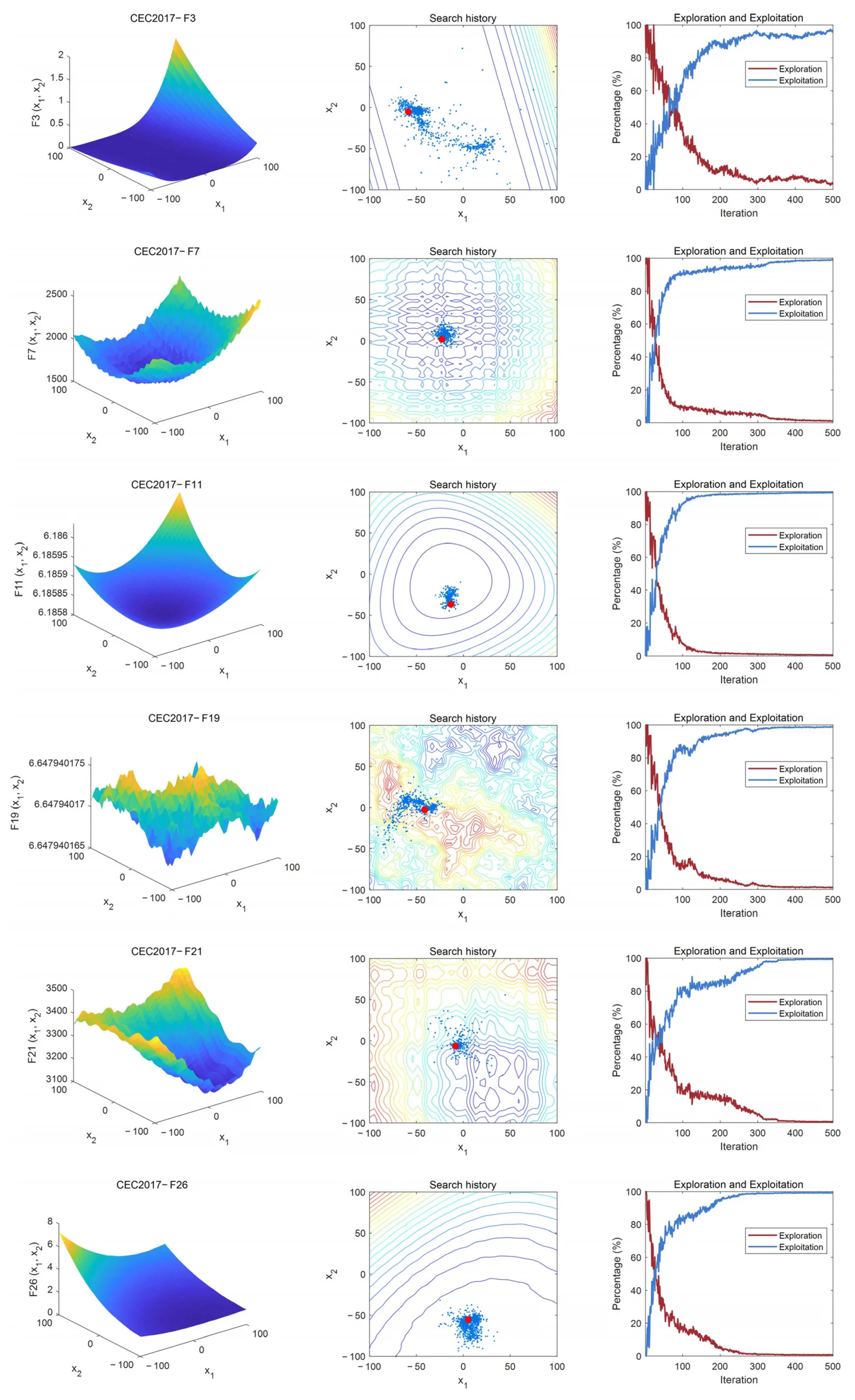
Convergence behaviors of SSMO in the search process. Blue dots represent sampled search positions, and red dots indicate the best solutions found.

Figure 7 provides a multi-perspective visualization of the convergence behavior of SSMO. It includes three-dimensional landscapes of six representative CEC2017 functions, historical search-trajectory scatter plots, and curves describing the variation of exploration and exploitation ratios. These results show that the population progressively moves toward promising regions, suggesting that SSMO can coordinate global exploration and local exploitation during the search process.

### 3.3. Results and Analysis of CEC2017

The CEC2017 experiments are conducted at 30D, 50D, and 100D. Table A1, Table A2 and Table A3 in Appendix A summarize the results obtained by each algorithm over 30 independent runs. Mean denotes the arithmetic mean of the final objective values, Std denotes their standard deviation, and Best denotes the minimum value among the 30 runs. These metrics describe the average solution quality, run-to-run variability, and the best observed outcome, respectively. The lowest numerical values for each function and metric are highlighted in bold. SSMO obtains favorable mean objective values and best-run results in many test functions. Relatively small standard deviations in several cases also indicate limited run-to-run variability of those functions.

**Figure 8:**
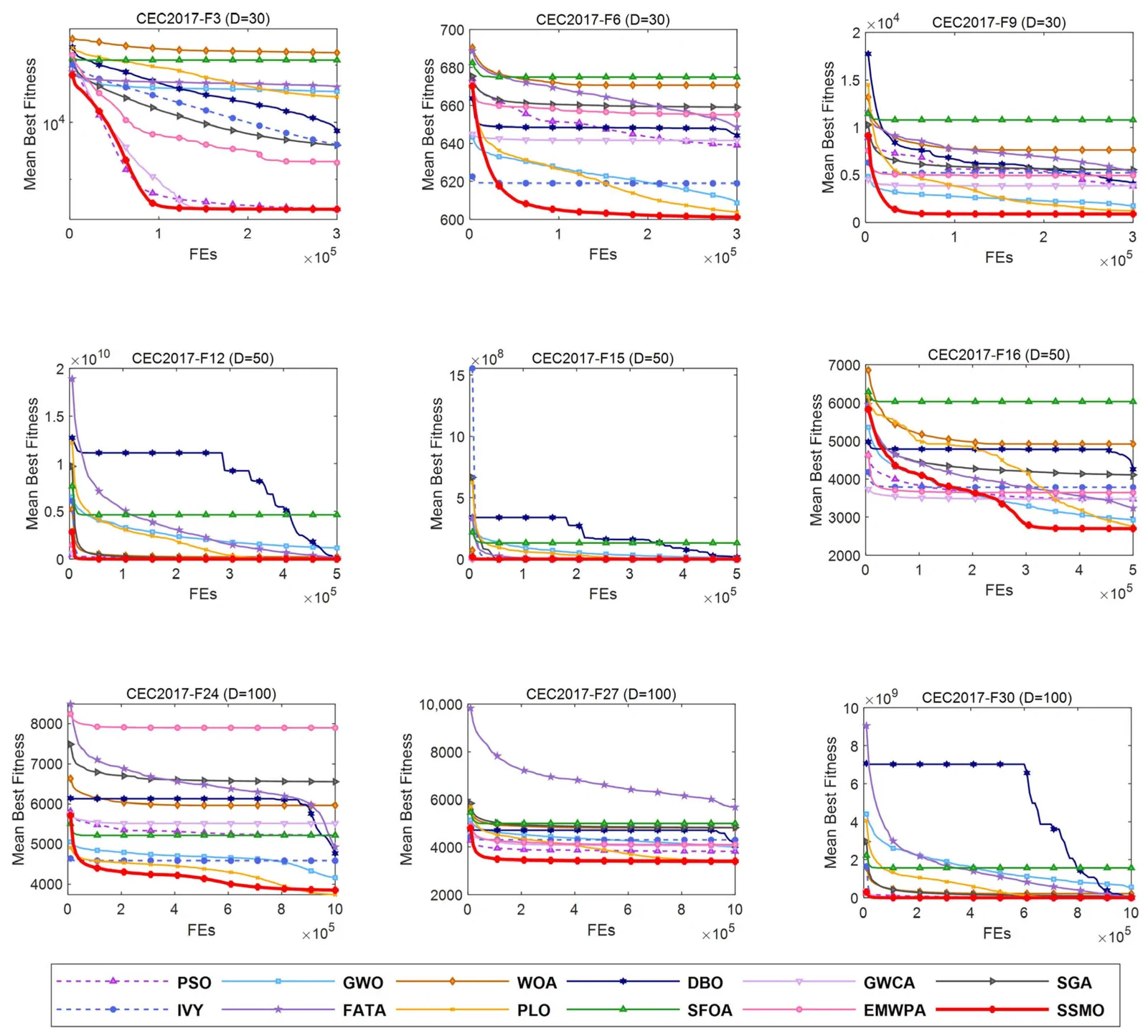
Comparison curves of different algorithms in CEC 2017 test suite.

**Figure 9:**
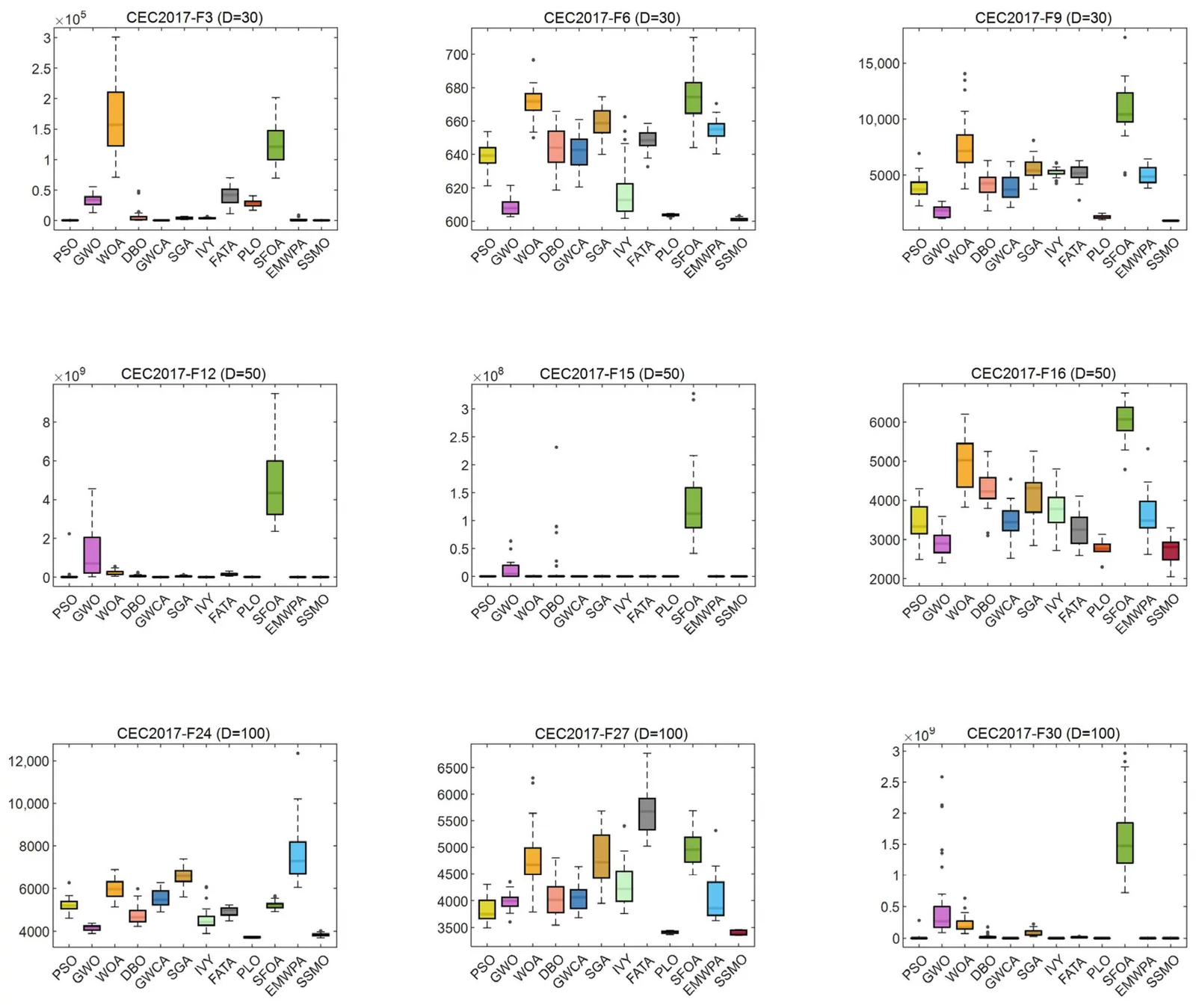
Comparison of box plots of different algorithms in CEC 2017 test suite.

**Figure A1:**
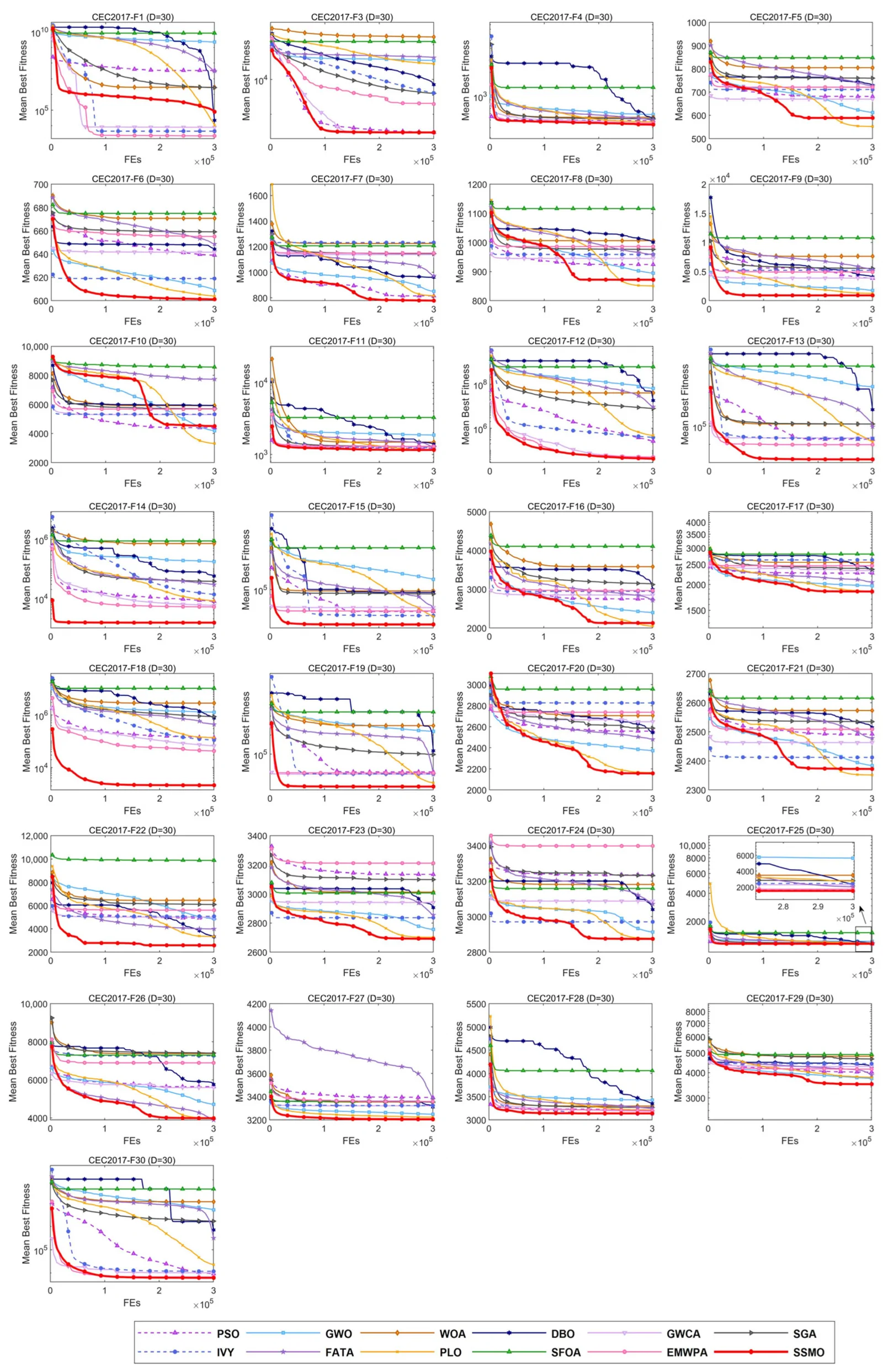
The comparison curves in the CEC 2017 test suite (Dim = 30).

**Figure A2:**
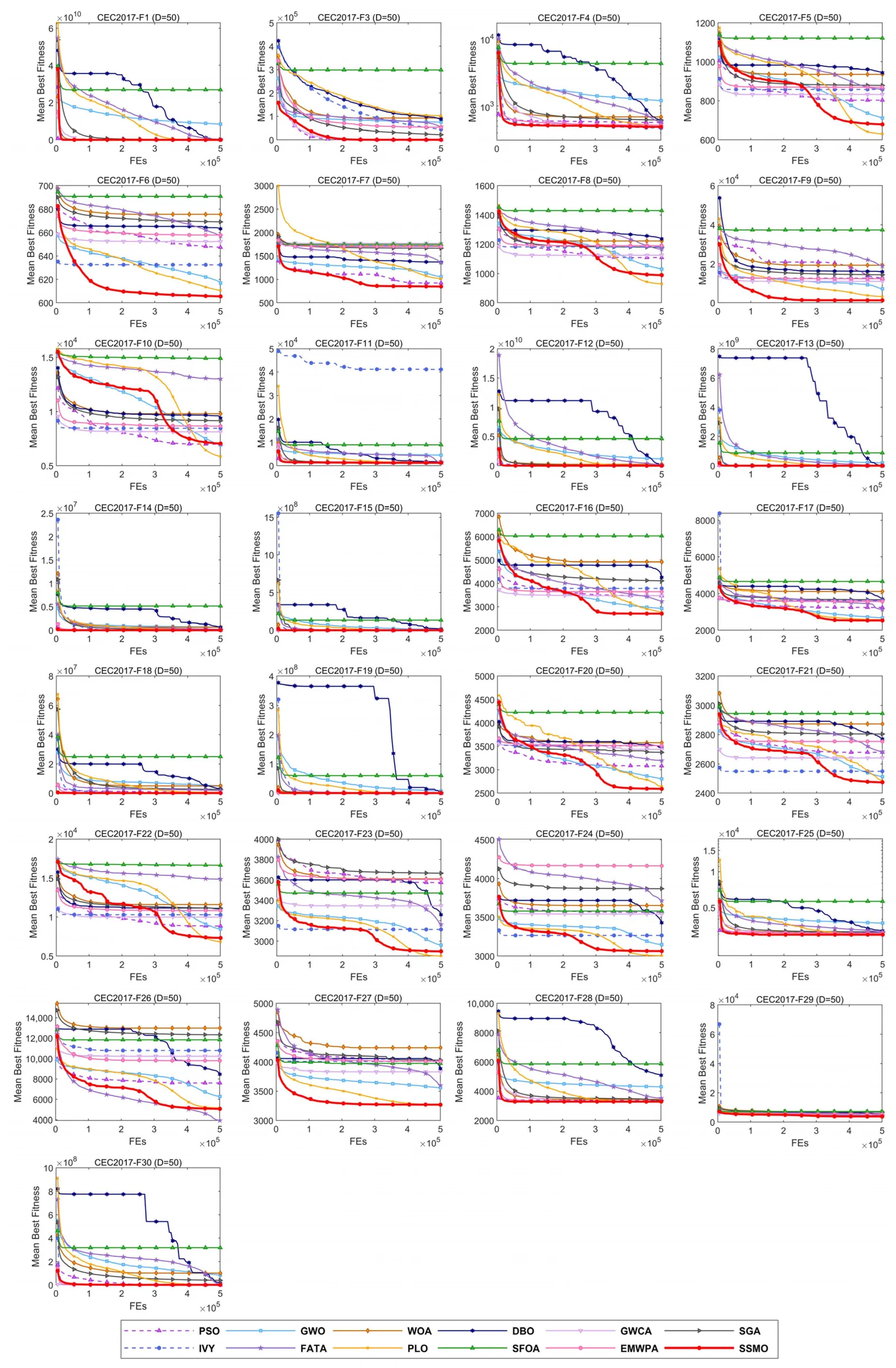
The comparison curves in the CEC 2017 test suite (Dim = 50).

**Figure A3:**
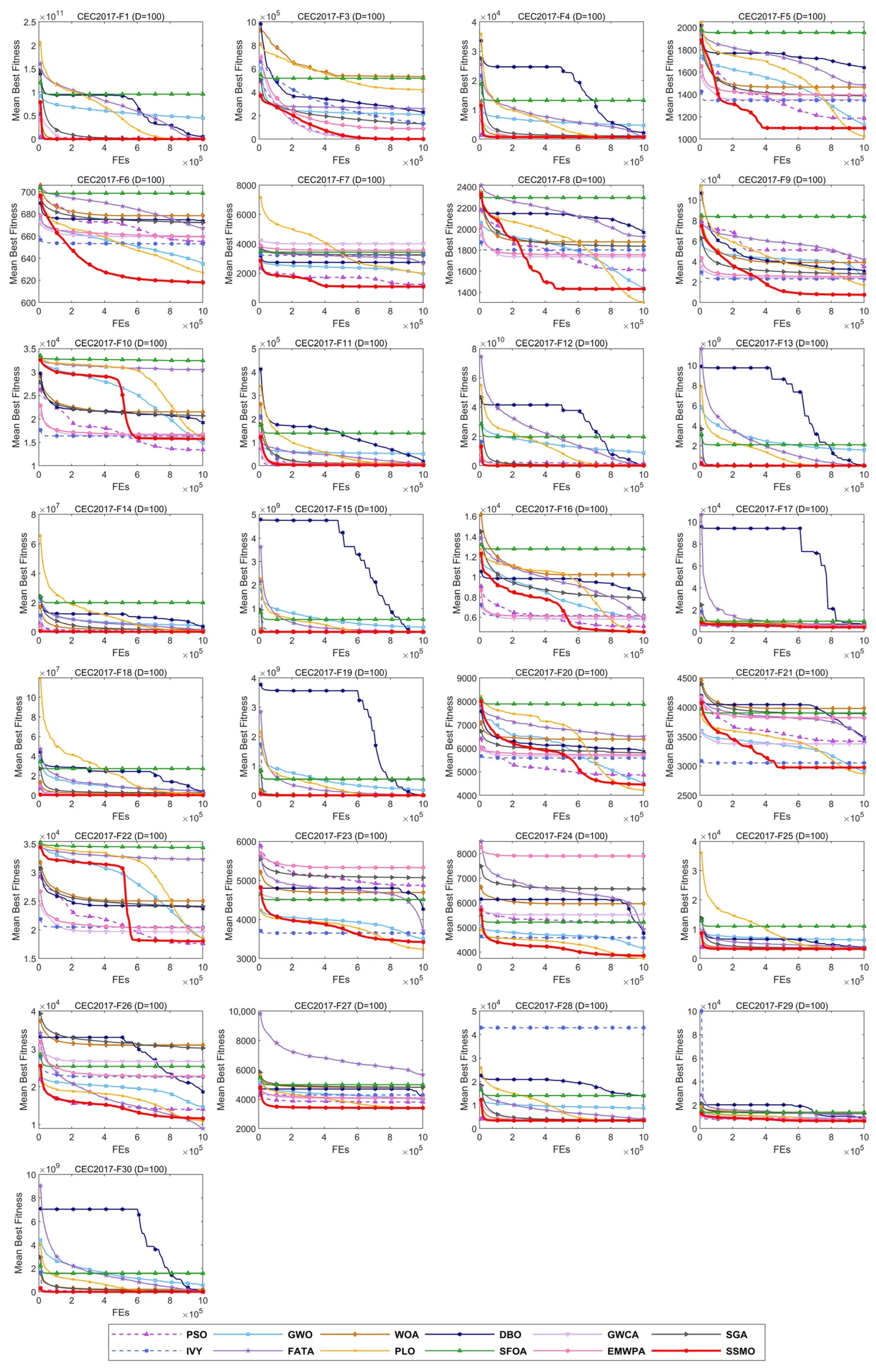
The comparison curves in the CEC 2017 test suite (Dim = 100).

**Figure A4:**
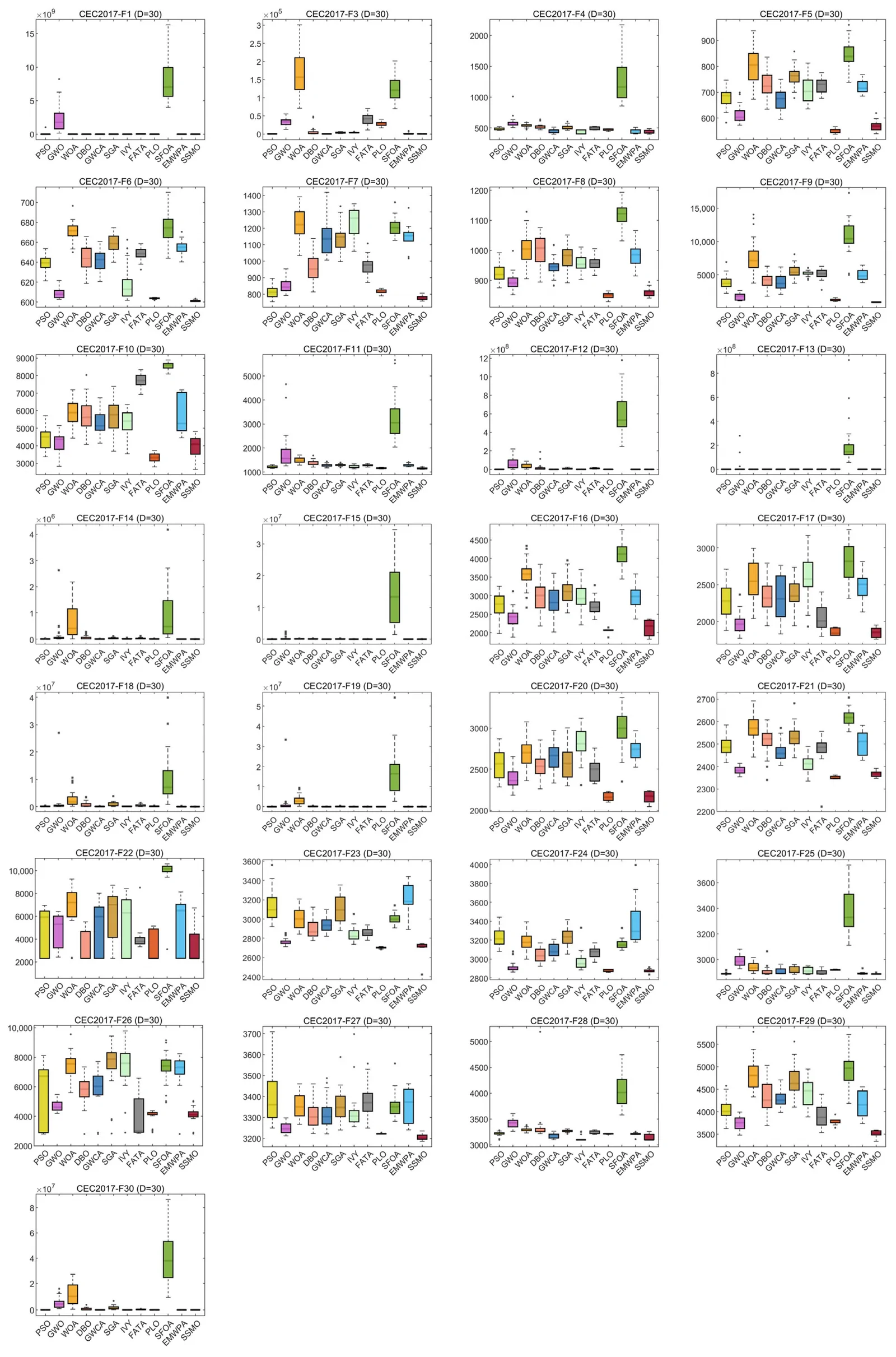
Box plot comparison of algorithms in the CEC 2017 test suite (Dim = 30).

**Figure A5:**
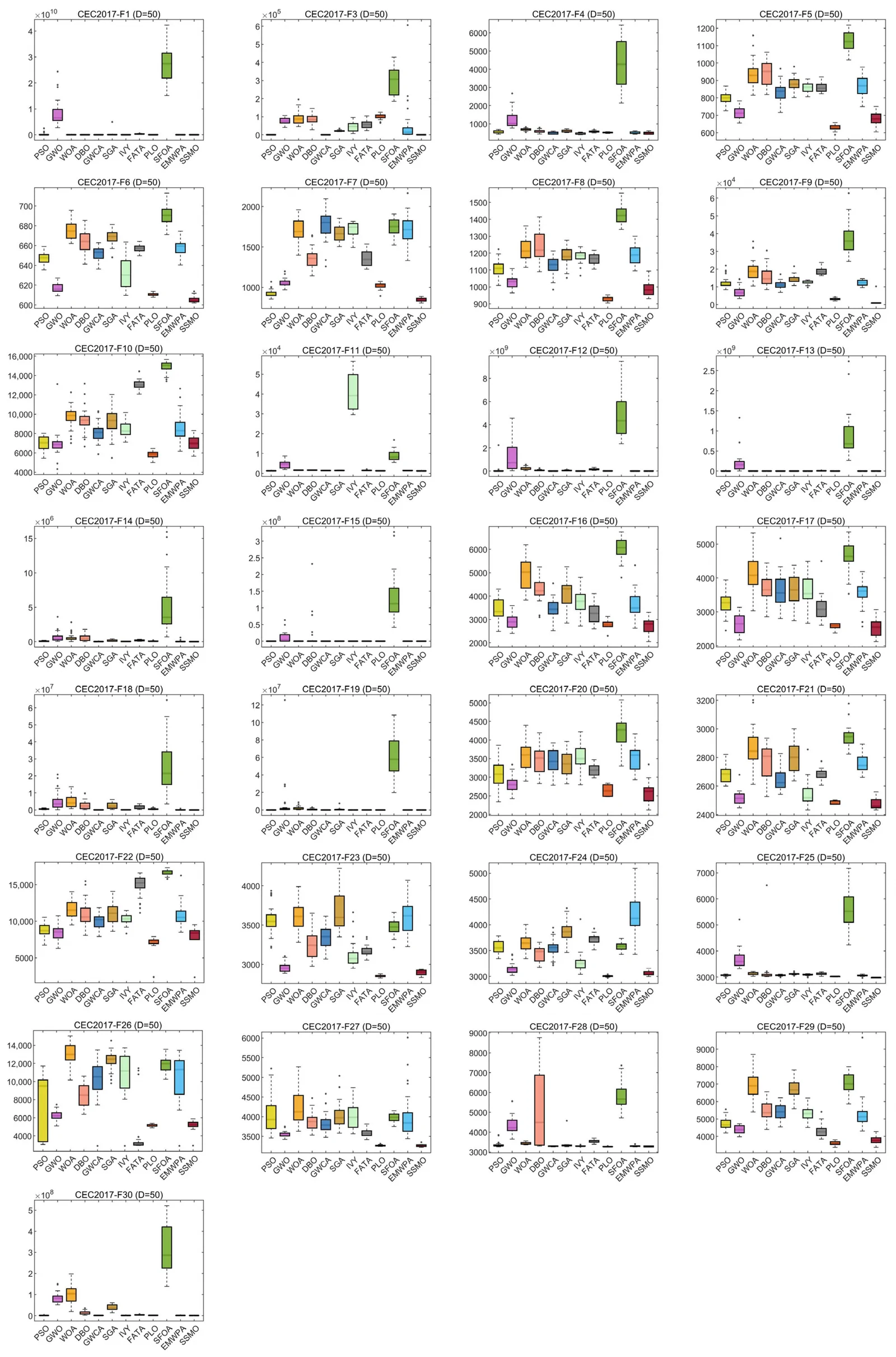
Box plot comparison of algorithms in the CEC 2017 test suite (Dim = 50).

**Figure A6:**
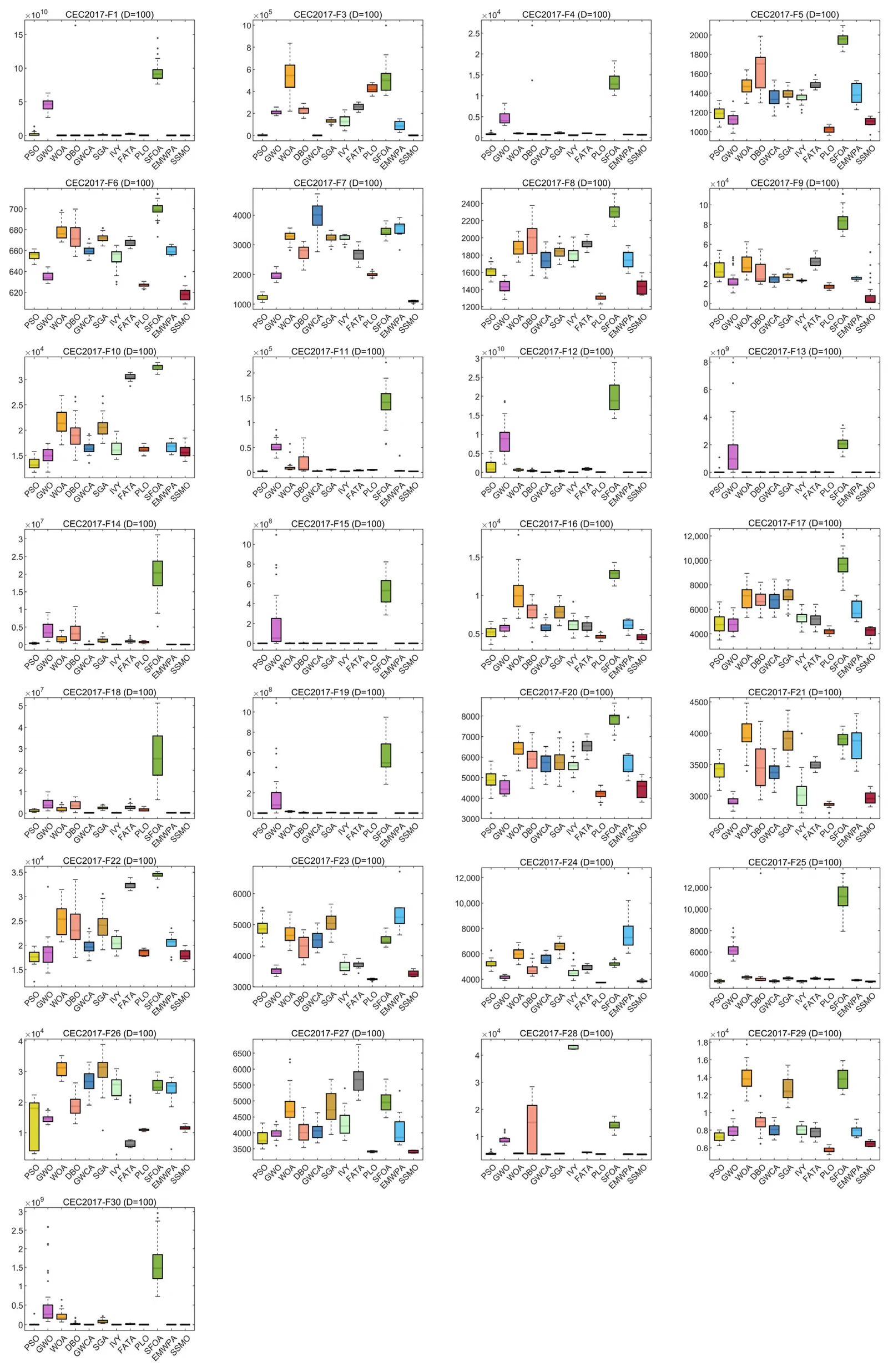
Box plot comparison of algorithms in the CEC 2017 test suite (Dim = 100).

Figure 8 and Figure 9 present the convergence curves and box plot distributions of selected CEC2017 functions under different dimensions. According to the convergence curves, SSMO generally shows fast convergence during the early stage of the search. It then continues to improve the solutions in later iterations, which indicates its ability to maintain effective search progress and approach promising regions. The box plots show that SSMO usually obtains lower objective values and more compact result distributions of the selected functions, indicating lower run-to-run variability of the selected functions. The complete convergence curves and box plots for all benchmark functions and dimensional settings are presented in Appendix B, Figure A1, Figure A2, Figure A3, Figure A4, Figure A5 and Figure A6.

The Wilcoxon rank-sum test is a nonparametric method for assessing differences between two independent samples without requiring normally distributed observations [42]. In this study, two-sided tests compare the final objective values obtained from independent runs of SSMO and each competing algorithm for each CEC2017 function and dimensional setting. To account for multiple comparisons, Holm’s correction [43] is applied to the 11 comparisons involving SSMO within each function and dimensional setting, with a family-wise significance level of 0.05. For significant comparisons, the direction is determined from the average ranks of the two groups in the pooled sample, with lower ranks corresponding to lower objective values. The symbols “+”, “−”, and “=“ denote a significant advantage for SSMO, a significant advantage for the competing algorithm, and no statistically significant difference, respectively.

Table 6 summarizes these outcomes across the 29 functions as win/tie/loss counts, corresponding to “+/=/−”. The aggregate counts for 30D, 50D, and 100D are 288/18/13, 277/26/16, and 251/52/16, respectively. Although ties and losses occur against some competitors, wins remain dominant in all three-dimensional settings. As the dimension increases, the number of wins decreases and the number of ties increases, suggesting that SSMO’s relative advantage becomes less pronounced in some functions. Nevertheless, the comparison outcomes generally favor SSMO across the tested CEC2017 dimensions under the adopted evaluation budgets.

The Friedman ranking analysis [44] summarizes the relative performance of the 12 algorithms across the CEC2017 functions. For each function and dimensional setting, the algorithms are ranked according to their mean final objective values over independent runs, with rank 1 assigned to the lowest mean value. Equal values receive the average of the ranks they occupy, and the calculations use unrounded numerical results. The overall average rank of each algorithm is obtained by averaging its function-wise ranks across the 29 functions, with equal weight assigned to each function. This procedure is performed separately for 30D, 50D, and 100D.

**Figure 10:**
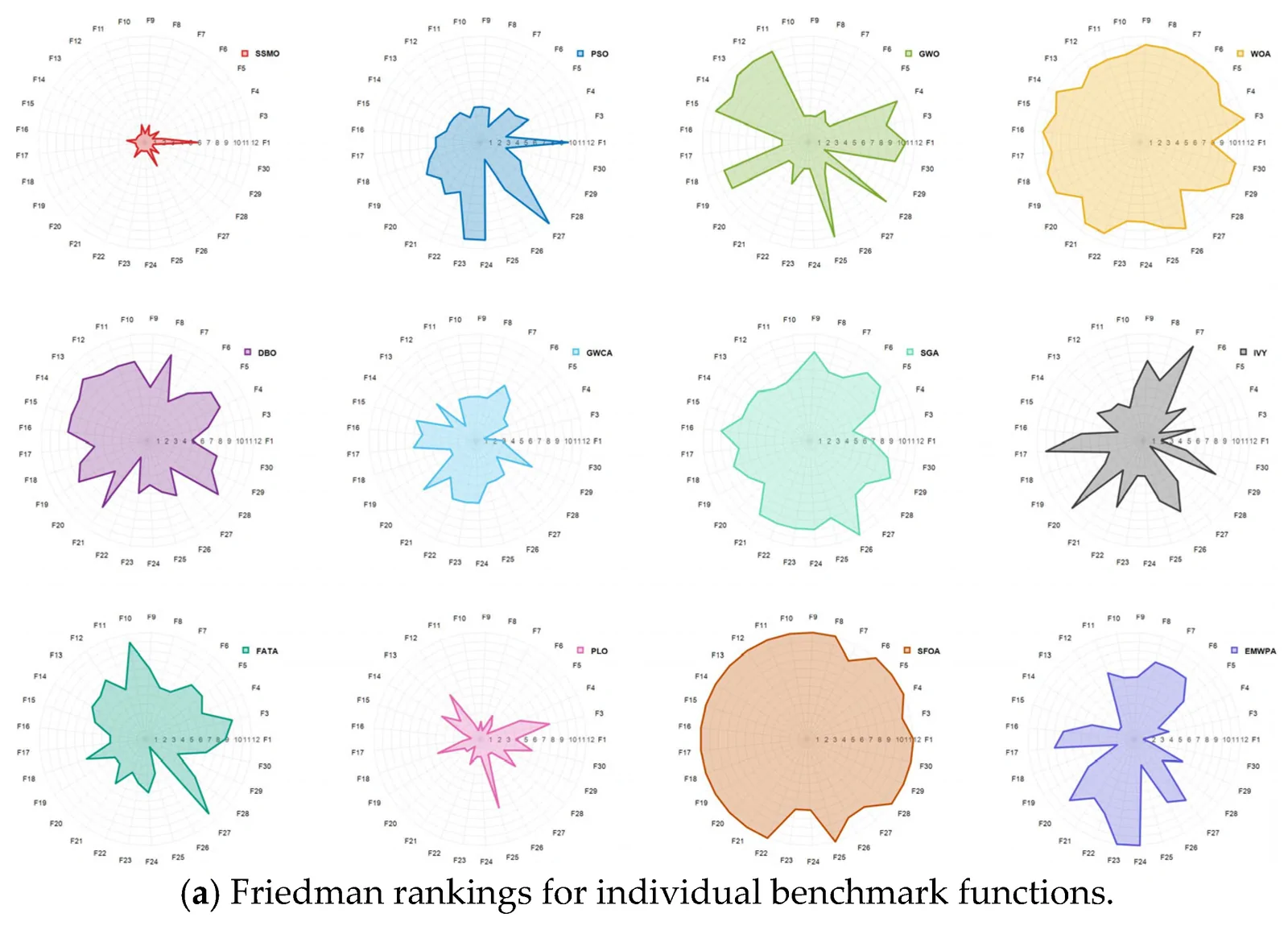

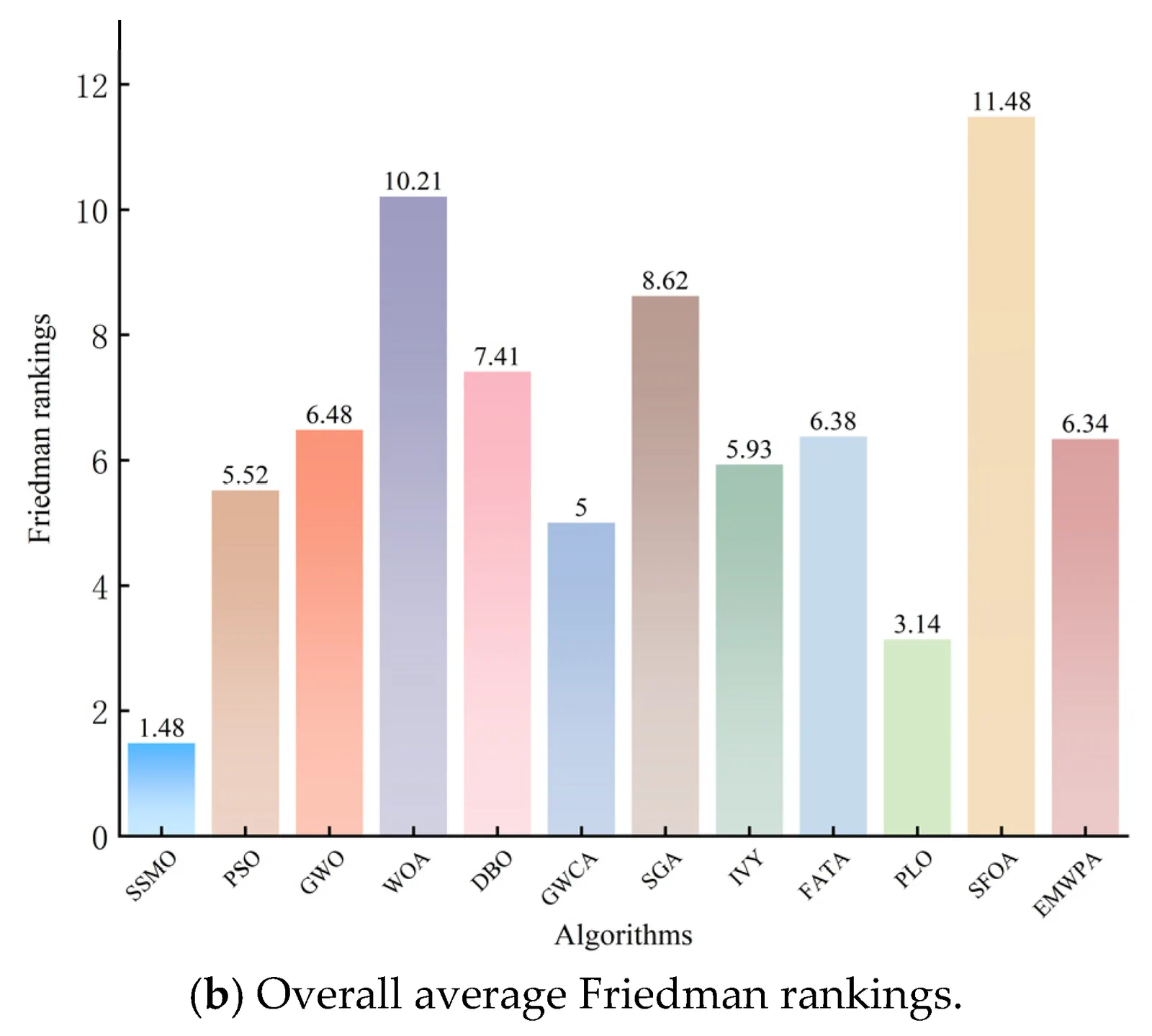
Friedman ranking results for the 30-dimensional CEC 2017 benchmark functions.

**Figure 11:**
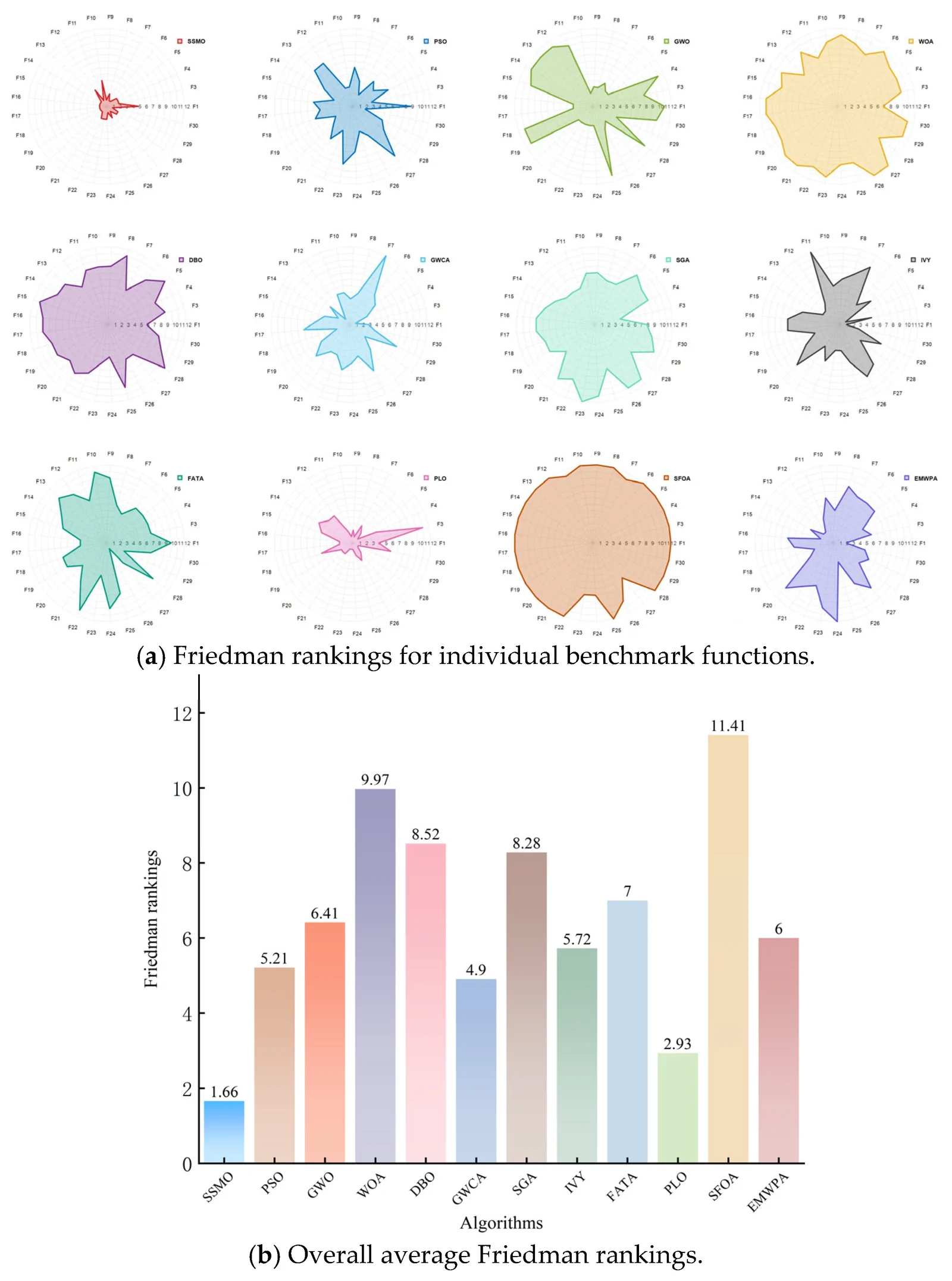
Friedman ranking results for the 50-dimensional CEC 2017 benchmark functions.

**Figure 12:**
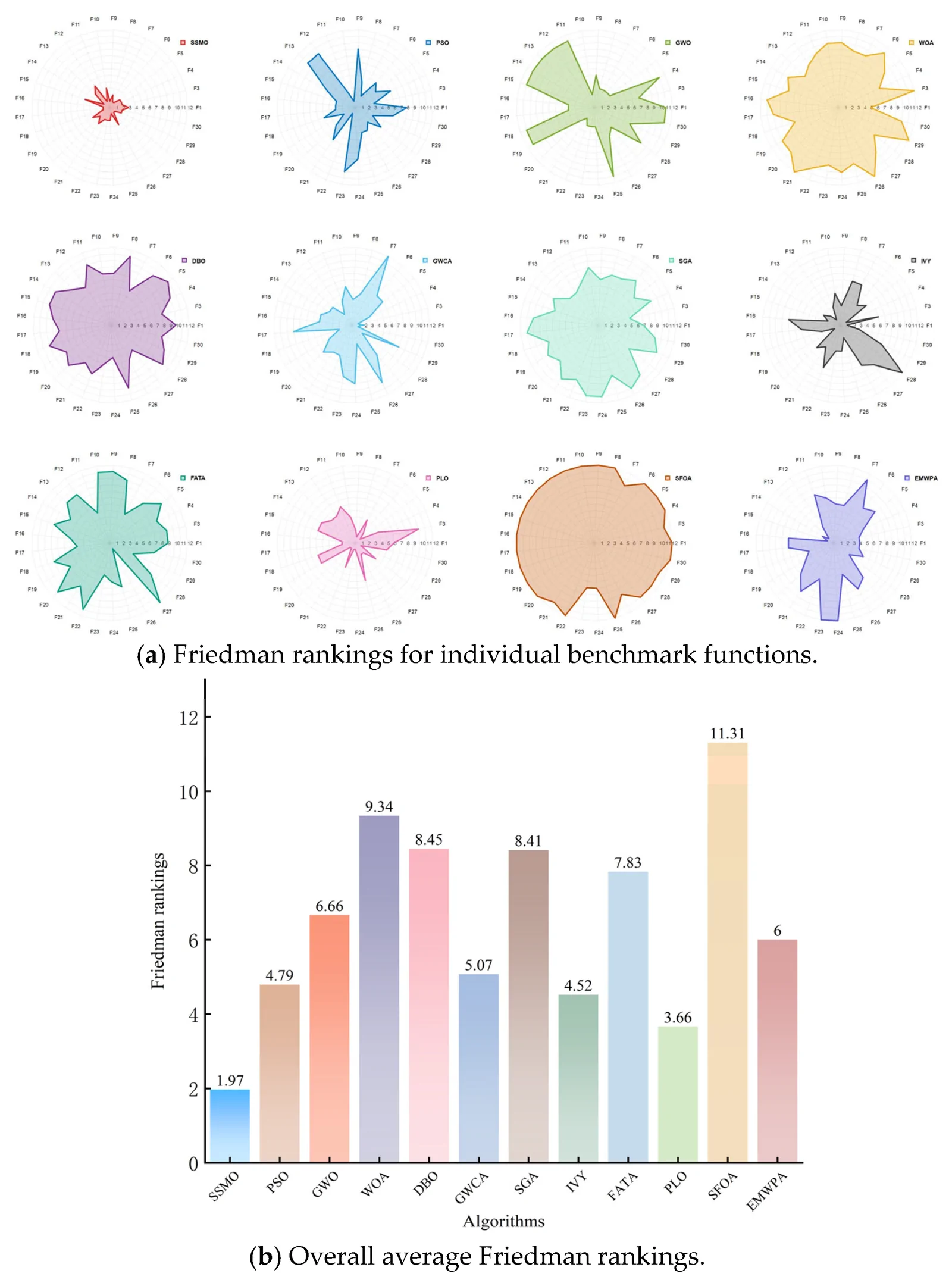
Friedman ranking results for the 100-dimensional CEC 2017 benchmark functions.

Figure 10, Figure 11 and Figure 12 present the function-wise ranks in radar charts and the overall average ranks in bar charts. SSMO generally occupies lower-ranked regions in the radar charts and obtains the lowest average ranks of 1.48, 1.66, and 1.97 at 30D, 50D, and 100D, respectively. Although its average rank increases with dimension, SSMO retains the lowest average rank in each setting. These results indicate favorable relative performance across the evaluated CEC2017 dimensions under the adopted function-evaluation budgets.

### 3.4. Results and Analysis of CEC2022

The CEC2022 benchmark suite is further used to evaluate SSMO and the competing algorithms under 10D and 20D settings. Table A4 and Table A5 in Appendix A present the mean, standard deviation, and best values obtained for the CEC2022 functions under the two-dimensional settings. SSMO obtains the lowest mean values in more than half of the 10D functions and remains competitive in most 20D functions, indicating effective search capability under the tested CEC2022 settings.

**Figure 13:**
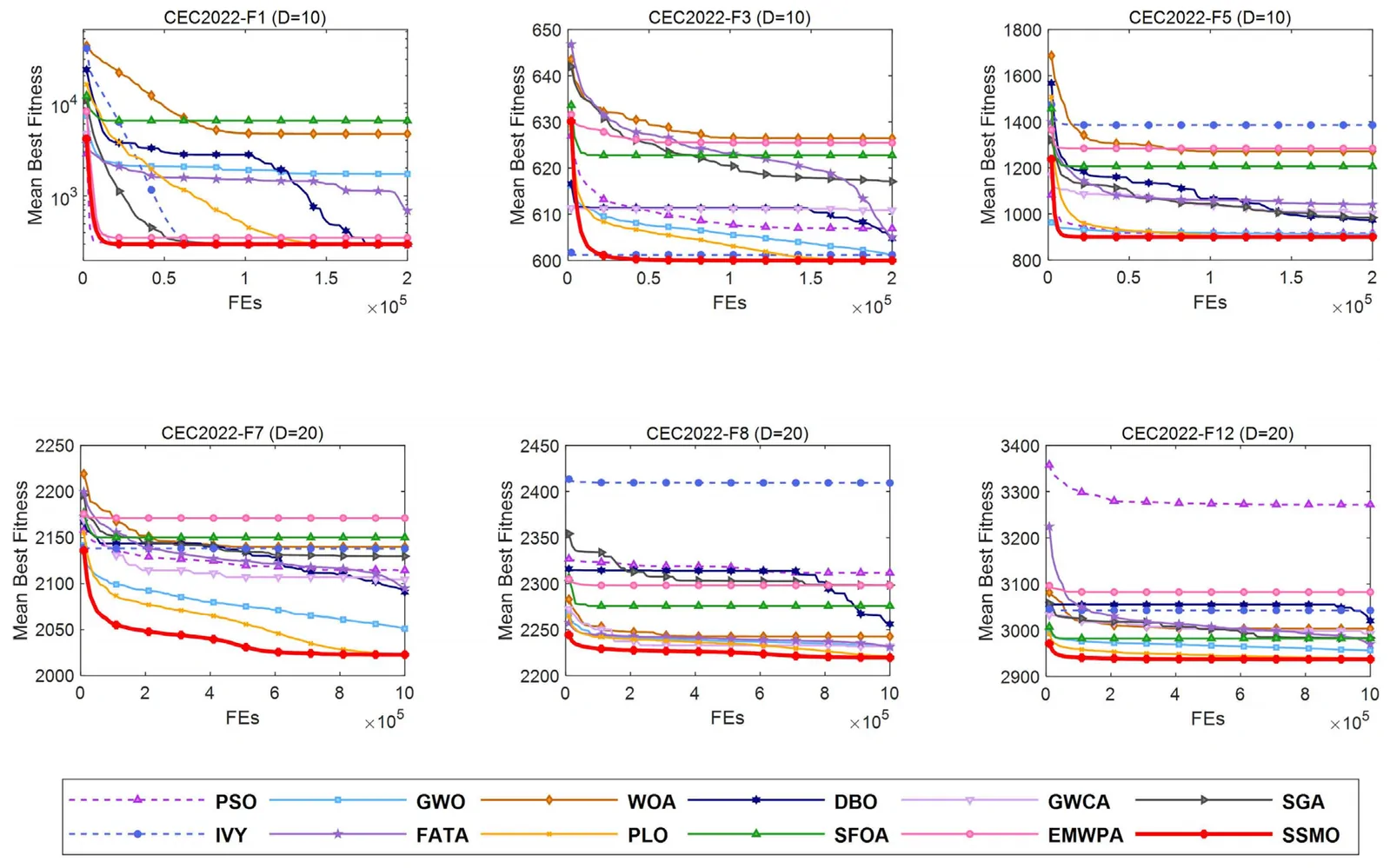
Comparison curves of different algorithms in CEC 2022 test suite.

**Figure 14:**
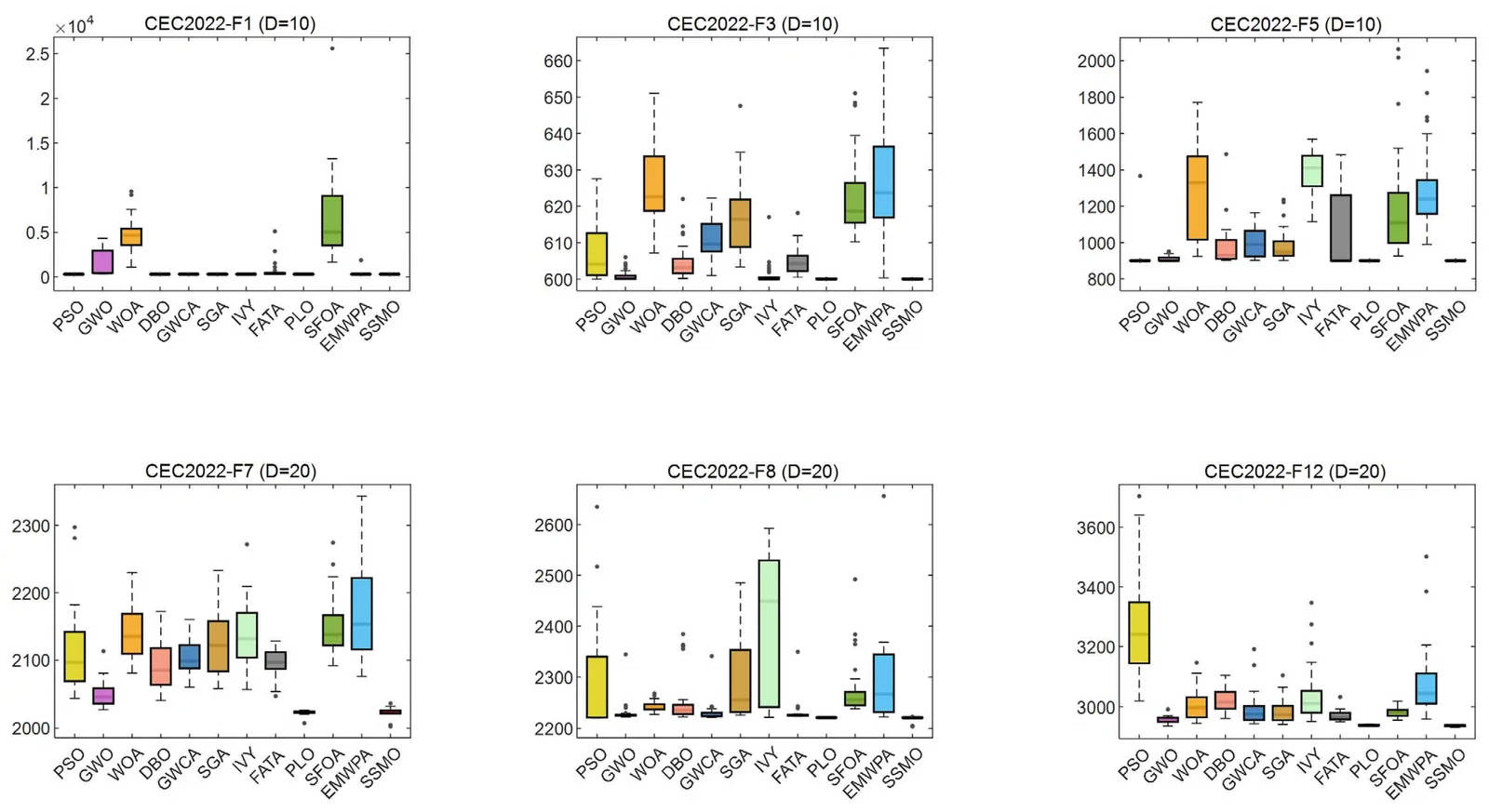
Comparison of box plots of different algorithms in CEC 2022 test suite.

**Figure A7:**
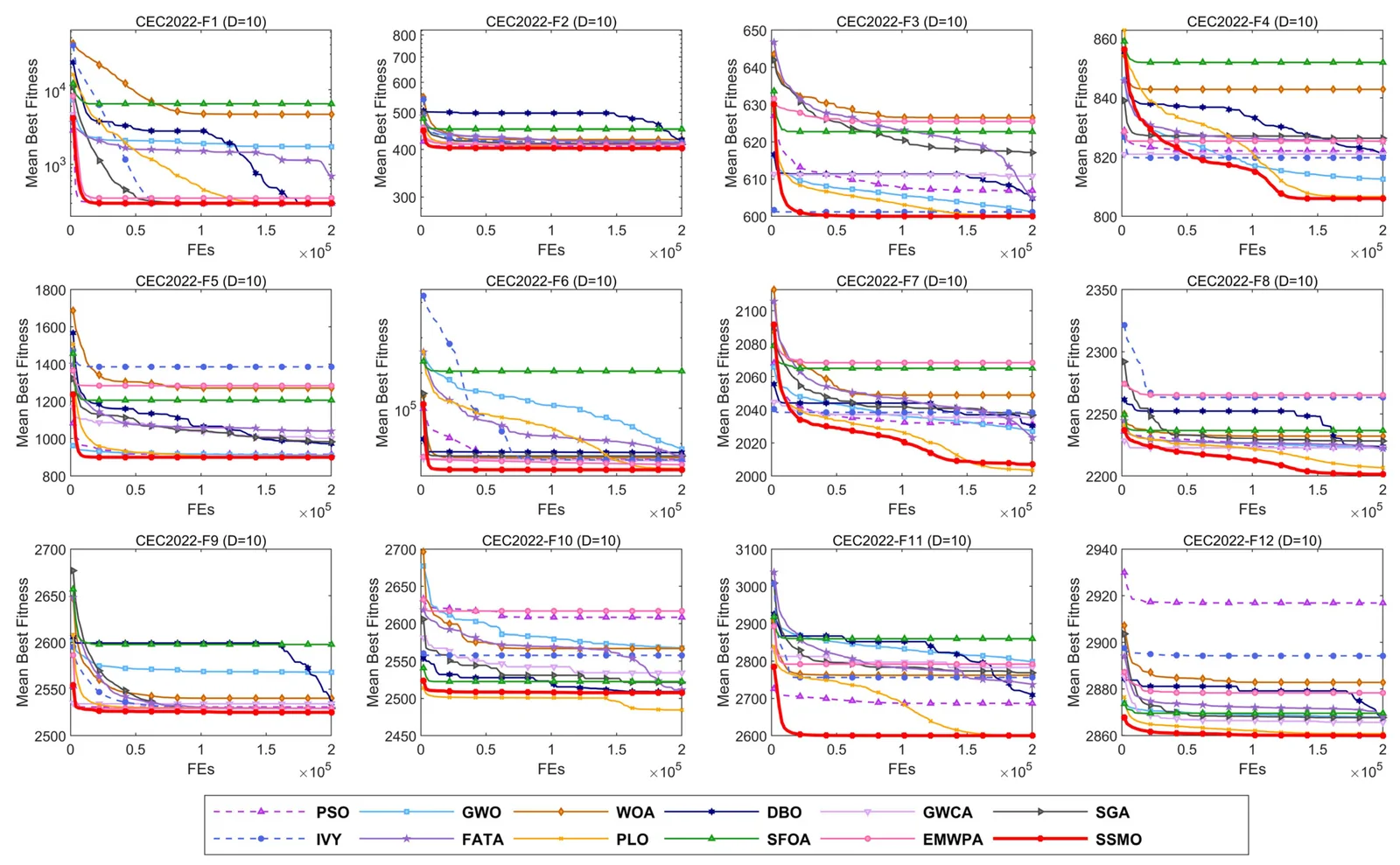
The comparison curves in the CEC 2022 test suite (Dim = 10).

**Figure A8:**
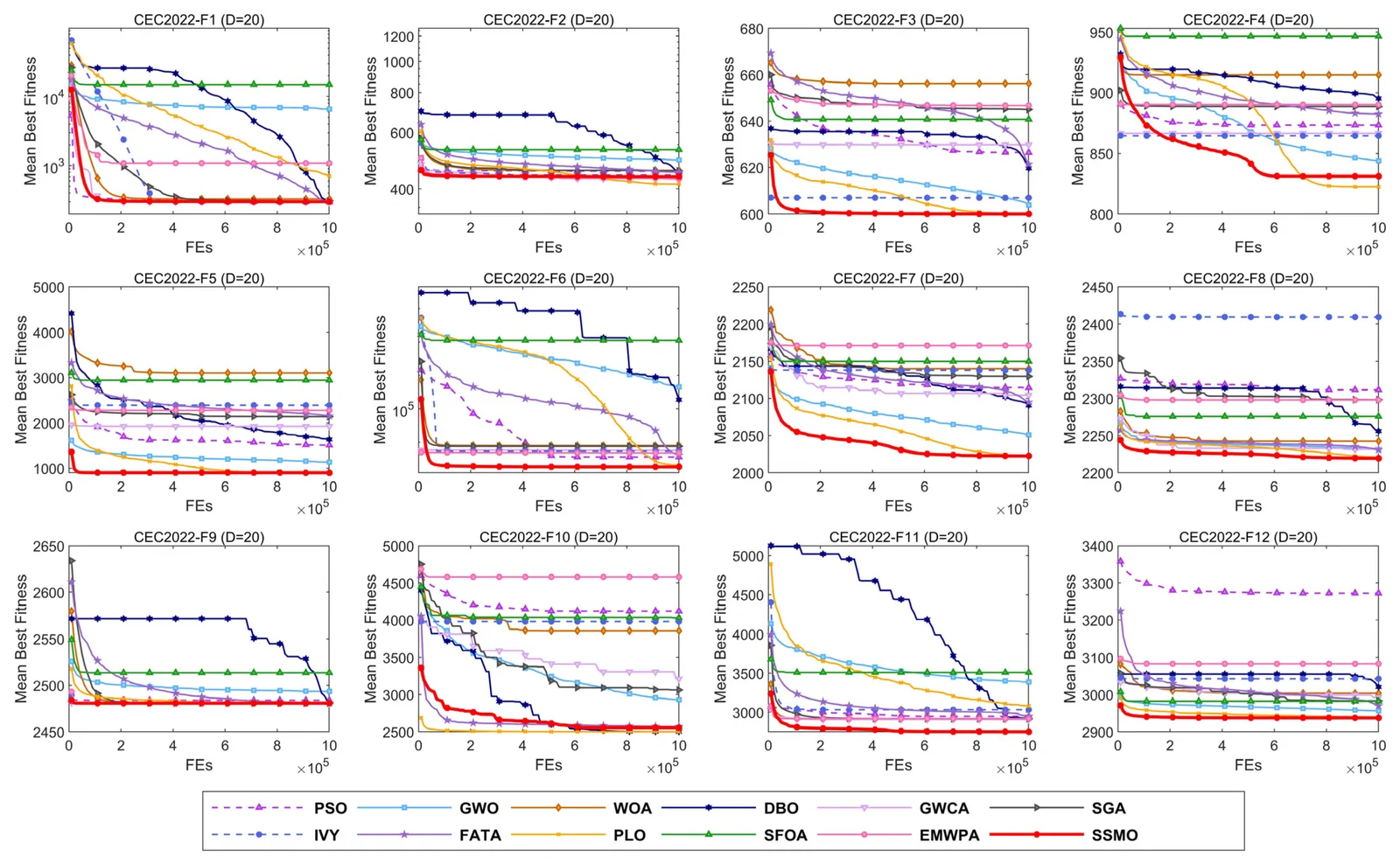
The comparison curves in the CEC 2022 test suite (Dim = 20).

**Figure A9:**
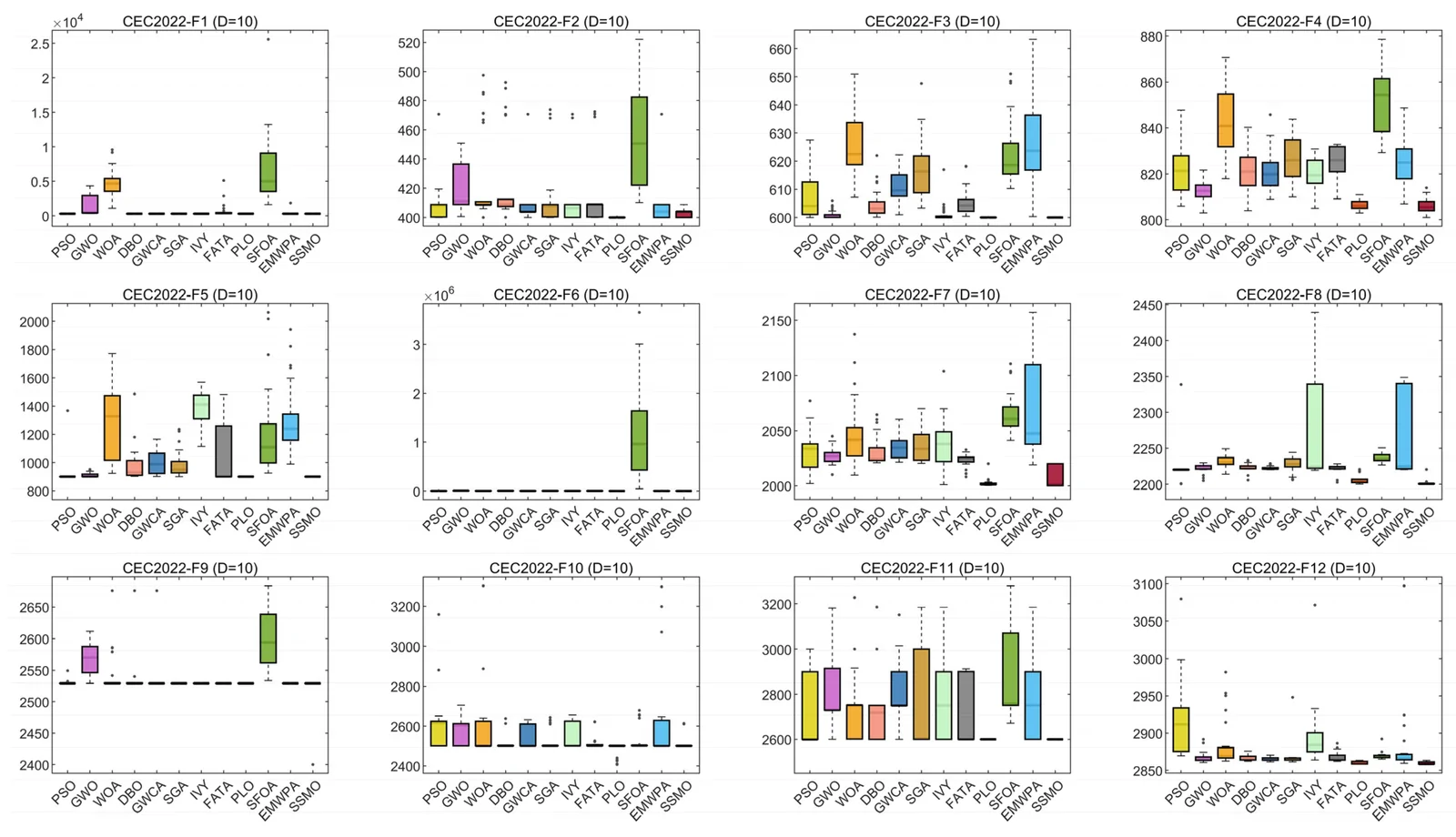
Box plot comparison of algorithms in the CEC 2022 test suite (Dim = 10).

**Figure A10:**
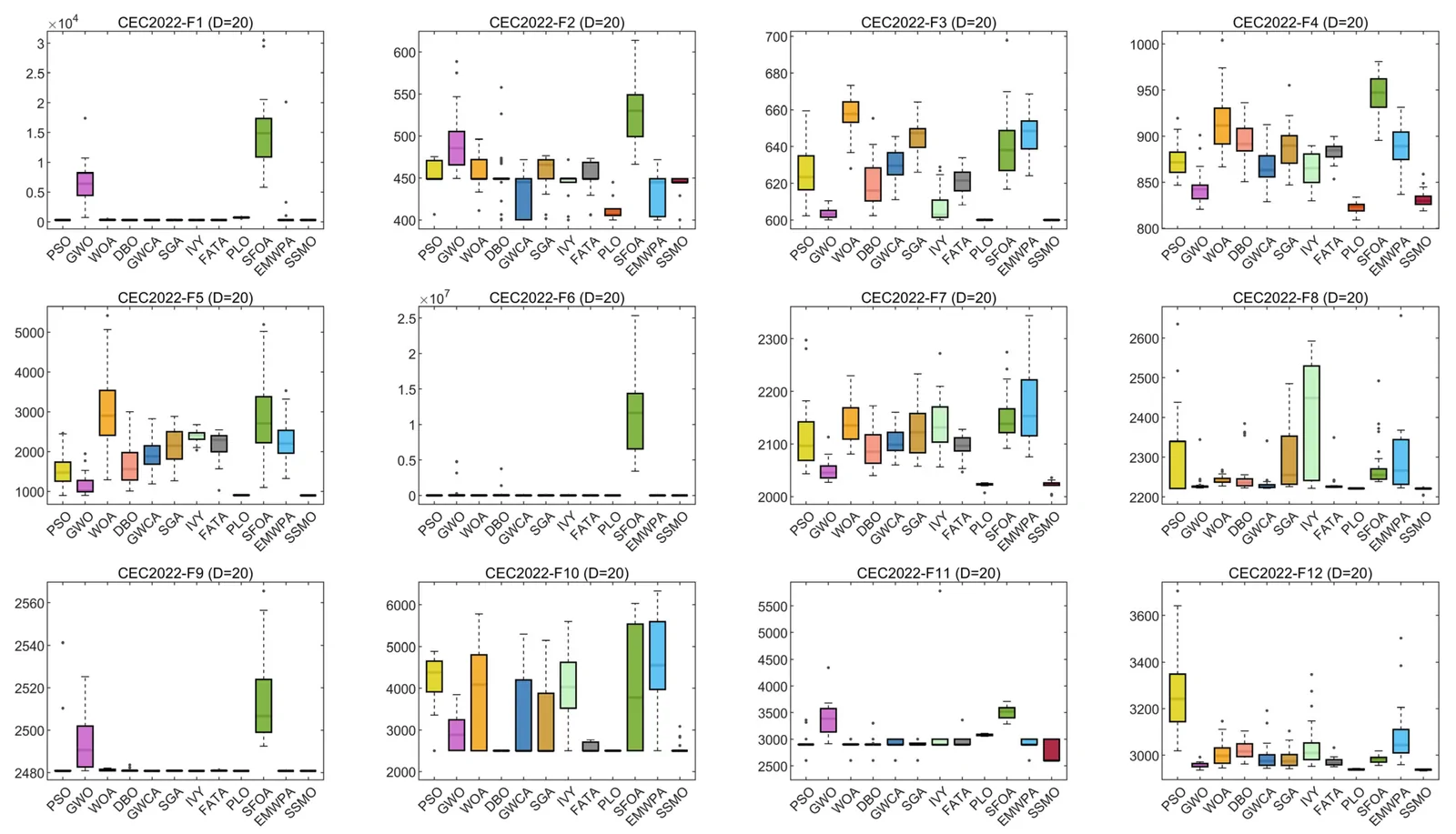
Box plot comparison of algorithms in the CEC 2022 test suite (Dim = 20).

Figure 13 and Figure 14 show the convergence curves and box plot results of selected functions from the CEC2022 suite. The convergence curves indicate that SSMO performs fast early-stage improvement and continues to refine the best solutions in later iterations. The box plots further show that SSMO generally produces lower and more concentrated objective values in the selected functions, reflecting good convergence accuracy and stability. Figure A7, Figure A8, Figure A9 and Figure A10 in Appendix B present the complete convergence curves and box plots for all CEC2022 functions.

Table 7 reports the results of Wilcoxon rank-sum tests comparing SSMO with the competing algorithms in CEC2022. For each function and dimensional setting, Holm’s correction is applied to the 11 comparisons involving SSMO at a family-wise significance level of 0.05. The outcomes are summarized across the 12 functions as win/tie/loss counts, corresponding to “+/=/−”. The aggregate counts are 117/8/7 at 10D and 109/12/11 at 20D. SSMO records more wins than losses against every competing algorithm in both settings, although the extent of its advantage varies among competitors. As the dimension increases from 10D to 20D, the number of wins decreases, while the numbers of ties and losses increase. Nevertheless, wins remain dominant in both settings, indicating favorable relative performance under the adopted evaluation budgets.

**Figure 15:**
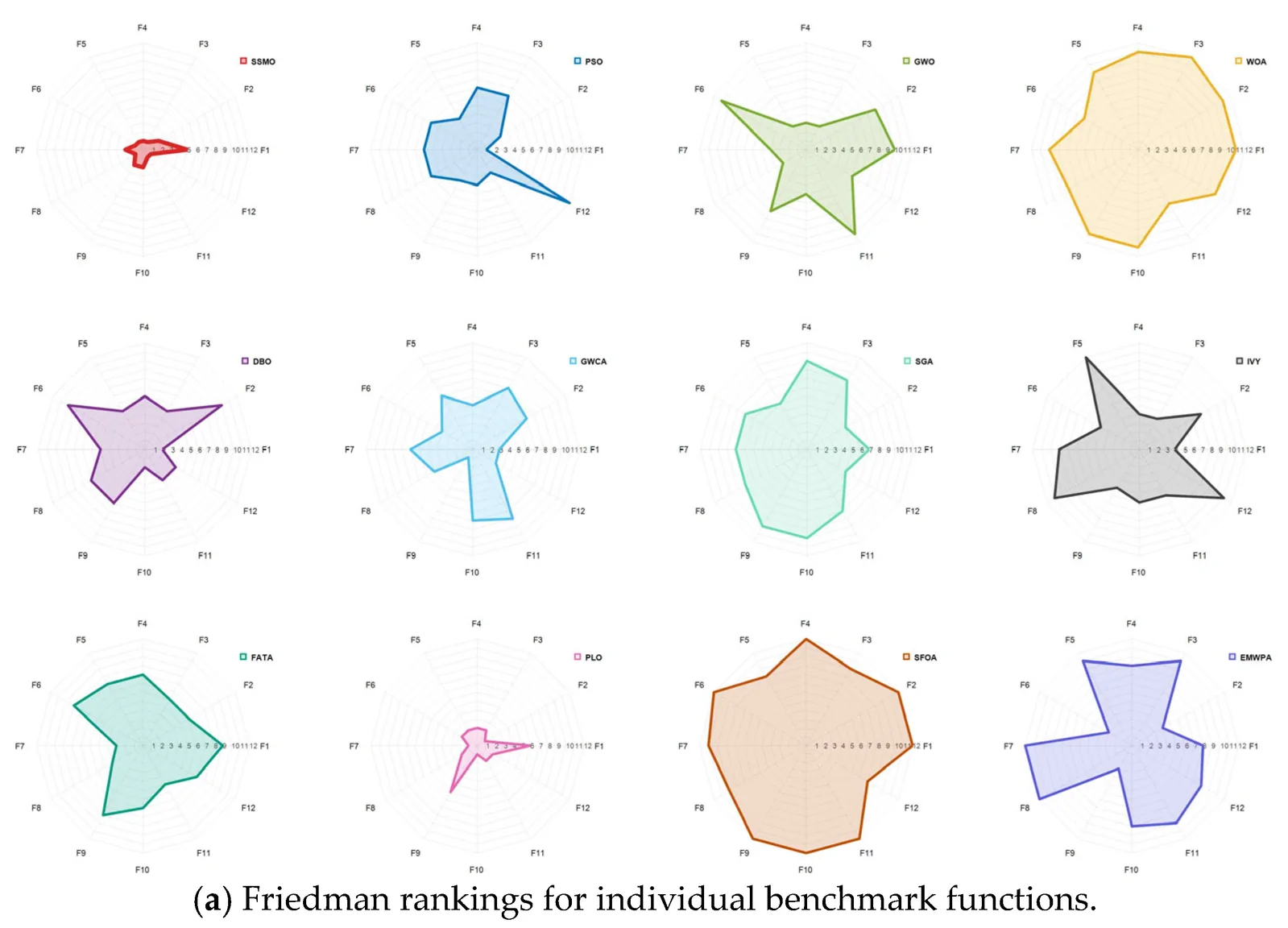

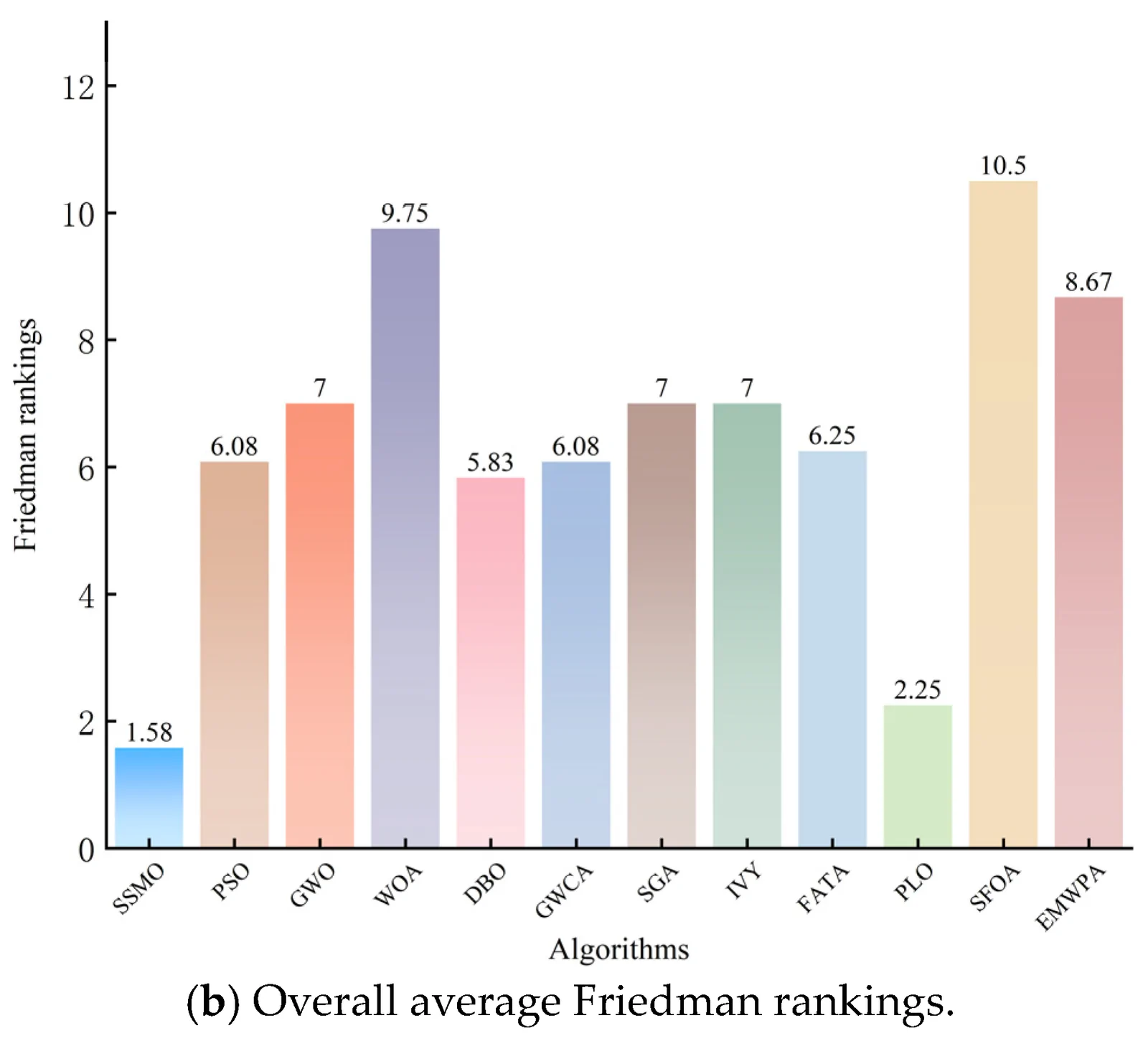
Friedman ranking results for the 10-dimensional CEC 2022 benchmark functions.

**Figure 16:**
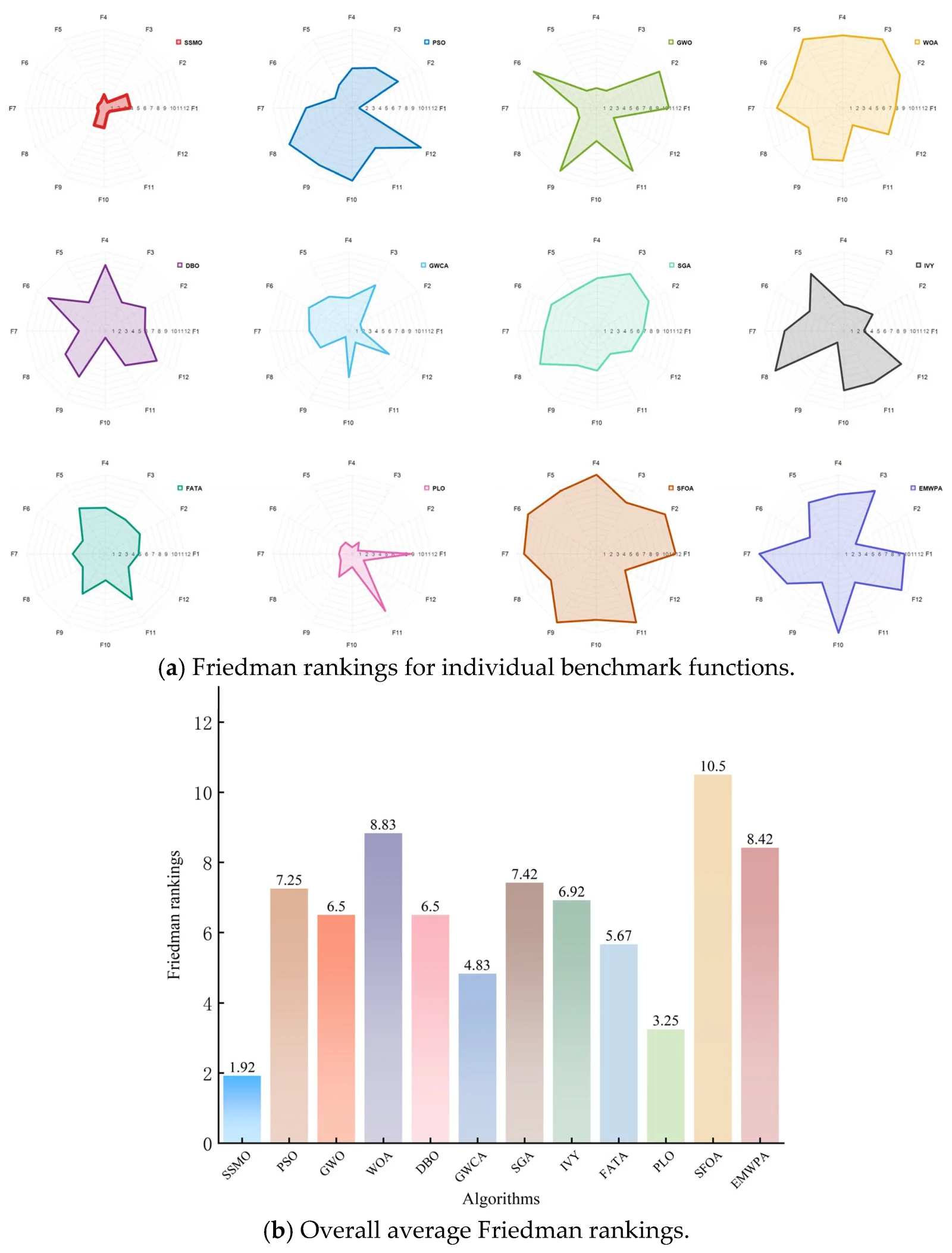
Friedman ranking results for the 20-dimensional CEC 2022 benchmark functions.

Figure 15 and Figure 16 present the function-wise and overall average Friedman rankings. For each function and dimensional setting, the algorithms are ranked according to their mean final objective values, with lower values receiving better ranks and equal values receiving average ranks. The overall average rank is calculated across the 12 functions, with equal weight assigned to each function. The radar charts display the function-wise ranks, while the bar charts show the overall average ranks. SSMO obtains the lowest average ranks of 1.58 and 1.92 at 10D and 20D, respectively. Although its average rank increases with dimension, SSMO retains the lowest average rank in both configurations. These results indicate favorable relative performance in CEC2022 within the tested dimensional range and under the corresponding function-evaluation budgets.

### 3.5. Comparison with DE Variants

The wind-driven migration mechanism of SSMO uses the positional difference between two randomly selected individuals to generate a search direction. This operation is conceptually related to the differential-vector principle of differential evolution (DE), although the complete update mechanism of SSMO differs from that of DE. To further examine the performance of SSMO in relation to this established search principle, four representative DE variants are included for comparison: SaDE, L-SHADE-EpSin, L-SHADE-SPACMA, and RSDE. SaDE adapts its trial-vector generation strategies and control parameters according to their previous search performance. L-SHADE-EpSin introduces an ensemble sinusoidal scheme for adapting the scaling factor, whereas L-SHADE-SPACMA combines semi-parameter adaptation with a modified CMA-ES framework. RSDE is a recent DE variant based on a ring sub-population architecture and a conditional similarity selection mechanism. These methods represent different developments in strategy adaptation, parameter adaptation, hybrid search, and population-structure design within the DE framework.

The experimental settings are consistent with those used in the preceding benchmark comparisons. Each algorithm is independently run 30 times under the same search bounds, dimensional settings, and function-evaluation budgets. The algorithm-specific parameters of the four DE variants follow their original publications [7,8,9,10], except for the population-size settings, which are specified in Section 3.1. The 29 valid functions of CEC2017 are tested at *D* = 30, while the 12 functions of CEC2022 are tested at *D* = 10. The mean, standard deviation, best value, and function-wise rank are reported in Table 8 and Table 9. The function-wise ranks are determined according to the mean objective values, with a lower rank indicating better performance.

As shown in Table 8, SSMO obtains the lowest average rank of 2.10, followed by L-SHADE-SPACMA, L-SHADE-EpSin, RSDE, and SaDE, with average ranks of 2.34, 3.21, 3.66, and 3.69, respectively. SSMO ranks first in 12 of the 29 functions, namely F7, F9, F11, F13–F15, F19, F23, F26, and F28–F30, and ranks among the top two algorithms in 20 functions. L-SHADE-SPACMA ranks first in nine functions: F1, F4, F5, F8, F10, F12, F18, F21, and F22. RSDE ranks first in four functions, whereas L-SHADE-EpSin and SaDE each rank first in two functions. Although SSMO ranks fifth in F1 and F12, it achieves the lowest average rank and the largest number of first-place results, indicating strong overall performance in the CEC2017 suite.

As reported in Table 9, SSMO again obtains the lowest average rank of 2.17, followed by SaDE, L-SHADE-SPACMA, RSDE, and L-SHADE-EpSin, with average ranks of 3.00, 3.04, 3.29, and 3.50, respectively. SSMO ranks first in F5, F6, F8, and F11 and ranks among the top two algorithms in 8 of the 12 functions. RSDE ranks first in F2, F3, F9, and F12 and shares the lowest mean with SaDE in F1. It therefore achieves the lowest mean in five functions, including the tied result, but ranks fifth in six functions. These results indicate that RSDE performs particularly well in some functions but shows substantial performance variation across the benchmark suite. In comparison, SSMO ranks fifth only in F3 and achieves a top-two rank in more functions, indicating more balanced performance across the CEC2022 suite.

Overall, SSMO obtains the best average rank in both benchmark suites and remains competitive with the four DE variants. The results also suggest that the performance of SSMO cannot be attributed solely to the use of a differential search direction. Instead, its overall search behavior results from the combined effects of elite guidance, wind-driven migration, molting memory guidance, and adaptive foraging.

### 3.6. Ablation Study

To examine the contribution of each mechanism within the complete SSMO framework, four reduced variants were constructed. SSMO-oElite, SSMO-oWind, SSMO-oMemory, and SSMO-oForaging remove elite guidance, wind-driven migration, molting memory guidance, and adaptive foraging, respectively. Only one mechanism is removed in each variant, while the other three mechanisms and their parameter settings remain unchanged. This design evaluates the effect of removing each mechanism when the remaining mechanisms are active.

The experiments were conducted in the 29 CEC2017 functions in 30 dimensions. The population size, computational budget, and number of independent runs were the same as those used in the main 30D experiment. The mean and standard deviation of the final objective values are reported in Table 10. The last row gives the win/tie/loss results at the 0.05 significance level.

The complete SSMO outperforms the four reduced variants in most functions. The win/tie/loss results are 24/3/2 against SSMO-oElite, 16/12/1 against SSMO-oWind, 27/2/0 against SSMO-oMemory, and 24/5/0 against SSMO-oForaging. Across the four comparisons, SSMO obtains 91 wins, 22 ties, and only 3 losses.

Among the four removal comparisons, SSMO records the largest number of wins against SSMO-oMemory, with 27 wins and no losses. This result suggests that retaining molting memory guidance is beneficial in most tested functions when the other three mechanisms remain active. Elite guidance also has a clear effect. Its removal causes large increases in the mean objective values in F1, F12, F13, F18, and F30.

The foraging strategy is also important. SSMO records 24 wins and no losses against SSMO-oForaging. Removing adaptive foraging substantially increases the mean objective values in F1, F3, F4, F12, and F30. These results suggest that retaining the foraging mechanism benefits solution quality in these functions in the presence of the other three mechanisms. The wind-driven mechanism has a more function-dependent effect, with 16 wins, 12 ties, and 1 loss. However, the clear degradation in F9, F14, F18, and F19 shows that this mechanism provides useful alternative search directions in several complex functions.

Overall, the complete SSMO generally performs better than the four reduced variants in the tested 30D CEC2017 functions, although the effect of removing each mechanism varies across functions. These results support retaining the four mechanisms in the evaluated configuration. However, the comparisons assess the contribution of each mechanism only when the other three remain active. They do not isolate its standalone effect or quantify interactions among mechanisms.

### 3.7. Parameter Sensitivity Analysis

To examine the influence of the main parameters and select a working configuration for SSMO, a one-factor-at-a-time sensitivity analysis was conducted for the elite proportion *L*/*N*, the reference-pool proportion *M*/*N*, the standard deviation of wind intensity *σ_η_*, and the foraging-parameter scale *q*. The first two parameters determine the sizes of the population subsets used to guide migration. The parameter *σ_η_* controls the variability of wind intensity, while *q* jointly scales the perturbation probabilities and amplitudes in the two foraging states.

The experiments were conducted in 29 CEC2017 benchmark functions in 30 dimensions. The maximum number of function evaluations was set to 300,000. Each parameter setting was evaluated over 30 independent runs. One parameter was varied at a time, while the remaining settings were kept unchanged. The candidate values and adopted settings are listed in Table 11.

**Figure 17:**
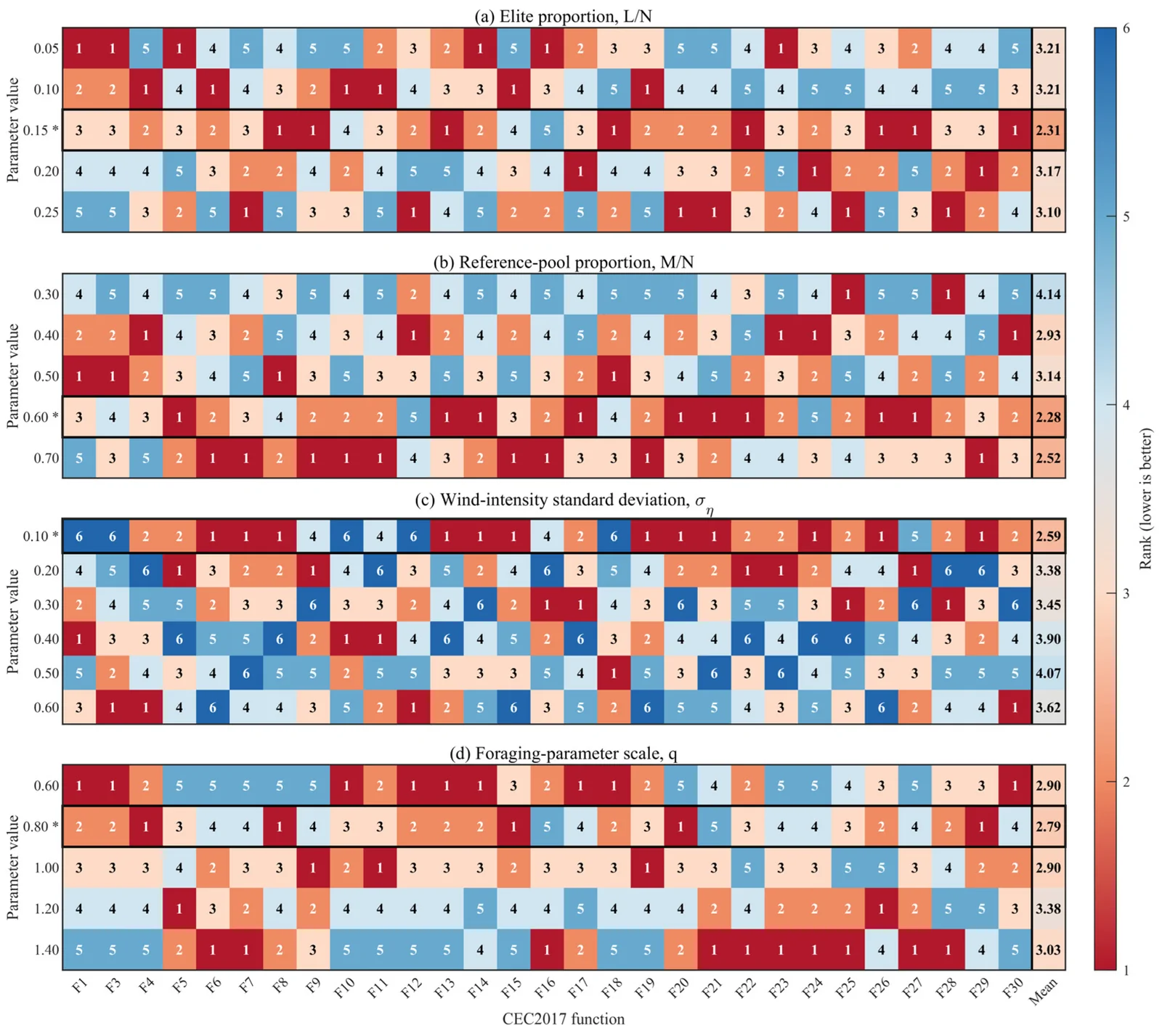
Ranking heatmaps of candidate SSMO parameter values in the 30D CEC2017 benchmark suite. An asterisk (*) indicates the selected value of each parameter, which yields the lowest mean rank among the tested candidates.

For each run, the final error was calculated as the nonnegative difference between the final objective value and the known optimum. A lower bound of 10^−16^ was applied before taking the base-10 logarithm. For each function, candidate values within the same parameter family were ranked according to their mean log-transformed final errors over the independent runs. Rank 1 indicates the lowest value, and equal values receive average ranks. The overall average rank was calculated across the 29 functions, with equal weight assigned to each function. Figure 17 presents the function-wise ranks and the average rank of each candidate setting.

The results for *L*/*N* and M/N are shown in Figure 17a,b. For *L*/*N*, the value 0.15 achieves the lowest mean rank of 2.31. Both smaller and larger elite proportions lead to higher mean ranks. This indicates that an excessively small elite set provides limited guidance, whereas a larger elite set may reduce the selectivity of the migration process. An elite proportion of 0.15 provides a suitable balance between these two effects.

The reference-pool proportion has a more evident influence on performance. The lowest mean rank of 2.28 is obtained at 0.60, followed by 0.70 with a mean rank of 2.52. By contrast, 0.30 produces the highest mean rank of 4.14. A small reference pool may limit the number of alternative migration directions available to the population. Overall, moderate-to-large values perform better than 0.30, while increasing the ratio beyond 0.60 provides no further improvement. Therefore, 0.60 was selected.

Figure 17c shows that the lowest mean rank of 2.59 is obtained when *σ_η_* is set to 0.10. Increasing the standard deviation does not provide consistent improvement across the test functions. A small value of *σ_η_* limits excessive fluctuations in the wind-driven search while retaining the stochastic variation required for exploration. Thus, 0.10 was adopted.

For the foraging-parameter scale, the value 0.80 achieves the best mean rank of 2.79, as shown in Figure 17d. The neighboring values 0.60 and 1.00 both obtain a mean rank of 2.90. The small difference among these three values indicates that SSMO is relatively insensitive to q within this range. However, values above 1.00 do not improve the overall ranking. Accordingly, 0.80 was selected.

Based on these comparisons, *L*/*N* = 0.15, *M*/*N* = 0.60, *σ_η_* = 0.10, and *q* = 0.80 were adopted as a working configuration for SSMO. The one-factor-at-a-time analysis characterizes the influence of each parameter while the remaining parameters are held at their reference values. The observed preferences therefore apply to the tested ranges and reference configuration, while possible interactions among parameters may influence their effects under other configurations.

The parameter settings selected in CEC2017 at 30D were used unchanged in the CEC2017 experiments at 50D and 100D and the CEC2022 experiments at 10D and 20D. Results from these experiments were not used to select or adjust the parameters. Since CEC2017 at 30D was used for both parameter selection and performance evaluation, its results are considered together with those obtained at higher dimensions and in CEC2022 to assess the performance of the selected configuration.

## 4. Application of SSMO to Engineering Design Problems

To further assess the practical applicability of SSMO, five classical engineering optimization problems are considered in this section. For these test cases, SSMO and all competing algorithms use the same parameter settings as those adopted in the preceding experiments. Specifically, the population size is set to 30, and each run is limited to 15,000 function evaluations. Each algorithm is independently executed 30 times. The design variables and mean objective function values obtained over the 30 runs are recorded for performance evaluation.

For all constrained engineering design problems, the inequality and equality constraints were handled using a common exterior quadratic penalty function:

$$F\left(X\right)=f\left(X\right)+\lambda \left[\sum_{i=1}^{m}{\left(\max\left(0,g_{i}\left(X\right)\right)\right)}^{2}+\sum_{j=1}^{p}h_{j}^{2}\left(X\right)\right]$$

where *f*(*X*) is the original objective function, and *g_i_*(*X*) and *h_j_*(*X*) denote the inequality and equality constraints, respectively. The penalty coefficient is fixed at *λ* = 10^10^. The same penalty formulation and coefficient are applied to SSMO and all competing algorithms for the four constrained problems. For the unconstrained gear train problem, the penalty term is zero.

The fixed coefficient is used to discourage constraint violations and maintain a consistent evaluation setting across algorithms. Its effective strength depends on the scales of the objective function and constraint residuals, which vary among the engineering problems. The balance between objective minimization and constraint satisfaction is therefore problem dependent, and the penalty setting may influence the search behavior and relative performance of the algorithms. The reported comparisons are interpreted under the adopted penalty formulation and coefficient.

### 4.1. Pressure Vessel Design

**Figure 18:**
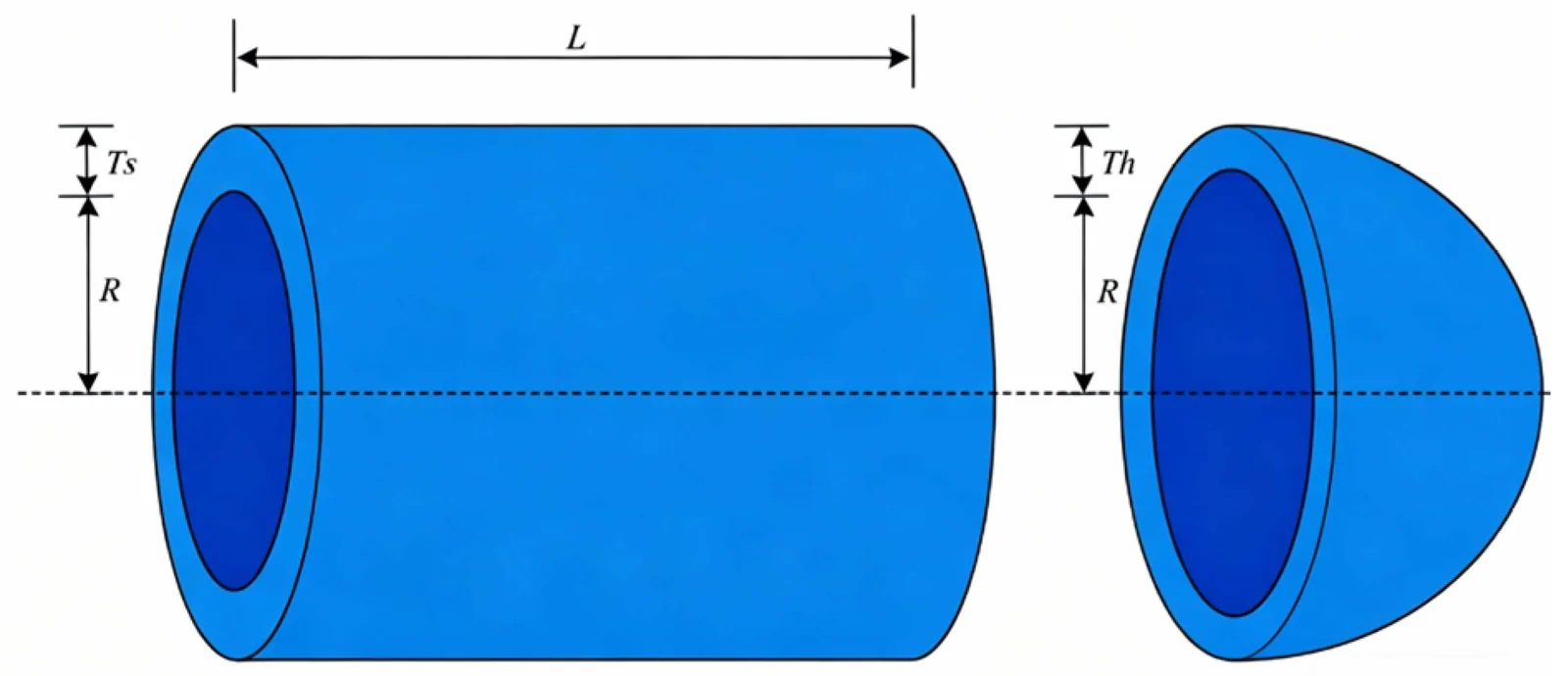
Schematic view of the pressure vessel design.

Figure 18 shows the pressure vessel design problem [45]. The objective of this problem is to minimize the total cost. It involves four decision variables: the shell thickness *T_s_*, head thickness *T_h_*, inner radius *R*, and cylindrical section length *L*. The mathematical formulation of this problem is given as follows:

Consider:

*X* = [*x*_1_, *x*_2_, *x*_3_, *x*_4_] = [*T_s_*, *T_h_*, *R*, *L*]

Objective function:

$$\mathrm{minf}\left(X\right)=3.1661x_{1}^{2}x_{4}+19.84x_{1}^{2}x_{3}+1.7781x_{2}x_{3}^{2}+0.6224x_{1}x_{3}x_{4}$$

Subject to:

$$\begin{array}{l} \begin{array}{l} g_{1}\left(X\right)=-x_{1}+0.0193x_{3}\leq 0\\ g_{2}\left(X\right)=-x_{2}+0.00954x_{3}\leq 0 \end{array}\\ \begin{array}{l} g_{3}\left(X\right)=-\pi x_{3}^{2}x_{4}-\frac{4}{3}\pi x_{3}^{3}+1296000\leq 0\\ g_{4}\left(X\right)=x_{4}-240\leq 0 \end{array} \end{array}$$

Variable Range:

$$\begin{array}{c} x_{1},x_{2}\in \left\{0.0625k|k=1,2,\ldots ,99\right\}\\ 10\leq x_{3},x_{4}\leq 200 \end{array}$$

Table 12 presents the reported design variables and statistical results for the pressure vessel design problem. SSMO ranks first with the lowest mean value of 6065.9 and the smallest standard deviation of 12.5359, followed by PLO and GWO in terms of mean performance. SSMO obtains a best objective value of approximately 6059.7, while DBO, GWCA, IVY, and EMWPA achieve comparable best values in individual runs. However, their higher mean values and standard deviations indicate lower consistency. SSMO obtains the lowest mean final fitness and the smallest run-to-run standard deviation among the compared algorithms under the adopted settings.

### 4.2. Welded Beam Design

**Figure 19:**
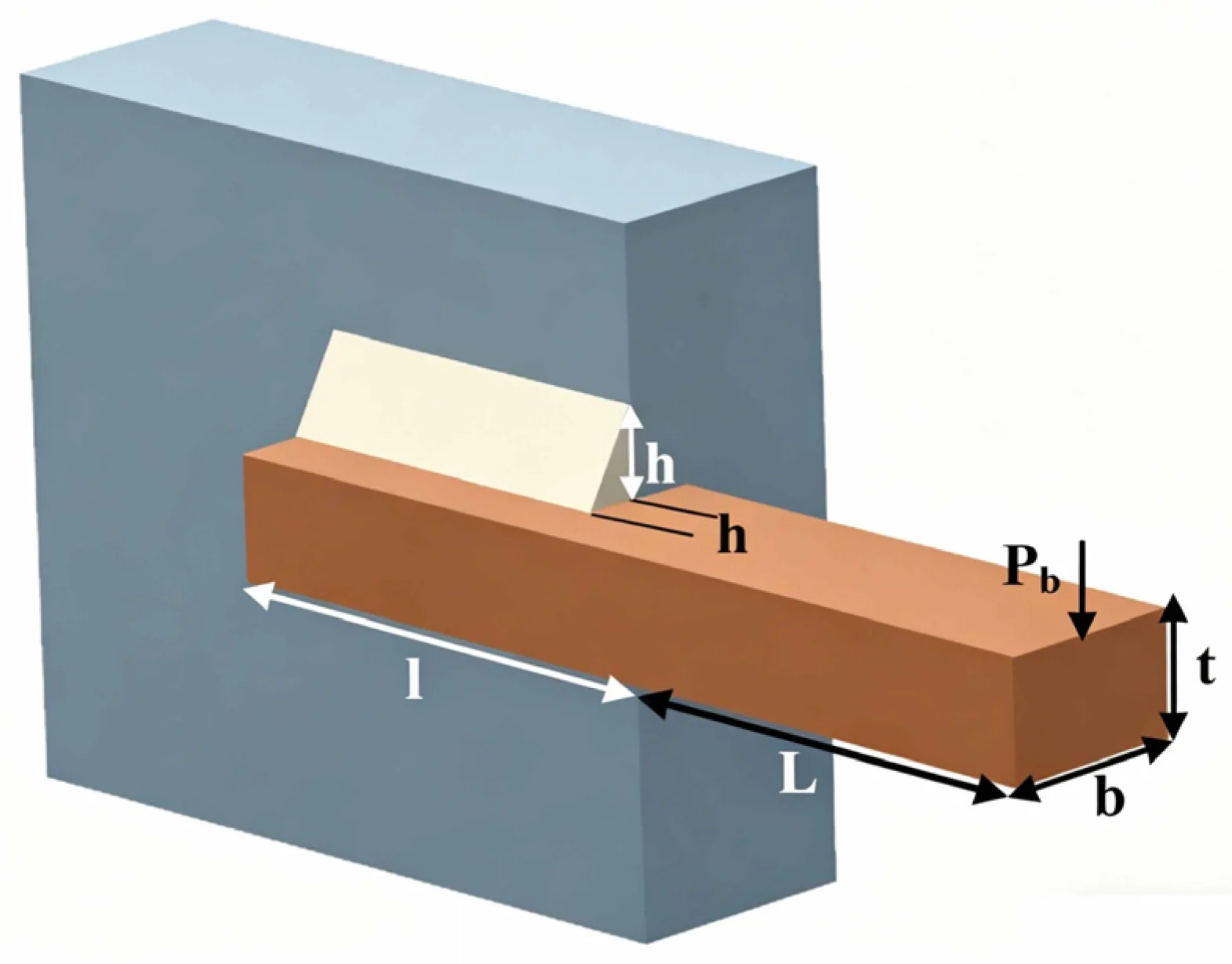
Schematic view of the welded beam design.

The welded beam design problem [46] aims to minimize the manufacturing cost of a welded beam structure, as illustrated in Figure 19. This problem is subject to five constraints related to shear stress, bending stress, buckling load, and end deflection. The design variables include weld thickness *h*, clamping bar length *l*, bar height *t*, and bar thickness *b*. The mathematical formulation of this problem is given as follows:

Consider:

*X* = [*x*_1_, *x*_2_, *x*_3_, *x*_4_] = [*h*, *l*, *t*, *b*]

Objective function:

$$\min f\left(X\right)=1.10471\;x_{1}^{2}x_{2}+0.04811\;x_{3}x_{4}\left(14+x_{2}\right)$$

Subject to:

$$\begin{array}{l} g_{1}\left(X\right)=\tau \left(X\right)-\tau_{\max}\leq 0\\ g_{2}\left(X\right)=\sigma \left(X\right)-\sigma_{\max}\leq 0\\ g_{3}\left(X\right)=x_{1}-x_{4}\leq 0\\ g_{4}\left(X\right)=\delta \left(X\right)-\delta_{\max}\leq 0\\ g_{5}\left(X\right)=P-P_{c}\left(X\right)\leq 0 \end{array}$$

where:

τX=τ′2+2τ′τ″x22R+τ″2,τ′=P2x1x2,τ″=MRJM=PL+x22,R=x224+x1+x322,σX=6PLx4x32J=22x1x2x224+x1+x322,δX=6PL3Ex4x32PCX=4.013EL2x46x32301−x32LE4GP = 6000 lb , L = 14 in , δmax= 0.25 in , E = 30 ×10 6 psiτmax= 136000psi, G = 12 ×10 6 psi, σmax= 30000 psi

Variable range:

0.125 ≤ *x*_1_ ≤ 2

0.1 ≤ *x*_2_, *x*_3_ ≤ 10

0.1 ≤ *x*_4_ ≤ 2

The results for the welded beam design problem are summarized in Table 13. SSMO achieves the lowest mean objective value of 1.6753. It also records the smallest standard deviation of 2.9670 × 10^−3^, indicating stable optimization performance. GWO and FATA rank second and third, respectively, with mean values close to that obtained by SSMO, whereas the remaining algorithms exhibit larger performance variations. In particular, WOA produces the highest mean value and standard deviation and ranks last. This combination of accuracy and stability gives SSMO a slight but consistent advantage over the competing algorithms.

### 4.3. Tension/Compression Spring Design

**Figure 20:**
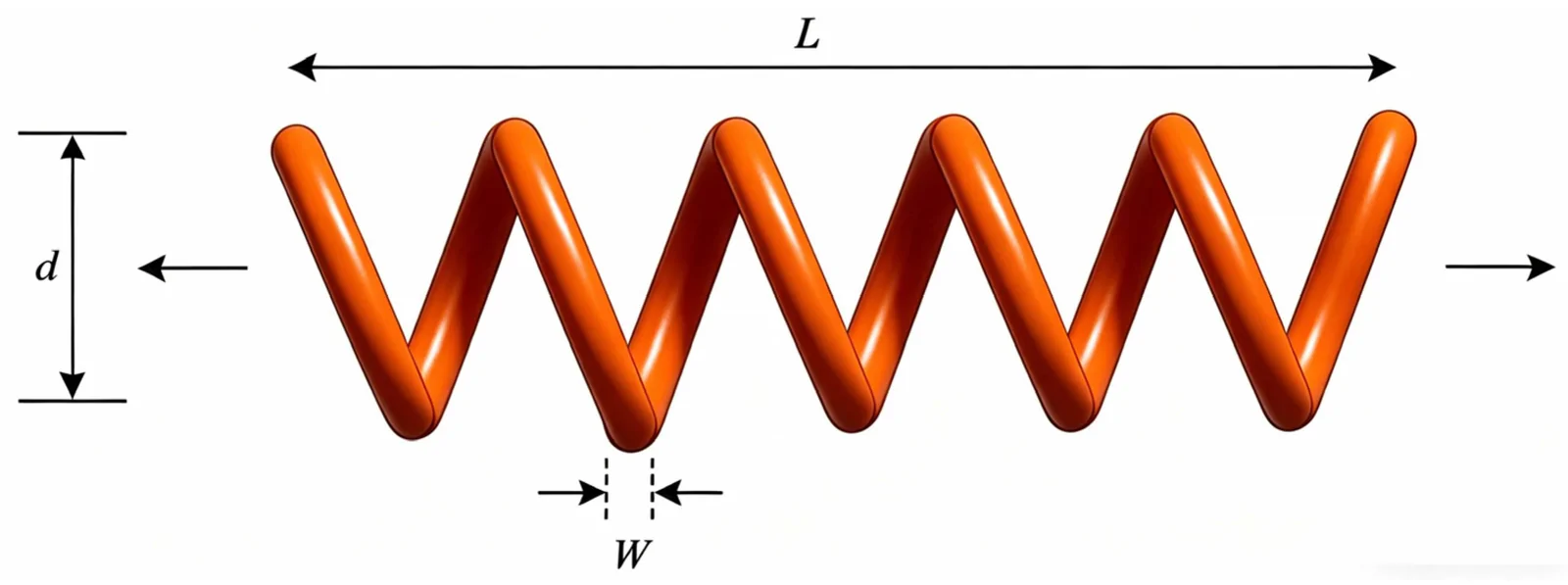
Schematic view of the tension/compression spring design.

The primary objective of the tension/compression spring design problem [47] is to minimize the weight of the spring. A schematic illustration of this problem is provided in Figure 20. This design problem involves several constraints related to minimum deflection, shear stress, surge frequency, and outer diameter limits. The optimization variables are the wire diameter *d*, mean coil diameter *D*, and number of active coils *N*. Accordingly, the mathematical model of this problem is expressed as follows:

Consider:

*X* = [*x*_1_, *x*_2_, *x*_3_] = [*d*, *D*, *N*]

Objective function:

$$\mathrm{minf}\left(X\right)=\left(x_{3}+2\right)\cdot x_{2}\cdot x_{1}^{2}$$

Subject to:

$$\begin{array}{l} g_{1}\left(X\right)=1-\frac{x_{3}\cdot x_{2}^{3}}{71785\times x_{1}^{4}}\leq 0\\ \begin{array}{l} g_{2}\left(X\right)=\frac{4\cdot x_{2}^{2}-x_{1}\cdot x_{2}}{12566\cdot \left({x_{2}x_{1}^{3}-x}_{1}^{4}\right)}+\frac{1}{5108\times x_{1}^{2}}-1\leq 0\\ g_{3}\left(X\right)=1-\frac{140.45\cdot x_{1}}{x_{2}^{2}\cdot x_{3}}\leq 0\\ g_{4}\left(X\right)=\frac{x_{1}+x_{2}}{1.5}-1\leq 0 \end{array} \end{array}$$

Variable range:

$$\begin{array}{c} 0.05\leq x_{1}\leq 2.00\\ 0.25\leq x_{2}\leq 1.30\\ 2.00\leq x_{3}\leq 15.00 \end{array}$$

Table 14 reports the comparative results for the tension/compression spring design problem. SSMO ranks first with the lowest mean objective value of 1.2706 × 10^−2^, followed by PLO and GWO. It also achieves the smallest standard deviation of 5.4900 × 10^−5^, demonstrating reliable search performance. SSMO, WOA, and EMWPA obtain comparable best values of approximately 1.2665 × 10^−2^; however, the larger mean values and standard deviations of WOA and EMWPA indicate less consistent behavior. SSMO obtains the lowest mean final fitness and standard deviation among the compared algorithms for this problem.

### 4.4. Gear Train Design

**Figure 21:**
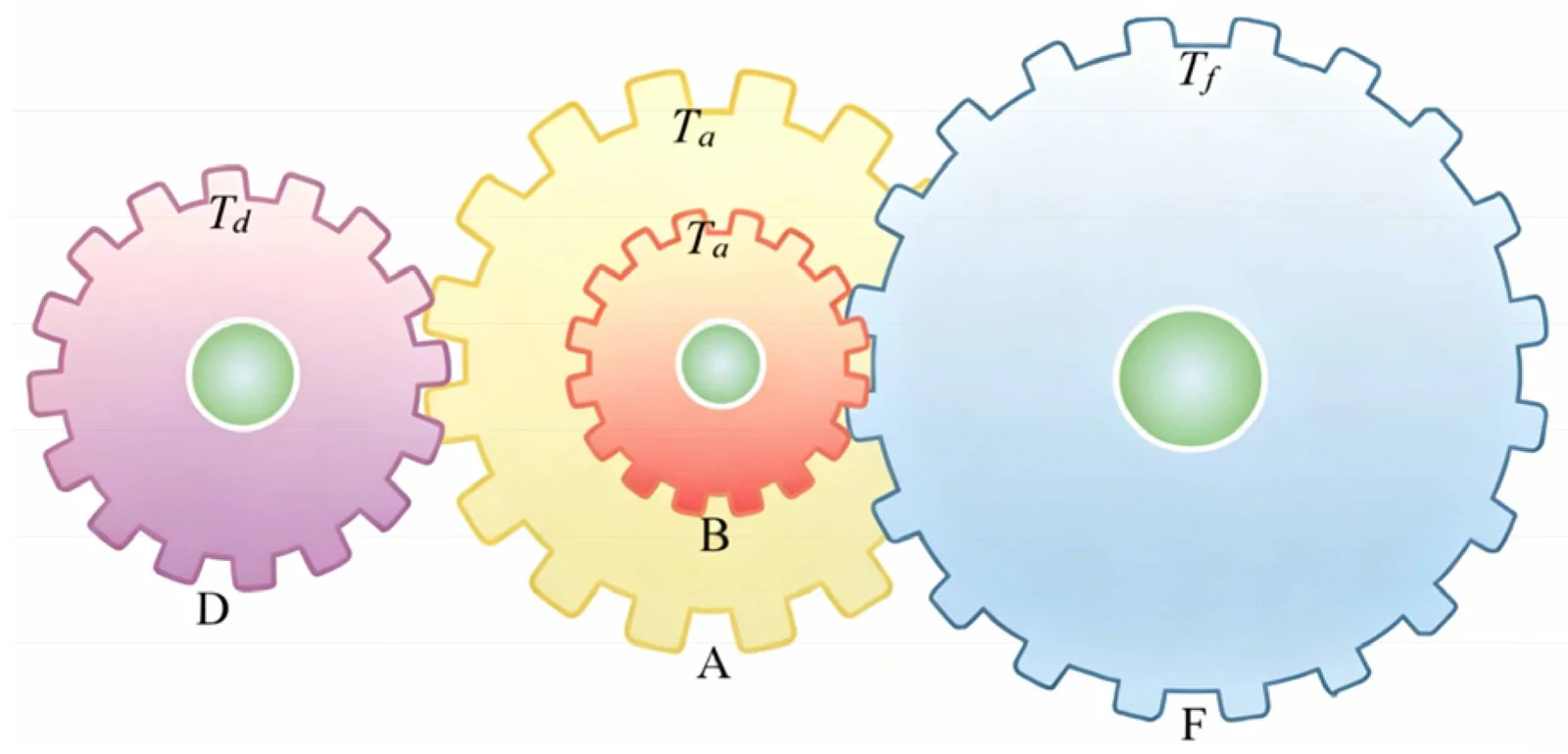
Schematic view of the gear train design.

As a representative unconstrained discrete optimization problem in mechanical engineering [48], the gear train design problem seeks to minimize the deviation in the transmission ratio. As shown in Figure 21, the model consists of four design variables, namely the numbers of teeth on four gears: *T_a_*, *T_b_*, *T_d_*, and *T_f_*. The number of teeth for each gear ranges from 12 to 60. The objective function is given as follows:

Consider:

*X* = [*x*_1_, *x*_2_, *x*_3_, *x*_4_] = [*T_a_*, *T_b_*, *T_d_*, *T_f_*]

Objective function:

$$\mathrm{minf}\left(X\right)={\left(\frac{1}{6.931}-\frac{T_{b}\cdot T_{d}}{T_{a}\cdot T_{f}}\right)}^{2}$$

Variable range:

12 ≤ *x_i_* ≤ 60, *i* = 1, 2, 3, 4

Table 15 summarizes the comparative results for the gear train design problem. For the gear train problem, each candidate solution is converted into integer tooth counts before objective-function evaluation. The four decision variables are rounded to the nearest integers and restricted to the interval [12, 60]. This decoding procedure is applied to all compared algorithms, and the reported design solutions contain the decoded integer tooth counts. SSMO ranks first with the lowest mean objective value of 5.6463 × 10^−11^ and the smallest standard deviation of 1.6680 × 10^−10^, followed by GWO and FATA. Several competing algorithms also attain the same best value of 2.7009 × 10^−12^ in individual runs; however, their larger mean values and standard deviations indicate that they reach this level of accuracy less consistently. In contrast, IVY and SFOA exhibit considerably higher mean errors. These results indicate favorable solution quality and low run-to-run variability under the adopted evaluation budget.

### 4.5. Robot Gripper Design

**Figure 22:**
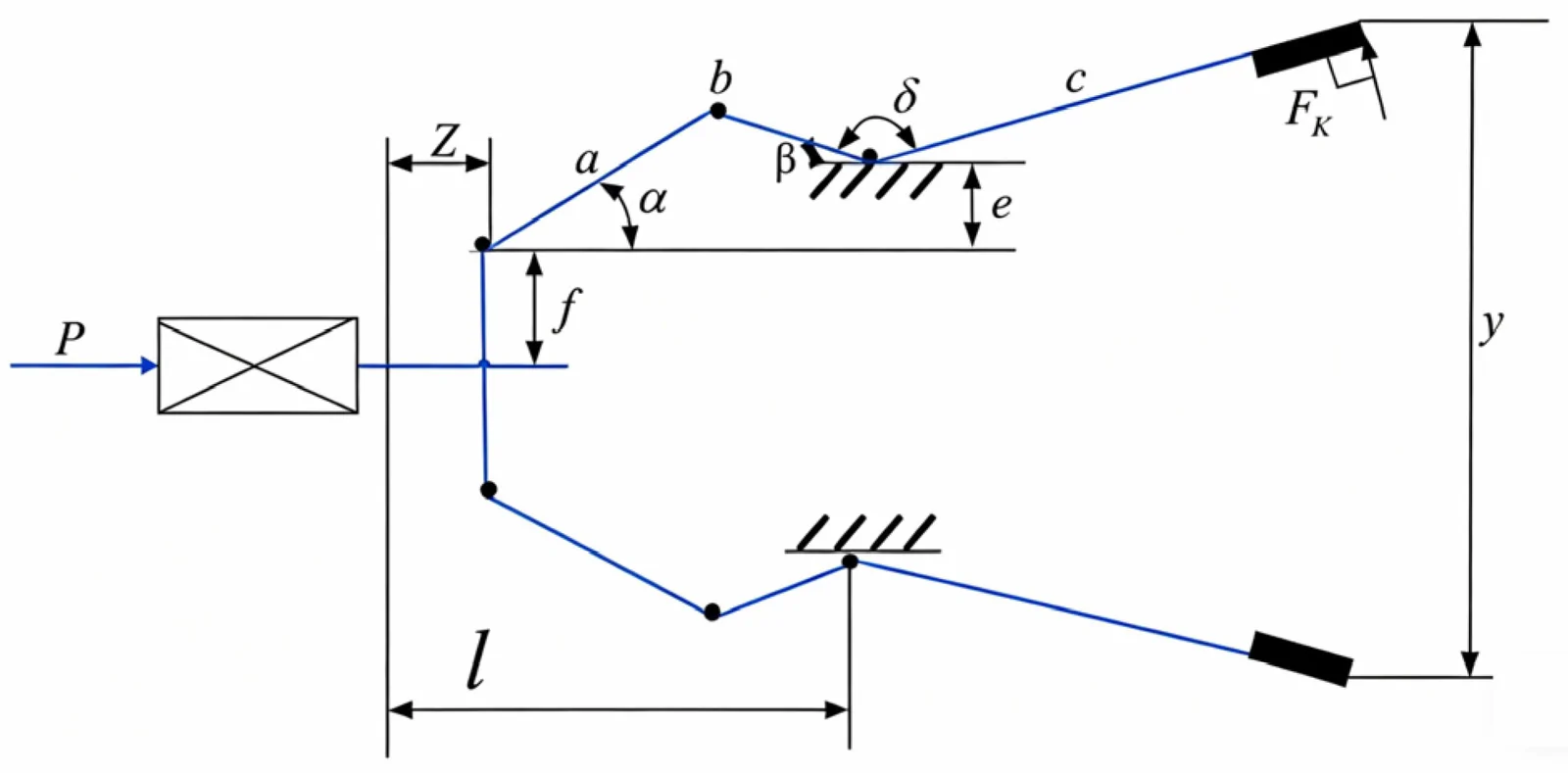
Schematic view of the robot gripper design.

Figure 22 shows the robot gripper design problem [49]. This problem aims to reduce the force variation of the gripper by minimizing the difference between the maximum and minimum gripping forces, which depends on the displacement range of the end effector. Seven design variables are considered for the gripper mechanism, namely the gripper widths *a* and *b*, gripper thickness *c*, maximum opening angle *δ*, vertical offset *e*, driving parameter *f*, and gripper length *l*. The corresponding mathematical model is formulated as follows:

Consider:

*X* = (*x*_1_, *x*_2_, *x*_3_, *x*_4_, *x*_5_, *x*_6_, *x*_7_) = (*a*, *b*, *c*, *e*, *f*, *l*, δ)

Objective function:

$$f\left(X\right)=-\underset{z}{\min}F_{k}\left(X,z\right)+\underset{z}{\max}F_{k}\left(X,z\right)$$

Subject to:

$$\begin{array}{l} g_{1}(X)\;=\;-Y_{\min}\;+\;y(x,Z_{\max})\;\leq \;0\\ g_{2}(X)\;=\;-y(x,Z_{\max})\;\leq \;0\\ g_{3}(X)\;=\;Y_{\max}\;-\;y(x,0)\;\leq \;0\\ g_{4}(X)\;=\;y(X,0)\;-\;Y_{G}\;\leq \;0\\ g_{5}(X)\;=\;l^{2}\;+\;e^{2}\;-\;(a\;+\;b{)}^{2}\;\leq \;0\\ g_{6}\left(X\right)=b^{2}-{\left(a-e\right)}^{2}-{\left(l-Z_{\max}\right)}^{2}\leq 0\\ g_{7}(X)=Z_{\max}-l\leq 0 \end{array}$$

where:

α=cos−1a2+g2−b22ag+ϕg=e2+z−l2β=cos−1b2+g2−a22bg−ϕϕ=tan−1el−z,y(X,z)=2(f+e+csin(β+δ))FK=Pbsinα+β2ccosαY min=50 ,Y max=100,Y G =150,Z max=100,P =100

Variable range:

0 ≤ *e* ≤ 50

100 ≤ *c* ≤ 200

10 ≤ *f*,*a*,*b* ≤ 150

1 ≤ *δ* ≤ 3.14

100 ≤ *l* ≤ 300

Table 16 presents the comparative results for the robot gripper design problem. SSMO achieves the lowest mean objective value of 2.6559, the smallest standard deviation of 0.15836, and the best single-run value of 2.5443, ranking first among all compared algorithms. PLO and FATA rank second and third, respectively, but exhibit higher mean values and greater variability. The unusually large values for SGA, IVY, and SFOA occur in the mean and standard deviation of the final penalized fitness, rather than in the design variables. These statistics are inflated by large final fitness values in several runs. Under the adopted penalty formulation, positive constraint residuals contribute quadratically to the fitness with a coefficient of 10^10^. The reported fitness statistics therefore reflect both the gripping-force objective and constraint penalties and should not be interpreted as physical force variation alone.

### 4.6. Constraint Satisfaction Analysis

A constraint assessment is conducted for the pressure-vessel, welded-beam, spring, and robot-gripper problems. For each algorithm and problem, the best-run design is selected according to the lowest final penalized fitness among the 30 runs in the dataset used for this assessment. Constraint violations are calculated using the corresponding unrounded design variables. The maximum inequality-constraint violation is defined as

$$V_{\max}\left(x\right)=\underset{j}{\max}\left\{\max\left(0,g_{j}\left(x\right)\right)\right\}$$

A value of zero indicates that the selected design satisfies all inequality constraints in the numerical evaluation, while a positive value represents the largest constraint violation. Table 17 summarizes the results. For the pressure-vessel problem, several selected designs exhibit small positive residuals, with the largest value being 1.4731 × 10^−6^. The selected SSMO design has a maximum violation of 3.1722 × 10^−7^. All selected welded-beam and robot-gripper designs have zero violations. For the spring problem, DBO, GWCA, and EMWPA exhibit positive residuals of the order of 10^−12^, while the remaining selected designs have zero violations. This assessment describes the constraint satisfaction of the selected best-run designs.

### 4.7. Statistical Significance Analysis

Statistical analysis is conducted using the final fitness values from 30 independent runs of each algorithm. Wilcoxon rank-sum tests compare SSMO with each competing algorithm. The tested values are the penalized fitness values for the constrained problems and the objective values for the gear-train problem. Holm’s correction is applied to the 11 comparisons within each problem at a family-wise significance level of 0.05. For significant comparisons, the direction is determined from the pooled average ranks, with lower ranks indicating better performance.

Table 18 summarizes the outcomes as win/tie/loss counts, where a tie denotes no statistically significant difference. SSMO shows significant advantages over all 11 competing algorithms in four problems. In the welded-beam problem, it shows significant advantages over eight algorithms, while no statistically significant differences are observed against GWO, FATA, or EMWPA. These results support favorable relative performance under the adopted experimental settings.

## 5. Application of SSMO to Photovoltaic Model Parameter Identification

In renewable energy applications, photovoltaic (PV) systems serve as a key technology for the direct conversion of solar radiation into electricity. Accordingly, the accurate estimation of model parameters from measured current–voltage (I–V) characteristics is essential for establishing reliable PV models. The single diode model (SDM), double diode model (DDM), triple diode model (TDM), and PV module model (PVM) are commonly adopted in engineering-oriented PV modeling studies [50,51,52,53].

For the comparisons between SSMO and the 11 general-purpose algorithms, the population size and maximum number of iterations are fixed at 30 and 500, respectively. SSMO and the 11 general-purpose comparison algorithms perform the same number of objective-function evaluations per iteration. Each algorithm is run independently 30 times to make the results more reliable. The measured I–V data used in this study are obtained from the benchmark dataset originally reported by Easwarakhanthan et al. For the SDM, DDM, and TDM experiments, the R.T.C. France solar-cell dataset is used, comprising 26 measured voltage–current pairs recorded at 33 °C under an irradiance of 1000 W/m^2^ [54]. The Photowatt-PWP201 [55] device is a commercial polycrystalline PV module composed of 36 series-connected cells. Its I–V characteristics are measured at an irradiance of 1000 W/m^2^ and a module temperature of 45 °C. The dataset contains 25 measured voltage–current pairs, with the terminal voltage ranging from 0.1248 to 17.4885 V and the measured current ranging from 1.0315 to −0.3030 A. These measurements cover the nearly short-circuit region, the knee region, and the region slightly beyond the open-circuit point.

### 5.1. Single Diode Model (SDM)

**Figure 23:**
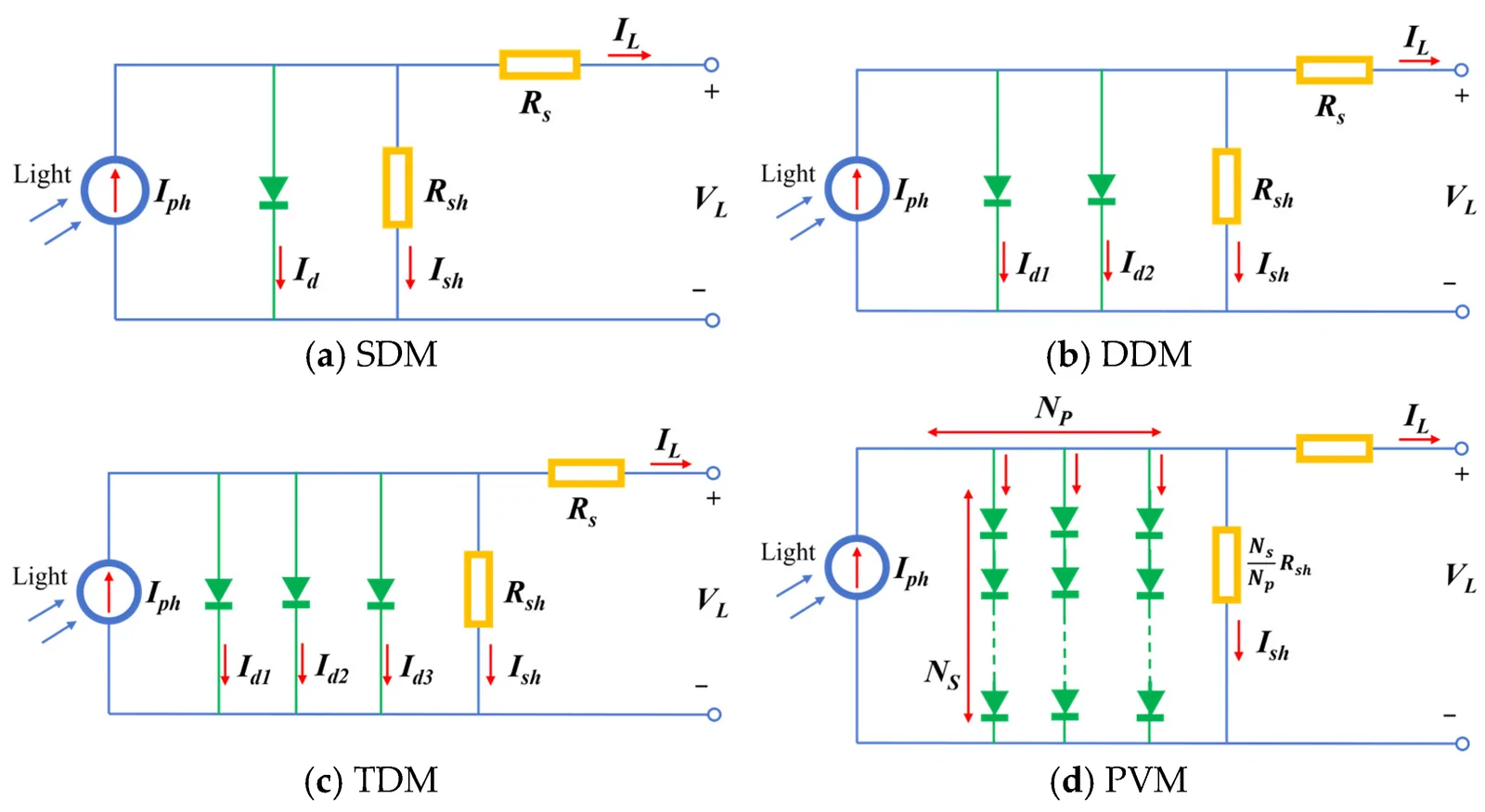
Schematic of the equivalent circuit models of PV systems.

The single diode model (SDM) represents one of the most fundamental equivalent-circuit formulations for PV systems and has been widely adopted because of its simple structure. The corresponding circuit configuration is shown in Figure 23a. Five unknown parameters are required to characterize this model. The output-current equation and the associated residual function are formulated in Equations (21) and (22), respectively. As expressed in Equation (23), the root mean square error (RMSE) is selected as the objective function to measure the discrepancy between the calculated and experimental I–V data.

(21) $$\left\{\begin{array}{l} I_{L}=I_{\mathrm{ph}}-I_{d}-I_{\mathrm{sh}}\\ I_{d}=I_{\mathrm{sd}}\left[\exp\left(\frac{q\left(V_{L}+R_{S}I_{L}\right)}{\mathrm{nkT}}\right)-1\right]\\ I_{\mathrm{sh}}=\frac{V_{L}+R_{S}I_{L}}{R_{\mathrm{sh}}} \end{array}\right.$$

(22) $$\left\{\begin{array}{l} f_{k}\left(V_{L},I_{L},X\right)=I_{\mathrm{ph}}-I_{\mathrm{sd}}\left[\exp\left(\frac{q\left(V_{L}+R_{S}I_{L}\right)}{\mathrm{nkT}}\right)-1\right]-\frac{V_{L}+R_{S}I_{L}}{R_{\mathrm{sh}}}-I_{L}\\ X=\{I_{\mathrm{ph}},I_{\mathrm{sd}},R_{s},R_{\mathrm{sh}},n\} \end{array}\right.$$

(23) $$\mathrm{RMSE}\left(X\right)=\sqrt{\frac{1}{N}\sum_{i=1}^{N}f^{2}\left(V_{L},I_{L},X\right)}$$

where *I_L_* and *V_L_* are the terminal current and voltage, respectively. The parameter *I_ph_* denotes the photocurrent, while *I_d_* and *I_sh_* correspond to the diode current and shunt current. In addition, *R_s_* and *R_sh_* represent the series and shunt resistances, *I_sd_* is the diode reverse saturation current, and *n* denotes the ideality factor. The constants *k*, *q*, and *T* indicate the Boltzmann constant, electron charge, and absolute temperature, respectively.

**Figure 24:**
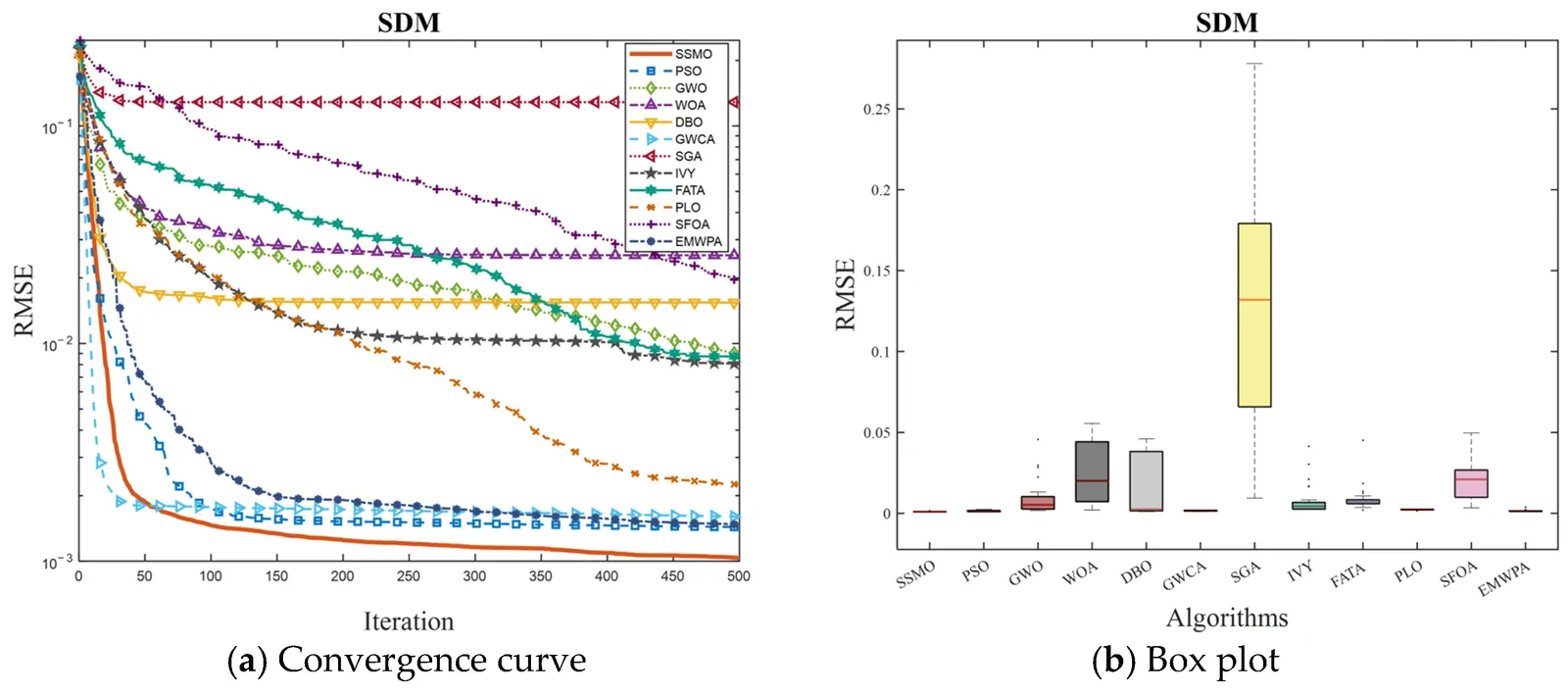
Convergence curves and box plots of SSMO for SDM parameter identification.

**Figure 25:**
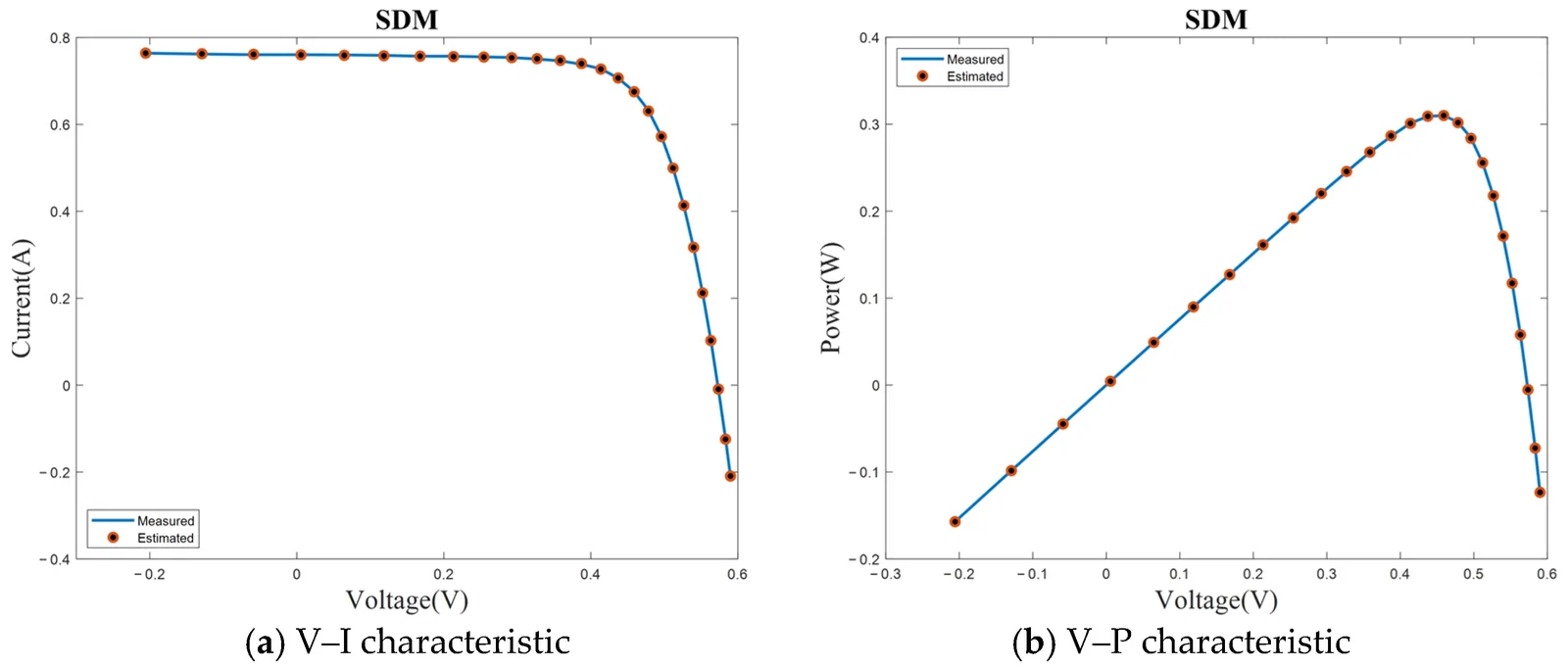
Comparisons between measured data and experimental data attained by SSMO for SDM.

The parameter-identification results obtained for the SDM are reported in Figure 24. The convergence profiles show that SSMO reaches the best final objective values with the most rapid search process among the compared algorithms, indicating advantages in both convergence efficiency and identification accuracy. The corresponding box plots also reveal a compact distribution of results, suggesting that SSMO exhibits strong run-to-run stability. Figure 25 compares the I–V characteristics generated by SSMO with the measured PV cell data, where the close overlap between the two curves verifies the high precision of the identified parameters.

Table 19 reports the statistical RMSE results for SDM parameter identification, including the maximum, minimum, mean, median, and standard deviation values obtained by SSMO and the competing methods. SSMO obtains the smallest RMSE values for all reported indicators, with an average RMSE of 1.0115 × 10^−3^, which is clearly lower than that of PSO 1.5365 × 10^−3^ and the other comparative algorithms. The standard deviation of 1.3936 × 10^−4^ further indicates that SSMO produces highly consistent results over independent runs. These observations demonstrate that SSMO can achieve accurate and robust parameter estimation for the SDM. The corresponding optimal parameter values are listed in Table A6 in Appendix A.

### 5.2. Double Diode Model (DDM)

The equivalent circuit of the double diode model (DDM) is depicted in Figure 23b. In contrast to the SDM, the DDM incorporates a second parallel diode branch, allowing the diffusion and recombination current components to be described separately. Consequently, seven parameters need to be determined in the DDM. The output current relation and residual expression are presented in Equations (24) and (25), respectively. The previously defined RMSE is again adopted as the objective function for parameter optimization.

(24) $$I_{L}=I_{\mathrm{ph}}-I_{\mathrm{sd}1}\left[\exp\left(\frac{q\left(V_{L}+R_{S}I_{L}\right)}{n_{1}\mathrm{kT}}\right)-1\right]-I_{\mathrm{sd}2}\left[\exp\left(\frac{q\left(V_{L}+R_{S}I_{L}\right)}{n_{2}\mathrm{kT}}\right)-1\right]-\frac{V_{L}+R_{S}I_{L}}{R_{\mathrm{sh}}}$$

(25) $$\left\{\begin{array}{l} f\left(V_{L},I_{L},X\right)=I_{\mathrm{ph}}-I_{\mathrm{sd}1}\left[\exp\left(\frac{q\left(V_{L}+R_{S}I_{L}\right)}{n_{1}\mathrm{kT}}\right)-1\right]-I_{\mathrm{sd}2}\left[\exp\left(\frac{q\left(V_{L}+R_{S}I_{L}\right)}{n_{2}\mathrm{kT}}\right)-1\right]\\ \;\;\;\;\;\;-\frac{V_{L}+R_{S}I_{L}}{R_{\mathrm{sh}}}-I_{L}\\ X=\left(I_{\mathrm{ph}},R_{s},R_{\mathrm{sh}},I_{\mathrm{sd}1},n_{1},I_{\mathrm{sd}2},n_{2}\right) \end{array}\right.$$

where *I_sd_*_1_ and *I_sd_*_2_ represent the saturation currents associated with the two diode branches, whereas *n*_1_ and *n*_2_ denote their corresponding ideality factors. The remaining symbols have the same meanings as those defined for the SDM.

**Figure 26:**
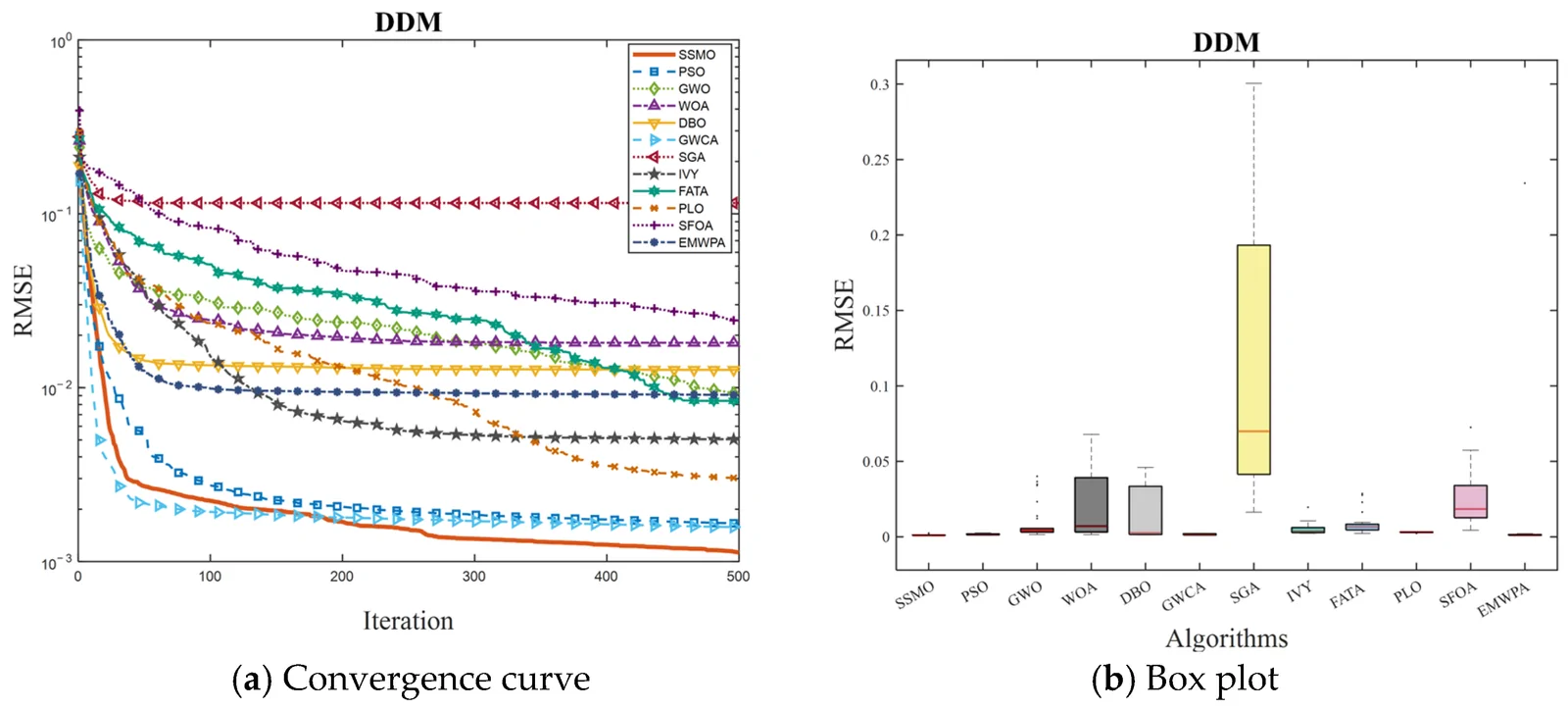
Convergence curves and box plots of SSMO for DDM parameter identification.

**Figure 27:**
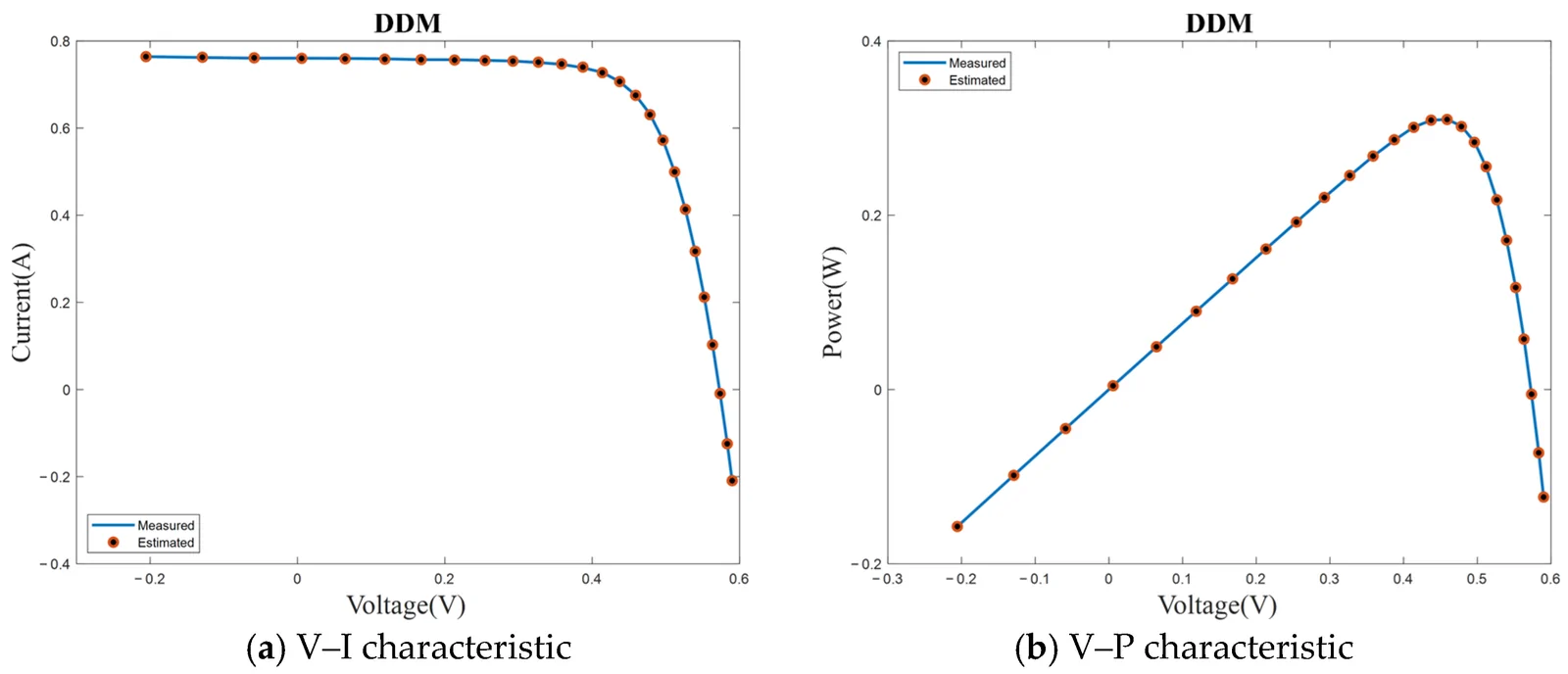
Comparisons between measured data and experimental data attained by SSMO for DDM.

The convergence behavior and fitting performance for DDM parameter identification are shown in Figure 26 and Figure 27. As observed from the convergence curves, SSMO approaches the optimum rapidly and yields the lowest final RMSE among all compared algorithms. The box plot results further demonstrate that its solutions remain stable across repeated independent runs. The fitting curves also show that the measured data can be accurately reproduced under the DDM framework, suggesting that SSMO remains reliable even when the model complexity increases.

Table 20 provides the RMSE-based statistical comparison for DDM parameter identification. SSMO consistently achieves the lowest error values for all statistical indicators, and its mean RMSE reaches 1.0092 × 10^−3^, which is much smaller than that of the closest competing method, EMWPA 1.5248 × 10^−3^. Its small standard deviation of 1.0654 × 10^−4^ further reflects the repeatability and stability of the proposed algorithm. These findings indicate that SSMO can effectively cope with the additional parameter complexity of the DDM while maintaining accurate and stable estimation performance. The identified optimal parameters are reported in Table A7 in Appendix A.

### 5.3. Triple Diode Model (TDM)

The equivalent circuit configuration of the triple diode model (TDM) is shown in Figure 23c. Compared with the DDM, the TDM further incorporates a third diode branch to describe additional recombination-related effects. As a result, nine parameters need to be estimated in this model. The output-current equation is formulated in Equation (26), the corresponding residual function is presented in Equation (27), and the RMSE is adopted as the optimization objective.

(26) $$I_{L}=I_{\mathrm{ph}}-I_{\mathrm{sd}1}\left[\exp\left(\frac{q\left(V_{L}+R_{s}I_{L}\right)}{n_{1}\mathrm{kT}}\right)-1\right]-I_{\mathrm{sd}2}\left[\exp\left(\frac{q\left(V_{L}+R_{s}I_{L}\right)}{n_{2}\mathrm{kT}}\right)-1\right]-I_{\mathrm{sd}3}\left[\exp\left(\frac{q\left(V_{L}+R_{s}I_{L}\right)}{n_{3}\mathrm{kT}}\right)-1\right]-\frac{V_{L}+R_{s}I_{L}}{R_{\mathrm{sh}}}$$

(27) $$\left\{\begin{array}{l} f\left(V_{L},I_{L},X\right)=I_{\mathrm{ph}}-I_{\mathrm{sd}1}\left[\exp\left(\frac{q\left(V_{L}+R_{s}I_{L}\right)}{n_{1}\mathrm{kT}}\right)-1\right]-I_{\mathrm{sd}2}\left[\exp\left(\frac{q\left(V_{L}+R_{s}I_{L}\right)}{n_{2}\mathrm{kT}}\right)-1\right]\\ \;\;\;\;\;\;-I_{\mathrm{sd}3}\left[\exp\left(\frac{q\left(V_{L}+R_{s}I_{L}\right)}{n_{3}\mathrm{kT}}\right)-1\right]-\frac{V_{L}+R_{s}I_{L}}{R_{\mathrm{sh}}}-I_{L}\\ X=\left(I_{\mathrm{ph}},R_{s},R_{\mathrm{sh}},I_{\mathrm{sd}1},n_{1},I_{\mathrm{sd}2},n_{2},I_{\mathrm{sd}3},n_{3}\right) \end{array}\right.$$

where *I_sd_*_3_ represents the saturation current of the third diode branch, and *n*_3_ denotes its ideality factor. The remaining variables are consistent with the definitions given for the SDM and DDM.

**Figure 28:**
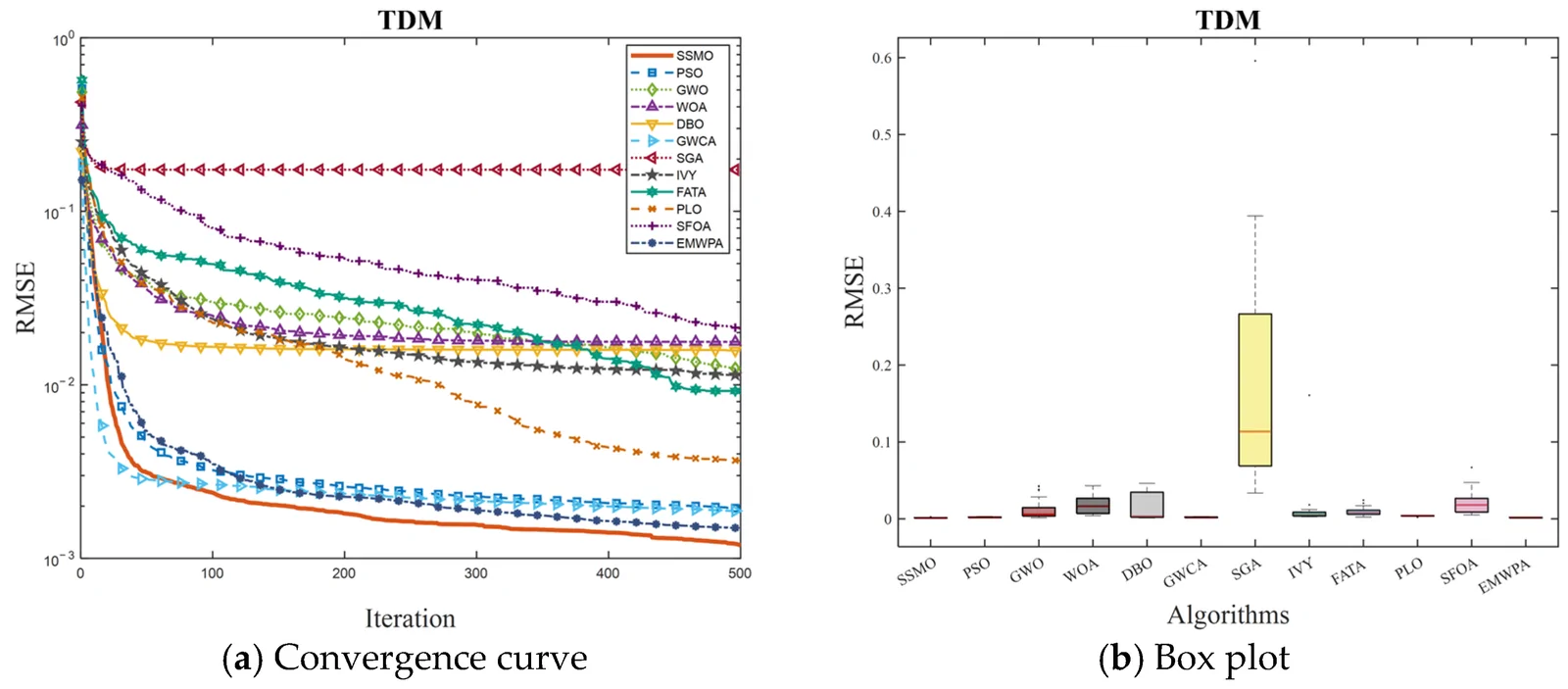
Convergence curves and box plots of SSMO for TDM parameter identification.

**Figure 29:**
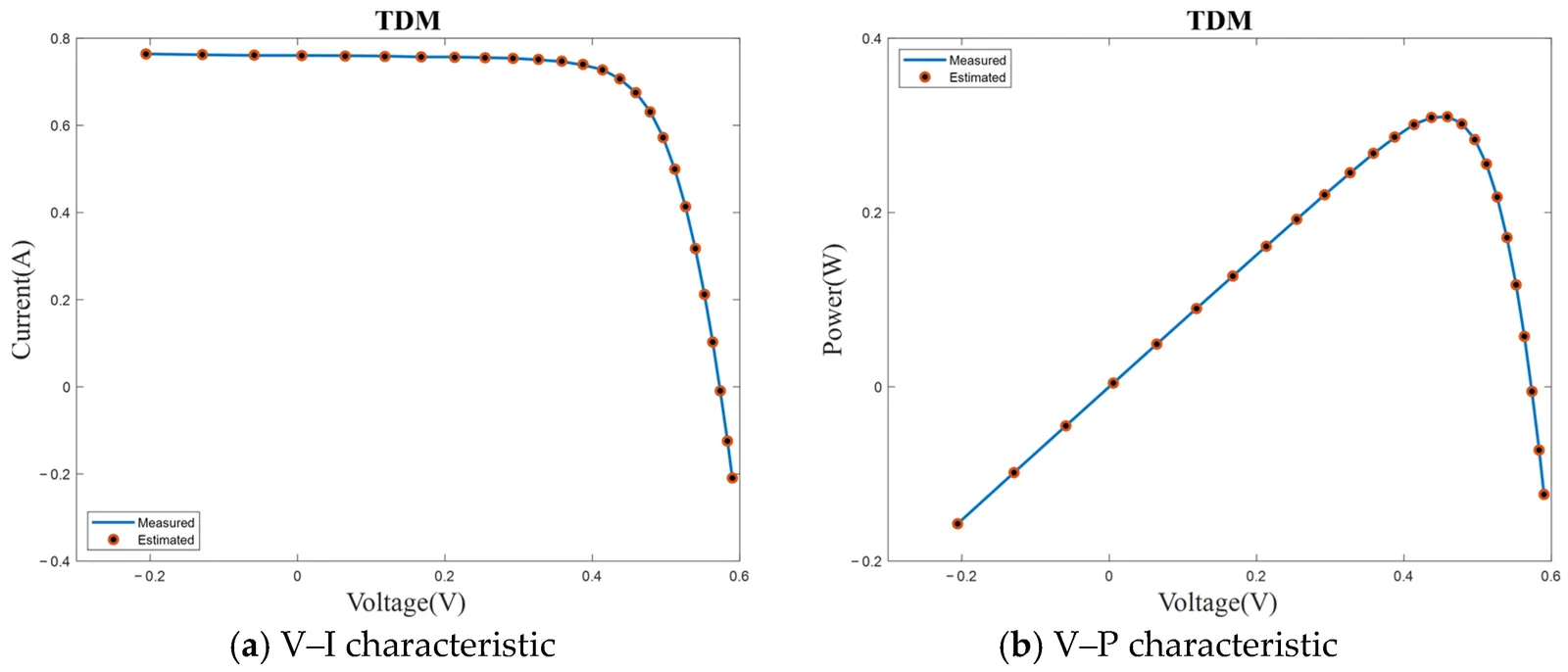
Comparisons between measured data and experimental data attained by SSMO for TDM.

The convergence characteristics and fitting results of SSMO for TDM parameter identification are presented in Figure 28 and Figure 29. Although the TDM introduces a more complex search space, SSMO still converges rapidly and obtains the lowest final RMSE among the compared algorithms. The box plot distributions further indicate that SSMO maintains stable performance over repeated independent runs. The fitted I–V curves are in close agreement with the measured PV data, confirming the accuracy of the identified TDM parameters.

Table 21 summarizes the RMSE-based statistical results for TDM parameter identification. Even under the increased complexity of the TDM, SSMO obtains the lowest mean RMSE of 1.1477 × 10^−3^ and a small standard deviation of 3.3102 × 10^−4^, which are lower than those of representative competitors such as PSO 2.9056 × 10^−3^ and WOA 2.5718 × 10^−2^. These results show that SSMO can identify TDM parameters with high accuracy while preserving good robustness. The corresponding optimal parameter values are listed in Table A8 in Appendix A.

### 5.4. PV Module Model (PVM)

The PV module model (PVM) extends the single-diode model to describe the electrical characteristics of a PV module, as illustrated in Figure 23d. Five parameters are estimated using a scaled parameterization with fixed coefficients *N_s_* = 3 and *N_p_* = 5. The current equation and residual function are given in Equations (28) and (29), respectively. The RMSE of the residuals is minimized during parameter estimation.

(28) $$I_{L}=N_{P}I_{\mathrm{ph}}-N_{P}I_{\mathrm{sd}}\left[\exp\left(\frac{q\left(V_{L}+I_{L}\frac{N_{S}}{N_{P}}R_{s}\right)}{N_{S}\mathrm{nkT}}\right)-1\right]-\frac{V_{L}+I_{L}\frac{N_{S}}{N_{P}}R_{s}}{\frac{N_{S}}{N_{P}}R_{\mathrm{sh}}}$$

(29) $$\left\{\begin{array}{l} f\left(V_{L},I_{L},X\right)=N_{P}I_{\mathrm{ph}}-N_{P}I_{\mathrm{sd}}\left[\exp\left(\frac{q\left(V_{L}N_{P}+N_{S}I_{L}R_{s}\right)}{N_{S}N_{P}\mathrm{nkT}}\right)-1\right]\\ \;\;\;\;\;\;-\frac{V_{L}N_{P}+I_{L}R_{s}N_{S}}{N_{S}R_{\mathrm{sh}}}-I_{L}\\ X=\left(I_{\mathrm{ph}},I_{\mathrm{sd}},R_{s},R_{\mathrm{sh}},n\right) \end{array}\right.$$

Following the numerical PVM formulation reported by Xiao et al. [55], the fixed scaling coefficients are set to *N_s_* = 3 and *N_p_* = 5. These coefficients define the parameterization used in the optimization process and are applied identically to all algorithms. They are numerical scaling factors rather than the physical numbers of series- and parallel-connected cells in the Photowatt-PWP201 module. The scaled parameters are related to their module-level equivalents as follows: *I_ph_*_,*m*_ = *N_p_I_ph_*, *I*_0,*m*_ = *N_p_I_SD_*, $R_{s,m}=\frac{N_{s}}{N_{p}}R_{s}$, $R_{\mathrm{sh},m}=\frac{N_{s}}{N_{p}}R_{\mathrm{sh}}$, *a_m_* = *N_s_n*. Substituting these relationships into the scaled PVM equation yields the corresponding aggregate module-level equation exactly. Therefore, the calculated currents, current residuals, and RMSE values remain unchanged.

**Figure 30:**
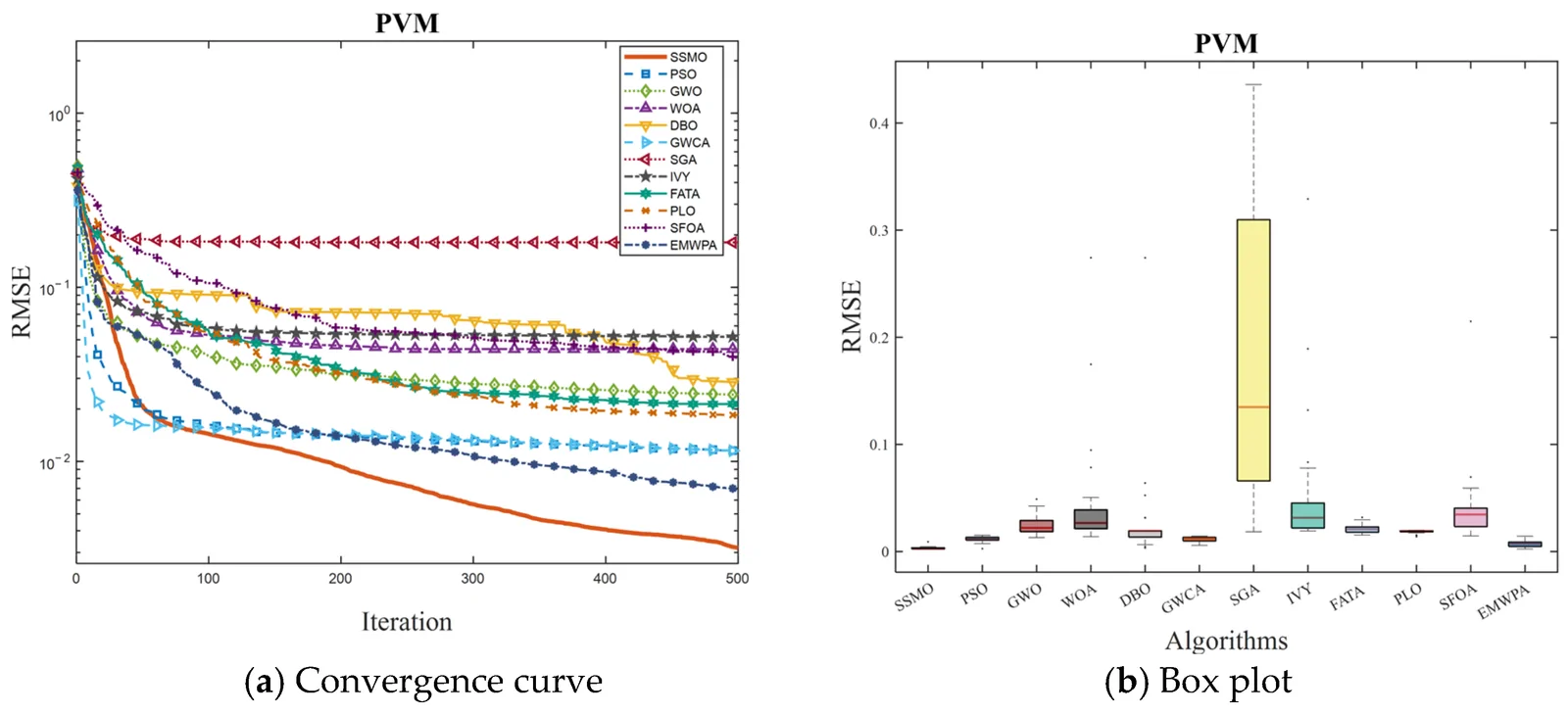
Convergence curves and box plots of SSMO for PVM parameter identification.

**Figure 31:**
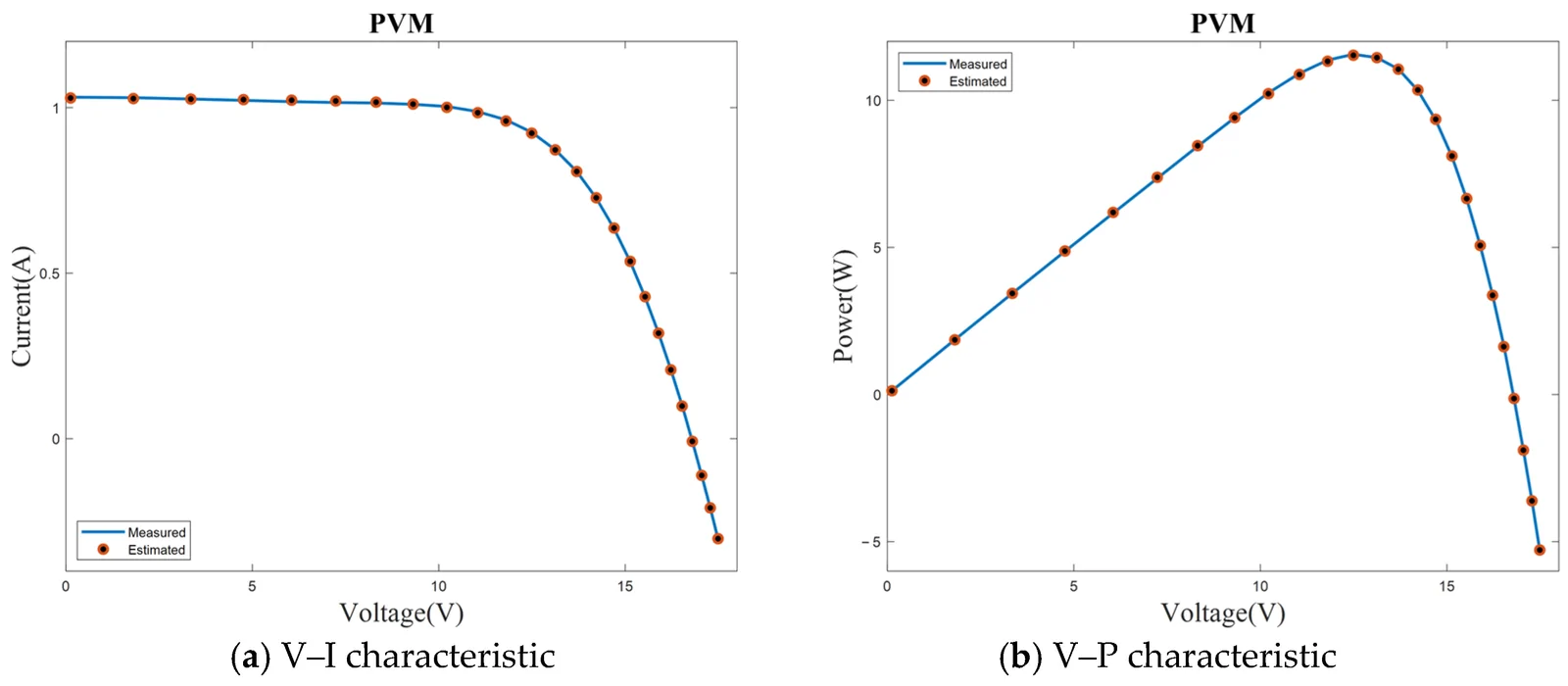
Comparisons between measured data and experimental data attained by SSMO for PVM.

The convergence curves and fitting performance obtained for the PVM are shown in Figure 30 and Figure 31. The convergence results indicate that SSMO reaches the lowest final RMSE with a fast search process, showing clear advantages in convergence efficiency and solution quality. The box plots show limited dispersion across independent runs, and the fitting curves further verify the accuracy of the module-level parameter estimation.

Table 22 reports the RMSE statistics obtained for PVM parameter identification. SSMO achieves the lowest mean RMSE of 3.4122 × 10^−3^, together with a standard deviation of 1.0193 × 10^−3^, indicating accurate and stable identification performance for the module-level model. Compared with representative algorithms such as PSO 1.2301 × 10^−2^ and WOA 3.6025 × 10^−2^, SSMO provides a more accurate solution for the nonlinear and strongly coupled five-parameter search problem. The identified optimal parameter values are given in Table A9 in Appendix A.

### 5.5. Extended Experiments on PV Methods

**Figure 32:**
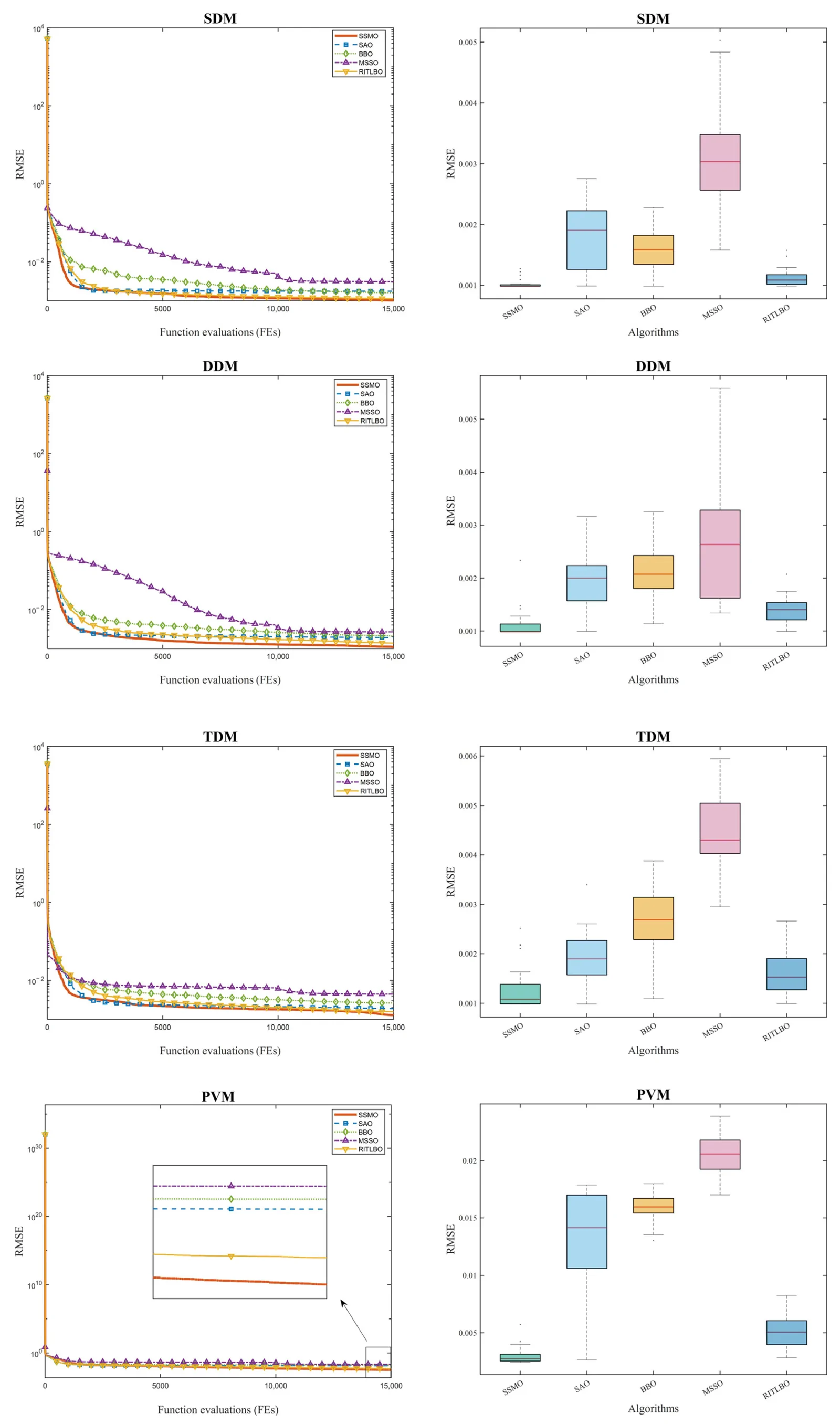
Convergence curves and box plots of five algorithms on four photovoltaic models.

To further examine the performance of SSMO in PV parameter identification, four recently reported optimization methods, namely SAO, RITLBO, MSSO, and BBO, are included for additional comparison. All five algorithms are independently executed 30 times with a population size of 30. Because RITLBO and MSSO do not necessarily require the same number of objective-function evaluations per iteration as the other algorithms, using only an identical iteration limit does not guarantee an equal computational budget. Therefore, a unified objective-function counter is employed for all algorithms, and each independent run terminates after exactly 15,000 actual function evaluations. The corresponding convergence behavior and statistical results are presented in Figure 32 and Table 23, respectively.

As illustrated in Figure 32, SSMO exhibits the most favorable overall convergence behavior across all four PV models. It rapidly reduces the RMSE and reaches a lower final error level than the other comparison algorithms. The box plots further show that the SSMO results are concentrated in the lower-RMSE regions for the SDM, DDM, TDM, and PVM cases. Although RITLBO produces slightly smaller variability for the DDM and TDM cases, its median and mean RMSE values remain higher than those obtained by SSMO.

The statistical results in Table 23 further confirm these observations. For the SDM, SSMO achieves the best values for all five statistical indicators, namely the maximum, minimum, mean, median, and standard deviation. For the DDM, SSMO obtains the lowest minimum, mean, and median RMSE values, whereas RITLBO produces slightly lower maximum RMSE and standard deviation values. For the TDM, SSMO ranks first in terms of the maximum, minimum, mean, and median RMSE, while RITLBO achieves a slightly smaller standard deviation. For the PVM, SSMO again achieves the best results for all five indicators. Notably, SSMO obtains the lowest mean and median RMSE values for every PV model, demonstrating its consistently high parameter-identification accuracy.

To assess whether the observed performance differences are statistically significant, Wilcoxon rank-sum tests are conducted between SSMO and each comparison algorithm using the final RMSE values obtained from 30 independent runs. Four pairwise comparisons are performed for each of the four PV models, resulting in 16 *p*-values, which are jointly adjusted using Holm’s procedure to control the family-wise error rate. The significance level is set to 0.05, and the Holm-adjusted results are reported in Table 24, where “+” indicates that SSMO achieves a significantly lower RMSE than the corresponding algorithm. All 16 adjusted *p*-values are below 0.05. The largest adjusted *p*-value is 6.203 × 10^−4^, obtained for the comparison between SSMO and RITLBO on the TDM, and remains well below the specified significance level. Together with the lower mean RMSE achieved by SSMO in all 16 comparisons, these results indicate that SSMO significantly outperforms SAO, BBO, MSSO, and RITLBO across the four PV parameter-identification models.

The benchmark and application experiments examine complementary aspects of SSMO. The CEC suites assess its search behavior across different function structures and dimensions, while the engineering problems examine constrained and discrete optimization settings. PV parameter identification involves a bounded nonlinear search in which interacting model parameters are estimated by minimizing the discrepancy between calculated and measured I–V data. Elite and memory guidance are intended to support solution refinement, while differential search and stochastic perturbations provide exploration of alternative parameter combinations. These search requirements motivate the application of SSMO to PV identification. The CEC results provide evidence of its general optimization capability, whereas the PV experiments directly assess its effectiveness on the specific parameter-estimation tasks considered here.

## 6. Conclusions and Future Work

This study presents the Sooty Shearwater Migration Optimizer (SSMO), a new metaheuristic algorithm motivated by the long-distance migration behavior of sooty shearwaters. In the proposed framework, the movement of individuals is regulated by four complementary mechanisms: elite guidance, wind-driven migration, molting memory guidance, and adaptive foraging. These mechanisms enhance information exchange, guide the search toward promising regions, and help maintain the balance between exploration and exploitation.

The optimization performance of SSMO is evaluated on two benchmark suites, namely CEC2017 under 30D, 50D, and 100D settings and CEC2022 under 10D and 20D settings. The experimental settings involve optimization tasks with different dimensional characteristics, covering both low and high dimensional cases. Compared with 11 representative optimization algorithms, SSMO shows overall competitive performance, with favorable results in best value, mean value, Friedman ranking, Wilcoxon test, and comprehensive statistical evaluation. The favorable results may partly reflect the combination of solution refinement through elite and memory guidance with exploration through differential search and stochastic perturbations. At higher CEC2017 dimensions, the reduced relative advantage may reflect the difficulty of maintaining sufficient search coverage with a fixed population size.

The practical applicability of SSMO is further assessed on five engineering design problems. In these cases, SSMO delivers the lowest average objective values among the compared methods. In addition, four PV parameter identification tasks are employed to assess its effectiveness in practical parameter estimation. Among the 12 general-purpose algorithms considered, SSMO obtains the lowest error values for the adopted evaluation metrics in these tasks. Additional comparisons with four specialized PV parameter identification algorithms further show that SSMO achieves competitive or better results for most evaluation metrics. These results indicate that SSMO has good robustness, generalization ability, and application potential for different complex optimization tasks.

Several limitations remain in the present study. The parameter configuration of SSMO was selected empirically on CEC2017 at 30D. The one-factor-at-a-time sensitivity analysis describes the effects of individual parameters but does not assess their interactions or establish a jointly optimal configuration. Although runtime measurements and comparisons with adaptive DE variants have been added, the computational assessment is limited to the tested platform and problem settings, and the algorithm comparisons cover a selected set of methods.

Future work will focus on joint parameter analysis and automatic parameter configuration, with separate problems used for parameter selection and evaluation. Alternative constraint-handling methods will be compared to examine how the penalty formulation affects feasibility and relative performance. Further computational studies will assess runtime across different platforms, dimensions, and objective-evaluation costs, alongside comparisons with additional strong continuous optimizers. These investigations will help identify the conditions under which SSMO is most effective and where further improvements are needed.

## Figures and Tables

**Table 1:** Representative algorithms relevant to the search mechanisms and PV parameter identification.

Category	Algorithm	Main Search Feature	Relevance to This Study
Differential search	Differential evolution (DE)	Differential-vector mutation and crossover	Classical differential-search method
	Self-adaptive differential evolution (SaDE)	Adaptive selection of trial-vector strategies and control parameters	Representative adaptive DE variant
	L-SHADE with ensemble sinusoidal parameter adaptation (L-SHADE-EpSin)	Success-history learning and ensemble sinusoidal parameter adaptation	High-performing adaptive DE variant
	L-SHADE with semi-parameter adaptation hybridized with CMA-ES (L-SHADE-SPACMA)	Semi-parameter adaptation combined with covariance-based search	Hybrid adaptive DE variant
	Ring sub-population architecture-based differential evolution (RSDE)	Ring-connected subpopulations and conditional information exchange	Recent population-structured DE variant
Population-guided search	Particle swarm optimization (PSO)	Individual-best and global-best guidance	Representative memory- and group-guided method
	Grey wolf optimizer (GWO)	Multi-leader guidance using the three leading solutions	Representative elite-guided method
	Whale optimization algorithm (WOA)	Random exploration, encircling, and spiral search	Representative phase-switching search method
	Dung beetle optimizer (DBO)	Population division and role-specific behavioral updates	Representative multi-behavior search method
PV parameter identification	Snow ablation optimizer (SAO)	Population division and dynamically controlled search	Recent method applied to PV parameter identification
	Rank-improved teaching–learning-based optimization (RITLBO)	Ranking-based teaching and learning strategies	Specialized PV comparison method
	Multi-strategy synergistic learning snake optimizer (MSSO)	Multi-strategy synergistic learning and local refinement	Specialized PV comparison method
	Beaver behavior optimizer (BBO)	Multiple behavior-based exploration and exploitation strategies	Specialized PV comparison method

**Table 2:** Definitions and settings of the parameters and stochastic variables in SSMO.

Parameter	Meaning	Range or Distribution	Adopted Value or Update Rule	Status	Equation
*N*	Population size	Positive integer	30	Fixed	Equation (1)
*D*	Dimension of the search space	Positive integer	Defined by the optimization problem	Fixed during each run	Equations (1) and (2)
*T*_max_	Maximum number of iterations	Positive integer	Defined by the experimental stopping condition	Fixed	Equation (14)
*lb*, *ub*	Lower and upper bounds of the decision variables	Problem-dependent *D*-dimensional vectors	Defined by each optimization problem	Fixed	Equations (2), (17) and (19)
*ω_k_*	Rank-based weight of the *k*-th elite individual	0 < *ω_k_* < 1	exp(−*k*/*L*)	Deterministic and rank-dependent	Equation (6)
*r_i_*, *γ_i_*	Uniform random variable and stochastic activity factor for elite guidance	*r_i_* ∼ *U*(0, 1); *γ_i_* ∈ [0.6, 1.4]	*γ_i_* = 3/5 + (4/5)*r_i_*	Dynamically sampled for each individual	Equation (7)
*z_i_*, *η_i_*	Standard normal variable and wind intensity coefficient	*z_i_* ∼ N(0, 1); *η_i_* ∼ N(0.5, 0.1^2^)	*η_i_* = 0.5 + *σ_η_z_i_*	Dynamically sampled for each individual	Equation (9)
*σ_η_*	Standard deviation of the wind intensity coefficient	Positive scalar	0.1	Fixed	Equation (9)
*r*_4_, *ψ_i_*	Uniform random variable and molting memory attraction coefficient	*r*_4_ ∼ *U*(0, 1); *ψ_i_* ∈ [0.8, 1.4]	*ψ_i_* = 4/5 + (3/5)*r*_4_	Dynamically sampled for each individual	Equation (12)
*μ*	Balance factor between elite guidance and molting memory guidance	[0, 1]	$1-{\left(t/T_{\max}\right)}^{2}$	Dynamically and deterministically updated	Equation (14)
*q*	Foraging-parameter scale	Positive scalar	0.8	Fixed	Equation (15)
*p_i_*	Probability of perturbing each dimension	[0, 1]	0.35*q* = 0.28 for poorer individuals; 0.12*q* = 0.096 for better individuals	Dynamically selected according to fitness	Equation (15)
*c_i_*	Foraging perturbation scale	Positive scalar	0.03*q* = 0.024 for poorer individuals; 0.005*q* = 0.004 for better individuals	Dynamically selected according to fitness	Equation (15)
*r*_5,*i*,*j*_	Random variable used to construct the dimensional mask	*U*(0, 1)	Independently sampled for each individual and dimension	Dynamically sampled	Equation (16)
*ξ_i_*	Gaussian perturbation vector	N(0, *I_D_*)	Independently generated for each individual	Dynamically sampled	Equation (17)

**Table 3:** Comparison of SSMO operators with related optimization mechanisms.

SSMO Operator	Mathematical Principle	Related Existing Mechanism	Difference in SSMO	Specific Contribution Within SSMO
Elite guidance	Rank-weighted aggregation of normalized direction vectors	Best-guided and elite-guided search	SSMO aggregates directions from *L* current elite individuals using exponentially decreasing rank weights and the stochastic factor *γ_i_*, rather than relying only on a single best solution.	It retains several promising directions and reduces excessive dependence on a single leader.
Wind-driven migration	Scaled difference between population members	Differential-vector principle in DE	One endpoint is selected from the first *M* ranked individuals and the other from the entire population. The difference is scaled by *η_i_* ∼ N(0.5, 0.1^2^) and is used without the conventional DE mutation–crossover sequence.	It introduces a rank-biased differential displacement while retaining information from individuals with different fitness levels.
Molting memory guidance	Displacement toward the global best-so-far solution	Global-best guidance in PSO and memory-based methods	SSMO does not use a velocity vector, inertia weight, or personal-best archive. The global best-so-far direction is directly weighted by 1 − *μ* in the position update.	It progressively strengthens best-so-far refinement during the later search stage.
Adaptive foraging	Fitness-dependent sparse Gaussian perturbation	Adaptive mutation and stochastic local search	The population median defines two perturbation states, and a Bernoulli dimensional mask controls which variables are modified. The expected coordinate-wise perturbation variance ratio between the two states is 105.	It applies broader modifications to poorer solutions and smaller adjustments to better solutions.

**Table 4:** Runtime comparison in selected 30-dimensional CEC2017 functions.

Algorithm	F1	F10	F20	F30	Average
SSMO	1.0544 ± 0.0092	1.4372 ± 0.0068	1.5088 ± 0.0099	2.8516 ± 0.0118	1.713
DE	1.5725 ± 0.0096	1.9525 ± 0.0072	2.0210 ± 0.0093	3.3706 ± 0.0228	2.2291
PSO	0.5237 ± 0.0191	0.8377 ± 0.0311	0.9190 ± 0.0076	2.2815 ± 0.0455	1.1405
GWO	1.0826 ± 0.0119	1.4500 ± 0.0248	1.5147 ± 0.0281	2.8432 ± 0.0282	1.7226

**Table 5:** Parameter setting of the comparison algorithms.

Algorithm	Year	Parameter	Value
PSO	1995	Inertia factor *ω* *c*_1_, *c*_2_	0.9 2, 2
GWO	2014	*α* *r*_1_, *r*_2_	[0, 2] [0, 1], [0, 1]
WOA	2016	*α*, *b*	[0, 2], 2
DBO	2023	*k*,*λ*, *b*,*S*	0.1, 0.1, 0.3, 0.5
GWCA	2023	*P*, *Q*, *T*, *m* *C_max_*, *C_min_*	9, 6, 8.3, 3 exp(3), exp(2)
SGA	2024	*θ*, *b* *d*, *e*	[0, 2*π*], [−2, 2] [−1.5, 1.5], [−1, 1]
IVY	2024	*β*	$\frac{2+\mathrm{rand}}{2}$
FATA	2024	*α* *Para*_1_, *Para*_2_	[0, 1] [−2, 2], [−150, 150]
PLO	2024	*m*, *a*	100, [1, 1.5]
SFOA	2025	*C*	0.8
EMWPA	2025	*E_max_*, *B_max_* *ω*, *λ*, *ϕ*	3, 1 4, 4000, 3.14

**Table 6:** Wilcoxon rank-sum test in CEC2017.

SSMO vs.	CEC2017(Dim = 30)	CEC2017(Dim = 50)	CEC2017(Dim = 100)
PSO	26/2/1	23/5/1	20/8/1
GWO	28/1/0	25/4/0	21/8/0
WOA	29/0/0	29/0/0	29/0/0
DBO	28/0/1	28/1/0	29/0/0
GWCA	24/3/2	24/2/3	15/11/3
SGA	29/0/0	29/0/0	29/0/0
IVY	25/2/2	25/3/1	15/11/3
FATA	28/1/0	28/0/1	28/0/1
PLO	18/5/6	13/8/8	16/6/7
SFOA	29/0/0	29/0/0	29/0/0
EMWPA	24/4/1	24/3/2	20/8/1
**Overall**	288/18/13	277/26/16	251/52/16

**Table 7:** Wilcoxon rank-sum test in CEC 2022.

SSMO vs.	CEC2022(Dim = 10)	CEC2022(Dim = 20)
PSO	8/1/3	10/1/1
GWO	12/0/0	12/0/0
WOA	12/0/0	11/1/0
DBO	10/1/1	10/1/1
GWCA	10/1/1	8/1/3
SGA	12/0/0	11/1/0
IVY	9/2/1	8/2/2
FATA	12/0/0	12/0/0
PLO	10/2/0	7/3/2
SFOA	12/0/0	12/0/0
EMWPA	10/1/1	8/2/2
**Overall**	117/8/7	109/12/11

**Table 8:** Results of SSMO and four DE variants in 30-dimensional CEC2017.

Function	F1				F3			
Metric	Mean	Std	Best	Rank	Mean	Std	Best	Rank
SSMO	7.82 × 10^4^	2.48 × 10^4^	4.56 × 10^4^	5	3.02 × 10^2^	6.15 × 10^−1^	3.01 × 10^2^	2
RSDE	1.00 × 10^2^	4.82 × 10^−7^	1.00 × 10^2^	3	3.00 × 10^2^	3.37 × 10^−12^	3.00 × 10^2^	1
L-SHADE-SPACMA	1.00 × 10^2^	1.56 × 10^−14^	1.00 × 10^2^	1	3.11 × 10^4^	3.80 × 10^4^	3.00 × 10^2^	5
L-SHADE-EpSin	1.00 × 10^2^	3.32 × 10^−10^	1.00 × 10^2^	2	2.34 × 10^4^	3.67 × 10^4^	3.00 × 10^2^	4
SaDE	8.58 × 10^2^	2.48 × 10^3^	1.00 × 10^2^	4	4.55 × 10^3^	3.18 × 10^3^	3.40 × 10^2^	3
Function	F4				F5			
Metric	Mean	Std	Best	Rank	Mean	Std	Best	Rank
SSMO	4.36 × 10^2^	3.00 × 10^1^	4.00 × 10^2^	3	5.69 × 10^2^	1.78 × 10^1^	5.40 × 10^2^	3
RSDE	4.29 × 10^2^	2.83 × 10^1^	4.00 × 10^2^	2	5.77 × 10^2^	2.04 × 10^1^	5.49 × 10^2^	5
L-SHADE-SPACMA	4.25 × 10^2^	2.87 × 10^1^	4.00 × 10^2^	1	5.36 × 10^2^	1.03 × 10^1^	5.20 × 10^2^	1
L-SHADE-EpSin	4.54 × 10^2^	2.96 × 10^1^	4.00 × 10^2^	4	5.39 × 10^2^	9.11 × 10^0^	5.23 × 10^2^	2
SaDE	4.65 × 10^2^	3.73 × 10^1^	4.00 × 10^2^	5	5.72 × 10^2^	3.45 × 10^1^	5.27 × 10^2^	4
Function	F6				F7			
Metric	Mean	Std	Best	Rank	Mean	Std	Best	Rank
SSMO	6.01 × 10^2^	6.98 × 10^−1^	6.00 × 10^2^	3	7.78 × 10^2^	1.18 × 10^1^	7.58 × 10^2^	1
RSDE	6.01 × 10^2^	9.63 × 10^−1^	6.00 × 10^2^	1	8.50 × 10^2^	3.94 × 10^1^	7.76 × 10^2^	5
L-SHADE-SPACMA	6.01 × 10^2^	8.24 × 10^−1^	6.00 × 10^2^	2	7.81 × 10^2^	2.03 × 10^1^	7.56 × 10^2^	3
L-SHADE-EpSin	6.02 × 10^2^	1.80 × 10^0^	6.00 × 10^2^	4	7.79 × 10^2^	1.42 × 10^1^	7.58 × 10^2^	2
SaDE	6.04 × 10^2^	3.18 × 10^0^	6.01 × 10^2^	5	8.36 × 10^2^	4.26 × 10^1^	7.68 × 10^2^	4
Function	F8				F9			
Metric	Mean	Std	Best	Rank	Mean	Std	Best	Rank
SSMO	8.61 × 10^2^	1.35 × 10^1^	8.41 × 10^2^	3	9.01 × 10^2^	4.73 × 10^−1^	9.00 × 10^2^	1
RSDE	8.69 × 10^2^	1.78 × 10^1^	8.42 × 10^2^	5	1.38 × 10^3^	3.64 × 10^2^	9.57 × 10^2^	5
L-SHADE-SPACMA	8.36 × 10^2^	8.57 × 10^0^	8.17 × 10^2^	1	1.02 × 10^3^	9.39 × 10^1^	9.14 × 10^2^	2
L-SHADE-EpSin	8.38 × 10^2^	9.72 × 10^0^	8.22 × 10^2^	2	1.06 × 10^3^	1.27 × 10^2^	9.29 × 10^2^	3
SaDE	8.64 × 10^2^	3.15 × 10^1^	8.31 × 10^2^	4	1.11 × 10^3^	2.04 × 10^2^	9.07 × 10^2^	4
Function	F10				F11			
Metric	Mean	Std	Best	Rank	Mean	Std	Best	Rank
SSMO	3.92 × 10^3^	6.99 × 10^2^	2.66 × 10^3^	4	1.15 × 10^3^	2.56 × 10^1^	1.11 × 10^3^	1
RSDE	3.48 × 10^3^	5.67 × 10^2^	2.51 × 10^3^	3	1.24 × 10^3^	6.82 × 10^1^	1.13 × 10^3^	5
L-SHADE-SPACMA	2.90 × 10^3^	2.73 × 10^2^	2.30 × 10^3^	1	1.24 × 10^3^	5.16 × 10^1^	1.15 × 10^3^	4
L-SHADE-EpSin	2.99 × 10^3^	2.88 × 10^2^	2.39 × 10^3^	2	1.23 × 10^3^	4.98 × 10^1^	1.14 × 10^3^	3
SaDE	7.86 × 10^3^	3.45 × 10^2^	7.19 × 10^3^	5	1.19 × 10^3^	5.43 × 10^1^	1.12 × 10^3^	2
Function	F12				F13			
Metric	Mean	Std	Best	Rank	Mean	Std	Best	Rank
SSMO	4.22 × 10^4^	2.49 × 10^4^	9.16 × 10^3^	5	1.49 × 10^3^	1.55 × 10^2^	1.36 × 10^3^	1
RSDE	1.40 × 10^4^	9.58 × 10^3^	3.34 × 10^3^	3	6.76 × 10^3^	9.37 × 10^3^	1.44 × 10^3^	4
L-SHADE-SPACMA	2.61 × 10^3^	3.94 × 10^2^	2.04 × 10^3^	1	3.66 × 10^3^	1.22 × 10^3^	2.09 × 10^3^	2
L-SHADE-EpSin	2.20 × 10^4^	1.70 × 10^4^	2.56 × 10^3^	4	3.81 × 10^3^	1.16 × 10^3^	1.79 × 10^3^	3
SaDE	1.11 × 10^4^	1.13 × 10^4^	2.46 × 10^3^	2	1.12 × 10^4^	1.37 × 10^4^	1.40 × 10^3^	5
Function	F14				F15			
Metric	Mean	Std	Best	Rank	Mean	Std	Best	Rank
SSMO	1.45 × 10^3^	1.33 × 10^1^	1.42 × 10^3^	1	1.53 × 10^3^	2.08 × 10^1^	1.51 × 10^3^	1
RSDE	1.62 × 10^3^	9.48 × 10^1^	1.47 × 10^3^	5	1.75 × 10^3^	2.01 × 10^2^	1.55 × 10^3^	2
L-SHADE-SPACMA	1.59 × 10^3^	6.66 × 10^1^	1.50 × 10^3^	3	1.85 × 10^3^	1.74 × 10^2^	1.59 × 10^3^	4
L-SHADE-EpSin	1.60 × 10^3^	6.32 × 10^1^	1.51 × 10^3^	4	1.83 × 10^3^	1.46 × 10^2^	1.55 × 10^3^	3
SaDE	1.46 × 10^3^	4.99 × 10^1^	1.42 × 10^3^	2	1.97 × 10^3^	2.08 × 10^3^	1.51 × 10^3^	5
Function	F16				F17			
Metric	Mean	Std	Best	Rank	Mean	Std	Best	Rank
SSMO	2.12 × 10^3^	2.18 × 10^2^	1.82 × 10^3^	3	1.85 × 10^3^	7.22 × 10^1^	1.76 × 10^3^	2
RSDE	2.19 × 10^3^	2.19 × 10^2^	1.74 × 10^3^	4	1.92 × 10^3^	1.19 × 10^2^	1.76 × 10^3^	5
L-SHADE-SPACMA	2.07 × 10^3^	2.14 × 10^2^	1.62 × 10^3^	2	1.86 × 10^3^	8.49 × 10^1^	1.73 × 10^3^	3
L-SHADE-EpSin	2.00 × 10^3^	1.95 × 10^2^	1.62 × 10^3^	1	1.86 × 10^3^	9.14 × 10^1^	1.75 × 10^3^	4
SaDE	2.26 × 10^3^	4.88 × 10^2^	1.60 × 10^3^	5	1.85 × 10^3^	8.53 × 10^1^	1.73 × 10^3^	1
Function	F18				F19			
Metric	Mean	Std	Best	Rank	Mean	Std	Best	Rank
SSMO	2.06 × 10^3^	1.56 × 10^2^	1.87 × 10^3^	2	1.92 × 10^3^	7.06 × 10^0^	1.91 × 10^3^	1
RSDE	7.64 × 10^3^	7.04 × 10^3^	2.01 × 10^3^	5	2.02 × 10^3^	6.25 × 10^1^	1.93 × 10^3^	2
L-SHADE-SPACMA	1.99 × 10^3^	8.04 × 10^1^	1.84 × 10^3^	1	2.12 × 10^3^	1.06 × 10^2^	1.99 × 10^3^	4
L-SHADE-EpSin	3.29 × 10^3^	3.50 × 10^3^	1.92 × 10^3^	4	2.06 × 10^3^	6.20 × 10^1^	1.97 × 10^3^	3
SaDE	2.20 × 10^3^	4.65 × 10^2^	1.85 × 10^3^	3	2.86 × 10^3^	3.48 × 10^3^	1.91 × 10^3^	5
Function	F20				F21			
Metric	Mean	Std	Best	Rank	Mean	Std	Best	Rank
SSMO	2.16 × 10^3^	7.26 × 10^1^	2.04 × 10^3^	2	2.37 × 10^3^	1.41 × 10^1^	2.35 × 10^3^	4
RSDE	2.22 × 10^3^	1.06 × 10^2^	2.06 × 10^3^	5	2.38 × 10^3^	1.23 × 10^1^	2.35 × 10^3^	5
L-SHADE-SPACMA	2.16 × 10^3^	8.86 × 10^1^	2.03 × 10^3^	3	2.34 × 10^3^	7.60 × 10^0^	2.32 × 10^3^	1
L-SHADE-EpSin	2.16 × 10^3^	8.33 × 10^1^	2.03 × 10^3^	4	2.34 × 10^3^	1.18 × 10^1^	2.33 × 10^3^	2
SaDE	2.10 × 10^3^	1.38 × 10^2^	2.00 × 10^3^	1	2.36 × 10^3^	2.48 × 10^1^	2.33 × 10^3^	3
Function	F22				F23			
Metric	Mean	Std	Best	Rank	Mean	Std	Best	Rank
SSMO	2.59 × 10^3^	1.10 × 10^3^	2.30 × 10^3^	2	2.69 × 10^3^	9.55 × 10^1^	2.42 × 10^3^	1
RSDE	2.66 × 10^3^	9.55 × 10^2^	2.30 × 10^3^	3	2.76 × 10^3^	3.84 × 10^1^	2.71 × 10^3^	5
L-SHADE-SPACMA	2.30 × 10^3^	1.74 × 10^0^	2.30 × 10^3^	1	2.69 × 10^3^	1.03 × 10^1^	2.67 × 10^3^	2
L-SHADE-EpSin	2.75 × 10^3^	9.14 × 10^2^	2.30 × 10^3^	4	2.70 × 10^3^	1.78 × 10^1^	2.66 × 10^3^	3
SaDE	4.64 × 10^3^	3.20 × 10^3^	2.30 × 10^3^	5	2.73 × 10^3^	3.53 × 10^1^	2.68 × 10^3^	4
Function	F24				F25			
Metric	Mean	Std	Best	Rank	Mean	Std	Best	Rank
SSMO	2.87 × 10^3^	2.05 × 10^1^	2.84 × 10^3^	2	2.89 × 10^3^	2.69 × 10^0^	2.89 × 10^3^	2
RSDE	2.94 × 10^3^	5.43 × 10^1^	2.86 × 10^3^	5	2.88 × 10^3^	1.62 × 10^1^	2.88 × 10^3^	1
L-SHADE-SPACMA	2.88 × 10^3^	2.72 × 10^1^	2.85 × 10^3^	3	2.89 × 10^3^	2.16 × 10^0^	2.88 × 10^3^	3
L-SHADE-EpSin	2.87 × 10^3^	2.06 × 10^1^	2.84 × 10^3^	1	2.90 × 10^3^	1.49 × 10^1^	2.89 × 10^3^	4
SaDE	2.90 × 10^3^	3.04 × 10^1^	2.85 × 10^3^	4	2.90 × 10^3^	2.07 × 10^1^	2.88 × 10^3^	5
Function	F26				F27			
Metric	Mean	Std	Best	Rank	Mean	Std	Best	Rank
SSMO	3.98 × 10^3^	6.22 × 10^2^	2.82 × 10^3^	1	3.21 × 10^3^	1.24 × 10^1^	3.19 × 10^3^	2
RSDE	4.63 × 10^3^	1.14 × 10^3^	2.80 × 10^3^	5	3.17 × 10^3^	1.29 × 10^1^	3.15 × 10^3^	1
L-SHADE-SPACMA	4.01 × 10^3^	5.16 × 10^2^	2.80 × 10^3^	2	3.23 × 10^3^	1.78 × 10^1^	3.20 × 10^3^	4
L-SHADE-EpSin	4.18 × 10^3^	3.28 × 10^2^	2.90 × 10^3^	3	3.24 × 10^3^	1.94 × 10^1^	3.21 × 10^3^	5
SaDE	4.53 × 10^3^	4.03 × 10^2^	2.90 × 10^3^	4	3.23 × 10^3^	2.73 × 10^1^	3.19 × 10^3^	3
Function	F28				F29			
Metric	Mean	Std	Best	Rank	Mean	Std	Best	Rank
SSMO	3.14 × 10^3^	6.04 × 10^1^	3.10 × 10^3^	1	3.51 × 10^3^	7.62 × 10^1^	3.34 × 10^3^	1
RSDE	3.16 × 10^3^	6.25 × 10^1^	3.10 × 10^3^	2	3.54 × 10^3^	1.70 × 10^2^	3.19 × 10^3^	5
L-SHADE-SPACMA	3.17 × 10^3^	6.16 × 10^1^	3.10 × 10^3^	3	3.52 × 10^3^	1.25 × 10^2^	3.33 × 10^3^	3
L-SHADE-EpSin	3.17 × 10^3^	5.91 × 10^1^	3.10 × 10^3^	4	3.54 × 10^3^	8.36 × 10^1^	3.35 × 10^3^	4
SaDE	3.18 × 10^3^	6.65 × 10^1^	3.10 × 10^3^	5	3.51 × 10^3^	1.24 × 10^2^	3.34 × 10^3^	2
Function	F30							
Metric	Mean	Std	Best	Rank				
SSMO	5.86 × 10^3^	6.02 × 10^2^	5.09 × 10^3^	1				
RSDE	6.64 × 10^3^	3.29 × 10^3^	3.41 × 10^3^	4				
L-SHADE-SPACMA	6.39 × 10^3^	2.86 × 10^3^	4.95 × 10^3^	2				
L-SHADE-EpSin	7.75 × 10^3^	3.95 × 10^3^	5.06 × 10^3^	5				
SaDE	6.43 × 10^3^	2.54 × 10^3^	5.02 × 10^3^	3				

**Table 9:** Results of SSMO and four DE variants in 10-dimensional CEC2022.

Function	F1				F2			
Metric	Mean	Std	Best	Rank	Mean	Std	Best	Rank
SSMO	3.00 × 10^2^	1.17 × 10^−4^	3.00 × 10^2^	3	4.03 × 10^2^	2.30 × 10^0^	4.00 × 10^2^	2
RSDE	3.00 × 10^2^	0.00 × 10^0^	3.00 × 10^2^	1.5	4.01 × 10^2^	1.86 × 10^0^	4.00 × 10^2^	1
L-SHADE-SPACMA	4.56 × 10^2^	4.39 × 10^2^	3.00 × 10^2^	5	4.04 × 10^2^	3.62 × 10^0^	4.00 × 10^2^	4
L-SHADE-EpSin	3.30 × 10^2^	1.02 × 10^2^	3.00 × 10^2^	4	4.07 × 10^2^	1.26 × 10^1^	4.00 × 10^2^	5
SaDE	3.00 × 10^2^	0.00 × 10^0^	3.00 × 10^2^	1.5	4.04 × 10^2^	3.52 × 10^0^	4.00 × 10^2^	3
Function	F3				F4			
Metric	Mean	Std	Best	Rank	Mean	Std	Best	Rank
SSMO	6.00 × 10^2^	2.66 × 10^−3^	6.00 × 10^2^	5	8.06 × 10^2^	2.73 × 10^0^	8.01 × 10^2^	3
RSDE	6.00 × 10^2^	2.95 × 10^−5^	6.00 × 10^2^	1	8.07 × 10^2^	2.77 × 10^0^	8.02 × 10^2^	4
L-SHADE-SPACMA	6.00 × 10^2^	4.46 × 10^−3^	6.00 × 10^2^	3	8.05 × 10^2^	2.02 × 10^0^	8.02 × 10^2^	2
L-SHADE-EpSin	6.00 × 10^2^	1.26 × 10^−2^	6.00 × 10^2^	4	8.05 × 10^2^	1.99 × 10^0^	8.02 × 10^2^	1
SaDE	6.00 × 10^2^	2.56 × 10^−4^	6.00 × 10^2^	2	8.09 × 10^2^	6.60 × 10^0^	8.00 × 10^2^	5
Function	F5				F6			
Metric	Mean	Std	Best	Rank	Mean	Std	Best	Rank
SSMO	9.00 × 10^2^	1.16 × 10^−5^	9.00 × 10^2^	1	1.80 × 10^3^	2.31 × 10^−1^	1.80 × 10^3^	1
RSDE	9.01 × 10^2^	2.17 × 10^0^	9.00 × 10^2^	5	1.82 × 10^3^	1.81 × 10^1^	1.80 × 10^3^	5
L-SHADE-SPACMA	9.00 × 10^2^	6.97 × 10^−1^	9.00 × 10^2^	4	1.81 × 10^3^	1.60 × 10^1^	1.80 × 10^3^	3
L-SHADE-EpSin	9.00 × 10^2^	3.94 × 10^−1^	9.00 × 10^2^	2	1.82 × 10^3^	2.12 × 10^1^	1.80 × 10^3^	4
SaDE	9.00 × 10^2^	4.95 × 10^−1^	9.00 × 10^2^	3	1.80 × 10^3^	5.25 × 10^0^	1.80 × 10^3^	2
Function	F7				F8			
Metric	Mean	Std	Best	Rank	Mean	Std	Best	Rank
SSMO	2.01 × 10^3^	9.40 × 10^0^	2.00 × 10^3^	3	2.20 × 10^3^	3.69 × 10^0^	2.20 × 10^3^	1
RSDE	2.01 × 10^3^	9.60 × 10^0^	2.00 × 10^3^	5	2.22 × 10^3^	8.99 × 10^0^	2.20 × 10^3^	5
L-SHADE-SPACMA	2.00 × 10^3^	6.93 × 10^0^	2.00 × 10^3^	1	2.21 × 10^3^	8.70 × 10^0^	2.20 × 10^3^	4
L-SHADE-EpSin	2.01 × 10^3^	9.41 × 10^0^	2.00 × 10^3^	2	2.21 × 10^3^	9.68 × 10^0^	2.20 × 10^3^	3
SaDE	2.01 × 10^3^	9.55 × 10^0^	2.00 × 10^3^	4	2.21 × 10^3^	8.98 × 10^0^	2.20 × 10^3^	2
Function	F9				F10			
Metric	Mean	Std	Best	Rank	Mean	Std	Best	Rank
SSMO	2.52 × 10^3^	2.36 × 10^1^	2.40 × 10^3^	2	2.51 × 10^3^	2.84 × 10^1^	2.50 × 10^3^	2
RSDE	2.49 × 10^3^	2.59 × 10^−1^	2.49 × 10^3^	1	2.54 × 10^3^	5.26 × 10^1^	2.50 × 10^3^	5
L-SHADE-SPACMA	2.53 × 10^3^	0.00 × 10^0^	2.53 × 10^3^	3.5	2.51 × 10^3^	2.77 × 10^1^	2.50 × 10^3^	1
L-SHADE-EpSin	2.53 × 10^3^	2.52 × 10^1^	2.53 × 10^3^	5	2.52 × 10^3^	3.84 × 10^1^	2.50 × 10^3^	3
SaDE	2.53 × 10^3^	0.00 × 10^0^	2.53 × 10^3^	3.5	2.53 × 10^3^	4.85 × 10^1^	2.50 × 10^3^	4
Function	F11				F12			
Metric	Mean	Std	Best	Rank	Mean	Std	Best	Rank
SSMO	2.60 × 10^3^	1.30 × 10^−2^	2.60 × 10^3^	1	2.86 × 10^3^	1.71 × 10^0^	2.86 × 10^3^	2
RSDE	2.72 × 10^3^	1.44 × 10^2^	2.60 × 10^3^	5	2.85 × 10^3^	2.26 × 10^0^	2.85 × 10^3^	1
L-SHADE-SPACMA	2.66 × 10^3^	9.49 × 10^1^	2.60 × 10^3^	3	2.86 × 10^3^	8.33 × 10^−1^	2.86 × 10^3^	3
L-SHADE-EpSin	2.67 × 10^3^	1.22 × 10^2^	2.60 × 10^3^	4	2.87 × 10^3^	1.32 × 10^0^	2.86 × 10^3^	5
SaDE	2.64 × 10^3^	6.77 × 10^1^	2.60 × 10^3^	2	2.87 × 10^3^	3.95 × 10^0^	2.86 × 10^3^	4

**Table 10:** Ablation study results for SSMO in the 30-dimensional CEC2017 benchmark suite.

Function	Metric	SSMO	SSMO-oElite	SSMO-oWind	SSMO-oMemory	SSMO-oForaging
F1	Mean	7.82 × 10^4^	1.01 × 10^7^	4.69 × 10^4^	7.16 × 10^4^	1.67 × 10^10^
	Std	2.48 × 10^4^	1.55 × 10^6^	2.19 × 10^4^	2.00 × 10^4^	7.34 × 10^9^
F3	Mean	3.02 × 10^2^	3.41 × 10^2^	3.02 × 10^2^	3.02 × 10^2^	3.55 × 10^4^
	Std	6.15 × 10^−1^	6.73 × 10^0^	5.82 × 10^−1^	7.65 × 10^−1^	1.15 × 10^4^
F4	Mean	4.36 × 10^2^	4.89 × 10^2^	4.71 × 10^2^	4.57 × 10^2^	2.67 × 10^3^
	Std	3.00 × 10^1^	1.80 × 10^1^	2.73 × 10^1^	3.40 × 10^1^	1.49 × 10^3^
F5	Mean	5.69 × 10^2^	6.22 × 10^2^	8.21 × 10^2^	6.14 × 10^2^	7.00 × 10^2^
	Std	1.78 × 10^1^	2.01 × 10^1^	5.47 × 10^1^	3.18 × 10^1^	1.76 × 10^1^
F6	Mean	6.01 × 10^2^	6.05 × 10^2^	6.69 × 10^2^	6.08 × 10^2^	6.42 × 10^2^
	Std	6.98 × 10^−1^	2.74 × 10^0^	6.87 × 10^0^	6.37 × 10^0^	6.01 × 10^0^
F7	Mean	7.78 × 10^2^	8.73 × 10^2^	8.44 × 10^2^	7.82 × 10^2^	1.03 × 10^3^
	Std	1.18 × 10^1^	2.48 × 10^1^	3.40 × 10^1^	1.67 × 10^1^	7.49 × 10^1^
F8	Mean	8.61 × 10^2^	8.94 × 10^2^	1.06 × 10^3^	8.82 × 10^2^	9.74 × 10^2^
	Std	1.35 × 10^1^	1.10 × 10^1^	4.17 × 10^1^	2.65 × 10^1^	2.49 × 10^1^
F9	Mean	9.01 × 10^2^	9.03 × 10^2^	7.88 × 10^3^	9.13 × 10^2^	3.55 × 10^3^
	Std	4.73 × 10^−1^	3.83 × 10^−1^	2.08 × 10^3^	7.18 × 10^0^	7.37 × 10^2^
F10	Mean	3.92 × 10^3^	4.81 × 10^3^	5.03 × 10^3^	4.40 × 10^3^	5.76 × 10^3^
	Std	6.99 × 10^2^	7.08 × 10^2^	7.89 × 10^2^	6.17 × 10^2^	5.23 × 10^2^
F11	Mean	1.15 × 10^3^	1.20 × 10^3^	1.21 × 10^3^	1.23 × 10^3^	1.53 × 10^3^
	Std	2.56 × 10^1^	4.72 × 10^1^	2.55 × 10^1^	5.28 × 10^1^	1.92 × 10^2^
F12	Mean	4.22 × 10^4^	1.46 × 10^6^	2.17 × 10^5^	6.15 × 10^4^	3.03 × 10^7^
	Std	2.49 × 10^4^	4.73 × 10^5^	5.98 × 10^4^	3.42 × 10^4^	3.02 × 10^7^
F13	Mean	1.49 × 10^3^	3.62 × 10^4^	1.11 × 10^4^	1.76 × 10^3^	1.14 × 10^4^
	Std	1.55 × 10^2^	1.53 × 10^4^	4.88 × 10^3^	3.02 × 10^2^	7.29 × 10^3^
F14	Mean	1.45 × 10^3^	1.52 × 10^3^	8.98 × 10^3^	1.50 × 10^3^	1.48 × 10^3^
	Std	1.33 × 10^1^	2.14 × 10^1^	6.48 × 10^3^	2.44 × 10^1^	1.55 × 10^1^
F15	Mean	1.53 × 10^3^	2.03 × 10^3^	2.15 × 10^3^	1.59 × 10^3^	2.06 × 10^3^
	Std	2.08 × 10^1^	3.03 × 10^2^	4.31 × 10^2^	6.30 × 10^1^	2.53 × 10^2^
F16	Mean	2.12 × 10^3^	2.35 × 10^3^	2.63 × 10^3^	2.12 × 10^3^	2.40 × 10^3^
	Std	2.18 × 10^2^	1.68 × 10^2^	1.99 × 10^2^	1.29 × 10^2^	1.83 × 10^2^
F17	Mean	1.85 × 10^3^	1.91 × 10^3^	2.08 × 10^3^	1.87 × 10^3^	1.96 × 10^3^
	Std	7.22 × 10^1^	6.22 × 10^1^	1.34 × 10^2^	6.61 × 10^1^	8.46 × 10^1^
F18	Mean	2.06 × 10^3^	2.16 × 10^4^	5.95 × 10^4^	2.05 × 10^3^	2.11 × 10^3^
	Std	1.56 × 10^2^	6.74 × 10^3^	4.28 × 10^4^	9.95 × 10^1^	1.08 × 10^2^
F19	Mean	1.92 × 10^3^	2.09 × 10^3^	5.40 × 10^3^	1.97 × 10^3^	2.01 × 10^3^
	Std	7.06 × 10^0^	6.29 × 10^1^	4.07 × 10^3^	3.12 × 10^1^	3.93 × 10^1^
F20	Mean	2.16 × 10^3^	2.28 × 10^3^	2.67 × 10^3^	2.23 × 10^3^	2.29 × 10^3^
	Std	7.26 × 10^1^	1.03 × 10^2^	1.70 × 10^2^	1.05 × 10^2^	1.17 × 10^2^
F21	Mean	2.37 × 10^3^	2.40 × 10^3^	2.56 × 10^3^	2.40 × 10^3^	2.49 × 10^3^
	Std	1.41 × 10^1^	1.79 × 10^1^	1.33 × 10^2^	2.47 × 10^1^	2.25 × 10^1^
F22	Mean	2.59 × 10^3^	4.26 × 10^3^	6.34 × 10^3^	3.93 × 10^3^	5.99 × 10^3^
	Std	1.10 × 10^3^	2.05 × 10^3^	7.66 × 10^2^	1.76 × 10^3^	1.45 × 10^3^
F23	Mean	2.69 × 10^3^	2.79 × 10^3^	3.55 × 10^3^	2.80 × 10^3^	2.93 × 10^3^
	Std	9.55 × 10^1^	2.94 × 10^1^	2.22 × 10^2^	3.90 × 10^1^	4.83 × 10^1^
F24	Mean	2.87 × 10^3^	2.92 × 10^3^	2.97 × 10^3^	2.96 × 10^3^	3.13 × 10^3^
	Std	2.05 × 10^1^	2.97 × 10^1^	1.98 × 10^2^	3.22 × 10^1^	8.93 × 10^1^
F25	Mean	2.89 × 10^3^	2.89 × 10^3^	2.89 × 10^3^	2.89 × 10^3^	3.43 × 10^3^
	Std	2.69 × 10^0^	3.76 × 10^0^	1.60 × 10^1^	6.26 × 10^0^	2.46 × 10^2^
F26	Mean	3.98 × 10^3^	4.28 × 10^3^	4.04 × 10^3^	5.08 × 10^3^	6.29 × 10^3^
	Std	6.22 × 10^2^	9.63 × 10^2^	1.90 × 10^3^	4.43 × 10^2^	3.93 × 10^2^
F27	Mean	3.21 × 10^3^	3.21 × 10^3^	3.54 × 10^3^	3.22 × 10^3^	3.29 × 10^3^
	Std	1.24 × 10^1^	1.25 × 10^1^	2.16 × 10^2^	1.41 × 10^1^	4.15 × 10^1^
F28	Mean	3.14 × 10^3^	3.20 × 10^3^	3.11 × 10^3^	3.16 × 10^3^	3.79 × 10^3^
	Std	6.04 × 10^1^	5.44 × 10^1^	3.42 × 10^1^	5.53 × 10^1^	3.39 × 10^2^
F29	Mean	3.51 × 10^3^	3.65 × 10^3^	4.09 × 10^3^	3.64 × 10^3^	3.94 × 10^3^
	Std	7.62 × 10^1^	1.92 × 10^2^	2.16 × 10^2^	1.06 × 10^2^	1.74 × 10^2^
F30	Mean	5.86 × 10^3^	5.81 × 10^4^	7.20 × 10^3^	5.38 × 10^3^	5.28 × 10^4^
	Std	6.02 × 10^2^	2.73 × 10^4^	1.03 × 10^3^	1.34 × 10^2^	4.99 × 10^4^
	+/=/−	/	24/3/2	16/12/1	27/2/0	24/5/0

**Table 11:** Candidate and adopted values of the main parameters in SSMO.

Parameter	Candidate Values	Adopted Value
*L*/*N*	0.05, 0.10, 0.15, 0.20, 0.25	0.15
*M*/*N*	0.30, 0.40, 0.50, 0.60, 0.70	0.60
*σ_η_*	0.10, 0.20, 0.30, 0.40, 0.50, 0.60	0.10
*q*	0.60, 0.80, 1.00, 1.20, 1.40	0.80

**Table 12:** Comparative results for the pressure vessel design problem.

Algorithm	Optimal Variables				Mean	Std	Best	Rank
	*x*1	*x*2	*x*3	*x*4				
SSMO	8.1250 × 10^−1^	4.3750 × 10^−1^	4.2098 × 10^1^	1.7664 × 10^2^	6.0659 × 10^3^	1.2536 × 10^1^	6.0597 × 10^3^	1
PSO	8.7500 × 10^−1^	4.3750 × 10^−1^	4.5337 × 10^1^	1.4025 × 10^2^	6.4662 × 10^3^	2.6552 × 10^2^	6.0905 × 10^3^	5
GWO	8.1250 × 10^−1^	4.3750 × 10^−1^	4.2099 × 10^1^	1.7664 × 10^2^	6.2201 × 10^3^	3.7320 × 10^2^	6.0598 × 10^3^	3
WOA	8.1250 × 10^−1^	6.2500 × 10^−1^	4.1429 × 10^1^	1.8512 × 10^2^	8.6718 × 10^3^	1.4698 × 10^3^	6.7152 × 10^3^	11
DBO	8.1250 × 10^−1^	4.3750 × 10^−1^	4.2098 × 10^1^	1.7664 × 10^2^	6.7456 × 10^3^	5.2550 × 10^2^	6.0597 × 10^3^	9
GWCA	8.1250 × 10^−1^	4.3750 × 10^−1^	4.2098 × 10^1^	1.7664 × 10^2^	6.6683 × 10^3^	4.2916 × 10^2^	6.0597 × 10^3^	6
SGA	8.1250 × 10^−1^	4.3750 × 10^−1^	4.2027 × 10^1^	1.7753 × 10^2^	6.7509 × 10^3^	5.4808 × 10^2^	6.0685 × 10^3^	10
IVY	8.1250 × 10^−1^	4.3750 × 10^−1^	4.2098 × 10^1^	1.7664 × 10^2^	6.7393 × 10^3^	4.5894 × 10^2^	6.0597 × 10^3^	8
FATA	8.1250 × 10^−1^	4.3750 × 10^−1^	4.2097 × 10^1^	1.7668 × 10^2^	6.4253 × 10^3^	4.5930 × 10^2^	6.0604 × 10^3^	4
PLO	8.1250 × 10^−1^	4.3750 × 10^−1^	4.2083 × 10^1^	1.7687 × 10^2^	6.1470 × 10^3^	4.5387 × 10^1^	6.0625 × 10^3^	2
SFOA	1.2500 × 10^0^	5.0000 × 10^−1^	5.1868 × 10^1^	8.6370 × 10^1^	2.0675 × 10^4^	1.2105 × 10^4^	7.9122 × 10^3^	12
EMWPA	8.1250 × 10^−1^	4.3750 × 10^−1^	4.2098 × 10^1^	1.7664 × 10^2^	6.6971 × 10^3^	5.6055 × 10^2^	6.0597 × 10^3^	7

**Table 13:** Comparative results for the welded beam design problem.

Algorithm	Optimal Variables				Mean	Std	Best	Rank
	*x*1	*x*2	*x*3	*x*4				
SSMO	1.9874 × 10^−1^	3.3394 × 10^0^	9.1956 × 10^0^	1.9885 × 10^−1^	1.6753 × 10^0^	2.9670 × 10^−3^	1.6711 × 10^0^	**1**
PSO	1.9875 × 10^−1^	3.3390 × 10^0^	9.1919 × 10^0^	1.9885 × 10^−1^	1.7974 × 10^0^	1.4962 × 10^−1^	1.6704 × 10^0^	6
GWO	1.9813 × 10^−1^	3.3554 × 10^0^	9.1876 × 10^0^	1.9904 × 10^−1^	1.6763 × 10^0^	3.2370 × 10^−3^	1.6724 × 10^0^	2
WOA	1.7540 × 10^−1^	3.6140 × 10^0^	9.9882 × 10^0^	1.9556 × 10^−1^	2.8953 × 10^0^	1.0917 × 10^0^	1.7780 × 10^0^	12
DBO	1.9873 × 10^−1^	3.3335 × 10^0^	9.2132 × 10^0^	1.9873 × 10^−1^	1.7666 × 10^0^	6.8075 × 10^−2^	1.6723 × 10^0^	4
GWCA	1.9881 × 10^−1^	3.3380 × 10^0^	9.1917 × 10^0^	1.9885 × 10^−1^	1.8712 × 10^0^	2.7118 × 10^−1^	1.6703 × 10^0^	8
SGA	1.9855 × 10^−1^	3.3536 × 10^0^	9.1827 × 10^0^	1.9924 × 10^−1^	1.9332 × 10^0^	4.0854 × 10^−1^	1.6735 × 10^0^	10
IVY	1.6113 × 10^−1^	4.2529 × 10^0^	9.1549 × 10^0^	2.0046 × 10^−1^	2.0269 × 10^0^	2.4543 × 10^−1^	1.7335 × 10^0^	11
FATA	1.9897 × 10^−1^	3.3373 × 10^0^	9.1873 × 10^0^	1.9908 × 10^−1^	1.6770 × 10^0^	5.0960 × 10^−3^	1.6715 × 10^0^	3
PLO	1.9469 × 10^−1^	3.4270 × 10^0^	9.2686 × 10^0^	1.9851 × 10^−1^	1.7730 × 10^0^	4.8782 × 10^−2^	1.6861 × 10^0^	5
SFOA	1.4721 × 10^−1^	4.6989 × 10^0^	9.1898 × 10^0^	1.9959 × 10^−1^	1.8972 × 10^0^	1.4489 × 10^−1^	1.7625 × 10^0^	9
EMWPA	1.9884 × 10^−1^	3.3373 × 10^0^	9.1919 × 10^0^	1.9884 × 10^−1^	1.8619 × 10^0^	4.1649 × 10^−1^	1.6702 × 10^0^	7

**Table 14:** Comparative results for the tension/compression spring design problem.

Algorithm	Optimal Variables			Mean	Std	Best	Rank
	*x*1	*x*2	*x*3				
SSMO	5.1715 × 10^−2^	3.5734 × 10^−1^	1.1253 × 10^1^	1.2706 × 10^−2^	5.4900 × 10^−5^	1.2665 × 10^−2^	1
PSO	5.1407 × 10^−2^	3.4998 × 10^−1^	1.1695 × 10^1^	1.3883 × 10^−2^	3.3750 × 10^−3^	1.2667 × 10^−2^	7
GWO	5.3144 × 10^−2^	3.9270 × 10^−1^	9.4575 × 10^0^	1.2836 × 10^−2^	1.6900 × 10^−4^	1.2707 × 10^−2^	3
WOA	5.1792 × 10^−2^	3.5920 × 10^−1^	1.1145 × 10^1^	1.3776 × 10^−2^	1.1660 × 10^−3^	1.2665 × 10^−2^	5
DBO	5.1378 × 10^−2^	3.4929 × 10^−1^	1.1738 × 10^1^	1.4246 × 10^−2^	2.2760 × 10^−3^	1.2667 × 10^−2^	11
GWCA	5.2415 × 10^−2^	3.7445 × 10^−1^	1.0321 × 10^1^	1.4033 × 10^−2^	1.7800 × 10^−3^	1.2675 × 10^−2^	8
SGA	5.0470 × 10^−2^	3.2809 × 10^−1^	1.3194 × 10^1^	1.4178 × 10^−2^	1.4460 × 10^−3^	1.2698 × 10^−2^	9
IVY	5.2357 × 10^−2^	3.7299 × 10^−1^	1.0395 × 10^1^	1.4197 × 10^−2^	1.6290 × 10^−3^	1.2673 × 10^−2^	10
FATA	5.1806 × 10^−2^	3.5947 × 10^−1^	1.1137 × 10^1^	1.2919 × 10^−2^	3.2600 × 10^−4^	1.2674 × 10^−2^	4
PLO	5.3053 × 10^−2^	3.9030 × 10^−1^	9.5662 × 10^0^	1.2805 × 10^−2^	7.5800 × 10^−5^	1.2706 × 10^−2^	2
SFOA	5.0317 × 10^−2^	3.2364 × 10^−1^	1.3592 × 10^1^	1.3783 × 10^−2^	1.9440 × 10^−3^	1.2776 × 10^−2^	6
EMWPA	5.1629 × 10^−2^	3.5529 × 10^−1^	1.1373 × 10^1^	1.6322 × 10^−2^	5.9120 × 10^−3^	1.2665 × 10^−2^	12

**Table 15:** Comparative results for the gear train design problem.

Algorithm	Optimal Variables				Mean	Std	Best	Rank
	*x*1	*x*2	*x*3	*x*4				
SSMO	43	19	16	49	5.6463 × 10^−11^	1.6680 × 10^−10^	2.7009 × 10^−12^	**1**
PSO	49	19	16	43	3.2539 × 10^−9^	5.9542 × 10^−9^	2.7009 × 10^−12^	6
GWO	49	19	16	43	4.5112 × 10^−10^	6.2144 × 10^−10^	2.7009 × 10^−12^	2
WOA	43	19	16	49	5.5060 × 10^−9^	9.2918 × 10^−9^	2.7009 × 10^−12^	8
DBO	53	30	13	51	4.2415 × 10^−9^	6.9229 × 10^−9^	2.3078 × 10^−11^	7
GWCA	43	19	16	49	2.8659 × 10^−9^	3.7512 × 10^−9^	2.7009 × 10^−12^	5
SGA	49	16	19	43	5.7002 × 10^−9^	8.7041 × 10^−9^	2.7009 × 10^−12^	9
IVY	53	15	26	51	3.4891 × 10^−7^	5.5726 × 10^−7^	2.3078 × 10^−11^	11
FATA	49	16	19	43	8.5759 × 10^−10^	8.6151 × 10^−10^	2.7009 × 10^−12^	3
PLO	57	13	31	49	9.4217 × 10^−10^	4.6319 × 10^−10^	9.9399 × 10^−11^	4
SFOA	31	15	17	57	1.3167 × 10^−6^	5.9460 × 10^−6^	1.0936 × 10^−9^	12
EMWPA	43	16	19	49	1.6765 × 10^−7^	3.1610 × 10^−7^	2.7009 × 10^−12^	10

**Table 16:** Comparative results for the robot gripper design problem.

Algorithm	Optimal Variables							Mean	Std	Best	Rank
	*x*1	*x*2	*x*3	*x*4	*x*5	*x*6	*x*7				
SSMO	1.5000 × 10^2^	1.4988 × 10^2^	2.0000 × 10^2^	1.5367 × 10^−3^	1.5000 × 10^2^	1.0096 × 10^2^	2.2993 × 10^0^	2.6559 × 10^0^	1.5836 × 10^−1^	2.5443 × 10^0^	1
PSO	1.5000 × 10^2^	1.2267 × 10^2^	1.8029 × 10^2^	2.7052 × 10^1^	1.1463 × 10^2^	1.0860 × 10^2^	2.4401 × 10^0^	4.3194 × 10^0^	5.1308 × 10^−1^	3.2394 × 10^0^	5
GWO	1.4963 × 10^2^	1.4947 × 10^2^	1.9619 × 10^2^	0.0000 × 10^0^	5.9149 × 10^1^	1.0478 × 10^2^	1.8561 × 10^0^	3.6893 × 10^0^	4.5070 × 10^−1^	2.7017 × 10^0^	4
WOA	1.4317 × 10^2^	1.2848 × 10^2^	1.7437 × 10^2^	1.3881 × 10^1^	1.7051 × 10^1^	1.2439 × 10^2^	1.8064 × 10^0^	1.2165 × 10^1^	1.8692 × 10^1^	3.7096 × 10^0^	9
DBO	1.5000 × 10^2^	1.4980 × 10^2^	2.0000 × 10^2^	0.0000 × 10^0^	1.2841 × 10^2^	1.0789 × 10^2^	2.2565 × 10^0^	4.9155 × 10^0^	1.5392 × 10^0^	2.6861 × 10^0^	6
GWCA	1.5000 × 10^2^	1.4988 × 10^2^	2.0000 × 10^2^	1.9205 ×10^−12^	1.5000 × 10^2^	1.0133 × 10^2^	2.3289 × 10^0^	6.6701 × 10^0^	1.1338 × 10^1^	2.5507 × 10^0^	8
SGA	1.4999 × 10^2^	1.4997 × 10^2^	1.9999 × 10^2^	9.2696 × 10^−3^	1.4999 × 10^2^	1.1160 × 10^2^	2.4483 × 10^0^	1.6152 × 10^13^	4.2869 × 10^13^	4.6222 × 10^0^	12
IVY	1.3191 × 10^2^	1.2212 × 10^2^	1.7287 × 10^2^	2.2833 × 10^0^	1.4993 × 10^2^	1.6534 × 10^2^	2.7667 × 10^0^	1.1701 × 10^12^	6.4002 × 10^12^	5.0167 × 10^0^	11
FATA	1.4992 × 10^2^	1.4979 × 10^2^	2.0000 × 10^2^	3.7053 × 10^−3^	1.4002 × 10^2^	1.0245 × 10^2^	2.2685 × 10^0^	3.6844 × 10^0^	5.0747 × 10^−1^	2.6013 × 10^0^	3
PLO	1.5000 × 10^2^	1.4986 × 10^2^	1.9941 × 10^2^	0.0000 × 10^0^	1.4980 × 10^2^	1.0279 × 10^2^	2.3238 × 10^0^	3.4559 × 10^0^	5.1641 × 10^−1^	2.5853 × 10^0^	2
SFOA	1.5000 × 10^2^	1.5000 × 10^2^	2.0000 × 10^2^	0.0000 × 10^0^	1.5000 × 10^2^	1.0000 × 10^2^	2.3380 × 10^0^	1.2038 × 10^10^	6.2561 × 10^10^	4.2893 × 10^0^	10
EMWPA	1.4990 × 10^2^	1.4978 × 10^2^	2.0000 × 10^2^	3.5708 × 10^−5^	1.4954 × 10^2^	1.0123 × 10^2^	2.3202 × 10^0^	5.0490 × 10^0^	3.4566 × 10^0^	2.5507 × 10^0^	7

**Table 17:** Maximum constraint violations of the best-run engineering design solutions.

Algorithm	Pressure Vessel	Welded Beam	Spring	Robot Gripper
SSMO	3.1722 × 10^−7^	0	0	0
PSO	2.3225 × 10^−7^	0	0	0
GWO	1.4731 × 10^−6^	0	0	0
WOA	0	0	0	0
DBO	3.2147 × 10^−7^	0	2.5000 × 10^−12^	0
GWCA	2.6908 × 10^−7^	0	1.1999 × 10^−12^	0
SGA	0	0	0	0
IVY	0	0	0	0
FATA	0	0	0	0
PLO	0	0	0	0
SFOA	0	0	0	0
EMWPA	3.1589 × 10^−7^	0	1.2157 × 10^−12^	0

**Table 18:** Wilcoxon rank-sum test results for engineering design problems.

Engineering Problem	Win	Tie	Loss
Pressure vessel	11	0	0
Welded beam	8	3	0
Spring	11	0	0
Gear train	11	0	0
Robot gripper	11	0	0

**Table 19:** Statistics of RMSE metrics of SSMO and other algorithms in SDM.

Algorithm	Max	Min	Mean	Median	Std
SSMO	1.7493× 10^−3^	9.8602 × 10^−4^	1.0115 × 10^−3^	9.8603 × 10^−4^	1.3936 × 10^−4^
PSO	3.2487 × 10^−3^	9.8618 × 10^−4^	1.5365 × 10^−3^	1.3789 × 10^−3^	4.9677 × 10^−4^
GWO	4.3522 × 10^−2^	1.6369 × 10^−3^	1.1074 × 10^−2^	6.9236 × 10^−3^	1.2443 × 10^−2^
WOA	5.3650 × 10^−2^	1.2896 × 10^−3^	1.3481 × 10^−2^	6.1971 × 10^−3^	1.6153 × 10^−2^
DBO	3.8151 × 10^−2^	9.9008 × 10^−4^	1.5203 × 10^−2^	2.4480 × 10^−3^	1.7682 × 10^−2^
GWCA	2.1592 × 10^−3^	9.8659 × 10^−4^	1.4764 × 10^−3^	1.4860 × 10^−3^	3.3830 × 10^−4^
SGA	3.0101 × 10^−1^	8.5679 × 10^−3^	1.5648 × 10^−1^	1.5433 × 10^−1^	8.7179 × 10^−2^
IVY	3.9710 × 10^−2^	2.4805 × 10^−3^	7.4594 × 10^−3^	3.4358 × 10^−3^	8.1419 × 10^−3^
FATA	1.7773 × 10^−2^	4.4147 × 10^−3^	8.2495 × 10^−3^	7.4602 × 10^−3^	3.0657 × 10^−3^
PLO	2.4740 × 10^−3^	1.6236 × 10^−3^	2.2979 × 10^−3^	2.3879 × 10^−3^	2.1737 × 10^−4^
SFOA	4.5257 × 10^−2^	5.4255 × 10^−3^	2.0696 × 10^−2^	1.8292 × 10^−2^	1.1983 × 10^−2^
EMWPA	2.0699 × 10^−1^	9.9159 × 10^−4^	8.3083 × 10^−3^	1.2897 × 10^−3^	3.7530 × 10^−2^

**Table 20:** Statistics of RMSE metrics of SSMO and other algorithms in DDM.

Algorithm	Max	Min	Mean	Median	Std
SSMO	1.5698 × 10^−3^	9.8253 × 10^−4^	1.0092 × 10^−3^	9.8592 × 10^−4^	1.0654 × 10^−4^
PSO	3.3392 × 10^−2^	9.8720 × 10^−4^	2.6622 × 10^−3^	1.4994 × 10^−3^	5.8246 × 10^−3^
GWO	4.4535 × 10^−2^	1.1662 × 10^−3^	8.3869 × 10^−3^	3.5621 × 10^−3^	1.1727 × 10^−2^
WOA	5.0977 × 10^−2^	1.3132 × 10^−3^	1.8251 × 10^−2^	1.0383 × 10^−2^	1.7195 × 10^−2^
DBO	4.6014 × 10^−2^	9.9502 × 10^−4^	8.6778 × 10^−3^	2.1275 × 10^−3^	1.3985 × 10^−2^
GWCA	2.8672 × 10^−3^	9.8540 × 10^−4^	1.7647 × 10^−3^	1.8954 × 10^−3^	4.5956 × 10^−4^
SGA	2.5921 × 10^−1^	3.0013 × 10^−3^	1.2096 × 10^−1^	1.1349 × 10^−1^	7.4664 × 10^−2^
IVY	4.0268 × 10^−2^	2.0126 × 10^−3^	7.4298 × 10^−3^	3.7294 × 10^−3^	8.4729 × 10^−3^
FATA	2.7071 × 10^−2^	3.5248 × 10^−3^	9.2421 × 10^−3^	7.4860 × 10^−3^	5.7647 × 10^−3^
PLO	3.2526 × 10^−3^	2.3382 × 10^−3^	2.9650 × 10^−3^	3.0757 × 10^−3^	2.4693 × 10^−4^
SFOA	5.3798 × 10^−2^	4.1820 × 10^−3^	1.6932 × 10^−2^	1.1954 × 10^−2^	1.3483 × 10^−2^
EMWPA	2.6283 × 10^−3^	9.8407 × 10^−4^	1.5248 × 10^−3^	1.4772 × 10^−3^	4.9438 × 10^−4^

**Table 21:** Statistics of RMSE metrics of SSMO and other algorithms in TDM.

Algorithm	Max	Min	Mean	Median	Std
SSMO	2.5092 × 10^−3^	9.8301 × 10^−4^	1.1477 × 10^−3^	9.9209 × 10^−4^	3.3102 × 10^−4^
PSO	3.0531 × 10^−2^	9.9036 × 10^−4^	2.9056 × 10^−3^	1.9090 × 10^−3^	5.2477 × 10^−3^
GWO	4.2838 × 10^−2^	1.1267 × 10^−3^	1.0174 × 10^−2^	5.0796 × 10^−3^	1.2451 × 10^−2^
WOA	2.2333 × 10^−1^	1.6431 × 10^−3^	2.5718 × 10^−2^	1.1337 × 10^−2^	4.1337 × 10^−2^
DBO	4.1032 × 10^−2^	1.2776 × 10^−3^	9.0987 × 10^−3^	2.3597 × 10^−3^	1.2802 × 10^−2^
GWCA	3.2511 × 10^−3^	9.9208 × 10^−4^	1.8663 × 10^−3^	1.7994 × 10^−3^	6.8292 × 10^−4^
SGA	4.5937 × 10^−1^	1.5574 × 10^−2^	1.8023 × 10^−1^	1.6888 × 10^−1^	1.2219 × 10^−1^
IVY	1.2853 × 10^−1^	2.4615 × 10^−3^	1.8280 × 10^−2^	4.7141 × 10^−3^	3.1635 × 10^−2^
FATA	3.7695 × 10^−2^	2.9586 × 10^−3^	9.0220 × 10^−3^	7.1987 × 10^−3^	6.8179 × 10^−3^
PLO	4.0611 × 10^−3^	1.5610 × 10^−3^	3.6006 × 10^−3^	3.8323 × 10^−3^	5.2841 × 10^−4^
SFOA	5.5822 × 10^−2^	2.3335 × 10^−3^	1.7890 × 10^−2^	1.2938 × 10^−2^	1.3666 × 10^−2^
EMWPA	3.6298 × 10^−2^	9.8588 × 10^−4^	2.7462 × 10^−3^	1.4549 × 10^−3^	6.3591 × 10^−3^

**Table 22:** Statistics of RMSE metrics of SSMO and other algorithms in PVM.

Algorithm	Max	Min	Mean	Median	Std
SSMO	5.6651 × 10^−3^	2.4400 × 10^−3^	3.4122 × 10^−3^	2.9974 × 10^−3^	1.0193 × 10^−3^
PSO	1.5537 × 10^−2^	8.2656 × 10^−3^	1.2301 × 10^−2^	1.2664 × 10^−2^	1.8618 × 10^−3^
GWO	4.3175 × 10^−2^	6.6379 × 10^−3^	2.3959 × 10^−2^	2.2547 × 10^−2^	7.6662 × 10^−3^
WOA	2.7705 × 10^−1^	1.0577 × 10^−2^	3.6025 × 10^−2^	2.4248 × 10^−2^	4.7014 × 10^−2^
DBO	6.6818 × 10^−2^	2.5806 × 10^−3^	1.7471 × 10^−2^	1.3924 × 10^−2^	1.3495 × 10^−2^
GWCA	1.4266 × 10^−2^	4.0161 × 10^−3^	1.1851 × 10^−2^	1.2368 × 10^−2^	2.1781 × 10^−3^
SGA	4.4028 × 10^−1^	2.6510 × 10^−2^	1.8719 × 10^−1^	1.7666 × 10^−1^	1.3255 × 10^−1^
IVY	2.7830 × 10^−1^	1.2124 × 10^−2^	4.9148 × 10^−2^	3.2027 × 10^−2^	5.8272 × 10^−2^
FATA	3.2650 × 10^−2^	1.2698 × 10^−2^	2.0761 × 10^−2^	1.9767 × 10^−2^	5.1243 × 10^−3^
PLO	1.9294 × 10^−2^	1.1024 × 10^−2^	1.6777 × 10^−2^	1.7527 × 10^−2^	2.5699 × 10^−3^
SFOA	7.9166 × 10^−2^	1.5027 × 10^−2^	3.3129 × 10^−2^	2.9667 × 10^−2^	1.3959 × 10^−2^
EMWPA	1.2925 × 10^−2^	2.9508 × 10^−3^	6.8110 × 10^−3^	6.7469 × 10^−3^	2.3592 × 10^−3^

**Table 23:** Statistical results of five algorithms on four photovoltaic models.

	Algorithm	Max	Min	Mean	Median	Std
	SSMO	1.2724 × 10^−3^	9.8603 × 10^−4^	1.0185 × 10^−3^	9.8706 × 10^−4^	7.3447 × 10^−5^
	SAO	2.7574 × 10^−3^	9.8981 × 10^−4^	1.7620 × 10^−3^	1.9063 × 10^−3^	5.3105 × 10^−4^
SDM	BBO	2.2792 × 10^−3^	9.8760 × 10^−4^	1.6051 × 10^−3^	1.5871 × 10^−3^	3.5498 × 10^−4^
	MSSO	5.0302 × 10^−3^	1.5815 × 10^−3^	3.1047 × 10^−3^	3.0370 × 10^−3^	8.1287 × 10^−4^
	RITLBO	1.5779 × 10^−3^	9.8882 × 10^−4^	1.1267 × 10^−3^	1.0882 × 10^−3^	1.4064 × 10^−4^
	SSMO	2.3353 × 10^−3^	9.8326 × 10^−4^	1.1000 × 10^−3^	9.8621 × 10^−4^	2.7147 × 10^−4^
	SAO	3.1679 × 10^−3^	9.9453 × 10^−4^	1.9043 × 10^−3^	1.9986 × 10^−3^	5.5634 × 10^−4^
DDM	BBO	3.2567 × 10^−3^	1.1360 × 10^−3^	2.1295 × 10^−3^	2.0747 × 10^−3^	5.3286 × 10^−4^
	MSSO	5.5918 × 10^−3^	1.3409 × 10^−3^	2.6554 × 10^−3^	2.6344 × 10^−3^	1.0349 × 10^−3^
	RITLBO	2.0744 × 10^−3^	9.9225 × 10^−4^	1.3773 × 10^−3^	1.4045 × 10^−3^	2.5464 × 10^−4^
	SSMO	2.5181 × 10^−3^	9.8268 × 10^−4^	1.2704 × 10^−3^	1.0787 × 10^−3^	4.3215 × 10^−4^
	SAO	3.3957 × 10^−3^	9.8361 × 10^−4^	1.8868 × 10^−3^	1.8946 × 10^−3^	5.4981 × 10^−4^
TDM	BBO	3.8767 × 10^−3^	1.0912 × 10^−3^	2.6349 × 10^−3^	2.6886 × 10^−3^	6.8602 × 10^−4^
	MSSO	5.9454 × 10^−3^	2.9494 × 10^−3^	4.4709 × 10^−3^	4.2973 × 10^−3^	8.2414 × 10^−4^
	RITLBO	2.6611 × 10^−3^	9.9439 × 10^−4^	1.5730 × 10^−3^	1.5265 × 10^−3^	3.9093 × 10^−4^
	SSMO	5.7158 × 10^−3^	2.4257 × 10^−3^	3.0030 × 10^−3^	2.7508 × 10^−3^	7.2257 × 10^−4^
	SAO	1.7860 × 10^−2^	2.6347 × 10^−3^	1.3119 × 10^−2^	1.4152 × 10^−2^	4.2234 × 10^−3^
PVM	BBO	1.7976 × 10^−2^	1.3011 × 10^−2^	1.5943 × 10^−2^	1.5958 × 10^−2^	1.2000 × 10^−3^
	MSSO	2.3859 × 10^−2^	1.7011 × 10^−2^	2.0506 × 10^−2^	2.0560 × 10^−2^	1.8056 × 10^−3^
	RITLBO	8.2609 × 10^−3^	2.8268 × 10^−3^	5.0638 × 10^−3^	5.0596 × 10^−3^	1.2434 × 10^−3^

**Table 24:** Holm-adjusted Wilcoxon rank-sum test results for four PV models.

Model	SSMO vs. SAO	SSMO vs. BBO	SSMO vs. MSSO	SSMO vs. RITLBO
SDM	1.951 × 10^−8^ (+)	5.072 × 10^−9^ (+)	4.832 × 10^−10^ (+)	3.323 × 10^−6^ (+)
DDM	9.250 × 10^−8^ (+)	8.002 × 10^−9^ (+)	2.348 × 10^−9^ (+)	2.240 × 10^−6^ (+)
TDM	9.885 × 10^−5^ (+)	2.045 × 10^−8^ (+)	4.832 × 10^−10^ (+)	6.203 × 10^−4^ (+)
PVM	2.871 × 10^−9^ (+)	4.832 × 10^−10^ (+)	4.832 × 10^−10^ (+)	5.556 × 10^−8^ (+)

## Data Availability

The original contributions presented in this study are included in the article. Further inquiries can be directed to the corresponding author.

## Appendix A

**Table A1:** Experimental results of 12 algorithms in CEC 2017 (Dim = 30).

Algorithm	Metric	SSMO	PSO	GWO	WOA	DBO	GWCA	SGA	IVY	FATA	PLO	SFOA	EMWPA
F1	Mean	7.82 × 10^4^	3.42 × 10^7^	2.21 × 10^9^	2.88 × 10^6^	2.21 × 10^4^	7.88 × 10^3^	2.75 × 10^6^	4.54 × 10^3^	3.15 × 10^7^	1.08 × 10^4^	8.17 × 10^9^	2.29 × 10^3^
	Std	2.48 × 10^4^	1.87 × 10^8^	1.94 × 10^9^	1.98 × 10^6^	4.13 × 10^4^	7.62 × 10^3^	5.55 × 10^6^	6.19 × 10^3^	1.52 × 10^7^	2.09 × 10^3^	3.21 × 10^9^	3.42 × 10^3^
	Best	4.56 × 10^4^	1.03 × 10^2^	1.50 × 10^8^	5.32 × 10^5^	3.69 × 10^2^	1.03 × 10^2^	6.04 × 10^4^	1.48 × 10^2^	9.47 × 10^6^	6.81 × 10^3^	4.00 × 10^9^	1.80 × 10^2^
F3	Mean	3.02 × 10^2^	3.05 × 10^2^	3.50 × 10^4^	1.66 × 10^5^	7.11 × 10^3^	3.00 × 10^2^	3.98 × 10^3^	4.07 × 10^3^	4.21 × 10^4^	2.80 × 10^4^	1.23 × 10^5^	1.96 × 10^3^
	Std	6.15 × 10^−1^	2.86 × 10^0^	1.02 × 10^4^	5.84 × 10^4^	1.16 × 10^4^	1.81 × 10^−12^	1.66 × 10^3^	1.11 × 10^3^	1.45 × 10^4^	5.45 × 10^3^	3.34 × 10^4^	3.20 × 10^3^
	Best	3.01 × 10^2^	3.01 × 10^2^	1.32 × 10^4^	7.10 × 10^4^	3.01 × 10^2^	3.00 × 10^2^	1.36 × 10^3^	2.64 × 10^3^	1.14 × 10^4^	1.69 × 10^4^	6.95 × 10^4^	3.00 × 10^2^
F4	Mean	4.36 × 10^2^	4.89 × 10^2^	5.84 × 10^2^	5.40 × 10^2^	5.21 × 10^2^	4.56 × 10^2^	5.17 × 10^2^	4.38 × 10^2^	4.98 × 10^2^	4.73 × 10^2^	1.29 × 10^3^	4.55 × 10^2^
	Std	3.00 × 10^1^	1.75 × 10^1^	9.05 × 10^1^	2.90 × 10^1^	3.58 × 10^1^	3.28 × 10^1^	3.42 × 10^1^	3.44 × 10^1^	2.07 × 10^1^	1.30 × 10^1^	3.70 × 10^2^	3.51 × 10^1^
	Best	4.00 × 10^2^	4.64 × 10^2^	5.06 × 10^2^	4.76 × 10^2^	4.72 × 10^2^	4.00 × 10^2^	4.65 × 10^2^	4.00 × 10^2^	4.69 × 10^2^	4.42 × 10^2^	8.57 × 10^2^	4.04 × 10^2^
F5	Mean	5.69 × 10^2^	6.82 × 10^2^	6.12 × 10^2^	8.05 × 10^2^	7.29 × 10^2^	6.70 × 10^2^	7.60 × 10^2^	7.11 × 10^2^	7.27 × 10^2^	5.52 × 10^2^	8.48 × 10^2^	7.21 × 10^2^
	Std	1.78 × 10^1^	3.69 × 10^1^	3.13 × 10^1^	6.63 × 10^1^	4.64 × 10^1^	3.83 × 10^1^	3.86 × 10^1^	5.46 × 10^1^	2.66 × 10^1^	7.54 × 10^0^	4.75 × 10^1^	2.77 × 10^1^
	Best	5.40 × 10^2^	5.83 × 10^2^	5.73 × 10^2^	6.73 × 10^2^	6.34 × 10^2^	5.96 × 10^2^	6.77 × 10^2^	6.35 × 10^2^	6.76 × 10^2^	5.38 × 10^2^	7.38 × 10^2^	6.85 × 10^2^
F6	Mean	6.01 × 10^2^	6.39 × 10^2^	6.09 × 10^2^	6.71 × 10^2^	6.44 × 10^2^	6.42 × 10^2^	6.59 × 10^2^	6.19 × 10^2^	6.49 × 10^2^	6.04 × 10^2^	6.75 × 10^2^	6.55 × 10^2^
	Std	6.98 × 10^−1^	7.73 × 10^0^	4.85 × 10^0^	9.88 × 10^0^	1.24 × 10^1^	9.83 × 10^0^	8.64 × 10^0^	1.75 × 10^1^	5.52 × 10^0^	8.31 × 10^−1^	1.56 × 10^1^	8.97 × 10^0^
	Best	6.00 × 10^2^	6.21 × 10^2^	6.03 × 10^2^	6.50 × 10^2^	6.19 × 10^2^	6.21 × 10^2^	6.40 × 10^2^	6.02 × 10^2^	6.33 × 10^2^	6.02 × 10^2^	6.44 × 10^2^	6.40 × 10^2^
F7	Mean	7.78 × 10^2^	8.15 × 10^2^	8.50 × 10^2^	1.22 × 10^3^	9.61 × 10^2^	1.14 × 10^3^	1.14 × 10^3^	1.23 × 10^3^	9.72 × 10^2^	8.16 × 10^2^	1.21 × 10^3^	1.15 × 10^3^
	Std	1.18 × 10^1^	3.91 × 10^1^	3.77 × 10^1^	9.26 × 10^1^	8.82 × 10^1^	9.90 × 10^1^	7.81 × 10^1^	9.51 × 10^1^	5.05 × 10^1^	1.23 × 10^1^	4.78 × 10^1^	8.73 × 10^1^
	Best	7.58 × 10^2^	7.53 × 10^2^	7.92 × 10^2^	1.03 × 10^3^	8.13 × 10^2^	1.01 × 10^3^	9.97 × 10^2^	1.06 × 10^3^	8.71 × 10^2^	7.90 × 10^2^	1.13 × 10^3^	1.02 × 10^3^
F8	Mean	8.61 × 10^2^	9.24 × 10^2^	8.94 × 10^2^	1.01 × 10^3^	1.00 × 10^3^	9.45 × 10^2^	9.76 × 10^2^	9.59 × 10^2^	9.58 × 10^2^	8.51 × 10^2^	1.12 × 10^3^	9.86 × 10^2^
	Std	1.35 × 10^1^	2.84 × 10^1^	2.78 × 10^1^	5.20 × 10^1^	4.75 × 10^1^	3.10 × 10^1^	3.63 × 10^1^	2.76 × 10^1^	1.90 × 10^1^	9.02 × 10^0^	3.81 × 10^1^	4.11 × 10^1^
	Best	8.41 × 10^2^	8.76 × 10^2^	8.53 × 10^2^	9.06 × 10^2^	8.95 × 10^2^	8.83 × 10^2^	8.92 × 10^2^	9.02 × 10^2^	9.16 × 10^2^	8.29 × 10^2^	1.03 × 10^3^	9.15 × 10^2^
F9	Mean	9.01 × 10^2^	3.84 × 10^3^	1.76 × 10^3^	7.63 × 10^3^	4.08 × 10^3^	3.88 × 10^3^	5.55 × 10^3^	5.22 × 10^3^	5.15 × 10^3^	1.24 × 10^3^	1.08 × 10^4^	4.93 × 10^3^
	Std	4.73 × 10^−1^	1.05 × 10^3^	4.88 × 10^2^	2.53 × 10^3^	1.07 × 10^3^	1.11 × 10^3^	1.03 × 10^3^	4.08 × 10^2^	7.27 × 10^2^	1.43 × 10^2^	2.40 × 10^3^	8.40 × 10^2^
	Best	9.00 × 10^2^	2.23 × 10^3^	1.09 × 10^3^	3.76 × 10^3^	1.80 × 10^3^	2.08 × 10^3^	3.72 × 10^3^	4.25 × 10^3^	2.75 × 10^3^	9.89 × 10^2^	5.00 × 10^3^	3.83 × 10^3^
F10	Mean	3.92 × 10^3^	4.40 × 10^3^	4.19 × 10^3^	5.92 × 10^3^	5.73 × 10^3^	5.30 × 10^3^	5.70 × 10^3^	5.32 × 10^3^	7.74 × 10^3^	3.32 × 10^3^	8.57 × 10^3^	5.68 × 10^3^
	Std	6.99 × 10^2^	6.22 × 10^2^	5.26 × 10^2^	7.41 × 10^2^	9.07 × 10^2^	6.63 × 10^2^	9.02 × 10^2^	7.10 × 10^2^	3.32 × 10^2^	2.53 × 10^2^	1.92 × 10^2^	1.05 × 10^3^
	Best	2.66 × 10^3^	3.37 × 10^3^	2.82 × 10^3^	4.43 × 10^3^	4.08 × 10^3^	4.15 × 10^3^	3.69 × 10^3^	3.54 × 10^3^	6.94 × 10^3^	2.79 × 10^3^	8.09 × 10^3^	4.45 × 10^3^
F11	Mean	1.15 × 10^3^	1.22 × 10^3^	1.85 × 10^3^	1.48 × 10^3^	1.38 × 10^3^	1.27 × 10^3^	1.29 × 10^3^	1.24 × 10^3^	1.28 × 10^3^	1.16 × 10^3^	3.25 × 10^3^	1.29 × 10^3^
	Std	2.56 × 10^1^	3.85 × 10^1^	7.95 × 10^2^	1.00 × 10^2^	1.01 × 10^2^	6.20 × 10^1^	4.06 × 10^1^	5.33 × 10^1^	3.66 × 10^1^	1.59 × 10^1^	8.81 × 10^2^	6.63 × 10^1^
	Best	1.11 × 10^3^	1.15 × 10^3^	1.25 × 10^3^	1.29 × 10^3^	1.20 × 10^3^	1.17 × 10^3^	1.20 × 10^3^	1.14 × 10^3^	1.20 × 10^3^	1.13 × 10^3^	2.04 × 10^3^	1.21 × 10^3^
F12	Mean	4.22 × 10^4^	2.62 × 10^5^	6.37 × 10^7^	4.00 × 10^7^	1.82 × 10^7^	4.83 × 10^4^	7.87 × 10^6^	3.84 × 10^5^	9.39 × 10^6^	4.72 × 10^5^	6.06 × 10^8^	4.98 × 10^4^
	Std	2.49 × 10^4^	1.91 × 10^5^	6.00 × 10^7^	2.27 × 10^7^	3.82 × 10^7^	8.09 × 10^4^	5.94 × 10^6^	2.96 × 10^5^	4.01 × 10^6^	2.68 × 10^5^	2.56 × 10^8^	1.86 × 10^4^
	Best	9.16 × 10^3^	4.03 × 10^4^	9.82 × 10^5^	2.64 × 10^6^	6.62 × 10^5^	5.70 × 10^3^	7.00 × 10^5^	5.26 × 10^4^	2.51 × 10^6^	1.93 × 10^5^	2.45 × 10^8^	1.83 × 10^4^
F13	Mean	1.49 × 10^3^	1.89 × 10^4^	1.48 × 10^7^	1.30 × 10^5^	8.03 × 10^5^	2.22 × 10^4^	1.30 × 10^5^	2.07 × 10^4^	9.21 × 10^4^	1.43 × 10^4^	2.00 × 10^8^	9.68 × 10^3^
	Std	1.55 × 10^2^	1.42 × 10^4^	5.62 × 10^7^	6.86 × 10^4^	1.06 × 10^6^	1.61 × 10^4^	1.06 × 10^5^	1.37 × 10^4^	1.90 × 10^5^	4.74 × 10^3^	1.73 × 10^8^	6.20 × 10^3^
	Best	1.36 × 10^3^	1.45 × 10^3^	2.38 × 10^4^	1.42 × 10^4^	1.61 × 10^4^	2.97 × 10^3^	2.59 × 10^4^	4.37 × 10^3^	2.06 × 10^4^	7.42 × 10^3^	5.70 × 10^7^	2.10 × 10^3^
F14	Mean	1.45 × 10^3^	7.86 × 10^3^	1.73 × 10^5^	7.17 × 10^5^	5.71 × 10^4^	5.72 × 10^3^	3.75 × 10^4^	1.35 × 10^4^	2.91 × 10^4^	8.15 × 10^3^	8.79 × 10^5^	5.10 × 10^3^
	Std	1.33 × 10^1^	6.56 × 10^3^	4.79 × 10^5^	6.65 × 10^5^	6.82 × 10^4^	4.17 × 10^3^	2.68 × 10^4^	1.14 × 10^4^	2.53 × 10^4^	4.61 × 10^3^	9.53 × 10^5^	4.40 × 10^3^
	Best	1.42 × 10^3^	1.70 × 10^3^	2.44 × 10^3^	1.07 × 10^4^	2.30 × 10^3^	1.69 × 10^3^	1.97 × 10^3^	1.99 × 10^3^	2.14 × 10^3^	2.39 × 10^3^	5.87 × 10^4^	1.66 × 10^3^
F15	Mean	1.53 × 10^3^	7.49 × 10^3^	3.34 × 10^5^	8.48 × 10^4^	7.08 × 10^4^	1.18 × 10^4^	5.98 × 10^4^	4.57 × 10^3^	8.97 × 10^3^	4.17 × 10^3^	1.37 × 10^7^	7.91 × 10^3^
	Std	2.08 × 10^1^	8.20 × 10^3^	6.88 × 10^5^	5.88 × 10^4^	5.21 × 10^4^	1.02 × 10^4^	6.42 × 10^4^	3.88 × 10^3^	4.55 × 10^3^	9.69 × 10^2^	9.00 × 10^6^	7.10 × 10^3^
	Best	1.51 × 10^3^	1.64 × 10^3^	1.38 × 10^4^	5.62 × 10^3^	1.03 × 10^4^	1.92 × 10^3^	4.57 × 10^3^	1.74 × 10^3^	3.86 × 10^3^	2.64 × 10^3^	1.49 × 10^6^	1.64 × 10^3^
F16	Mean	2.12 × 10^3^	2.74 × 10^3^	2.40 × 10^3^	3.58 × 10^3^	2.99 × 10^3^	2.85 × 10^3^	3.12 × 10^3^	2.94 × 10^3^	2.71 × 10^3^	2.05 × 10^3^	4.10 × 10^3^	2.96 × 10^3^
	Std	2.18 × 10^2^	3.13 × 10^2^	2.57 × 10^2^	3.81 × 10^2^	3.75 × 10^2^	3.90 × 10^2^	3.76 × 10^2^	3.26 × 10^2^	2.33 × 10^2^	9.06 × 10^1^	3.26 × 10^2^	3.49 × 10^2^
	Best	1.82 × 10^3^	1.98 × 10^3^	1.88 × 10^3^	2.68 × 10^3^	2.18 × 10^3^	2.02 × 10^3^	2.53 × 10^3^	2.21 × 10^3^	2.35 × 10^3^	1.87 × 10^3^	3.45 × 10^3^	2.37 × 10^3^
F17	Mean	1.85 × 10^3^	2.27 × 10^3^	1.97 × 10^3^	2.55 × 10^3^	2.33 × 10^3^	2.34 × 10^3^	2.37 × 10^3^	2.63 × 10^3^	2.05 × 10^3^	1.86 × 10^3^	2.80 × 10^3^	2.47 × 10^3^
	Std	7.22 × 10^1^	2.27 × 10^2^	1.36 × 10^2^	2.66 × 10^2^	2.21 × 10^2^	2.98 × 10^2^	1.94 × 10^2^	2.70 × 10^2^	1.78 × 10^2^	5.02 × 10^1^	2.58 × 10^2^	2.14 × 10^2^
	Best	1.76 × 10^3^	1.88 × 10^3^	1.77 × 10^3^	2.04 × 10^3^	1.94 × 10^3^	1.83 × 10^3^	1.94 × 10^3^	1.93 × 10^3^	1.80 × 10^3^	1.81 × 10^3^	2.31 × 10^3^	2.13 × 10^3^
F18	Mean	2.06 × 10^3^	1.37 × 10^5^	1.27 × 10^6^	2.82 × 10^6^	7.58 × 10^5^	6.57 × 10^4^	8.61 × 10^5^	1.10 × 10^5^	4.34 × 10^5^	1.35 × 10^5^	1.04 × 10^7^	4.26 × 10^4^
	Std	1.56 × 10^2^	9.03 × 10^4^	4.87 × 10^6^	2.72 × 10^6^	7.76 × 10^5^	4.07 × 10^4^	7.93 × 10^5^	5.24 × 10^4^	3.21 × 10^5^	6.25 × 10^4^	8.81 × 10^6^	6.94 × 10^4^
	Best	1.87 × 10^3^	4.47 × 10^4^	4.69 × 10^4^	1.35 × 10^5^	2.84 × 10^4^	9.53 × 10^3^	7.60 × 10^4^	4.09 × 10^4^	5.12 × 10^4^	4.91 × 10^4^	7.97 × 10^5^	4.01 × 10^3^
F19	Mean	1.92 × 10^3^	1.15 × 10^4^	1.56 × 10^6^	3.15 × 10^6^	1.50 × 10^5^	8.65 × 10^3^	9.49 × 10^4^	8.99 × 10^3^	8.79 × 10^3^	3.02 × 10^3^	1.66 × 10^7^	1.01 × 10^4^
	Std	7.06 × 10^0^	1.60 × 10^4^	6.03 × 10^6^	2.36 × 10^6^	1.39 × 10^5^	6.55 × 10^3^	1.01 × 10^5^	6.67 × 10^3^	4.78 × 10^3^	6.44 × 10^2^	1.06 × 10^7^	9.51 × 10^3^
	Best	1.91 × 10^3^	2.02 × 10^3^	3.56 × 10^3^	1.82 × 10^5^	9.15 × 10^3^	2.07 × 10^3^	6.79 × 10^3^	2.17 × 10^3^	2.49 × 10^3^	2.15 × 10^3^	2.68 × 10^6^	1.99 × 10^3^
F20	Mean	2.16 × 10^3^	2.56 × 10^3^	2.37 × 10^3^	2.71 × 10^3^	2.54 × 10^3^	2.65 × 10^3^	2.59 × 10^3^	2.83 × 10^3^	2.48 × 10^3^	2.16 × 10^3^	2.96 × 10^3^	2.74 × 10^3^
	Std	7.26 × 10^1^	1.66 × 10^2^	1.20 × 10^2^	1.70 × 10^2^	1.57 × 10^2^	1.61 × 10^2^	2.03 × 10^2^	1.84 × 10^2^	1.24 × 10^2^	5.06 × 10^1^	2.42 × 10^2^	1.21 × 10^2^
	Best	2.04 × 10^3^	2.28 × 10^3^	2.18 × 10^3^	2.36 × 10^3^	2.26 × 10^3^	2.33 × 10^3^	2.30 × 10^3^	2.31 × 10^3^	2.32 × 10^3^	2.10 × 10^3^	2.35 × 10^3^	2.52 × 10^3^
F21	Mean	2.37 × 10^3^	2.49 × 10^3^	2.38 × 10^3^	2.57 × 10^3^	2.51 × 10^3^	2.46 × 10^3^	2.53 × 10^3^	2.41 × 10^3^	2.48 × 10^3^	2.35 × 10^3^	2.62 × 10^3^	2.51 × 10^3^
	Std	1.41 × 10^1^	4.09 × 10^1^	1.65 × 10^1^	6.04 × 10^1^	5.82 × 10^1^	4.01 × 10^1^	4.64 × 10^1^	3.83 × 10^1^	5.66 × 10^1^	6.06 × 10^0^	3.60 × 10^1^	5.39 × 10^1^
	Best	2.35 × 10^3^	2.42 × 10^3^	2.35 × 10^3^	2.44 × 10^3^	2.34 × 10^3^	2.40 × 10^3^	2.44 × 10^3^	2.34 × 10^3^	2.22 × 10^3^	2.34 × 10^3^	2.55 × 10^3^	2.43 × 10^3^
F22	Mean	2.59 × 10^3^	4.93 × 10^3^	4.86 × 10^3^	6.46 × 10^3^	3.33 × 10^3^	5.01 × 10^3^	6.10 × 10^3^	5.08 × 10^3^	3.99 × 10^3^	3.35 × 10^3^	9.89 × 10^3^	5.61 × 10^3^
	Std	1.10 × 10^3^	1.92 × 10^3^	1.42 × 10^3^	2.27 × 10^3^	1.28 × 10^3^	2.17 × 10^3^	2.23 × 10^3^	2.54 × 10^3^	9.12 × 10^2^	1.34 × 10^3^	1.32 × 10^3^	2.36 × 10^3^
	Best	2.30 × 10^3^	2.30 × 10^3^	2.43 × 10^3^	2.32 × 10^3^	2.30 × 10^3^	2.30 × 10^3^	2.31 × 10^3^	2.30 × 10^3^	3.34 × 10^3^	2.31 × 10^3^	3.11 × 10^3^	2.30 × 10^3^
F23	Mean	2.69 × 10^3^	3.14 × 10^3^	2.76 × 10^3^	3.01 × 10^3^	2.91 × 10^3^	2.94 × 10^3^	3.10 × 10^3^	2.84 × 10^3^	2.86 × 10^3^	2.70 × 10^3^	3.01 × 10^3^	3.21 × 10^3^
	Std	9.55 × 10^1^	1.50 × 10^2^	3.13 × 10^1^	1.07 × 10^2^	9.82 × 10^1^	7.43 × 10^1^	1.38 × 10^2^	6.80 × 10^1^	4.31 × 10^1^	7.38 × 10^0^	4.91 × 10^1^	1.68 × 10^2^
	Best	2.42 × 10^3^	2.92 × 10^3^	2.71 × 10^3^	2.84 × 10^3^	2.78 × 10^3^	2.82 × 10^3^	2.88 × 10^3^	2.74 × 10^3^	2.78 × 10^3^	2.69 × 10^3^	2.91 × 10^3^	2.89 × 10^3^
F24	Mean	2.87 × 10^3^	3.24 × 10^3^	2.91 × 10^3^	3.18 × 10^3^	3.04 × 10^3^	3.09 × 10^3^	3.23 × 10^3^	2.97 × 10^3^	3.07 × 10^3^	2.88 × 10^3^	3.16 × 10^3^	3.40 × 10^3^
	Std	2.05 × 10^1^	9.85 × 10^1^	4.77 × 10^1^	9.34 × 10^1^	7.19 × 10^1^	6.82 × 10^1^	9.81 × 10^1^	8.55 × 10^1^	4.77 × 10^1^	1.11 × 10^1^	4.76 × 10^1^	2.72 × 10^2^
	Best	2.84 × 10^3^	3.08 × 10^3^	2.86 × 10^3^	3.00 × 10^3^	2.92 × 10^3^	2.98 × 10^3^	3.05 × 10^3^	2.88 × 10^3^	2.97 × 10^3^	2.86 × 10^3^	3.09 × 10^3^	3.18 × 10^3^
F25	Mean	2.89 × 10^3^	2.89 × 10^3^	2.99 × 10^3^	2.94 × 10^3^	2.91 × 10^3^	2.91 × 10^3^	2.92 × 10^3^	2.91 × 10^3^	2.90 × 10^3^	2.92 × 10^3^	3.39 × 10^3^	2.89 × 10^3^
	Std	2.69 × 10^0^	6.14 × 10^0^	4.37 × 10^1^	3.21 × 10^1^	3.38 × 10^1^	2.31 × 10^1^	2.55 × 10^1^	2.46 × 10^1^	1.82 × 10^1^	3.82 × 10^0^	1.68 × 10^2^	1.39 × 10^1^
	Best	2.89 × 10^3^	2.88 × 10^3^	2.93 × 10^3^	2.89 × 10^3^	2.88 × 10^3^	2.89 × 10^3^	2.89 × 10^3^	2.88 × 10^3^	2.88 × 10^3^	2.91 × 10^3^	3.11 × 10^3^	2.88 × 10^3^
F26	Mean	3.98 × 10^3^	5.64 × 10^3^	4.71 × 10^3^	7.37 × 10^3^	5.76 × 10^3^	5.59 × 10^3^	7.40 × 10^3^	7.26 × 10^3^	3.90 × 10^3^	3.98 × 10^3^	7.29 × 10^3^	6.90 × 10^3^
	Std	6.22 × 10^2^	2.05 × 10^3^	3.51 × 10^2^	8.43 × 10^2^	9.54 × 10^2^	1.52 × 10^3^	1.69 × 10^3^	1.57 × 10^3^	1.33 × 10^3^	4.87 × 10^2^	9.91 × 10^2^	1.57 × 10^3^
	Best	2.82 × 10^3^	2.80 × 10^3^	4.20 × 10^3^	5.61 × 10^3^	2.80 × 10^3^	2.80 × 10^3^	2.83 × 10^3^	2.90 × 10^3^	2.85 × 10^3^	2.92 × 10^3^	4.94 × 10^3^	2.80 × 10^3^
F27	Mean	3.21 × 10^3^	3.39 × 10^3^	3.25 × 10^3^	3.35 × 10^3^	3.31 × 10^3^	3.32 × 10^3^	3.35 × 10^3^	3.32 × 10^3^	3.38 × 10^3^	3.22 × 10^3^	3.36 × 10^3^	3.36 × 10^3^
	Std	1.24 × 10^1^	1.14 × 10^2^	2.42 × 10^1^	5.79 × 10^1^	5.93 × 10^1^	6.79 × 10^1^	7.09 × 10^1^	8.86 × 10^1^	6.83 × 10^1^	2.34 × 10^0^	5.34 × 10^1^	8.37 × 10^1^
	Best	3.19 × 10^3^	3.25 × 10^3^	3.21 × 10^3^	3.27 × 10^3^	3.22 × 10^3^	3.22 × 10^3^	3.24 × 10^3^	3.26 × 10^3^	3.25 × 10^3^	3.22 × 10^3^	3.28 × 10^3^	3.24 × 10^3^
F28	Mean	3.14 × 10^3^	3.21 × 10^3^	3.43 × 10^3^	3.30 × 10^3^	3.35 × 10^3^	3.18 × 10^3^	3.27 × 10^3^	3.13 × 10^3^	3.25 × 10^3^	3.21 × 10^3^	4.06 × 10^3^	3.20 × 10^3^
	Std	6.04 × 10^1^	4.29 × 10^1^	7.57 × 10^1^	4.03 × 10^1^	3.51 × 10^2^	5.21 × 10^1^	2.32 × 10^1^	6.01 × 10^1^	2.92 × 10^1^	5.73 × 10^0^	3.23 × 10^2^	4.88 × 10^1^
	Best	3.10 × 10^3^	3.10 × 10^3^	3.26 × 10^3^	3.23 × 10^3^	3.22 × 10^3^	3.10 × 10^3^	3.21 × 10^3^	3.10 × 10^3^	3.20 × 10^3^	3.20 × 10^3^	3.58 × 10^3^	3.11 × 10^3^
F29	Mean	3.51 × 10^3^	4.03 × 10^3^	3.75 × 10^3^	4.81 × 10^3^	4.30 × 10^3^	4.29 × 10^3^	4.67 × 10^3^	4.43 × 10^3^	3.89 × 10^3^	3.79 × 10^3^	4.91 × 10^3^	4.16 × 10^3^
	Std	7.62 × 10^1^	2.17 × 10^2^	1.45 × 10^2^	3.31 × 10^2^	3.53 × 10^2^	1.96 × 10^2^	3.39 × 10^2^	2.95 × 10^2^	2.42 × 10^2^	7.80 × 10^1^	3.69 × 10^2^	2.86 × 10^2^
	Best	3.34 × 10^3^	3.63 × 10^3^	3.48 × 10^3^	4.33 × 10^3^	3.69 × 10^3^	3.99 × 10^3^	4.10 × 10^3^	3.88 × 10^3^	3.54 × 10^3^	3.64 × 10^3^	4.19 × 10^3^	3.74 × 10^3^
F30	Mean	5.86 × 10^3^	9.28 × 10^3^	5.17 × 10^6^	1.16 × 10^7^	7.06 × 10^5^	1.01 × 10^4^	1.68 × 10^6^	1.17 × 10^4^	3.14 × 10^5^	2.25 × 10^4^	4.10 × 10^7^	6.66 × 10^3^
	Std	6.02 × 10^2^	3.68 × 10^3^	3.79 × 10^6^	8.57 × 10^6^	8.58 × 10^5^	3.07 × 10^3^	1.39 × 10^6^	3.76 × 10^3^	1.95 × 10^5^	6.89 × 10^3^	2.14 × 10^7^	1.54 × 10^3^
	Best	5.09 × 10^3^	5.70 × 10^3^	9.56 × 10^5^	5.04 × 10^5^	7.37 × 10^3^	5.41 × 10^3^	1.17 × 10^5^	7.92 × 10^3^	6.24 × 10^4^	1.18 × 10^4^	9.32 × 10^6^	5.33 × 10^3^

**Table A2:** Experimental results of 12 algorithms in CEC 2017 (Dim = 50).

Algorithm	Metric	SSMO	PSO	GWO	WOA	DBO	GWCA	SGA	IVY	FATA	PLO	SFOA	EMWPA
F1	Mean	4.81 × 10^5^	1.99 × 10^8^	8.37 × 10^9^	9.95 × 10^6^	2.90 × 10^6^	7.53 × 10^3^	1.89 × 10^8^	3.81 × 10^3^	3.12 × 10^8^	2.12 × 10^5^	2.69 × 10^10^	6.03 × 10^3^
	Std	9.44 × 10^4^	5.52 × 10^8^	5.01 × 10^9^	8.10 × 10^6^	5.91 × 10^6^	9.17 × 10^3^	9.02 × 10^8^	4.98 × 10^3^	1.05 × 10^8^	5.10 × 10^4^	6.87 × 10^9^	1.50 × 10^4^
	Best	3.23 × 10^5^	1.21 × 10^2^	2.72 × 10^9^	1.80 × 10^6^	8.19 × 10^2^	1.06 × 10^2^	2.02 × 10^6^	1.00 × 10^2^	1.61 × 10^8^	1.35 × 10^5^	1.51 × 10^10^	1.01 × 10^2^
F3	Mean	3.08 × 10^2^	3.81 × 10^2^	7.52 × 10^4^	9.26 × 10^4^	8.76 × 10^4^	3.00 × 10^2^	2.17 × 10^4^	4.41 × 10^4^	5.62 × 10^4^	1.02 × 10^5^	2.99 × 10^5^	5.34 × 10^4^
	Std	1.86 × 10^0^	2.07 × 10^1^	1.83 × 10^4^	3.69 × 10^4^	2.73 × 10^4^	1.36 × 10^−5^	4.05 × 10^3^	2.32 × 10^4^	2.07 × 10^4^	1.31 × 10^4^	7.45 × 10^4^	1.18 × 10^5^
	Best	3.05 × 10^2^	3.41 × 10^2^	4.05 × 10^4^	4.56 × 10^4^	2.86 × 10^4^	3.00 × 10^2^	1.67 × 10^4^	6.98 × 10^3^	2.37 × 10^4^	6.62 × 10^4^	1.86 × 10^5^	8.18 × 10^2^
F4	Mean	4.90 × 10^2^	5.68 × 10^2^	1.20 × 10^3^	6.89 × 10^2^	5.99 × 10^2^	4.90 × 10^2^	6.17 × 10^2^	4.69 × 10^2^	5.80 × 10^2^	5.17 × 10^2^	4.26 × 10^3^	5.18 × 10^2^
	Std	6.29 × 10^1^	6.68 × 10^1^	4.50 × 10^2^	5.92 × 10^1^	8.12 × 10^1^	5.35 × 10^1^	6.57 × 10^1^	3.51 × 10^1^	4.71 × 10^1^	2.31 × 10^1^	1.30 × 10^3^	6.37 × 10^1^
	Best	4.22 × 10^2^	4.30 × 10^2^	7.65 × 10^2^	5.39 × 10^2^	4.40 × 10^2^	4.21 × 10^2^	5.19 × 10^2^	4.00 × 10^2^	5.01 × 10^2^	4.70 × 10^2^	2.14 × 10^3^	4.26 × 10^2^
F5	Mean	6.79 × 10^2^	8.03 × 10^2^	7.11 × 10^2^	9.36 × 10^2^	9.45 × 10^2^	8.32 × 10^2^	8.79 × 10^2^	8.58 × 10^2^	8.60 × 10^2^	6.31 × 10^2^	1.12 × 10^3^	8.69 × 10^2^
	Std	3.50 × 10^1^	3.26 × 10^1^	3.53 × 10^1^	7.75 × 10^1^	7.27 × 10^1^	5.92 × 10^1^	3.98 × 10^1^	2.84 × 10^1^	2.37 × 10^1^	1.59 × 10^1^	5.19 × 10^1^	5.61 × 10^1^
	Best	6.04 × 10^2^	7.26 × 10^2^	6.56 × 10^2^	8.16 × 10^2^	8.19 × 10^2^	7.16 × 10^2^	8.01 × 10^2^	8.06 × 10^2^	8.24 × 10^2^	5.88 × 10^2^	1.02 × 10^3^	7.50 × 10^2^
F6	Mean	6.05 × 10^2^	6.47 × 10^2^	6.17 × 10^2^	6.76 × 10^2^	6.63 × 10^2^	6.52 × 10^2^	6.69 × 10^2^	6.32 × 10^2^	6.57 × 10^2^	6.11 × 10^2^	6.91 × 10^2^	6.58 × 10^2^
	Std	2.89 × 10^0^	6.36 × 10^0^	4.48 × 10^0^	8.69 × 10^0^	1.09 × 10^1^	6.85 × 10^0^	7.14 × 10^0^	1.66 × 10^1^	3.71 × 10^0^	1.38 × 10^0^	1.05 × 10^1^	7.84 × 10^0^
	Best	6.02 × 10^2^	6.35 × 10^2^	6.09 × 10^2^	6.62 × 10^2^	6.41 × 10^2^	6.36 × 10^2^	6.48 × 10^2^	6.10 × 10^2^	6.50 × 10^2^	6.08 × 10^2^	6.71 × 10^2^	6.40 × 10^2^
F7	Mean	8.47 × 10^2^	9.24 × 10^2^	1.06 × 10^3^	1.72 × 10^3^	1.37 × 10^3^	1.77 × 10^3^	1.66 × 10^3^	1.72 × 10^3^	1.36 × 10^3^	1.02 × 10^3^	1.75 × 10^3^	1.70 × 10^3^
	Std	2.13 × 10^1^	4.65 × 10^1^	4.74 × 10^1^	1.39 × 10^2^	1.15 × 10^2^	1.88 × 10^2^	8.99 × 10^1^	8.18 × 10^1^	8.95 × 10^1^	3.59 × 10^1^	1.04 × 10^2^	1.84 × 10^2^
	Best	8.07 × 10^2^	8.54 × 10^2^	9.69 × 10^2^	1.40 × 10^3^	1.14 × 10^3^	1.26 × 10^3^	1.50 × 10^3^	1.49 × 10^3^	1.22 × 10^3^	8.93 × 10^2^	1.52 × 10^3^	1.33 × 10^3^
F8	Mean	9.89 × 10^2^	1.11 × 10^3^	1.03 × 10^3^	1.22 × 10^3^	1.24 × 10^3^	1.13 × 10^3^	1.18 × 10^3^	1.18 × 10^3^	1.17 × 10^3^	9.30 × 10^2^	1.43 × 10^3^	1.19 × 10^3^
	Std	4.04 × 10^1^	4.69 × 10^1^	3.58 × 10^1^	6.45 × 10^1^	9.18 × 10^1^	5.20 × 10^1^	5.64 × 10^1^	3.46 × 10^1^	3.10 × 10^1^	1.41 × 10^1^	5.63 × 10^1^	5.05 × 10^1^
	Best	9.30 × 10^2^	1.01 × 10^3^	9.65 × 10^2^	1.12 × 10^3^	1.09 × 10^3^	9.84 × 10^2^	1.05 × 10^3^	1.07 × 10^3^	1.10 × 10^3^	9.05 × 10^2^	1.34 × 10^3^	1.09 × 10^3^
F9	Mean	1.23 × 10^3^	1.25 × 10^4^	7.11 × 10^3^	1.93 × 10^4^	1.59 × 10^4^	1.10 × 10^4^	1.44 × 10^4^	1.26 × 10^4^	1.88 × 10^4^	3.13 × 10^3^	3.73 × 10^4^	1.23 × 10^4^
	Std	1.71 × 10^3^	3.04 × 10^3^	2.93 × 10^3^	6.06 × 10^3^	5.40 × 10^3^	2.40 × 10^3^	2.09 × 10^3^	9.93 × 10^2^	2.00 × 10^3^	5.15 × 10^2^	8.55 × 10^3^	1.54 × 10^3^
	Best	9.06 × 10^2^	8.40 × 10^3^	3.34 × 10^3^	1.05 × 10^4^	8.46 × 10^3^	6.81 × 10^3^	1.07 × 10^4^	9.75 × 10^3^	1.63 × 10^4^	2.09 × 10^3^	2.43 × 10^4^	9.56 × 10^3^
F10	Mean	7.05 × 10^3^	6.99 × 10^3^	6.91 × 10^3^	9.79 × 10^3^	9.39 × 10^3^	8.12 × 10^3^	9.15 × 10^3^	8.45 × 10^3^	1.30 × 10^4^	5.85 × 10^3^	1.49 × 10^4^	8.65 × 10^3^
	Std	6.79 × 10^2^	7.38 × 10^2^	1.36 × 10^3^	1.16 × 10^3^	1.30 × 10^3^	9.65 × 10^2^	1.36 × 10^3^	7.56 × 10^2^	4.95 × 10^2^	3.45 × 10^2^	5.54 × 10^2^	1.36 × 10^3^
	Best	5.65 × 10^3^	5.45 × 10^3^	4.32 × 10^3^	7.01 × 10^3^	6.65 × 10^3^	5.86 × 10^3^	5.47 × 10^3^	7.10 × 10^3^	1.21 × 10^4^	5.00 × 10^3^	1.34 × 10^4^	6.18 × 10^3^
F11	Mean	1.22 × 10^3^	1.28 × 10^3^	4.54 × 10^3^	1.60 × 10^3^	1.61 × 10^3^	1.41 × 10^3^	1.45 × 10^3^	4.11 × 10^4^	1.49 × 10^3^	1.27 × 10^3^	8.95 × 10^3^	1.40 × 10^3^
	Std	3.68 × 10^1^	4.29 × 10^1^	1.80 × 10^3^	8.20 × 10^1^	1.17 × 10^2^	8.91 × 10^1^	1.01 × 10^2^	1.16 × 10^4^	1.77 × 10^2^	2.42 × 10^1^	2.60 × 10^3^	8.48 × 10^1^
	Best	1.17 × 10^3^	1.21 × 10^3^	1.92 × 10^3^	1.43 × 10^3^	1.38 × 10^3^	1.28 × 10^3^	1.29 × 10^3^	2.96 × 10^4^	1.30 × 10^3^	1.22 × 10^3^	5.49 × 10^3^	1.26 × 10^3^
F12	Mean	7.65 × 10^5^	9.45 × 10^7^	1.18 × 10^9^	2.38 × 10^8^	6.75 × 10^7^	3.80 × 10^5^	4.84 × 10^7^	1.00 × 10^6^	1.59 × 10^8^	5.76 × 10^6^	4.62 × 10^9^	5.21 × 10^5^
	Std	4.67 × 10^5^	4.07 × 10^8^	1.27 × 10^9^	1.23 × 10^8^	5.92 × 10^7^	1.48 × 10^5^	3.29 × 10^7^	8.11 × 10^5^	7.29 × 10^7^	1.83 × 10^6^	1.63 × 10^9^	4.59 × 10^5^
	Best	2.07 × 10^5^	3.29 × 10^5^	2.12 × 10^7^	4.26 × 10^7^	7.60 × 10^6^	1.03 × 10^5^	1.19 × 10^7^	1.45 × 10^5^	5.14 × 10^7^	2.22 × 10^6^	2.36 × 10^9^	3.89 × 10^4^
F13	Mean	7.96 × 10^3^	3.18 × 10^5^	1.97 × 10^8^	2.26 × 10^5^	5.67 × 10^5^	1.53 × 10^4^	1.69 × 10^5^	1.34 × 10^4^	5.94 × 10^6^	3.73 × 10^4^	8.81 × 10^8^	9.72 × 10^3^
	Std	6.48 × 10^3^	1.69 × 10^6^	2.58 × 10^8^	1.65 × 10^5^	4.52 × 10^5^	1.28 × 10^4^	1.12 × 10^5^	1.01 × 10^4^	4.10 × 10^6^	7.39 × 10^3^	5.66 × 10^8^	6.05 × 10^3^
	Best	2.19 × 10^3^	1.97 × 10^3^	4.75 × 10^4^	5.52 × 10^4^	7.38 × 10^4^	3.61 × 10^3^	1.95 × 10^4^	5.32 × 10^3^	1.21 × 10^6^	2.23 × 10^4^	2.66 × 10^8^	2.48 × 10^3^
F14	Mean	1.55 × 10^3^	6.19 × 10^4^	6.68 × 10^5^	6.37 × 10^5^	5.76 × 10^5^	1.27 × 10^4^	1.55 × 10^5^	3.11 × 10^4^	1.88 × 10^5^	7.44 × 10^4^	5.15 × 10^6^	4.52 × 10^4^
	Std	4.49 × 10^1^	5.16 × 10^4^	7.37 × 10^5^	5.69 × 10^5^	4.88 × 10^5^	1.09 × 10^4^	1.12 × 10^5^	2.04 × 10^4^	8.97 × 10^4^	4.50 × 10^4^	4.04 × 10^6^	1.19 × 10^5^
	Best	1.47 × 10^3^	3.64 × 10^3^	4.58 × 10^4^	1.57 × 10^4^	6.19 × 10^3^	2.89 × 10^3^	2.69 × 10^4^	6.32 × 10^3^	5.00 × 10^4^	9.00 × 10^3^	7.16 × 10^5^	2.76 × 10^3^
F15	Mean	1.78 × 10^3^	6.74 × 10^3^	1.15 × 10^7^	8.59 × 10^4^	1.50 × 10^7^	9.66 × 10^3^	6.12 × 10^4^	1.49 × 10^4^	2.58 × 10^4^	1.25 × 10^4^	1.32 × 10^8^	5.75 × 10^3^
	Std	1.42 × 10^2^	5.70 × 10^3^	1.54 × 10^7^	6.88 × 10^4^	4.63 × 10^7^	6.84 × 10^3^	5.59 × 10^4^	6.54 × 10^3^	4.65 × 10^3^	3.21 × 10^3^	6.86 × 10^7^	4.41 × 10^3^
	Best	1.61 × 10^3^	1.83 × 10^3^	1.70 × 10^4^	1.30 × 10^4^	4.14 × 10^4^	1.90 × 10^3^	1.21 × 10^4^	3.02 × 10^3^	1.33 × 10^4^	5.36 × 10^3^	4.14 × 10^7^	1.88 × 10^3^
F16	Mean	2.70 × 10^3^	3.47 × 10^3^	2.93 × 10^3^	4.92 × 10^3^	4.26 × 10^3^	3.47 × 10^3^	4.11 × 10^3^	3.78 × 10^3^	3.23 × 10^3^	2.76 × 10^3^	6.03 × 10^3^	3.64 × 10^3^
	Std	3.30 × 10^2^	4.68 × 10^2^	3.15 × 10^2^	6.45 × 10^2^	4.69 × 10^2^	3.93 × 10^2^	5.76 × 10^2^	4.84 × 10^2^	4.04 × 10^2^	2.36 × 10^2^	4.35 × 10^2^	5.59 × 10^2^
	Best	2.05 × 10^3^	2.49 × 10^3^	2.40 × 10^3^	3.82 × 10^3^	3.10 × 10^3^	2.52 × 10^3^	2.85 × 10^3^	2.72 × 10^3^	2.60 × 10^3^	2.30 × 10^3^	4.79 × 10^3^	2.62 × 10^3^
F17	Mean	2.52 × 10^3^	3.24 × 10^3^	2.66 × 10^3^	4.11 × 10^3^	3.69 × 10^3^	3.61 × 10^3^	3.66 × 10^3^	3.62 × 10^3^	3.12 × 10^3^	2.57 × 10^3^	4.66 × 10^3^	3.56 × 10^3^
	Std	2.54 × 10^2^	3.31 × 10^2^	2.84 × 10^2^	4.63 × 10^2^	3.84 × 10^2^	5.20 × 10^2^	4.10 × 10^2^	4.46 × 10^2^	3.73 × 10^2^	9.61 × 10^1^	4.28 × 10^2^	3.79 × 10^2^
	Best	2.12 × 10^3^	2.45 × 10^3^	2.17 × 10^3^	3.03 × 10^3^	2.86 × 10^3^	2.80 × 10^3^	2.74 × 10^3^	2.66 × 10^3^	2.61 × 10^3^	2.38 × 10^3^	3.53 × 10^3^	2.57 × 10^3^
F18	Mean	4.81 × 10^3^	5.76 × 10^5^	5.06 × 10^6^	4.91 × 10^6^	2.79 × 10^6^	5.82 × 10^4^	2.54 × 10^6^	1.07 × 10^5^	1.66 × 10^6^	5.63 × 10^5^	2.50 × 10^7^	8.94 × 10^4^
	Std	2.34 × 10^3^	2.43 × 10^5^	5.17 × 10^6^	3.65 × 10^6^	2.25 × 10^6^	2.73 × 10^4^	1.91 × 10^6^	3.54 × 10^4^	8.98 × 10^5^	3.17 × 10^5^	1.41 × 10^7^	1.35 × 10^5^
	Best	2.23 × 10^3^	1.24 × 10^5^	1.44 × 10^5^	8.04 × 10^5^	1.84 × 10^5^	1.55 × 10^4^	2.91 × 10^5^	4.52 × 10^4^	2.87 × 10^5^	1.10 × 10^5^	3.52 × 10^6^	8.39 × 10^3^
F19	Mean	2.09 × 10^3^	1.55 × 10^4^	7.57 × 10^6^	1.97 × 10^6^	7.17 × 10^5^	1.80 × 10^4^	6.49 × 10^5^	2.01 × 10^4^	3.19 × 10^4^	7.53 × 10^3^	5.99 × 10^7^	1.64 × 10^4^
	Std	2.17 × 10^2^	9.13 × 10^3^	2.34 × 10^7^	1.87 × 10^6^	9.13 × 10^5^	1.08 × 10^4^	1.34 × 10^6^	1.02 × 10^4^	1.63 × 10^4^	2.85 × 10^3^	2.41 × 10^7^	8.38 × 10^3^
	Best	1.98 × 10^3^	2.10 × 10^3^	1.90 × 10^5^	1.16 × 10^5^	4.85 × 10^3^	2.28 × 10^3^	4.14 × 10^4^	6.57 × 10^3^	9.83 × 10^3^	3.08 × 10^3^	1.97 × 10^7^	3.65 × 10^3^
F20	Mean	2.59 × 10^3^	3.08 × 10^3^	2.81 × 10^3^	3.57 × 10^3^	3.46 × 10^3^	3.44 × 10^3^	3.37 × 10^3^	3.51 × 10^3^	3.20 × 10^3^	2.64 × 10^3^	4.23 × 10^3^	3.51 × 10^3^
	Std	3.49 × 10^2^	3.50 × 10^2^	2.19 × 10^2^	3.97 × 10^2^	3.79 × 10^2^	3.15 × 10^2^	3.19 × 10^2^	3.21 × 10^2^	1.49 × 10^2^	1.62 × 10^2^	3.63 × 10^2^	3.62 × 10^2^
	Best	2.12 × 10^3^	2.34 × 10^3^	2.42 × 10^3^	2.90 × 10^3^	2.83 × 10^3^	2.78 × 10^3^	2.82 × 10^3^	2.80 × 10^3^	2.98 × 10^3^	2.48 × 10^3^	3.30 × 10^3^	2.93 × 10^3^
F21	Mean	2.47 × 10^3^	2.68 × 10^3^	2.51 × 10^3^	2.87 × 10^3^	2.77 × 10^3^	2.64 × 10^3^	2.80 × 10^3^	2.55 × 10^3^	2.69 × 10^3^	2.48 × 10^3^	2.94 × 10^3^	2.75 × 10^3^
	Std	3.54 × 10^1^	5.81 × 10^1^	4.84 × 10^1^	1.32 × 10^2^	1.19 × 10^2^	6.56 × 10^1^	1.08 × 10^2^	8.83 × 10^1^	3.93 × 10^1^	1.27 × 10^1^	7.20 × 10^1^	5.96 × 10^1^
	Best	2.43 × 10^3^	2.60 × 10^3^	2.45 × 10^3^	2.61 × 10^3^	2.53 × 10^3^	2.54 × 10^3^	2.64 × 10^3^	2.43 × 10^3^	2.61 × 10^3^	2.47 × 10^3^	2.82 × 10^3^	2.66 × 10^3^
F22	Mean	7.32 × 10^3^	8.84 × 10^3^	8.43 × 10^3^	1.16 × 10^4^	1.09 × 10^4^	9.99 × 10^3^	1.11 × 10^4^	1.03 × 10^4^	1.49 × 10^4^	6.83 × 10^3^	1.66 × 10^4^	1.08 × 10^4^
	Std	2.70 × 10^3^	9.03 × 10^2^	1.01 × 10^3^	1.24 × 10^3^	1.76 × 10^3^	1.02 × 10^3^	1.37 × 10^3^	6.83 × 10^2^	1.38 × 10^3^	1.54 × 10^3^	3.75 × 10^2^	1.54 × 10^3^
	Best	2.33 × 10^3^	6.75 × 10^3^	6.29 × 10^3^	9.50 × 10^3^	8.06 × 10^3^	7.92 × 10^3^	8.63 × 10^3^	8.32 × 10^3^	1.11 × 10^4^	2.35 × 10^3^	1.58 × 10^4^	8.50 × 10^3^
F23	Mean	2.90 × 10^3^	3.57 × 10^3^	2.96 × 10^3^	3.61 × 10^3^	3.26 × 10^3^	3.35 × 10^3^	3.67 × 10^3^	3.11 × 10^3^	3.16 × 10^3^	2.85 × 10^3^	3.47 × 10^3^	3.61 × 10^3^
	Std	3.64 × 10^1^	2.00 × 10^2^	5.90 × 10^1^	1.80 × 10^2^	1.84 × 10^2^	1.34 × 10^2^	2.39 × 10^2^	1.64 × 10^2^	6.89 × 10^1^	1.30 × 10^1^	8.96 × 10^1^	1.88 × 10^2^
	Best	2.83 × 10^3^	3.21 × 10^3^	2.89 × 10^3^	3.28 × 10^3^	2.98 × 10^3^	3.07 × 10^3^	3.35 × 10^3^	2.95 × 10^3^	3.06 × 10^3^	2.83 × 10^3^	3.22 × 10^3^	3.23 × 10^3^
F24	Mean	3.06 × 10^3^	3.56 × 10^3^	3.15 × 10^3^	3.65 × 10^3^	3.43 × 10^3^	3.54 × 10^3^	3.87 × 10^3^	3.27 × 10^3^	3.72 × 10^3^	3.01 × 10^3^	3.58 × 10^3^	4.16 × 10^3^
	Std	4.05 × 10^1^	1.27 × 10^2^	9.81 × 10^1^	1.47 × 10^2^	1.36 × 10^2^	1.79 × 10^2^	2.02 × 10^2^	1.90 × 10^2^	9.59 × 10^1^	1.32 × 10^1^	7.50 × 10^1^	3.93 × 10^2^
	Best	3.00 × 10^3^	3.34 × 10^3^	3.02 × 10^3^	3.34 × 10^3^	3.17 × 10^3^	3.22 × 10^3^	3.46 × 10^3^	3.04 × 10^3^	3.51 × 10^3^	2.97 × 10^3^	3.43 × 10^3^	3.43 × 10^3^
F25	Mean	2.97 × 10^3^	3.07 × 10^3^	3.70 × 10^3^	3.13 × 10^3^	3.20 × 10^3^	3.07 × 10^3^	3.12 × 10^3^	3.09 × 10^3^	3.13 × 10^3^	3.02 × 10^3^	5.64 × 10^3^	3.05 × 10^3^
	Std	9.67 × 10^0^	3.06 × 10^1^	3.73 × 10^2^	4.54 × 10^1^	6.29 × 10^2^	3.02 × 10^1^	3.29 × 10^1^	2.96 × 10^1^	3.99 × 10^1^	1.06 × 10^1^	7.91 × 10^2^	3.16 × 10^1^
	Best	2.96 × 10^3^	2.96 × 10^3^	3.32 × 10^3^	3.03 × 10^3^	3.01 × 10^3^	2.98 × 10^3^	3.05 × 10^3^	2.98 × 10^3^	3.03 × 10^3^	3.00 × 10^3^	4.23 × 10^3^	2.96 × 10^3^
F26	Mean	5.05 × 10^3^	7.60 × 10^3^	6.27 × 10^3^	1.30 × 10^4^	8.47 × 10^3^	1.02 × 10^4^	1.23 × 10^4^	1.08 × 10^4^	3.93 × 10^3^	5.15 × 10^3^	1.18 × 10^4^	9.80 × 10^3^
	Std	8.09 × 10^2^	3.43 × 10^3^	5.20 × 10^2^	1.27 × 10^3^	1.20 × 10^3^	2.16 × 10^3^	1.02 × 10^3^	2.68 × 10^3^	2.46 × 10^3^	1.39 × 10^2^	9.16 × 10^2^	3.75 × 10^3^
	Best	2.93 × 10^3^	3.05 × 10^3^	5.10 × 10^3^	1.02 × 10^4^	6.38 × 10^3^	2.90 × 10^3^	9.81 × 10^3^	2.90 × 10^3^	2.94 × 10^3^	4.89 × 10^3^	1.02 × 10^4^	2.90 × 10^3^
F27	Mean	3.27 × 10^3^	4.03 × 10^3^	3.56 × 10^3^	4.24 × 10^3^	3.89 × 10^3^	3.83 × 10^3^	4.03 × 10^3^	4.01 × 10^3^	3.60 × 10^3^	3.27 × 10^3^	3.98 × 10^3^	4.01 × 10^3^
	Std	3.81 × 10^1^	4.52 × 10^2^	7.14 × 10^1^	4.27 × 10^2^	2.21 × 10^2^	2.51 × 10^2^	3.32 × 10^2^	3.39 × 10^2^	1.02 × 10^2^	1.55 × 10^1^	1.05 × 10^2^	5.86 × 10^2^
	Best	3.22 × 10^3^	3.46 × 10^3^	3.43 × 10^3^	3.63 × 10^3^	3.53 × 10^3^	3.48 × 10^3^	3.58 × 10^3^	3.57 × 10^3^	3.42 × 10^3^	3.25 × 10^3^	3.75 × 10^3^	3.44 × 10^3^
F28	Mean	3.28 × 10^3^	3.37 × 10^3^	4.30 × 10^3^	3.44 × 10^3^	5.09 × 10^3^	3.29 × 10^3^	3.37 × 10^3^	3.30 × 10^3^	3.53 × 10^3^	3.27 × 10^3^	5.87 × 10^3^	3.30 × 10^3^
	Std	2.68 × 10^1^	1.36 × 10^2^	4.17 × 10^2^	5.29 × 10^1^	1.83 × 10^3^	1.59 × 10^1^	2.29 × 10^2^	2.02 × 10^1^	7.20 × 10^1^	8.85 × 10^0^	6.84 × 10^2^	2.59 × 10^1^
	Best	3.26 × 10^3^	3.26 × 10^3^	3.65 × 10^3^	3.36 × 10^3^	3.30 × 10^3^	3.26 × 10^3^	3.27 × 10^3^	3.26 × 10^3^	3.39 × 10^3^	3.26 × 10^3^	4.72 × 10^3^	3.26 × 10^3^
F29	Mean	3.80 × 10^3^	4.74 × 10^3^	4.40 × 10^3^	6.95 × 10^3^	5.48 × 10^3^	5.39 × 10^3^	6.75 × 10^3^	5.33 × 10^3^	4.32 × 10^3^	3.62 × 10^3^	7.13 × 10^3^	5.30 × 10^3^
	Std	2.16 × 10^2^	3.39 × 10^2^	2.36 × 10^2^	7.70 × 10^2^	5.60 × 10^2^	4.48 × 10^2^	5.61 × 10^2^	3.74 × 10^2^	3.58 × 10^2^	1.01 × 10^2^	5.29 × 10^2^	9.47 × 10^2^
	Best	3.38 × 10^3^	4.20 × 10^3^	3.98 × 10^3^	5.40 × 10^3^	4.40 × 10^3^	4.54 × 10^3^	5.59 × 10^3^	4.51 × 10^3^	3.85 × 10^3^	3.38 × 10^3^	5.86 × 10^3^	4.31 × 10^3^
F30	Mean	7.69 × 10^5^	9.43 × 10^5^	8.40 × 10^7^	1.01 × 10^8^	1.37 × 10^7^	1.03 × 10^6^	3.89 × 10^7^	9.51 × 10^5^	3.87 × 10^6^	1.43 × 10^6^	3.19 × 10^8^	1.06 × 10^6^
	Std	1.09 × 10^5^	5.65 × 10^5^	2.41 × 10^7^	3.81 × 10^7^	8.23 × 10^6^	2.31 × 10^5^	1.30 × 10^7^	2.27 × 10^5^	1.12 × 10^6^	2.06 × 10^5^	1.11 × 10^8^	2.92 × 10^5^
	Best	5.94 × 10^5^	6.06 × 10^5^	5.10 × 10^7^	2.00 × 10^7^	2.12 × 10^6^	6.85 × 10^5^	1.40 × 10^7^	6.63 × 10^5^	2.22 × 10^6^	9.97 × 10^5^	1.38 × 10^8^	7.38 × 10^5^

**Table A3:** Experimental results of 12 algorithms in CEC 2017 (Dim = 100).

Algorithm	Metric	SSMO	PSO	GWO	WOA	DBO	GWCA	SGA	IVY	FATA	PLO	SFOA	EMWPA
F1	Mean	3.08 × 10^6^	2.14 × 10^9^	4.50 × 10^10^	4.02 × 10^7^	5.47 × 10^9^	1.70 × 10^4^	5.82 × 10^8^	4.53 × 10^3^	2.14 × 10^9^	7.81 × 10^7^	9.56 × 10^10^	8.87 × 10^6^
	Std	5.90 × 10^5^	2.93 × 10^9^	8.96 × 10^9^	1.60 × 10^7^	2.98 × 10^10^	1.69 × 10^4^	4.58 × 10^8^	4.39 × 10^3^	6.00 × 10^8^	2.24 × 10^7^	1.57 × 10^10^	7.42 × 10^6^
	Best	1.78 × 10^6^	1.60 × 10^3^	2.68 × 10^10^	1.46 × 10^7^	3.50 × 10^4^	1.22 × 10^2^	3.80 × 10^7^	4.24 × 10^2^	1.39 × 10^9^	2.29 × 10^7^	7.62 × 10^10^	4.57 × 10^5^
F3	Mean	6.95 × 10^2^	2.26 × 10^3^	2.10 × 10^5^	5.33 × 10^5^	2.29 × 10^5^	3.11 × 10^2^	1.30 × 10^5^	1.32 × 10^5^	2.58 × 10^5^	4.20 × 10^5^	5.19 × 10^5^	8.92 × 10^4^
	Std	9.56 × 10^1^	2.37 × 10^3^	1.98 × 10^4^	1.58 × 10^5^	3.43 × 10^4^	1.08 × 10^1^	1.94 × 10^4^	4.97 × 10^4^	2.83 × 10^4^	3.71 × 10^4^	1.35 × 10^5^	4.05 × 10^4^
	Best	6.01 × 10^2^	9.67 × 10^2^	1.78 × 10^5^	2.20 × 10^5^	1.56 × 10^5^	3.00 × 10^2^	8.79 × 10^4^	4.21 × 10^4^	2.10 × 10^5^	3.55 × 10^5^	3.62 × 10^5^	2.62 × 10^4^
F4	Mean	6.40 × 10^2^	8.60 × 10^2^	4.70 × 10^3^	9.97 × 10^2^	2.13 × 10^3^	6.45 × 10^2^	1.07 × 10^3^	5.87 × 10^2^	1.04 × 10^3^	7.09 × 10^2^	1.32 × 10^4^	7.29 × 10^2^
	Std	2.95 × 10^1^	2.29 × 10^2^	1.52 × 10^3^	9.22 × 10^1^	5.22 × 10^3^	4.50 × 10^1^	1.39 × 10^2^	3.27 × 10^1^	6.55 × 10^1^	2.14 × 10^1^	2.24 × 10^3^	3.31 × 10^1^
	Best	5.97 × 10^2^	6.42 × 10^2^	2.89 × 10^3^	8.23 × 10^2^	7.01 × 10^2^	5.66 × 10^2^	8.63 × 10^2^	5.51 × 10^2^	8.88 × 10^2^	6.68 × 10^2^	1.01 × 10^4^	6.71 × 10^2^
F5	Mean	1.10 × 10^3^	1.19 × 10^3^	1.13 × 10^3^	1.47 × 10^3^	1.64 × 10^3^	1.35 × 10^3^	1.39 × 10^3^	1.35 × 10^3^	1.49 × 10^3^	1.03 × 10^3^	1.95 × 10^3^	1.40 × 10^3^
	Std	5.60 × 10^1^	6.74 × 10^1^	6.76 × 10^1^	9.87 × 10^1^	1.94 × 10^2^	9.94 × 10^1^	5.75 × 10^1^	4.95 × 10^1^	3.64 × 10^1^	3.65 × 10^1^	7.05 × 10^1^	1.04 × 10^2^
	Best	9.68 × 10^2^	1.05 × 10^3^	9.84 × 10^2^	1.29 × 10^3^	1.30 × 10^3^	1.16 × 10^3^	1.26 × 10^3^	1.20 × 10^3^	1.43 × 10^3^	9.62 × 10^2^	1.83 × 10^3^	1.23 × 10^3^
F6	Mean	6.18 × 10^2^	6.55 × 10^2^	6.35 × 10^2^	6.78 × 10^2^	6.74 × 10^2^	6.59 × 10^2^	6.72 × 10^2^	6.53 × 10^2^	6.67 × 10^2^	6.27 × 10^2^	6.99 × 10^2^	6.60 × 10^2^
	Std	6.03 × 10^0^	3.98 × 10^0^	4.18 × 10^0^	8.13 × 10^0^	1.19 × 10^1^	4.68 × 10^0^	4.48 × 10^0^	9.44 × 10^0^	2.84 × 10^0^	1.98 × 10^0^	8.34 × 10^0^	4.11 × 10^0^
	Best	6.09 × 10^2^	6.47 × 10^2^	6.28 × 10^2^	6.68 × 10^2^	6.54 × 10^2^	6.50 × 10^2^	6.64 × 10^2^	6.28 × 10^2^	6.62 × 10^2^	6.23 × 10^2^	6.73 × 10^2^	6.55 × 10^2^
F7	Mean	1.09 × 10^3^	1.23 × 10^3^	1.96 × 10^3^	3.27 × 10^3^	2.73 × 10^3^	4.00 × 10^3^	3.23 × 10^3^	3.22 × 10^3^	2.68 × 10^3^	2.00 × 10^3^	3.43 × 10^3^	3.56 × 10^3^
	Std	3.91 × 10^1^	8.94 × 10^1^	1.29 × 10^2^	1.73 × 10^2^	2.37 × 10^2^	4.43 × 10^2^	1.53 × 10^2^	1.01 × 10^2^	2.00 × 10^2^	6.58 × 10^1^	1.72 × 10^2^	3.19 × 10^2^
	Best	1.01 × 10^3^	1.06 × 10^3^	1.73 × 10^3^	2.81 × 10^3^	2.15 × 10^3^	2.76 × 10^3^	2.85 × 10^3^	2.92 × 10^3^	2.24 × 10^3^	1.87 × 10^3^	3.13 × 10^3^	2.82 × 10^3^
F8	Mean	1.43 × 10^3^	1.61 × 10^3^	1.44 × 10^3^	1.88 × 10^3^	1.97 × 10^3^	1.74 × 10^3^	1.84 × 10^3^	1.80 × 10^3^	1.93 × 10^3^	1.30 × 10^3^	2.30 × 10^3^	1.76 × 10^3^
	Std	8.53 × 10^1^	6.79 × 10^1^	6.94 × 10^1^	9.47 × 10^1^	2.11 × 10^2^	1.06 × 10^2^	6.60 × 10^1^	7.92 × 10^1^	4.76 × 10^1^	3.41 × 10^1^	8.32 × 10^1^	1.07 × 10^2^
	Best	1.33 × 10^3^	1.49 × 10^3^	1.28 × 10^3^	1.72 × 10^3^	1.56 × 10^3^	1.53 × 10^3^	1.69 × 10^3^	1.66 × 10^3^	1.83 × 10^3^	1.23 × 10^3^	2.13 × 10^3^	1.59 × 10^3^
F9	Mean	7.64 × 10^3^	3.49 × 10^4^	2.44 × 10^4^	3.91 × 10^4^	3.06 × 10^4^	2.39 × 10^4^	2.80 × 10^4^	2.30 × 10^4^	4.21 × 10^4^	1.69 × 10^4^	8.39 × 10^4^	2.52 × 10^4^
	Std	1.30 × 10^4^	9.83 × 10^3^	9.93 × 10^3^	9.99 × 10^3^	1.16 × 10^4^	3.19 × 10^3^	2.50 × 10^3^	5.49 × 10^2^	5.06 × 10^3^	2.00 × 10^3^	9.80 × 10^3^	1.68 × 10^3^
	Best	1.08 × 10^3^	2.19 × 10^4^	1.06 × 10^4^	2.36 × 10^4^	1.93 × 10^4^	1.63 × 10^4^	2.30 × 10^4^	2.14 × 10^4^	3.37 × 10^4^	1.30 × 10^4^	6.80 × 10^4^	2.25 × 10^4^
F10	Mean	1.57 × 10^4^	1.34 × 10^4^	1.49 × 10^4^	2.15 × 10^4^	1.92 × 10^4^	1.64 × 10^4^	2.07 × 10^4^	1.63 × 10^4^	3.05 × 10^4^	1.63 × 10^4^	3.24 × 10^4^	1.67 × 10^4^
	Std	1.20 × 10^3^	1.13 × 10^3^	1.44 × 10^3^	2.61 × 10^3^	2.98 × 10^3^	1.24 × 10^3^	2.16 × 10^3^	1.42 × 10^3^	5.80 × 10^2^	5.49 × 10^2^	5.55 × 10^2^	1.04 × 10^3^
	Best	1.38 × 10^4^	1.17 × 10^4^	1.17 × 10^4^	1.71 × 10^4^	1.40 × 10^4^	1.35 × 10^4^	1.74 × 10^4^	1.42 × 10^4^	2.87 × 10^4^	1.49 × 10^4^	3.10 × 10^4^	1.51 × 10^4^
F11	Mean	2.12 × 10^3^	2.28 × 10^3^	5.08 × 10^4^	1.14 × 10^4^	1.89 × 10^4^	2.60 × 10^3^	5.73 × 10^3^	2.32 × 10^3^	3.93 × 10^3^	5.23 × 10^3^	1.39 × 10^5^	6.20 × 10^3^
	Std	1.18 × 10^2^	4.57 × 10^2^	1.26 × 10^4^	1.08 × 10^4^	1.99 × 10^4^	3.79 × 10^2^	8.83 × 10^2^	3.23 × 10^2^	8.14 × 10^2^	7.40 × 10^2^	3.57 × 10^4^	9.66 × 10^3^
	Best	1.87 × 10^3^	1.82 × 10^3^	2.89 × 10^4^	5.20 × 10^3^	2.56 × 10^3^	2.03 × 10^3^	3.43 × 10^3^	1.70 × 10^3^	2.67 × 10^3^	3.53 × 10^3^	5.67 × 10^4^	2.55 × 10^3^
F12	Mean	1.03 × 10^7^	1.59 × 10^9^	8.77 × 10^9^	6.63 × 10^8^	4.25 × 10^8^	1.44 × 10^6^	3.56 × 10^8^	1.27 × 10^6^	8.75 × 10^8^	5.18 × 10^7^	1.98 × 10^10^	4.22 × 10^6^
	Std	2.80 × 10^6^	1.75 × 10^9^	4.44 × 10^9^	2.45 × 10^8^	2.40 × 10^8^	6.44 × 10^5^	1.60 × 10^8^	4.08 × 10^5^	2.37 × 10^8^	1.09 × 10^7^	4.01 × 10^9^	4.09 × 10^6^
	Best	6.44 × 10^6^	1.35 × 10^7^	2.14 × 10^9^	1.77 × 10^8^	8.20 × 10^7^	4.48 × 10^5^	4.00 × 10^7^	5.87 × 10^5^	5.04 × 10^8^	3.03 × 10^7^	1.42 × 10^10^	1.46 × 10^6^
F13	Mean	1.44 × 10^4^	4.97 × 10^7^	1.58 × 10^9^	1.07 × 10^5^	6.07 × 10^6^	1.45 × 10^4^	8.48 × 10^4^	1.13 × 10^4^	1.66 × 10^7^	7.13 × 10^4^	2.10 × 10^9^	1.02 × 10^4^
	Std	7.87 × 10^3^	2.05 × 10^8^	1.91 × 10^9^	4.57 × 10^4^	1.02 × 10^7^	6.83 × 10^3^	3.39 × 10^4^	5.42 × 10^3^	1.46 × 10^7^	7.54 × 10^3^	5.36 × 10^8^	4.72 × 10^3^
	Best	6.22 × 10^3^	5.44 × 10^3^	7.52 × 10^5^	4.20 × 10^4^	4.49 × 10^4^	7.07 × 10^3^	3.28 × 10^4^	5.85 × 10^3^	3.23 × 10^6^	6.07 × 10^4^	1.13 × 10^9^	6.95 × 10^3^
F14	Mean	5.61 × 10^4^	4.06 × 10^5^	4.09 × 10^6^	1.74 × 10^6^	3.57 × 10^6^	9.78 × 10^4^	1.29 × 10^6^	7.00 × 10^4^	1.02 × 10^6^	7.59 × 10^5^	2.00 × 10^7^	6.32 × 10^4^
	Std	2.06 × 10^4^	1.53 × 10^5^	2.39 × 10^6^	1.03 × 10^6^	2.76 × 10^6^	1.77 × 10^5^	6.22 × 10^5^	2.54 × 10^4^	4.19 × 10^5^	2.47 × 10^5^	6.14 × 10^6^	2.30 × 10^4^
	Best	1.95 × 10^4^	1.18 × 10^5^	8.99 × 10^5^	5.28 × 10^5^	2.82 × 10^5^	9.56 × 10^3^	5.75 × 10^5^	3.16 × 10^4^	4.25 × 10^5^	2.59 × 10^5^	5.14 × 10^6^	2.57 × 10^4^
F15	Mean	4.22 × 10^3^	4.07 × 10^3^	1.99 × 10^8^	2.35 × 10^5^	1.42 × 10^6^	4.47 × 10^3^	6.62 × 10^4^	3.51 × 10^3^	7.65 × 10^5^	2.86 × 10^4^	5.29 × 10^8^	3.26 × 10^3^
	Std	3.03 × 10^3^	2.43 × 10^3^	2.90 × 10^8^	8.59 × 10^5^	2.33 × 10^6^	3.46 × 10^3^	3.13 × 10^4^	1.90 × 10^3^	6.31 × 10^5^	6.22 × 10^3^	1.37 × 10^8^	1.53 × 10^3^
	Best	2.10 × 10^3^	1.94 × 10^3^	3.44 × 10^4^	3.19 × 10^4^	5.70 × 10^4^	2.10 × 10^3^	2.06 × 10^4^	1.92 × 10^3^	2.67 × 10^5^	1.98 × 10^4^	2.86 × 10^8^	1.99 × 10^3^
F16	Mean	4.55 × 10^3^	5.11 × 10^3^	5.76 × 10^3^	1.02 × 10^4^	7.80 × 10^3^	5.80 × 10^3^	7.93 × 10^3^	6.16 × 10^3^	5.92 × 10^3^	4.56 × 10^3^	1.28 × 10^4^	6.09 × 10^3^
	Std	6.40 × 10^2^	7.08 × 10^2^	5.86 × 10^2^	2.45 × 10^3^	1.41 × 10^3^	6.52 × 10^2^	1.03 × 10^3^	9.35 × 10^2^	6.60 × 10^2^	3.54 × 10^2^	7.27 × 10^2^	6.94 × 10^2^
	Best	3.73 × 10^3^	3.55 × 10^3^	4.60 × 10^3^	6.98 × 10^3^	3.90 × 10^3^	4.63 × 10^3^	6.03 × 10^3^	4.40 × 10^3^	4.61 × 10^3^	3.96 × 10^3^	1.12 × 10^4^	4.77 × 10^3^
F17	Mean	4.15 × 10^3^	4.79 × 10^3^	4.79 × 10^3^	6.96 × 10^3^	6.74 × 10^3^	6.80 × 10^3^	7.07 × 10^3^	5.35 × 10^3^	5.16 × 10^3^	4.16 × 10^3^	9.73 × 10^3^	5.88 × 10^3^
	Std	5.04 × 10^2^	6.91 × 10^2^	6.26 × 10^2^	9.42 × 10^2^	7.26 × 10^2^	8.29 × 10^2^	7.37 × 10^2^	5.06 × 10^2^	5.03 × 10^2^	2.17 × 10^2^	9.64 × 10^2^	7.73 × 10^2^
	Best	3.18 × 10^3^	3.50 × 10^3^	3.76 × 10^3^	5.34 × 10^3^	5.30 × 10^3^	5.35 × 10^3^	5.47 × 10^3^	4.15 × 10^3^	4.16 × 10^3^	3.80 × 10^3^	7.55 × 10^3^	5.00 × 10^3^
F18	Mean	1.34 × 10^5^	1.21 × 10^6^	4.24 × 10^6^	1.91 × 10^6^	3.78 × 10^6^	1.46 × 10^5^	2.49 × 10^6^	1.51 × 10^5^	2.88 × 10^6^	1.74 × 10^6^	2.71 × 10^7^	1.54 × 10^5^
	Std	6.39 × 10^4^	5.70 × 10^5^	2.31 × 10^6^	1.02 × 10^6^	2.08 × 10^6^	7.05 × 10^4^	6.36 × 10^5^	3.84 × 10^4^	1.09 × 10^6^	6.05 × 10^5^	1.14 × 10^7^	7.82 × 10^4^
	Best	6.65 × 10^4^	3.08 × 10^5^	1.09 × 10^6^	5.70 × 10^5^	2.32 × 10^5^	3.66 × 10^4^	1.21 × 10^6^	8.86 × 10^4^	1.24 × 10^6^	9.54 × 10^5^	6.31 × 10^6^	3.42 × 10^4^
F19	Mean	6.76 × 10^3^	6.09 × 10^3^	1.71 × 10^8^	1.61 × 10^7^	3.39 × 10^6^	7.15 × 10^3^	5.12 × 10^6^	5.93 × 10^3^	1.31 × 10^6^	2.67 × 10^4^	5.50 × 10^8^	8.10 × 10^3^
	Std	5.27 × 10^3^	6.81 × 10^3^	2.37 × 10^8^	4.96 × 10^6^	3.45 × 10^6^	7.65 × 10^3^	2.81 × 10^6^	2.37 × 10^3^	4.27 × 10^5^	8.90 × 10^3^	1.58 × 10^8^	1.06 × 10^4^
	Best	2.88 × 10^3^	2.13 × 10^3^	6.84 × 10^5^	5.31 × 10^6^	1.26 × 10^5^	2.61 × 10^3^	8.26 × 10^5^	2.89 × 10^3^	4.78 × 10^5^	1.11 × 10^4^	2.85 × 10^8^	2.41 × 10^3^
F20	Mean	4.46 × 10^3^	4.86 × 10^3^	4.52 × 10^3^	6.39 × 10^3^	5.86 × 10^3^	5.67 × 10^3^	5.81 × 10^3^	5.59 × 10^3^	6.51 × 10^3^	4.21 × 10^3^	7.87 × 10^3^	5.73 × 10^3^
	Std	4.43 × 10^2^	4.89 × 10^2^	4.03 × 10^2^	5.78 × 10^2^	6.23 × 10^2^	4.91 × 10^2^	6.81 × 10^2^	4.55 × 10^2^	3.24 × 10^2^	2.44 × 10^2^	3.97 × 10^2^	8.82 × 10^2^
	Best	3.80 × 10^3^	3.26 × 10^3^	4.10 × 10^3^	5.33 × 10^3^	4.49 × 10^3^	4.65 × 10^3^	4.58 × 10^3^	4.32 × 10^3^	5.88 × 10^3^	3.67 × 10^3^	6.83 × 10^3^	4.84 × 10^3^
F21	Mean	2.97 × 10^3^	3.42 × 10^3^	2.91 × 10^3^	3.98 × 10^3^	3.47 × 10^3^	3.38 × 10^3^	3.89 × 10^3^	3.05 × 10^3^	3.49 × 10^3^	2.86 × 10^3^	3.90 × 10^3^	3.82 × 10^3^
	Std	1.13 × 10^2^	1.67 × 10^2^	6.21 × 10^1^	2.68 × 10^2^	3.27 × 10^2^	1.58 × 10^2^	2.33 × 10^2^	2.53 × 10^2^	6.52 × 10^1^	4.36 × 10^1^	1.20 × 10^2^	3.02 × 10^2^
	Best	2.83 × 10^3^	3.09 × 10^3^	2.76 × 10^3^	3.40 × 10^3^	2.94 × 10^3^	3.06 × 10^3^	3.47 × 10^3^	2.73 × 10^3^	3.37 × 10^3^	2.73 × 10^3^	3.59 × 10^3^	3.40 × 10^3^
F22	Mean	1.80 × 10^4^	1.76 × 10^4^	1.85 × 10^4^	2.50 × 10^4^	2.37 × 10^4^	1.96 × 10^4^	2.41 × 10^4^	2.05 × 10^4^	3.23 × 10^4^	1.85 × 10^4^	3.43 × 10^4^	2.04 × 10^4^
	Std	1.37 × 10^3^	1.50 × 10^3^	3.15 × 10^3^	3.16 × 10^3^	4.05 × 10^3^	1.63 × 10^3^	2.92 × 10^3^	1.61 × 10^3^	6.39 × 10^2^	5.73 × 10^2^	6.26 × 10^2^	2.01 × 10^3^
	Best	1.67 × 10^4^	1.25 × 10^4^	1.43 × 10^4^	2.07 × 10^4^	1.75 × 10^4^	1.68 × 10^4^	1.91 × 10^4^	1.78 × 10^4^	3.12 × 10^4^	1.77 × 10^4^	3.19 × 10^4^	1.70 × 10^4^
F23	Mean	3.42 × 10^3^	4.87 × 10^3^	3.50 × 10^3^	4.69 × 10^3^	4.27 × 10^3^	4.51 × 10^3^	5.07 × 10^3^	3.65 × 10^3^	3.71 × 10^3^	3.24 × 10^3^	4.51 × 10^3^	5.33 × 10^3^
	Std	1.02 × 10^2^	2.90 × 10^2^	8.72 × 10^1^	2.96 × 10^2^	3.40 × 10^2^	2.74 × 10^2^	2.91 × 10^2^	1.74 × 10^2^	9.72 × 10^1^	2.58 × 10^1^	1.71 × 10^2^	5.62 × 10^2^
	Best	3.32 × 10^3^	4.28 × 10^3^	3.33 × 10^3^	4.17 × 10^3^	3.70 × 10^3^	4.09 × 10^3^	4.44 × 10^3^	3.39 × 10^3^	3.44 × 10^3^	3.17 × 10^3^	4.27 × 10^3^	4.67 × 10^3^
F24	Mean	3.85 × 10^3^	5.22 × 10^3^	4.17 × 10^3^	5.97 × 10^3^	4.76 × 10^3^	5.52 × 10^3^	6.56 × 10^3^	4.59 × 10^3^	4.92 × 10^3^	3.74 × 10^3^	5.22 × 10^3^	7.90 × 10^3^
	Std	7.87 × 10^1^	3.38 × 10^2^	1.16 × 10^2^	4.54 × 10^2^	4.14 × 10^2^	3.77 × 10^2^	4.67 × 10^2^	5.05 × 10^2^	2.05 × 10^2^	2.21 × 10^1^	1.93 × 10^2^	1.94 × 10^3^
	Best	3.72 × 10^3^	4.61 × 10^3^	3.90 × 10^3^	5.14 × 10^3^	4.23 × 10^3^	4.90 × 10^3^	5.60 × 10^3^	3.90 × 10^3^	4.48 × 10^3^	3.70 × 10^3^	4.91 × 10^3^	6.05 × 10^3^
F25	Mean	3.26 × 10^3^	3.29 × 10^3^	6.28 × 10^3^	3.65 × 10^3^	3.79 × 10^3^	3.29 × 10^3^	3.56 × 10^3^	3.29 × 10^3^	3.55 × 10^3^	3.47 × 10^3^	1.10 × 10^4^	3.40 × 10^3^
	Std	5.89 × 10^1^	7.59 × 10^1^	7.28 × 10^2^	7.50 × 10^1^	1.80 × 10^3^	7.29 × 10^1^	6.56 × 10^1^	6.62 × 10^1^	4.77 × 10^1^	2.22 × 10^1^	1.30 × 10^3^	4.33 × 10^1^
	Best	3.16 × 10^3^	3.12 × 10^3^	5.17 × 10^3^	3.48 × 10^3^	3.34 × 10^3^	3.14 × 10^3^	3.39 × 10^3^	3.14 × 10^3^	3.46 × 10^3^	3.41 × 10^3^	7.93 × 10^3^	3.31 × 10^3^
F26	Mean	1.16 × 10^4^	1.40 × 10^4^	1.47 × 10^4^	3.10 × 10^4^	1.87 × 10^4^	2.67 × 10^4^	3.03 × 10^4^	2.26 × 10^4^	8.96 × 10^3^	1.10 × 10^4^	2.54 × 10^4^	2.28 × 10^4^
	Std	6.75 × 10^2^	7.39 × 10^3^	1.34 × 10^3^	2.51 × 10^3^	3.00 × 10^3^	3.49 × 10^3^	5.18 × 10^3^	8.26 × 10^3^	6.04 × 10^3^	2.40 × 10^2^	1.98 × 10^3^	6.91 × 10^3^
	Best	1.01 × 10^4^	3.19 × 10^3^	1.27 × 10^4^	2.68 × 10^4^	1.29 × 10^4^	1.90 × 10^4^	1.08 × 10^4^	2.90 × 10^3^	5.17 × 10^3^	1.04 × 10^4^	2.29 × 10^4^	4.63 × 10^3^
F27	Mean	3.40 × 10^3^	3.81 × 10^3^	4.00 × 10^3^	4.81 × 10^3^	4.05 × 10^3^	4.06 × 10^3^	4.81 × 10^3^	4.30 × 10^3^	5.67 × 10^3^	3.41 × 10^3^	4.99 × 10^3^	4.10 × 10^3^
	Std	4.75 × 10^1^	2.18 × 10^2^	1.54 × 10^2^	5.72 × 10^2^	3.15 × 10^2^	2.48 × 10^2^	4.92 × 10^2^	3.81 × 10^2^	4.39 × 10^2^	1.98 × 10^1^	3.26 × 10^2^	5.43 × 10^2^
	Best	3.36 × 10^3^	3.50 × 10^3^	3.60 × 10^3^	3.79 × 10^3^	3.54 × 10^3^	3.68 × 10^3^	3.95 × 10^3^	3.76 × 10^3^	5.02 × 10^3^	3.37 × 10^3^	4.48 × 10^3^	3.63 × 10^3^
F28	Mean	3.38 × 10^3^	3.66 × 10^3^	8.68 × 10^3^	3.74 × 10^3^	1.40 × 10^4^	3.39 × 10^3^	3.70 × 10^3^	4.29 × 10^4^	4.14 × 10^3^	3.49 × 10^3^	1.41 × 10^4^	3.44 × 10^3^
	Std	1.97 × 10^1^	4.10 × 10^2^	1.42 × 10^3^	7.33 × 10^1^	8.37 × 10^3^	3.30 × 10^1^	7.17 × 10^1^	7.33 × 10^2^	1.13 × 10^2^	2.23 × 10^1^	1.80 × 10^3^	3.65 × 10^1^
	Best	3.35 × 10^3^	3.39 × 10^3^	6.80 × 10^3^	3.61 × 10^3^	3.48 × 10^3^	3.33 × 10^3^	3.51 × 10^3^	4.21 × 10^4^	3.83 × 10^3^	3.45 × 10^3^	1.05 × 10^4^	3.37 × 10^3^
F29	Mean	6.46 × 10^3^	7.20 × 10^3^	7.96 × 10^3^	1.39 × 10^4^	8.82 × 10^3^	8.07 × 10^3^	1.26 × 10^4^	7.97 × 10^3^	7.81 × 10^3^	5.70 × 10^3^	1.39 × 10^4^	7.93 × 10^3^
	Std	3.31 × 10^2^	5.04 × 10^2^	7.98 × 10^2^	1.50 × 10^3^	1.09 × 10^3^	6.89 × 10^2^	1.29 × 10^3^	6.30 × 10^2^	5.95 × 10^2^	3.12 × 10^2^	1.23 × 10^3^	6.43 × 10^2^
	Best	6.11 × 10^3^	6.24 × 10^3^	6.81 × 10^3^	1.13 × 10^4^	6.46 × 10^3^	6.88 × 10^3^	1.06 × 10^4^	6.64 × 10^3^	6.63 × 10^3^	5.23 × 10^3^	1.20 × 10^4^	7.14 × 10^3^
F30	Mean	1.48 × 10^4^	9.74 × 10^6^	5.59 × 10^8^	2.19 × 10^8^	2.58 × 10^7^	1.41 × 10^4^	8.53 × 10^7^	1.88 × 10^4^	1.51 × 10^7^	3.42 × 10^5^	1.58 × 10^9^	4.32 × 10^4^
	Std	5.85 × 10^3^	5.06 × 10^7^	6.81 × 10^8^	1.29 × 10^8^	3.52 × 10^7^	6.21 × 10^3^	5.02 × 10^7^	6.26 × 10^3^	4.74 × 10^6^	8.50 × 10^4^	5.80 × 10^8^	3.47 × 10^4^
	Best	8.98 × 10^3^	6.30 × 10^3^	8.18 × 10^7^	6.83 × 10^7^	1.82 × 10^6^	6.84 × 10^3^	2.74 × 10^7^	8.04 × 10^3^	6.34 × 10^6^	1.89 × 10^5^	7.21 × 10^8^	9.86 × 10^3^

**Table A4:** Experimental results of 12 algorithms in CEC 2022 (Dim = 10).

Algorithm	Metric	SSMO	PSO	GWO	WOA	DBO	GWCA	SGA	IVY	FATA	PLO	SFOA	EMWPA
F1	Mean	3.00 × 10^2^	3.00 × 10^2^	1.72 × 10^3^	4.69 × 10^3^	3.00 × 10^2^	3.00 × 10^2^	3.00 × 10^2^	3.00 × 10^2^	6.91 × 10^2^	3.00 × 10^2^	6.50 × 10^3^	3.52 × 10^2^
	Std	1.17 × 10^−4^	1.83 × 10^−14^	1.64 × 10^3^	1.94 × 10^3^	1.28 × 10^−13^	7.54 × 10^−14^	4.76 × 10^−1^	1.02 × 10^−13^	9.77 × 10^2^	1.25 × 10^−4^	4.68 × 10^3^	2.86 × 10^2^
	Best	3.00 × 10^2^	3.00 × 10^2^	3.27 × 10^2^	1.09 × 10^3^	3.00 × 10^2^	3.00 × 10^2^	3.00 × 10^2^	3.00 × 10^2^	3.01 × 10^2^	3.00 × 10^2^	1.64 × 10^3^	3.00 × 10^2^
F2	Mean	4.03 × 10^2^	4.08 × 10^2^	4.19 × 10^2^	4.24 × 10^2^	4.24 × 10^2^	4.16 × 10^2^	4.12 × 10^2^	4.17 × 10^2^	4.15 × 10^2^	4.00 × 10^2^	4.52 × 10^2^	4.11 × 10^2^
	Std	2.30 × 10^0^	1.34 × 10^1^	1.72 × 10^1^	3.03 × 10^1^	3.02 × 10^1^	2.53 × 10^1^	2.04 × 10^1^	2.72 × 10^1^	2.27 × 10^1^	9.29 × 10^−2^	3.40 × 10^1^	2.06 × 10^1^
	Best	4.00 × 10^2^	4.00 × 10^2^	4.01 × 10^2^	4.00 × 10^2^	4.00 × 10^2^	4.00 × 10^2^	4.00 × 10^2^	4.00 × 10^2^	4.00 × 10^2^	4.00 × 10^2^	4.10 × 10^2^	4.00 × 10^2^
F3	Mean	6.00 × 10^2^	6.07 × 10^2^	6.01 × 10^2^	6.26 × 10^2^	6.05 × 10^2^	6.11 × 10^2^	6.17 × 10^2^	6.01 × 10^2^	6.05 × 10^2^	6.00 × 10^2^	6.23 × 10^2^	6.25 × 10^2^
	Std	2.66 × 10^−3^	7.31 × 10^0^	1.46 × 10^0^	1.20 × 10^1^	5.03 × 10^0^	6.19 × 10^0^	1.05 × 10^1^	3.23 × 10^0^	4.07 × 10^0^	8.71 × 10^−3^	1.15 × 10^1^	1.42 × 10^1^
	Best	6.00 × 10^2^	6.00 × 10^2^	6.00 × 10^2^	6.07 × 10^2^	6.00 × 10^2^	6.01 × 10^2^	6.03 × 10^2^	6.00 × 10^2^	6.00 × 10^2^	6.00 × 10^2^	6.10 × 10^2^	6.00 × 10^2^
F4	Mean	8.06 × 10^2^	8.22 × 10^2^	8.13 × 10^2^	8.43 × 10^2^	8.21 × 10^2^	8.21 × 10^2^	8.26 × 10^2^	8.20 × 10^2^	8.25 × 10^2^	8.07 × 10^2^	8.52 × 10^2^	8.25 × 10^2^
	Std	2.73 × 10^0^	1.03 × 10^1^	4.32 × 10^0^	1.47 × 10^1^	8.74 × 10^0^	8.22 × 10^0^	8.57 × 10^0^	6.78 × 10^0^	6.78 × 10^0^	2.03 × 10^0^	1.33 × 10^1^	1.03 × 10^1^
	Best	8.01 × 10^2^	8.06 × 10^2^	8.03 × 10^2^	8.18 × 10^2^	8.04 × 10^2^	8.09 × 10^2^	8.10 × 10^2^	8.05 × 10^2^	8.09 × 10^2^	8.03 × 10^2^	8.29 × 10^2^	8.07 × 10^2^
F5	Mean	9.00 × 10^2^	9.16 × 10^2^	9.11 × 10^2^	1.27 × 10^3^	9.73 × 10^2^	1.00 × 10^3^	9.84 × 10^2^	1.39 × 10^3^	1.04 × 10^3^	9.00 × 10^2^	1.21 × 10^3^	1.28 × 10^3^
	Std	1.16 × 10^−5^	8.53 × 10^1^	1.52 × 10^1^	2.56 × 10^2^	1.18 × 10^2^	7.49 × 10^1^	8.89 × 10^1^	1.19 × 10^2^	2.22 × 10^2^	1.63 × 10^−2^	2.98 × 10^2^	2.42 × 10^2^
	Best	9.00 × 10^2^	9.00 × 10^2^	9.00 × 10^2^	9.23 × 10^2^	9.02 × 10^2^	9.02 × 10^2^	9.00 × 10^2^	1.11 × 10^3^	9.00 × 10^2^	9.00 × 10^2^	9.25 × 10^2^	9.90 × 10^2^
F6	Mean	1.80 × 10^3^	3.48 × 10^3^	6.96 × 10^3^	3.92 × 10^3^	5.54 × 10^3^	3.21 × 10^3^	4.39 × 10^3^	3.42 × 10^3^	4.44 × 10^3^	1.93 × 10^3^	1.13 × 10^6^	2.51 × 10^3^
	Std	2.31 × 10^−1^	2.02 × 10^3^	1.72 × 10^3^	2.07 × 10^3^	2.02 × 10^3^	1.35 × 10^3^	2.24 × 10^3^	1.71 × 10^3^	2.13 × 10^3^	7.22 × 10^1^	9.35 × 10^5^	7.08 × 10^2^
	Best	1.80 × 10^3^	1.81 × 10^3^	2.59 × 10^3^	1.86 × 10^3^	1.84 × 10^3^	1.85 × 10^3^	1.88 × 10^3^	1.87 × 10^3^	1.88 × 10^3^	1.83 × 10^3^	4.52 × 10^4^	1.81 × 10^3^
F7	Mean	2.01 × 10^3^	2.03 × 10^3^	2.03 × 10^3^	2.05 × 10^3^	2.03 × 10^3^	2.04 × 10^3^	2.04 × 10^3^	2.04 × 10^3^	2.02 × 10^3^	2.00 × 10^3^	2.07 × 10^3^	2.07 × 10^3^
	Std	9.40 × 10^0^	1.59 × 10^1^	7.71 × 10^0^	2.81 × 10^1^	1.23 × 10^1^	1.07 × 10^1^	1.43 × 10^1^	2.00 × 10^1^	5.76 × 10^0^	5.77 × 10^0^	1.72 × 10^1^	4.36 × 10^1^
	Best	2.00 × 10^3^	2.00 × 10^3^	2.01 × 10^3^	2.01 × 10^3^	2.02 × 10^3^	2.02 × 10^3^	2.02 × 10^3^	2.00 × 10^3^	2.01 × 10^3^	2.00 × 10^3^	2.04 × 10^3^	2.02 × 10^3^
F8	Mean	2.20 × 10^3^	2.22 × 10^3^	2.22 × 10^3^	2.23 × 10^3^	2.22 × 10^3^	2.22 × 10^3^	2.23 × 10^3^	2.26 × 10^3^	2.22 × 10^3^	2.21 × 10^3^	2.24 × 10^3^	2.27 × 10^3^
	Std	3.69 × 10^0^	2.24 × 10^1^	6.23 × 10^0^	7.05 × 10^0^	5.25 × 10^0^	2.29 × 10^0^	8.89 × 10^0^	6.75 × 10^1^	5.26 × 10^0^	6.85 × 10^0^	5.75 × 10^0^	5.64 × 10^1^
	Best	2.20 × 10^3^	2.20 × 10^3^	2.20 × 10^3^	2.21 × 10^3^	2.21 × 10^3^	2.22 × 10^3^	2.21 × 10^3^	2.22 × 10^3^	2.20 × 10^3^	2.20 × 10^3^	2.23 × 10^3^	2.22 × 10^3^
F9	Mean	2.52 × 10^3^	2.53 × 10^3^	2.57 × 10^3^	2.54 × 10^3^	2.53 × 10^3^	2.53 × 10^3^	2.53 × 10^3^	2.53 × 10^3^	2.53 × 10^3^	2.53 × 10^3^	2.60 × 10^3^	2.53 × 10^3^
	Std	2.36 × 10^1^	5.10 × 10^0^	2.59 × 10^1^	3.05 × 10^1^	2.68 × 10^1^	2.68 × 10^1^	2.46 × 10^−4^	1.80 × 10^−11^	7.29 × 10^−3^	1.00 × 10^−5^	4.44 × 10^1^	2.84 × 10^−9^
	Best	2.40 × 10^3^	2.53 × 10^3^	2.53 × 10^3^	2.53 × 10^3^	2.53 × 10^3^	2.53 × 10^3^	2.53 × 10^3^	2.53 × 10^3^	2.53 × 10^3^	2.53 × 10^3^	2.53 × 10^3^	2.53 × 10^3^
F10	Mean	2.51 × 10^3^	2.61 × 10^3^	2.57 × 10^3^	2.57 × 10^3^	2.51 × 10^3^	2.53 × 10^3^	2.52 × 10^3^	2.56 × 10^3^	2.51 × 10^3^	2.48 × 10^3^	2.52 × 10^3^	2.62 × 10^3^
	Std	2.84 × 10^1^	1.31 × 10^2^	6.26 × 10^1^	1.63 × 10^2^	3.19 × 10^1^	5.63 × 10^1^	4.80 × 10^1^	6.62 × 10^1^	3.06 × 10^1^	3.29 × 10^1^	5.41 × 10^1^	2.06 × 10^2^
	Best	2.50 × 10^3^	2.50 × 10^3^	2.50 × 10^3^	2.50 × 10^3^	2.50 × 10^3^	2.50 × 10^3^	2.50 × 10^3^	2.50 × 10^3^	2.50 × 10^3^	2.41 × 10^3^	2.50 × 10^3^	2.50 × 10^3^
F11	Mean	2.60 × 10^3^	2.69 × 10^3^	2.80 × 10^3^	2.76 × 10^3^	2.71 × 10^3^	2.78 × 10^3^	2.77 × 10^3^	2.76 × 10^3^	2.74 × 10^3^	2.60 × 10^3^	2.86 × 10^3^	2.79 × 10^3^
	Std	1.30 × 10^−2^	1.48 × 10^2^	1.71 × 10^2^	1.70 × 10^2^	1.42 × 10^2^	1.48 × 10^2^	2.13 × 10^2^	1.77 × 10^2^	1.51 × 10^2^	2.57 × 10^−2^	1.94 × 10^2^	1.75 × 10^2^
	Best	2.60 × 10^3^	2.60 × 10^3^	2.60 × 10^3^	2.60 × 10^3^	2.60 × 10^3^	2.60 × 10^3^	2.60 × 10^3^	2.60 × 10^3^	2.60 × 10^3^	2.60 × 10^3^	2.67 × 10^3^	2.60 × 10^3^
F12	Mean	2.86 × 10^3^	2.92 × 10^3^	2.87 × 10^3^	2.88 × 10^3^	2.87 × 10^3^	2.87 × 10^3^	2.87 × 10^3^	2.89 × 10^3^	2.87 × 10^3^	2.86 × 10^3^	2.87 × 10^3^	2.88 × 10^3^
	Std	1.71 × 10^0^	4.83 × 10^1^	7.61 × 10^0^	3.08 × 10^1^	3.64 × 10^0^	2.22 × 10^0^	1.53 × 10^1^	3.86 × 10^1^	5.98 × 10^0^	1.68 × 10^0^	4.73 × 10^0^	4.36 × 10^1^
	Best	2.86 × 10^3^	2.87 × 10^3^	2.86 × 10^3^	2.86 × 10^3^	2.86 × 10^3^	2.86 × 10^3^	2.86 × 10^3^	2.86 × 10^3^	2.86 × 10^3^	2.86 × 10^3^	2.87 × 10^3^	2.86 × 10^3^

**Table A5:** Experimental results of 12 algorithms in CEC 2022 (Dim = 20).

Algorithm	Metric	SSMO	PSO	GWO	WOA	DBO	GWCA	SGA	IVY	FATA	PLO	SFOA	EMWPA
F1	Mean	3.00 × 10^2^	3.00 × 10^2^	6.58 × 10^3^	3.28 × 10^2^	3.01 × 10^2^	3.00 × 10^2^	3.07 × 10^2^	3.00 × 10^2^	3.01 × 10^2^	7.08 × 10^2^	1.49 × 10^4^	1.08 × 10^3^
	Std	1.87 × 10^−1^	3.50 × 10^−14^	3.27 × 10^3^	3.13 × 10^1^	4.41 × 10^0^	1.04 × 10^−13^	7.86 × 10^0^	2.95 × 10^−13^	6.12 × 10^−1^	6.30 × 10^1^	5.55 × 10^3^	3.64 × 10^3^
	Best	3.00 × 10^2^	3.00 × 10^2^	7.14 × 10^2^	3.01 × 10^2^	3.00 × 10^2^	3.00 × 10^2^	3.00 × 10^2^	3.00 × 10^2^	3.00 × 10^2^	5.49 × 10^2^	5.80 × 10^3^	3.00 × 10^2^
F2	Mean	4.37 × 10^2^	4.54 × 10^2^	4.92 × 10^2^	4.57 × 10^2^	4.52 × 10^2^	4.29 × 10^2^	4.55 × 10^2^	4.42 × 10^2^	4.51 × 10^2^	4.14 × 10^2^	5.31 × 10^2^	4.30 × 10^2^
	Std	1.93 × 10^1^	1.68 × 10^1^	3.42 × 10^1^	1.82 × 10^1^	3.16 × 10^1^	2.38 × 10^1^	2.14 × 10^1^	1.61 × 10^1^	1.65 × 10^1^	1.64 × 10^1^	3.66 × 10^1^	2.36 × 10^1^
	Best	4.00 × 10^2^	4.07 × 10^2^	4.49 × 10^2^	4.11 × 10^2^	4.00 × 10^2^	4.00 × 10^2^	4.01 × 10^2^	4.00 × 10^2^	4.06 × 10^2^	4.00 × 10^2^	4.66 × 10^2^	4.00 × 10^2^
F3	Mean	6.00 × 10^2^	6.26 × 10^2^	6.04 × 10^2^	6.56 × 10^2^	6.20 × 10^2^	6.30 × 10^2^	6.45 × 10^2^	6.07 × 10^2^	6.21 × 10^2^	6.00 × 10^2^	6.41 × 10^2^	6.47 × 10^2^
	Std	1.48 × 10^−2^	1.44 × 10^1^	3.13 × 10^0^	1.09 × 10^1^	1.25 × 10^1^	8.67 × 10^0^	8.75 × 10^0^	8.40 × 10^0^	7.23 × 10^0^	3.10 × 10^−2^	1.87 × 10^1^	1.01 × 10^1^
	Best	6.00 × 10^2^	6.02 × 10^2^	6.00 × 10^2^	6.28 × 10^2^	6.02 × 10^2^	6.11 × 10^2^	6.26 × 10^2^	6.00 × 10^2^	6.08 × 10^2^	6.00 × 10^2^	6.17 × 10^2^	6.24 × 10^2^
F4	Mean	8.31 × 10^2^	8.73 × 10^2^	8.44 × 10^2^	9.15 × 10^2^	8.95 × 10^2^	8.66 × 10^2^	8.89 × 10^2^	8.64 × 10^2^	8.82 × 10^2^	8.22 × 10^2^	9.46 × 10^2^	8.90 × 10^2^
	Std	9.55 × 10^0^	1.79 × 10^1^	1.79 × 10^1^	3.09 × 10^1^	1.87 × 10^1^	1.72 × 10^1^	2.28 × 10^1^	1.79 × 10^1^	9.87 × 10^0^	5.45 × 10^0^	2.15 × 10^1^	2.11 × 10^1^
	Best	8.19 × 10^2^	8.47 × 10^2^	8.21 × 10^2^	8.67 × 10^2^	8.51 × 10^2^	8.29 × 10^2^	8.47 × 10^2^	8.30 × 10^2^	8.53 × 10^2^	8.09 × 10^2^	8.95 × 10^2^	8.37 × 10^2^
F5	Mean	9.00 × 10^2^	1.51 × 10^3^	1.13 × 10^3^	3.10 × 10^3^	1.64 × 10^3^	1.93 × 10^3^	2.14 × 10^3^	2.39 × 10^3^	2.15 × 10^3^	9.06 × 10^2^	2.94 × 10^3^	2.28 × 10^3^
	Std	7.44 × 10^−2^	4.65 × 10^2^	2.52 × 10^2^	1.10 × 10^3^	4.54 × 10^2^	4.16 × 10^2^	4.66 × 10^2^	1.48 × 10^2^	3.44 × 10^2^	4.37 × 10^0^	1.05 × 10^3^	5.46 × 10^2^
	Best	9.00 × 10^2^	9.00 × 10^2^	9.02 × 10^2^	1.29 × 10^3^	1.01 × 10^3^	1.19 × 10^3^	1.27 × 10^3^	2.04 × 10^3^	1.03 × 10^3^	9.00 × 10^2^	1.10 × 10^3^	1.32 × 10^3^
F6	Mean	1.85 × 10^3^	3.63 × 10^3^	4.55 × 10^5^	7.99 × 10^3^	1.88 × 10^5^	5.74 × 10^3^	7.61 × 10^3^	5.47 × 10^3^	4.11 × 10^3^	2.06 × 10^3^	1.11 × 10^7^	4.82 × 10^3^
	Std	3.95 × 10^1^	2.22 × 10^3^	1.30 × 10^6^	5.39 × 10^3^	7.17 × 10^5^	4.60 × 10^3^	7.03 × 10^3^	5.43 × 10^3^	2.91 × 10^3^	5.44 × 10^1^	5.06 × 10^6^	3.76 × 10^3^
	Best	1.81 × 10^3^	1.85 × 10^3^	1.91 × 10^3^	1.95 × 10^3^	2.00 × 10^3^	1.93 × 10^3^	1.97 × 10^3^	1.89 × 10^3^	1.99 × 10^3^	1.96 × 10^3^	3.41 × 10^6^	1.89 × 10^3^
F7	Mean	2.02 × 10^3^	2.11 × 10^3^	2.05 × 10^3^	2.14 × 10^3^	2.09 × 10^3^	2.10 × 10^3^	2.13 × 10^3^	2.14 × 10^3^	2.09 × 10^3^	2.02 × 10^3^	2.15 × 10^3^	2.17 × 10^3^
	Std	7.59 × 10^0^	6.25 × 10^1^	1.98 × 10^1^	3.93 × 10^1^	3.28 × 10^1^	2.74 × 10^1^	4.98 × 10^1^	4.97 × 10^1^	1.97 × 10^1^	3.34 × 10^0^	4.29 × 10^1^	7.45 × 10^1^
	Best	2.00 × 10^3^	2.04 × 10^3^	2.03 × 10^3^	2.08 × 10^3^	2.04 × 10^3^	2.06 × 10^3^	2.06 × 10^3^	2.06 × 10^3^	2.05 × 10^3^	2.01 × 10^3^	2.09 × 10^3^	2.08 × 10^3^
F8	Mean	2.22 × 10^3^	2.31 × 10^3^	2.23 × 10^3^	2.24 × 10^3^	2.26 × 10^3^	2.23 × 10^3^	2.30 × 10^3^	2.41 × 10^3^	2.23 × 10^3^	2.22 × 10^3^	2.28 × 10^3^	2.30 × 10^3^
	Std	5.21 × 10^0^	1.04 × 10^2^	2.19 × 10^1^	9.50 × 10^0^	5.02 × 10^1^	2.16 × 10^1^	7.97 × 10^1^	1.31 × 10^2^	2.29 × 10^1^	2.96 × 10^−1^	5.69 × 10^1^	8.78 × 10^1^
	Best	2.20 × 10^3^	2.22 × 10^3^	2.22 × 10^3^	2.23 × 10^3^	2.22 × 10^3^	2.22 × 10^3^	2.23 × 10^3^	2.22 × 10^3^	2.22 × 10^3^	2.22 × 10^3^	2.24 × 10^3^	2.22 × 10^3^
F9	Mean	2.48 × 10^3^	2.48 × 10^3^	2.49 × 10^3^	2.48 × 10^3^	2.48 × 10^3^	2.48 × 10^3^	2.48 × 10^3^	2.48 × 10^3^	2.48 × 10^3^	2.48 × 10^3^	2.51 × 10^3^	2.48 × 10^3^
	Std	3.87 × 10^−6^	1.21 × 10^1^	1.16 × 10^1^	3.92 × 10^−1^	5.75 × 10^−1^	2.66 × 10^−12^	5.55 × 10^−2^	6.04 × 10^−7^	1.01 × 10^−1^	1.93 × 10^−4^	1.99 × 10^1^	2.08 × 10^−2^
	Best	2.48 × 10^3^	2.48 × 10^3^	2.48 × 10^3^	2.48 × 10^3^	2.48 × 10^3^	2.48 × 10^3^	2.48 × 10^3^	2.48 × 10^3^	2.48 × 10^3^	2.48 × 10^3^	2.49 × 10^3^	2.48 × 10^3^
F10	Mean	2.55 × 10^3^	4.12 × 10^3^	2.93 × 10^3^	3.86 × 10^3^	2.50 × 10^3^	3.21 × 10^3^	3.06 × 10^3^	3.98 × 10^3^	2.57 × 10^3^	2.50 × 10^3^	4.03 × 10^3^	4.58 × 10^3^
	Std	1.35 × 10^2^	7.34 × 10^2^	4.07 × 10^2^	1.09 × 10^3^	2.20 × 10^−1^	1.03 × 10^3^	9.10 × 10^2^	9.16 × 10^2^	1.04 × 10^2^	2.47 × 10^−1^	1.51 × 10^3^	1.13 × 10^3^
	Best	2.50 × 10^3^	2.50 × 10^3^	2.50 × 10^3^	2.50 × 10^3^	2.50 × 10^3^	2.50 × 10^3^	2.50 × 10^3^	2.50 × 10^3^	2.50 × 10^3^	2.50 × 10^3^	2.50 × 10^3^	2.50 × 10^3^
F11	Mean	2.75 × 10^3^	2.95 × 10^3^	3.39 × 10^3^	2.91 × 10^3^	2.92 × 10^3^	2.91 × 10^3^	2.92 × 10^3^	3.03 × 10^3^	2.95 × 10^3^	3.08 × 10^3^	3.51 × 10^3^	2.92 × 10^3^
	Std	1.96 × 10^2^	1.47 × 10^2^	2.79 × 10^2^	6.89 × 10^1^	1.50 × 10^2^	9.44 × 10^1^	7.27 × 10^1^	5.21 × 10^2^	9.08 × 10^1^	1.76 × 10^1^	1.15 × 10^2^	9.85 × 10^1^
	Best	2.60 × 10^3^	2.60 × 10^3^	2.92 × 10^3^	2.60 × 10^3^	2.60 × 10^3^	2.60 × 10^3^	2.60 × 10^3^	2.90 × 10^3^	2.90 × 10^3^	3.05 × 10^3^	3.29 × 10^3^	2.60 × 10^3^
F12	Mean	2.94 × 10^3^	3.27 × 10^3^	2.96 × 10^3^	3.00 × 10^3^	3.02 × 10^3^	2.99 × 10^3^	2.98 × 10^3^	3.04 × 10^3^	2.97 × 10^3^	2.94 × 10^3^	2.98 × 10^3^	3.08 × 10^3^
	Std	2.67 × 10^0^	1.83 × 10^2^	1.11 × 10^1^	4.60 × 10^1^	3.75 × 10^1^	5.59 × 10^1^	3.93 × 10^1^	9.58 × 10^1^	1.66 × 10^1^	2.08 × 10^0^	1.56 × 10^1^	1.19 × 10^2^
	Best	2.93 × 10^3^	3.02 × 10^3^	2.94 × 10^3^	2.94 × 10^3^	2.96 × 10^3^	2.94 × 10^3^	2.94 × 10^3^	2.95 × 10^3^	2.95 × 10^3^	2.94 × 10^3^	2.96 × 10^3^	2.96 × 10^3^

**Table A6:** Results of parameter extraction for each algorithm in SDM.

Algorithm	*I_ph_* (A)	*I_sd_* (A)	*R_s_* (Ω)	*R_sh_* (Ω)	*n*
SSMO	7.6077 × 10^−1^	3.2307 × 10^−7^	3.6376 × 10^−2^	5.3732 × 10^1^	1.4812 × 10^0^
PSO	7.6078 × 10^−1^	3.2006 × 10^−7^	3.6414 × 10^−2^	5.3472 × 10^1^	1.4803 × 10^0^
GWO	7.5918 × 10^−1^	3.0878 × 10^−7^	3.7156 × 10^−2^	9.1414 × 10^1^	1.4762 × 10^0^
WOA	7.6112 × 10^−1^	4.8230 × 10^−7^	3.4640 × 10^−2^	6.0117 × 10^1^	1.5227 × 10^0^
DBO	7.6076 × 10^−1^	3.3810 × 10^−7^	3.6182 × 10^−2^	5.4822 × 10^1^	1.4858 × 10^0^
GWCA	7.6077 × 10^−1^	3.2875 × 10^−7^	3.6307 × 10^−2^	5.4236 × 10^1^	1.4830 × 10^0^
SGA	7.5807 × 10^−1^	7.6876 × 10^−7^	3.6255 × 10^−2^	6.9424 × 10^1^	1.5747 × 10^0^
IVY	7.6110 × 10^−1^	9.9928 × 10^−7^	3.1336 × 10^−2^	8.9873 × 10^1^	1.6045 × 10^0^
FATA	7.5752 × 10^−1^	1.0000 × 10^−6^	3.0035 × 10^−2^	7.1411 × 10^1^	1.6061 × 10^0^
PLO	7.5921 × 10^−1^	5.5867 × 10^−7^	3.4075 × 10^−2^	9.8792 × 10^1^	1.5384 × 10^0^
SFOA	7.5910 × 10^−1^	7.9209 × 10^−8^	4.1416 × 10^−2^	7.3201 × 10^1^	1.3503 × 10^0^
EMWPA	7.6086 × 10^−1^	3.0661 × 10^−7^	3.6584 × 10^−2^	5.1860 × 10^1^	1.4760 × 10^0^

**Table A7:** Results of parameter extraction for each algorithm in DDM.

Algorithm	*I_ph_* (A)	*R_s_* (Ω)	*R_sh_* (Ω)	*I_sd_*_1_(A)	*n*_1_	*I_sd_*_2_ (A)	*n*_2_
SSMO	7.6078 × 10^−1^	3.6695 × 10^−2^	5.5307 × 10^1^	2.3465 × 10^−7^	1.4542 × 10^0^	6.7977 × 10^−7^	2.0000 × 10^0^
PSO	7.6072 × 10^−1^	3.6500 × 10^−2^	5.6963 × 10^1^	2.4721 × 10^−7^	1.4593 × 10^0^	6.7717 × 10^−7^	2.0000 × 10^0^
GWO	7.6093 × 10^−1^	3.6030 × 10^−2^	5.3169 × 10^1^	5.2009 × 10^−7^	1.5869 × 10^0^	3.3002 × 10^−8^	1.3565 × 10^0^
WOA	7.6000 × 10^−1^	3.6381 × 10^−2^	8.4649 × 10^1^	5.6325 × 10^−7^	1.8653 × 10^0^	2.3541 × 10^−7^	1.4581 × 10^0^
DBO	7.6074 × 10^−1^	3.6088 × 10^−2^	5.5582 × 10^1^	0.0000 × 10^0^	2.0000 × 10^0^	3.4574 × 10^−7^	1.4881 × 10^0^
GWCA	7.6076 × 10^−1^	3.6414 × 10^−2^	5.5383 × 10^1^	2.8439 × 10^−7^	1.4708 × 10^0^	3.4901 × 10^−7^	2.0000 × 10^0^
SGA	7.5694 × 10^−1^	3.4473 × 10^−2^	7.7262 × 10^1^	2.7205 × 10^−7^	1.4781 × 10^0^	7.7000 × 10^−7^	1.8489 × 10^0^
IVY	7.6139 × 10^−1^	3.4630 × 10^−2^	9.8899 × 10^1^	2.3902 × 10^−10^	1.9649 × 10^0^	5.8676 × 10^−7^	1.5434 × 10^0^
FATA	7.6410 × 10^−1^	3.3916 × 10^−2^	6.1456 × 10^1^	4.4799 × 10^−7^	1.5213 × 10^0^	4.8598 × 10^−7^	1.9064 × 10^0^
PLO	7.6134 × 10^−1^	3.2130 × 10^−2^	9.9974 × 10^1^	9.9984 × 10^−7^	1.9857 × 10^0^	6.0390 × 10^−7^	1.5553 × 10^0^
SFOA	7.6140 × 10^−1^	3.5337 × 10^−2^	8.7267 × 10^1^	7.8860 × 10^−7^	1.7680 × 10^0^	3.2418 × 10^−7^	1.5073 × 10^0^
EMWPA	7.6078 × 10^−1^	3.6650 × 10^−2^	5.4495 × 10^1^	2.3731 × 10^−7^	1.4562 × 10^0^	3.7873 × 10^−7^	1.8713 × 10^0^

**Table A8:** Results of parameter extraction for each algorithm in TDM.

Algorithm	*I_ph_* (A)	*R_s_* (Ω)	*R_sh_* (Ω)	*I_sd_*_1_ (A)	*n*_1_	*I_sd_*_2_ (A)	*n*_2_	*I_sd_*_3_ (A)	*n*_3_
SSMO	7.6077 × 10^−1^	3.6835 × 10^−2^	5.5828 × 10^1^	4.8862 × 10^−7^	1.9675 × 10^0^	2.0691 × 10^−7^	1.4438 × 10^0^	3.4552 × 10^−7^	1.9960 × 10^0^
PSO	7.6083 × 10^−1^	3.6991 × 10^−2^	5.3723 × 10^1^	7.0296 × 10^−7^	2.0000 × 10^0^	0.0000 × 10^0^	1.8885 × 10^0^	2.1585 × 10^−7^	1.4463 × 10^0^
GWO	7.6020 × 10^−1^	3.6061 × 10^−2^	5.8787 × 10^1^	4.2489 × 10^−7^	1.6016 × 10^0^	8.4193 × 10^−8^	1.4071 × 10^0^	0.0000 × 10^0^	1.0474 × 10^0^
WOA	7.6241 × 10^−1^	3.4585 × 10^−2^	4.7926 × 10^1^	0.0000 × 10^0^	1.3497 × 10^0^	1.0140 × 10^−7^	1.7717 × 10^0^	4.4125 × 10^−7^	1.5165 × 10^0^
DBO	7.6049 × 10^−1^	3.4697 × 10^−2^	7.1423 × 10^1^	0.0000 × 10^0^	2.0000 × 10^0^	4.8455 × 10^−7^	1.5232 × 10^0^	0.0000 × 10^0^	2.0000 × 10^0^
GWCA	7.6089 × 10^−1^	3.7062 × 10^−2^	5.3282 × 10^1^	6.2516 × 10^−7^	1.9528 × 10^0^	1.8072 × 10^−7^	1.4337 × 10^0^	8.0019 × 10^−8^	1.7104 × 10^0^
SGA	7.7258 × 10^−1^	3.7554 × 10^−2^	9.9992 × 10^1^	1.0000 × 10^−6^	2.0000 × 10^0^	9.9982 × 10^−7^	1.9820 × 10^0^	1.0000 × 10^−6^	1.6212 × 10^0^
IVY	7.6107 × 10^−1^	3.1386 × 10^−2^	9.1550 × 10^1^	5.3040 × 10^−13^	1.6161 × 10^0^	9.9566 × 10^−7^	1.6043 × 10^0^	1.0012 × 10^−10^	1.3106 × 10^0^
FATA	7.6533 × 10^−1^	3.6377 × 10^−2^	3.6775 × 10^1^	3.6150 × 10^−7^	1.6865 × 10^0^	7.5999 × 10^−8^	1.3866 × 10^0^	1.4511 × 10^−7^	1.5834 × 10^0^
PLO	7.6049 × 10^−1^	3.5789 × 10^−2^	9.5225 × 10^1^	2.6274 × 10^−7^	1.6370 × 10^0^	9.8731 × 10^−7^	1.8995 × 10^0^	1.1334 × 10^−7^	1.4171 × 10^0^
SFOA	7.5998 × 10^−1^	3.7287 × 10^−2^	9.7138 × 10^1^	2.4847 × 10^−7^	1.4570 × 10^0^	0.0000 × 10^0^	1.0057 × 10^0^	2.8968 × 10^−7^	1.9850 × 10^0^
EMWPA	7.6082 × 10^−1^	3.6869 × 10^−2^	5.4262 × 10^1^	6.0872 × 10^−7^	1.9810 × 10^0^	1.0069 × 10^−7^	1.4298 × 10^0^	1.3238 × 10^−7^	1.4773 × 10^0^

**Table A9:** Estimated parameters of the PVM in the scaled parameterization.

Algorithm	*I_ph_* (A)	*I_sd_* (A)	*R_s_* (Ω)	*R_sh_* (Ω)	*n*
SSMO	2.0592 × 10^−1^	7.5753 × 10^−7^	1.9887 × 10^0^	1.9053 × 10^3^	1.6322 × 10^1^
PSO	2.0731 × 10^−1^	4.8087 × 10^−6^	1.5220 × 10^0^	2.0000 × 10^3^	1.9151 × 10^1^
GWO	2.0938 × 10^−1^	1.1037 × 10^−6^	1.8603 × 10^0^	5.9946 × 10^2^	1.6852 × 10^1^
WOA	2.0894 × 10^−1^	6.4566 × 10^−6^	1.4851 × 10^0^	1.4298 × 10^3^	1.9693 × 10^1^
DBO	2.0599 × 10^−1^	7.7022 × 10^−7^	2.0000 × 10^0^	2.0000 × 10^3^	1.6342 × 10^1^
GWCA	2.0648 × 10^−1^	1.6417 × 10^−6^	1.8174 × 10^0^	2.0000 × 10^3^	1.7397 × 10^1^
SGA	2.1215 × 10^−1^	4.6670 × 10^−5^	4.4422 × 10^−1^	1.9627 × 10^3^	2.4308 × 10^1^
IVY	2.0787 × 10^−1^	1.9281 × 10^−6^	1.5561 × 10^0^	6.7829 × 10^2^	1.7677 × 10^1^
FATA	2.0739 × 10^−1^	6.3914 × 10^−6^	1.3049 × 10^0^	1.8377 × 10^3^	1.9695 × 10^1^
PLO	2.0841 × 10^−1^	8.0382 × 10^−6^	1.3439 × 10^0^	1.6456 × 10^3^	2.0121 × 10^1^
SFOA	2.0749 × 10^−1^	9.5860 × 10^−6^	1.1228 × 10^0^	1.1357 × 10^3^	2.0514 × 10^1^
EMWPA	2.0626 × 10^−1^	1.1194 × 10^−6^	1.9053 × 10^0^	1.9146 × 10^3^	1.6848 × 10^1^
